# Germaborene reactivity study – addition of carbon nucleophiles, cycloaddition reactions, coordination chemistry†

**DOI:** 10.1039/d4sc03743j

**Published:** 2024-06-21

**Authors:** Christian Reik, Lukas W. Jenner, Hartmut Schubert, Klaus Eichele, Lars Wesemann

**Affiliations:** a Institut für Anorganische Chemie Auf der Morgenstelle 18 72076 Tübingen Germany lars.wesemann@uni-tuebingen.de

## Abstract

^Me^NHC substituted germaborenium cation 2 was synthesized directly in reaction of bromo-substituted germaborene 1b with ^Me^NHC. The adamantyl isonitrile substituted germaborenium cation 4 was obtained stepwise: substitution of the chloride atom against adamantyl isonitrile at the B–Cl unit in 1a, simultaneous migration of the chloride to the germanium atom followed by chloride abstraction using Na[BAr^F^_4_] gives the germaborenium cation 4. Substitution of the bromide atom in 1b against carbon monoxide followed by bromide abstraction using Ag[Al(O*t*Bu^F^)_4_] leads to compound 6 exhibiting a B=C double bond substituted at the boron atom by a germylium cation. Treating the germaborene [Ge=B–Ph] (1c) with selenium, a cycloaddition product 7 was characterised featuring a GeBSe heterocycle. Carbon dioxide reacts with 1b to give a four membered ring molecule 8 as the product of a B–C and Ge–O bond formation. In reaction of 1b with dimethylbutadiene, a product 9 of a [2 + 4] cycloaddition was isolated. Transition metal fragments [Fe(CO)_4_ (10), CuBr (11), AuCl (12)] show coordination at the germaborene double bond. Molecular structures of the germaborene coordination compounds 10–12 are presented and the ligand properties are discussed. After treating the germaborene [Ge=B–Br] (1b) with [Cp*Al]_4_, insertion of a Cp*Al moiety into the B–Br bond was found (13).

## Abstract 2

Germaborene chemistry is presented: halide substitution by carbon nucleophiles, Cp*Al insertion into the B–Br bond, cycloaddition of CO_2_, dimethylbutadiene and selenium and coordination of CuBr, AuCl and Fe(CO)_4_ fragments at the B=Ge double bond.

## Graphical abstract

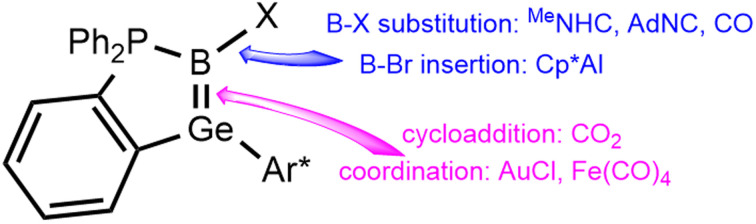

## Introduction

In main group element chemistry, studies of boron-element double bonds are an attractive area of research.^1–3^ In addition to the development of a synthesis strategy, studies on the reactivity of the [B=E] unit and the investigation of the electronic structures of the unsaturated molecules are rewarding challenges in molecular chemistry. Research in this area has led to the presentation of many examples of boron-element double bonds: B=B,^4–8^ B=C,^9–11^ B=Si,^12–16^ B=Ge,^17–19^ B=Sn,^20^ B=N,^21–29^ B=P,^30–34^ B=As,^33,35^ B=O,^36–41^ B=S,^41–43^ B=Se,^36,41,43,44^ and B=Te.^41,44^ Our research is focused on the chemistry of heavy elements of the group 14. While compounds featuring the B=C-double bond have been known for more than forty years, Sekiguchi *et al.* presented the first example for a B=Si double bond in 2006.^12^ The reactivity of the B=C double bond has been of major interest since it was first synthesized. Research groups of Berndt, Nöth and Paetzold studied the chemistry of B=C double bonds intensively and presented results of [2 + 2], [2 + 3]-cycloaddition reactions with ketones, alkynes, iminoboranes, and azides.^10,45–47^ Furthermore, reactions of various reagents like HCl, Br_2_, MeLi, MeBBr_2_, *t*BuNC, HNMe_2_ with the B=C bond were presented. In these reactions, the boron atom reacts as an electrophile, adding, for example, the [CH_3_]^−^ or [Cl]^−^ anion, and the carbon atom of the B=C unit shows the reactivity of a nucleophile. Thus, addition of MeBBr_2_ leads to formation of B–Br and C–BMeBr units.^47^ In the current literature, anionic borataalkenes serve as borata-Wittig olefination reagents and borataalkenes were investigated as π-ligands in organometallic chemistry.^48–54^ The chemistry of Lewis base stabilized alkylideneboranes is also studied intensively with respect to B=C bond cleavage, 1,2-dipolar reactivity studies and cycloaddition reactions.^48,55–63^

**Scheme 1:**
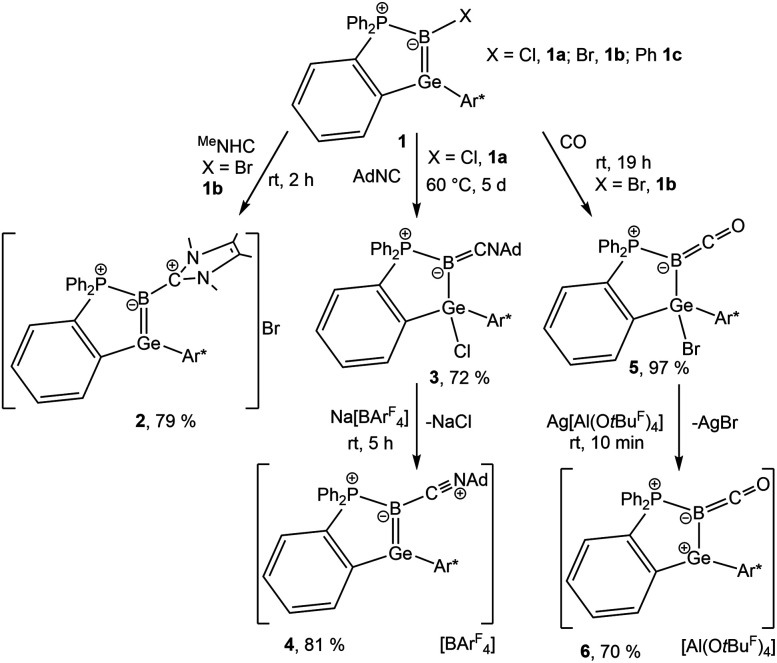
Reactions of germaborene (1a X = Cl, 1b X = Br, 1c X = Ph) with carbon-based nucleophiles ^Me^NHC, AdNC and CO (Ar* = 2,6-Trip_2_C_6_H_3_, Trip = 2,4,6-triisopropylphenyl).

Borasilene was reacted with elemental sulphur and selenium to give the three-membered BSiS and BSiSe heterocycles.^16^ Treating borasilene with oxygen, a splitting of the double bond was observed and 1,3,2,4-dioxasilaboretane was isolated.^16^ The electrophilicity of the boron atom in the borasilene B=Si unit was demonstrated in reaction of the borasilene with lithium trimethylsilylacetylide adding the anionic acetylide at the boron atom.^14^ The chloride adduct of borasilene exhibits an intramolecular C–H addition reaction under formation of Si–H and B–C bonds.^13^ This anionic borasilene adduct also shows a reaction with sulphur to give 1,3,2,4-dithiasilaboretane.^13^ As an interesting addition to the field of B–Si multiple bonds, examples for 2π-aromatic disiladiboretenes exhibiting a planar geometry were published recently.^15,64^ Kinjo *et al.* presented the synthesis of an allene type linear [Ge=B=N] molecule exhibiting the first Ge=B double bond in 2020.^17^ At the same time, we presented another approach to the synthesis of germaborenes by treating an intramolecular phosphine-germylene Lewis pair with boron trihalides (BCl_3_, BBr_3_) followed by Mg reduction.^18^ The germaborenes 1a, b (Scheme 1) are light sensitive compounds and react at room temperature with light of 530 nm wavelength in a [2 + 2] cycloaddition reaction with a Trip moiety of the terphenyl substituent Ar* (Ar* = 2,6-Trip_2_C_6_H_3_, Trip = 2,4,6-triisopropylphenyl). This reaction, which has been the only example for a cycloaddition reaction of germaborenes so far, is reversible by irradiation with light of 366 nm wavelength, recovering the starting material.^18^ Germaborene 1a, 1b and also the phenyl substituted derivative 1c were shown to react as a source of borylenes [BX] (X = Cl, Br) and [BPh] in reaction with azides RN_3_ (R = SiMe_3_, adamantyl) to yield iminoborane derivatives.^65^ In analogy to the synthesis of 1, we applied an intramolecular stannylene Lewis pair to this procedure to give the first examples of stannaborenes realized in a stannaborenyl anion and a stannaborenium cation.^20^ The stannaborenium cation adds ammonia at the Sn=B double bond to give a B–H and Sn–NH_2_ unit.^20^

We set up the first systematic reactivity study of germaborenes because in terms of orbital overlap and therefore bonding energy, the germaborene consists of a less favourable element combination, which should result in high reactivity. Furthermore, the different substituents on the boron atom in 1 allow to study the influence of the substituents on the reactivity. Since the chemistry of the homologous boraalkenes and borasilenes has been reported, a comparative study with germaborene reactivity is of interest. Finally, the germaborene can be synthesized straightforwardly in a yield of up to 75% from up to 500 mg starting material making a study of germaborene 1 chemistry possible. We present reactions of germaborene with carbon-based nucleophiles leading to unprecedented germaborenium cations. So far unknown cycloaddition reactions with selenium, carbon dioxide and dimethylbutadiene are presented, and results are compared with the chemistry of boraalkenes and borasilenes. The coordination chemistry of the Ge=B moiety is discussed in iron tetracarbonyl and coinage metal complexes and the ligand properties are compared in view of olefin and boraalkene coordination compounds. An insertion reaction of [Cp*Al]_4_ into the B–Br unit of the germaborene is shown as an example for an electropositive substituent on boron.

## Results and discussion

Halide substituted germaborenes 1 (a: X = Cl, b: X = Br) were treated with ^Me^NHC, AdNC and CO (Scheme 1). Interestingly, the Lewis bases do not attack the germanium atom and instead form a bond with the boron atom although the Ge–B σ- and π-bonds in the germaborene are polarized toward the boron atom.^18^ At room temperature, the heterocyclic carbene reacts at the boron atom to directly yield a so far unknown ^Me^NHC-substituted germaborenium cation (2) (Scheme 1). The isonitrile and CO show a stepwise reaction with the halide substituted germaborene. In both cases, a carbon–boron bond was formed, and the halide migrates to the germanium atom. Obviously, the isonitrile is less reactive and heating to 60 °C is necessary for product formation. The carbon monoxide reaction product (5) however, also featuring a halide migration to the germanium atom, is formed at room temperature. Both halides 3 and 5, showing a short B–C interaction, were transformed to the cations 4 and 6 by halide abstraction using Na[BAr^F^_4_] or Ag[Al(O*t*Bu^F^)_4_] (Scheme 1).^66,67^

**Fig. 1:**
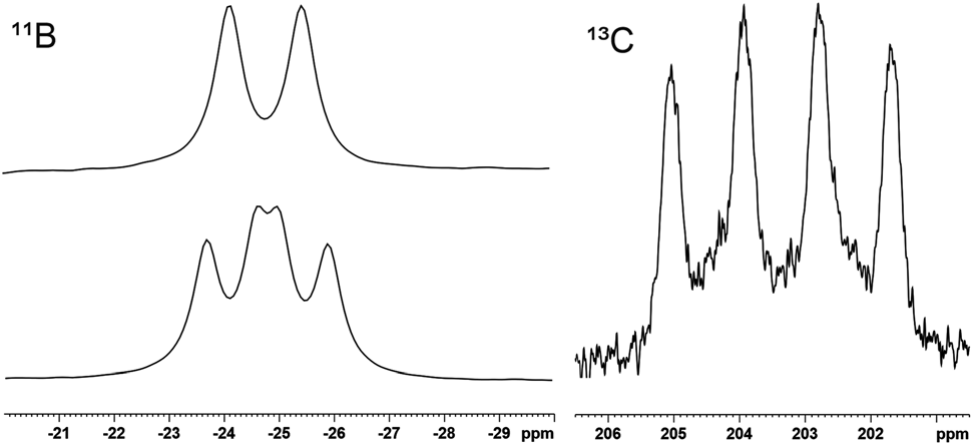
^11^B NMR (128.37 MHz) (top left, natural abundance 6: d ^1^*J*_^31^P–^11^B_ = 168 Hz; bottom left, ^13^CO labelled sample of 6: dd, ^1^*J*_^31^P–^11^B_ = 160.1 Hz, ^1^*J*_^13^C–^11^B_ = 119.3 Hz) and ^13^C NMR (100.62 MHz) carbonyl region (right) of cation 6, ^13^CO labelled (^1^*J*_^11^B–^13^C_ = 111.6 Hz).

Compounds 2–6 were characterised by NMR spectroscopy and selected signals are listed in Table 1. In the ^11^B NMR spectrum the adducts 3 and 5 show a signal at lower frequency in comparison to the cationic products of halide abstraction 4 and 6. ^13^C NMR signals of the CO-substituent at the boron atom in 5 and 6 were only observed with ^13^CO-gas (220.4 ppm 5, 203.4 ppm 6, see Fig. 1 and SI for spectra).

**Table 1:** Selected NMR data of compounds 1–6^a^

	^11^B *δ* [ppm] (^1^*J* B–P [Hz])	^31^P *δ* [ppm] (^1^*J*^11^B–P [Hz])
1a, b, c^18,65^	17.3, 10.3, 16.2	5.2, 7.3, 12.6
2	0.1 (d, 127.9 Hz)	17.9 (br)
3	−21.4 (d, 115.0 Hz)	27.8 (br)
4	−13.4 (d, 149.0 Hz)	28.5 (q, 152.4 Hz)
5	−39.7 (d, 121.3 Hz)	40.1 (br)
6	−24.8 (d, 168.2 Hz)	32.3 (q, 166.1 Hz)

a q: non-binomial quartet, br: unresolved quartet.

**Fig. 2:**
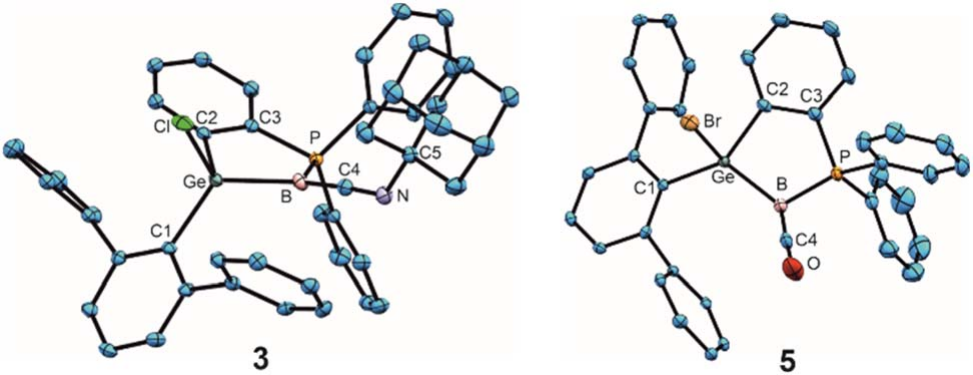
ORTEPs of the molecular structures of adducts 3 and 5. Thermal ellipsoids are shown at 50% probability level. Hydrogen atoms and iPr groups have been omitted (Table 2).

Adducts 3 and 5 exhibit B–C bond lengths of 1.433(3) and 1.418(3) Å which are comparable with molecules showing B=C double bonds [1.401(5)–1.475(8)] and are short distances in comparison with the group of low valent boron isonitrile [1.420(6)–1.569(3) Å]^59,68–71^ and carbon monoxide [1.445(3)–1.492(4) Å]^58,59,70,72–76^ adducts (Fig. 2, molecular structure of 3 and 5).^77–81^ The sum of angles around the boron atom of adduct 3 is with 359.1(1)° close to 360° which can be interpreted as an indicator for delocalisation of the boron electron pair into the B=C double bond. In the case of the CO-adduct 5, however, a smaller angle of 353.7(2)° around the boron atom, and therefore a slight pyramidalization, was observed, which indicates a partially localized electron pair on the boron atom.

**Table 2:** Selected interatomic distances [Å] and angles [°] of 1a, 2–6

	Ge–B	B–C4	C4–E	Ge–B–P	Ge–B–C4	P–B–C4
1a^18^	1.886(2)			103.1(1)		
2	1.890(2)	1.562(3)		102.6(1)	134.6(2)	122.8(2)
3	2.016(1)	1.433(3)	E = N 1.212(2)	105.1(1)	134.1(1)	119.9(1)
4	1.912(3)	1.483(4)	E = N 1.155(3)	104.2(1)	133.3(2)	122.4(2)
5	1.999(2)	1.418(3)	E = O 1.158(3)	106.8(1)	130.7(2)	116.2(2)
6	1.931(2)	1.437(3)	E = O 1.143(3)	106.2(1)	133.6(2)	120.0(2)

**Fig. 3:**
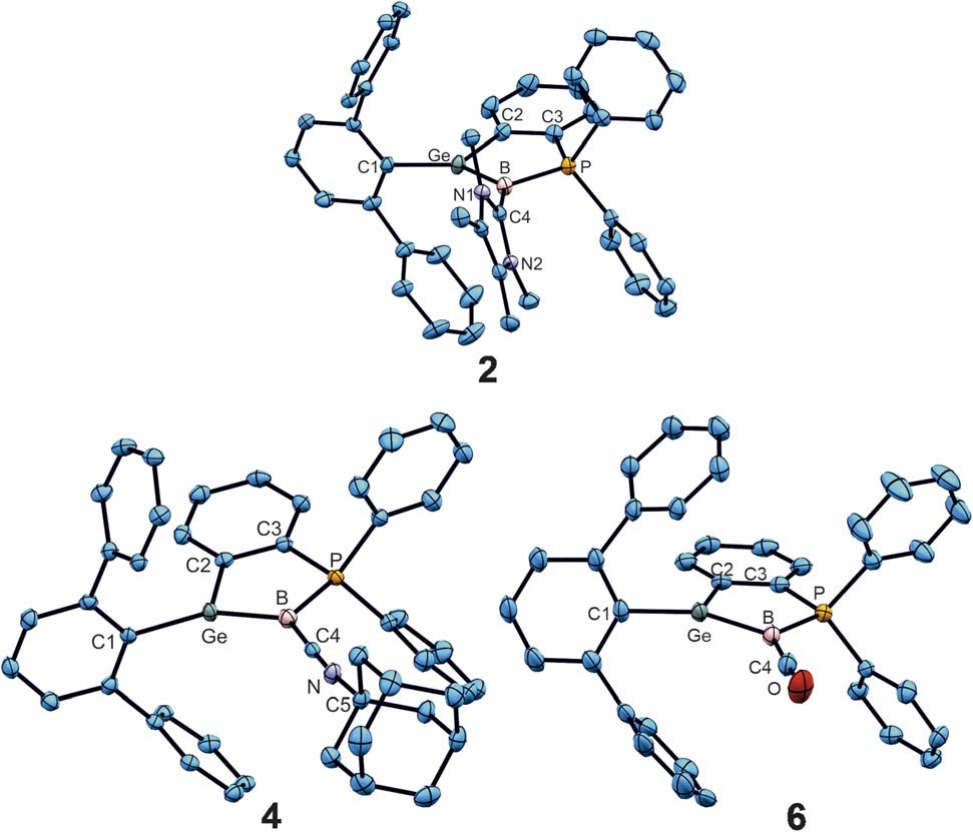
ORTEPs of the molecular structures of cations 2, 4 and 6. Thermal ellipsoids are shown at 50% probability level. Hydrogen atoms, iPr groups and the anions have been omitted (Table 2).

In the series of cationic Lewis-base adducts 2, 4 and 6 (Fig. 3) the B–C interatomic distances [1.562(3), 1.483(4), 1.437(3) Å] decrease while the Ge–B [1.890(2), 1.912(3), 1.931(2) Å] bond lengths increase. ^Me^NHC-adduct 2 features a short Ge=B double bond^17,18,65,82^ and a B–C single bond with the NHC-donor.^83–85^ Apparently for steric reasons, an angle of 74.6° was found between the [^Me^NHC-ligand] and [GeBPC2C3] planes in compound 2, which makes π-back donation from the boron atom to the NHC-molecule less favourable. The isonitrile- and CO-donor cations (4, 6) (Fig. 3) show shorter B–C and longer Ge–B interatomic distances in comparison to cation 2. The isonitrile donor in 4 shows less back bonding from the boron atom to the carbon atom C4 upon cationization which goes along with a larger angle at the nitrogen atom C4–N–C5 [3: 127.6(2), 4: 175.0(3)°] (Fig. 3) and a longer B–C bond together with a shorter C–N bond in comparison to 3. The B–C–N bond length found in 4 are comparable with isonitrile adducts of low valent boron compounds [B–C: 1.420(6)–1.569(3); C–N 1.152(3)–1.243(3) Å].^59,68–71^ The B–C distance in 6 is comparable with a long double bond between these elements.^77–81^ CO adducts of low valent boron compounds exhibit longer B–C bond lengths [1.445(3)–1.492(4) Å].^58,59,70,72–76^ The IR stretching frequencies for the CO unit in 5 (1984 cm^−1^) and 6 (2024 cm^−1^) reflect a considerable amount of π-back donation by the borylene boron atom. CO adducts of borylenes like [(DippNC)(OC)BTp] (1930, 2094 cm^−1^) and [(OC)_2_BTp] (1942, 2060 cm^−1^) exhibit comparable CO frequencies [Dipp = 2,6-diisopropylphenyl, Tp = 2,6-di(2,4,6-triisopropylphenyl)phenyl].^70,72,74,76,86^ The direct substitution of a bromide substituent at a low valent boron atom against a carbon monoxide was observed by Xie *et al.* reacting a bissilylene stabilized bromoborylene with tungsten hexacarbonyl Table 2.^76,87^

**Scheme 2:**
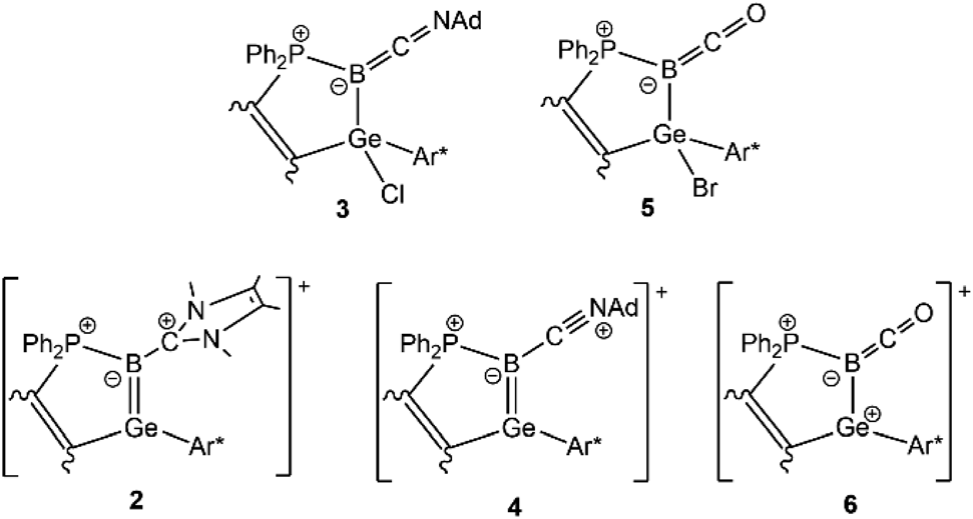
Leading Lewis structures for adducts 3, 5 and cations 2, 4, 6.

**Fig. 4:**
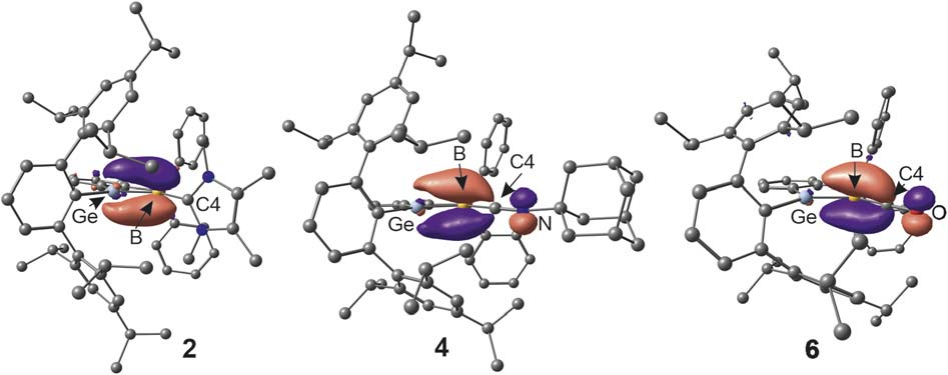
HOMOs representing the Ge–B π-bond in 2 and 4 and B–C π-bond in 6 (contour value 0.062).^88^

To evaluate the electronic situations in the molecules 2–6, DFT calculations [BP86/ωB97X-D3BJ, def2-SVP/TZVP(Ge,B,C4)] together with NBO analysis (Table 3) have been carried out on the basis of the solid state molecular structures (see ESI†). The leading Lewis structures of compounds 2–6 are shown in Scheme 2 and the HOMOs of cations 2, 4 and 6 are depicted in Fig. 4.

**Table 3:** Selected results of NBO calculations of 2, 3, 5, 6 BP86/D3BJ, 4 wB97X-D3; def2-SVP/TZVP (Ge, B, P, C4, O)

	2	3	4	5	6
Ge–B [Å]	1.89855	2.00338	1.89128	1.99776	1.94257
*q* [e] Ge, B	1.25, −0.76	1.39, −0.83	1.54, −1.04	1.32, −1.00	1.51, −0.99
Wiberg/Löwdin	1.51/1.60	0.87/1.11	1.34/1.49	0.904/1.14	1.10/1.34
σ-bond occ.	1.9069	1.8645	1.9180	1.8688	1.8710
Ge–B% (NBO)	43, 57	41, 59	40, 60	40, 60	40, 60
π-bond occ.	1.7099	1.7489	1.6285	1.7397	1.6547
Ge–B% (NBO)	37, 63		27, 73		
B–C% (NBO)		55, 45		59, 41	60, 40
σ-bond occ.	1.9625	1.9661	1.9671	1.9757	1.9754
B–C% (NBO)	34, 66	38, 62	37, 63	39, 61	39, 61
B–C [Å]	1.54180	1.42172	1.47599	1.42612	1.43407
Wiberg/Löwdin	0.96/1.32	1.56/1.71	1.19/1.45	1.51/1.72	1.44/1.63

In all cases, the Ge–B σ-bond shows a slight polarisation towards the boron atom and the B–C σ-bond is polarized to the carbon atom. In the case of the adducts 3 and 5, a B=C π-bond was observed exhibiting a polarisation to the boron atom. To a small extent, the B=C π-bond of 5 exhibits hyperconjugation with the Ge–Br σ*-bond. The cations 2 and 4 exhibit a Ge–B π-bond with a polarisation toward the boron atom, which is more distinctive for the isonitrile adduct 4. Thus, 2 and 4 are examples for unprecedented germaborenium cations. The bonding situation in ^Me^NHC-adduct 2 can be compared with the homologous ^Me^NHC-supported stannaborenium cation, featuring a Sn=B double bond.^20^ The CO-cation 6 however, features a B–C π-bond, which is polarized to the boron atom. Obviously the π-accepting character of the CO-ligand dominates the delocalisation of the electron pair. The formation of the germyl cation in 6 does not lead to formation of a Ge=B double bond like in the case of 4. The difference between isonitrile *versus* CO delocalisation of the electron pair at a low valent boron atom can be compared with the electronic situation found in [Ar*B(CO)CNDipp].^86,89^

**Scheme 3:**
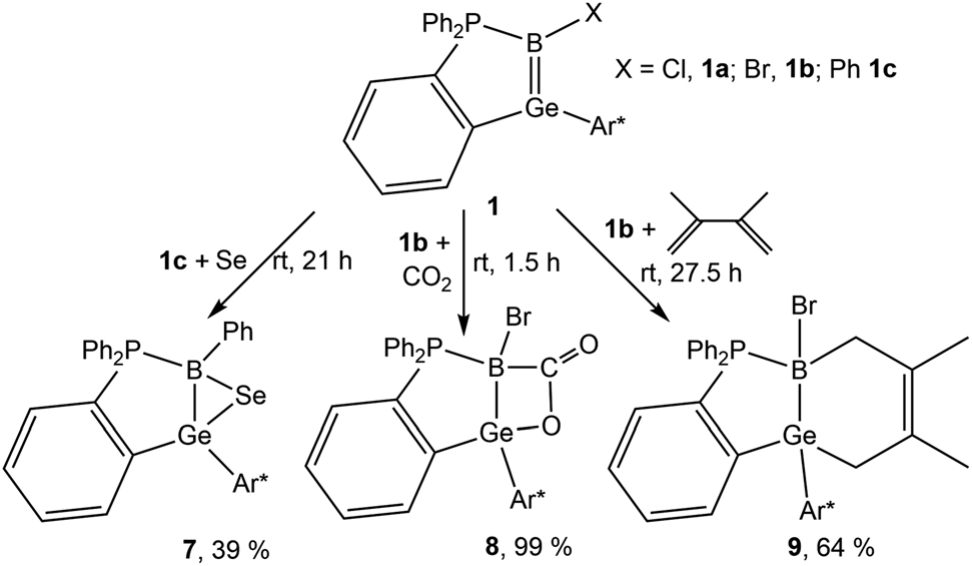
Reactions of germaborene 1b (X = Br)^18^ and 1c (X = Ph)^65^ with selenium, carbon dioxide and dimethylbutadiene (Ar* = 2,6-Trip_2_C_6_H_3_, Trip = 2,4,6-triisopropylphenyl).

**Fig. 5:**
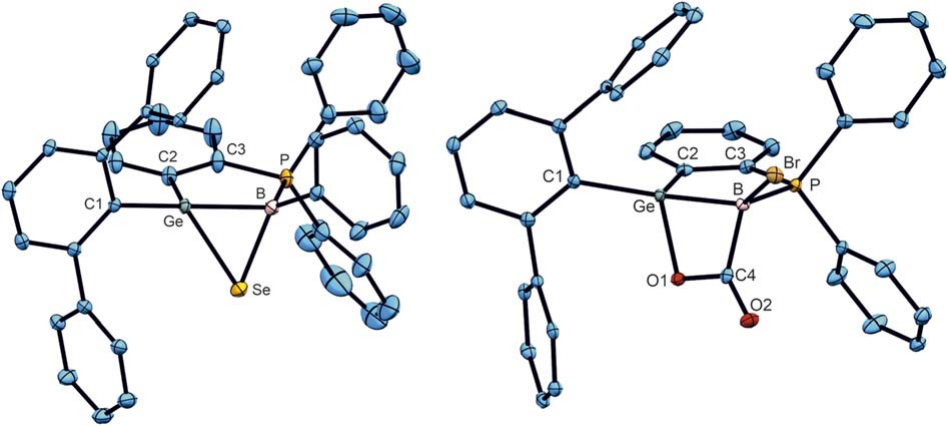
ORTEPs of the molecular structures of 7 and 8. Thermal ellipsoids are shown at 50% probability level. Hydrogen atoms and iPr groups have been omitted. Interatomic distances in Å and angles in (°). 7: Ge–B 2.0570(17), Ge–Se 2.3576(2), B–Se 2.1012(17), B–P 1.9601(18), Ge–Se–B 54.6(1), Ge–B–Se 69.1(1), Se–Ge–B 56.4(1); 8: Ge–B 2.085(2), Ge–O1 1.8969(14), B–P 1.971(2), B–C4 1.629(3), C4–O2 1.210(3), O1–Ge–B 74.0(1), C4–B–Ge 81.9(1), O1–C4–O2 121.7(2), O2–C4–B 132.2(2); 9: Ge–B 2.1089(17), B–C7 1.626(2), C6–C7 1.521(2), C5–C6 1.342(2), C4–C5 1.507(2), Ge–C4 1.9842(15).

A cycloaddition reaction of germaborenes has been only reported in the case of the intramolecular reversible [2 + 2] addition between Ge=B double bond and an arene ring of the terphenyl substituent.^18^ To further investigate the reactivity of the Ge=B double bond in germaborenes, reactions with selenium, carbon dioxide and dimethylbutadiene were carried out. Selenium reacts at room temperature with the germaborene 1c and formation of a so far unknown GeBSe-heterocycle was characterised by single crystal structure analysis (Scheme 3, Fig. 5) and shows a signal in the ^77^Se NMR spectrum at −386.8 ppm. This type of addition was reported for the homologues boraalkene and borasilene and also for digermenes and diborenes leading to the corresponding three-membered ring molecules.^16,62,90–94^ The Ge–B bond in 7 shows an elongation [2.0570(17) Å] compared to the starting material and is close to the value of a Ge–B single bond [2.095(5) Å].^18^ In 7, a Ge–Se bond [2.3576(2) Å] and a B–Se bond [2.1012(17) Å] were formed. Both bond lengths and the angle at the Se atom of 54.6(1)° are comparable with distances and angles found in the cycles [CBSe: B–Se 2.097(5), C–Se–B 45.5(2); SiBSe: B–Se 1.963(3), Si–Se–B 54.9(1); Ge_2_Se: Ge–Se 2.3961(4), 2.4017(4) Å, Ge–Se–Ge 59.2(1); B_2_Se: B–Se 2.115(2), 2.063(2), 2.073(2), 2.102(5), 2.039(6) Å, B–Se–B: 50.03(9)°].^90–93^ The signal found for 7 in the ^77^Se NMR spectrum at −386.8 ppm lies in the range of signals found for comparable three membered ring molecules: CBSe −317.0, −453.4, −368.1; SiBSe −400.7; Ge_2_Se −331.0; B_2_Se −361.5 ppm.^16,62,90,93^

Treating the germaborene 1b with carbon dioxide, the product of a [2 + 2] cycloaddition, the first example for a BGeOC heterocycle, was obtained (Scheme 3 and Fig. 5). A B–C 1.629(3) and a Ge–O 1.8969(14) Å single bond were formed and the Ge–B bond length [2.085(2) Å] is elongated and comparable with a single bond between these elements.^18,83,84^ The homologous boraalkene adds CO_2_ under formation of a C–C and B–O bond.^62^ In the boraalkene the boron atom reacts as an electrophile and in the germaborene the boron atom exhibits nucleophilic reactivity. Further examples for carbon dioxide [2 + 2] cycloaddition reactions were presented for a variety of unsaturated low valent main group compounds like, *e.g.*, diborenes,^93,95–97^ dialumenes^98^ and disilenes.^99^

Treating the germaborene 1b with dimethylbutadiene, the product (9) of a [2 + 4] cycloaddition was isolated (Scheme 3), and the molecular structure is shown in the ESI.† In Table 4^11^B and ^31^P NMR data are listed. The shift of the ^11^B NMR signals to lower frequencies for compounds 7–9 can be explained with the increase of the coordination number in comparison to the starting material 1. The signal for the carbon atom at boron in the CO_2_-product 8 was observed at 184.2 ppm using ^13^CO_2_.

**Table 4:** Selected NMR data of compounds 7–14

	^11^B *δ*[ppm]	^31^P *δ*[ppm]
7	−18.1	6.5
8	−12.9	14.5
9	−9.7	14.6
10	−13.5	18.6
11	2.5	4.2
12	0.4	1.0
13	0.1	36.6
14	3.6	35.2

**Scheme 4:**
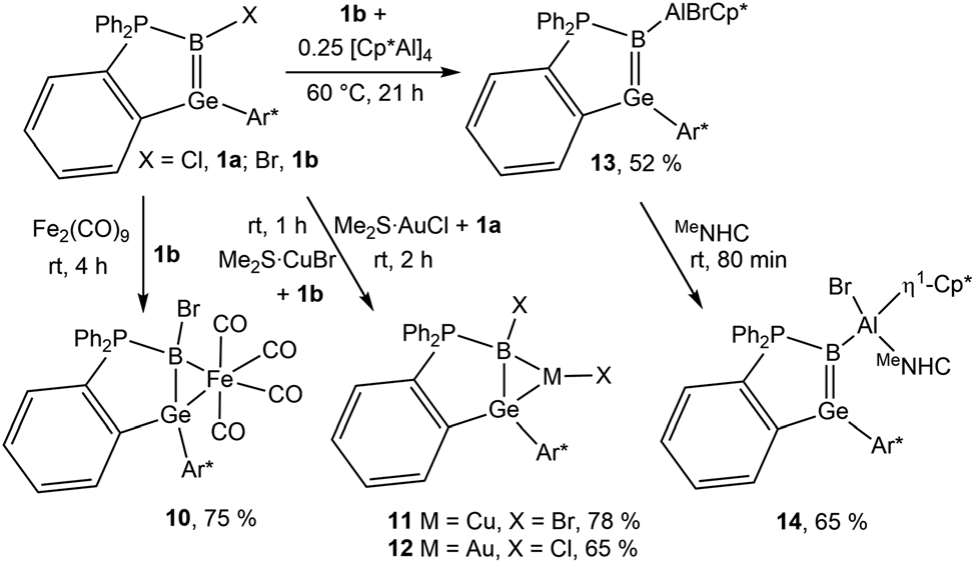
Reactions of germaborene with Fe_2_(CO)_9_, [Me_2_S·CuBr], [Me_2_S·AuCl] and [Cp*Al]_4_.

**Fig. 6:**
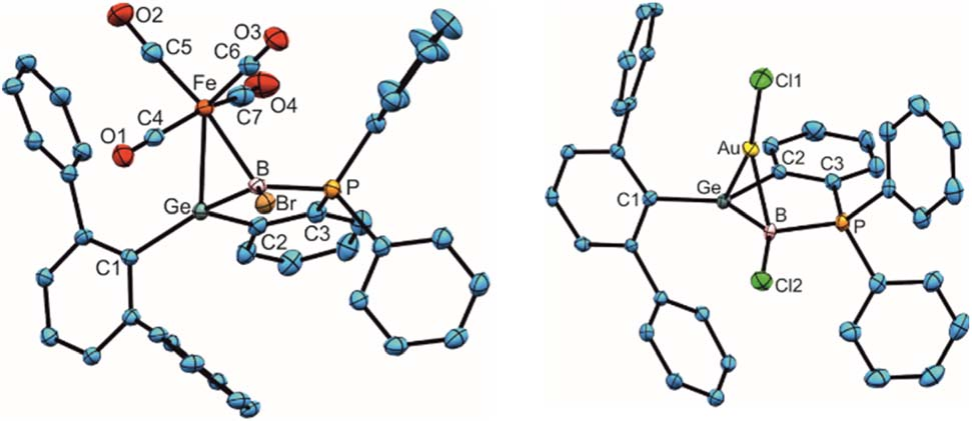
ORTEPs of the molecular structures of 10 and 12. Thermal ellipsoids are shown at 50% probability level. Hydrogen atoms and *i*Pr groups have been omitted. Interatomic distances in Å and angles in (°). 10: Ge–B 2.017(4), Ge–Fe 2.4718(6), Fe–B 2.257(4), Fe–C5 1.802(4), Fe–C6 1.792(4), Fe–C7 1.800(4), Fe–C4 1.797(4), B–P 1.930(4), B–Br 1.990(4), Ge–Fe–B 50.3(1), Fe–Ge–B 59.3(1), Fe–B–Ge 70.4(1), B–Fe–C5 170.8(2), B–Fe–C7 86.1(1); 11: Ge–B 1.9266(18), Cu–B 2.1049(18), Ge–Cu 2.4627(3), B–P 1.9112(17), Cu–Br1 2.2632(3), B–Br2 1.9421(18), B–Ge–Cu 55.7(1), B–Cu–Ge 49.1(1), Cu–B–Ge 75.2(1), Br2–B–Cu 106.9(1), Br1–Cu–Ge 164.7(1); 12: Ge–B 1.958(5), Ge–Au 2.5057(5), Au–B 2.195(5), B–P 1.945(5), Au–Cl1 2.3330(12), B–Cl2 1.780(5), Ge–Au–B 48.7(1), Au–B–Ge 74.0(2), B–Ge–Au 57.4(1).

In view of a known coordination chemistry of the homologous boraalkenes the ligand properties of the Ge=B double bond were tested in reactions with Fe_2_(CO)_9_, [Me_2_S·CuBr] and [Me_2_S·AuCl] (Scheme 4).^49–52^ First coordination compounds with the germaborene ligand were isolated and the molecular structures together with selected interatomic distances and angles are depicted in Fig. 6 (molecular structure of 11 is shown in the ESI†). Interatomic distances concerning the Fe-coordination at the Ge–B unit (Table 5) can be compared with the Fe–B distance found in the boraalkene Fe(CO)_4_ complex of amino-9-fluorenylideneborane [Fe–B: 2.125(5) Å].^49^ The Ge–Fe bond length lies in the range of germylene–iron coordination compounds [2.4112(3)–2.5970(3) Å].^100^ Copper and gold coordination at the germaborene (11, 12) can be compared with coordination of the coinage metals at homologous borataalkene which shows a slippage from *η*^2^ [Cu: Cu–B 2.12(2)], to *η*^1^ [Au: Au–B 2.23(1) Å].^53^ Cu–B and Au–B bond lengths can also be compared with diborene coordination compounds: [Cu–B 2.149(3), 2.146(3); Au–B 2.271(3), 2.354(2), 2.394(8) Å]^54,101,102^ The Cu–Ge bond length observed in 11 is slightly smaller than distances found for copper coordination at germanium cluster compounds [Cu–Ge 2.4752(4)–2.5043(4) Å].^103,104^ In the case of the found Au–Ge interatomic distance in 12 the bond length lies in the range of GeCl_3_ coordination at gold: Au–Ge 2.4150(6)–2.5351(7) Å.^105^

**Table 5:** Selected interatomic distances [Å] of 10–12

M	Ge–B	Ge–M	B–M
10 Fe	2.017(4)	2.4718(6)	2.257(4)
11 Cu	1.927(2)	2.4627(3)	2.105(2)
12 Au	1.958(4)	2.5057(4)	2.195(5)

In comparison to the starting material [1a: 1.886(2); 1b: 1.895(3) Å], an increase of the Ge–B bond length upon coordination of the metal fragments was found (Table 5). Coordination of the Fe(CO)_4_ fragment gives the largest elongation and for CuBr-coordination only a slight increase of the Ge–B bond length was observed. In the IR spectrum of the Fe(CO)_4_ complex of amino-9-fluorenylideneborane showing coordination of the iron fragment at a B=C bond the CO stretching frequencies were found at *ν* = 2064, 2011, 1962 cm^−1^. An olefin Fe(CO)_4_ complex was found to show CO wavenumbers at 2071, 2005, 1975 cm^−1^.^106^ The CO stretching frequencies of the germaborene Fe(CO)_4_ complex 10 were observed at slightly lower wavenumbers 2051, 1983 and 1960 cm^−1^ indicating the germaborene as a slightly better donor ligand in comparison to the amino-9-fluorenylideneborane boraalkene and olefin ligand.

**Fig. 7:**
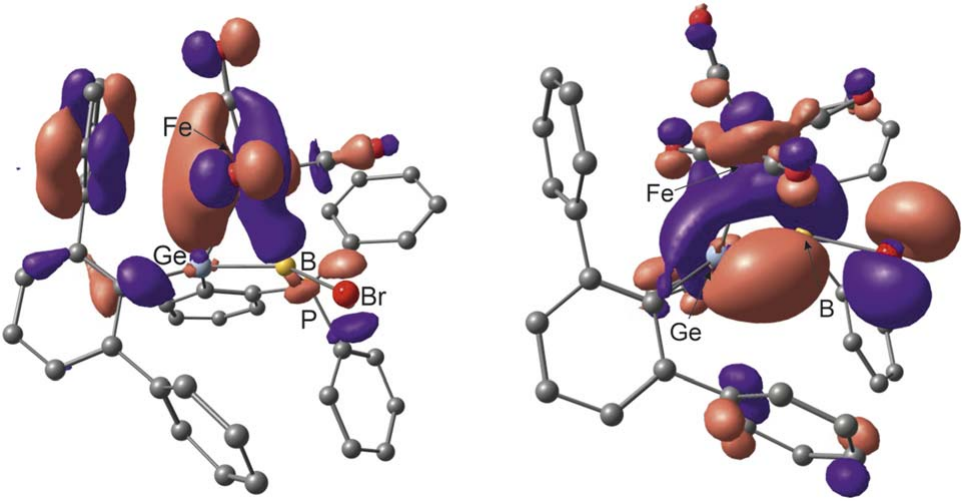
HOMO-1 and HOMO of 10 representing the π-acceptor and σ-donor interaction between Ge–B double bond and Fe(CO)_4_ fragment (contour value 0.03).^88^

Investigated by DFT calculations and NBO analyses, the electronic situation of the coordination compounds can be discussed based on the Dewar–Chatt–Duncanson (DCD)^107,108^ bonding model (see Fig. 7 and Table SI3 in the ESI†). The HOMO of the Fe(CO)_4_ complex resembles the σ-donor component of the Fe–(Ge–B) interaction and the HOMO-1 the π-acceptor interaction (Fig. 7). The copper and gold complexes with a d^10^-valence electron count exhibit a small degree of π-back bonding in the π*-MO of the germaborene. The σ-donor interaction however, which can be described as a donation from the π-MO to the s-orbital of the metal, is more pronounced in the case of the gold coordination compound (Table SI3 in the ESI†).

**Fig. 8:**
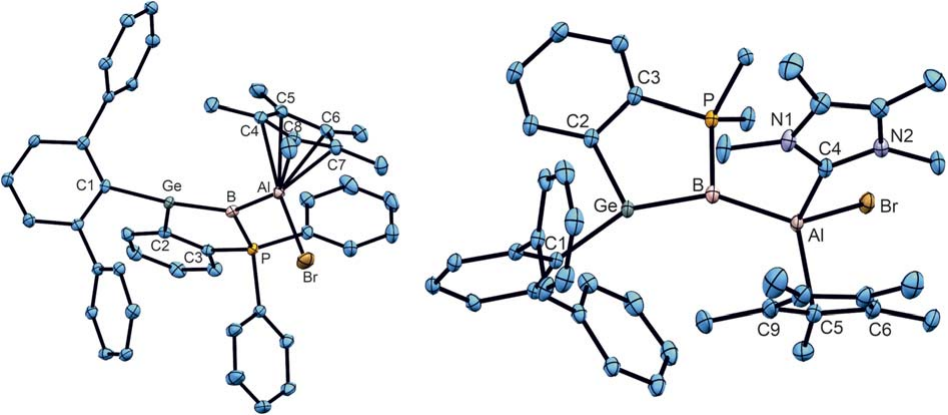
ORTEPs of the molecular structures of 13 and 14. Thermal ellipsoids are shown at 50% probability level. Hydrogen atoms and iPr groups have been omitted. Interatomic distances in Å and angles in (°). 13: Ge–B 1.901(2), B–P 1.8764(19), B–Al 2.052(2), Al–Br 2.3547(6), Al–C4 2.2833(18), Al–C5 2.396(2), Al–C6 2.303(2), Al–C7 2.190(2), Al–C8 2.1626(19), P–B–Ge 99.4(1), P–B–Al 119.2(1), Ge–B–Al 141.3(1), B–Al–Br 107.6(1); 14: Ge–B 1.9163(13), B–Al 2.1103(13), B–P 1.8885(14), Al–C4 2.0609(13), Al–Br 2.4328(4), Al–C5 2.0860(13), Ge–B–P 98.0(1), Ge–B–Al 145.3(1), Al–B–P 116.2(1), B–Al–Br 104.4(1).

In the case of the stannaborene, we recently presented a magnesium substituted [Sn=B–MgBr] stannaborene derivative.^20^ The change of polarity of the Ge=B–X bond in germaborene chemistry from halide (1a, 1b) or phenyl (1c) to magnesium or another electropositive substituent would make a new reactivity pattern at the boron atom of the Ge=B unit possible. Therefore, we studied the synthesis of the homologous magnesium derivative. However, so far, we cannot present a reliable procedure. To connect a less electronegative substituent at the boron atom, we also investigated the incorporation of an aluminium substituent reacting [Cp*Al]_4_ with germaborene 1b to a give a [Ge=B–Al] unit.^109–112^ In the final procedure, 1b was treated with [Cp*Al]_4_ in benzene at 60 °C for 21 hours to give the aluminium substituted product 13.^109–112^ The colour of the solution changed from red to orange and after evaporation of the solvent, crystals were obtained from *n*-pentane (yield 52%). The molecular structure of the insertion product 13 of a Cp*Al molecule into a B–Br bond is shown in Fig. 8. The electronic structure of 13 was analysed by DFT calculations together with NBO analysis. The B–Al σ-bond is polarised towards the boron atom: B 74.4%, Al 25.6% (σ-bond occ. 1.93 e^−^); to compare with 1b: B–Br: B 33.3%, Br 66.7% (σ-bond occ. 1.98 e^−^). However, the reactivity of this negatively charged GeB-unit should be checked in further investigations. In the following, a ^Me^NHC adduct (14) of this aluminium compound was synthesized showing a slippage from *η*^5^-to *η*^1^-coordination of the Cp* moiety at aluminium. The phenyl substituted germaborene 1c however, shows no reaction with [Cp*Al]_4_ at 60 °C in benzene. Reaction of a transient CAAC-adduct of phenylborylene with Cp^3t^Al [Cp^3t^ = *η*^5^-1,3,4-tri(*tert*-butyl)-cyclopentadienyl] results in the formation of B–Al bond [2.069(2) Å], which is discussed as an example for a B–Al multiple bond.^113,114^ Although short Al–B bond lengths were observed in 13 and 14 [13: 2.052(2), 14: 2.1103(13) Å], analyses of the electronic situations give no indications for a partial double bond character between boron and aluminium. Furthermore, the Ge–B distances show only a slight elongation in comparison to the germaborene starting material.^18^ Cationization by halide abstraction was not successful so far.

## Conclusions

Unprecedented germaborenium cations featuring a Ge=B double bond were isolated substituted by a ^Me^NHC or an adamantyl isonitrile ligand at the boron atom. In the ^Me^NHC case, this cation was obtained directly by reacting bromo substituted germaborene 1b with ^Me^NHC. The formation of the isonitrile substituted cation was realized in two steps: substitution of the chloride atom of the B–Cl unit in germaborene 1a against the adamantyl isonitrile going along with simultaneous transfer of the chloride to the germanium atom followed by chloride abstraction using Na[BAr^F^_4_]. Due to the strong π-acceptor properties of carbon monoxide, the analogous CO-substitution product at boron exhibits a B=C double bond substituted by a germylium cation at the boron atom.

In reaction of the phenyl substituted germaborene 1c with selenium, a so far unknown GeBSe heterocycle was isolated. Carbon dioxide reacts *via* a [2 + 2] cycloaddition reaction with bromo germaborene 1b. Bond formation between the electrophilic carbon atom and the nucleophilic boron atom gives a four membered GeBCO heterocycle. First coordination compounds with the Ge=B double bond were observed by coordination of the metal fragments [Fe(CO)_4_, CuBr, AuCl]. A comparison of IR data of analogue Fe(CO)_4_ complexes with boraalkene and olefin ligands allows a categorization of the ligands, with the germaborene being the slightly better donor ligand.

By insertion of a Cp*Al fragment into the B–Br bond of germaborene an electropositive substituent was introduced to germaborene chemistry giving the boron atom a putative nucleophilic character, which is to be verified in further germaborene chemistry studies.

## Data availability

Full experimental and computational details are provided as part of the ESI.†

## Author contributions

Investigations, writing, review C. R.; preparation of 2 L. W. J.; special NMR experiments K. E.; discussion and X-ray measurements H. S.; supervision, funding acquisition, DFT calculation, manuscript writing and review L. W.

## Conflicts of interest

There are no conflicts to declare.

## Supplementary Material

SC-015-D4SC03743J-s001

SC-015-D4SC03743J-s002
