# Supplementary material for: Germaborene reactivity study – addition of carbon nucleophiles, cycloaddition reactions, coordination chemistry
Source: Chem Sci. 2024 Jun 21;15(29):11358–66. doi: 10.1039/d4sc03743j (PMC11268465; doi:10.1039/d4sc03743j)
Supplement: SC-015-D4SC03743J-s001 [file SC-015-D4SC03743J-s001.pdf]

Electronic Supplementary Material (ESI) for Chemical Science.
This journal is © The Royal Society of Chemistry 2024

Supporting Information

Christian Reik, Lukas W. Jenner, Hartmut Schubert, Klaus Eichele, Lars Wesemann

Institut für Anorganische Chemie, Auf der Morgenstelle 18, 72076 Tübingen, Germany, E-Mail: lars.wesemann@uni-tuebingen.de

Content

Experimental Section ... 2

Syntheses ... 3

Data of crystal structure analyses ... 13

NMR spectroscopy ... 17

NMR spectra of compound **2**. ... 17

NMR spectra of compound **3**. ... 21

NMR spectra of compound **4**. ... 25

NMR spectra of compound **5**. ... 30

NMR spectra of compound **6**. ... 37

NMR spectra of compound **7**. ... 45

NMR spectra of compound **8**. ... 50

NMR spectra of compound **9**. ... 55

NMR spectra of compound **10**. ... 59

NMR spectra of compound **12**. ... 67

NMR spectra of compound **13**. ... 71

NMR spectra of compound **14**. ... 75

Quantum chemical calculations ... 79

References ... 119

Experimental Section

General procedures. All manipulations were carried out under argon atmosphere using standard Schlenk techniques and gloveboxes. Benzene was dried with activated aluminium oxide, *n*-pentane and *n*-hexane were obtained from a MBraun solvent purification system (SPS). All other solvents (Et_2O, THF, toluene, benzene-d_6) were distilled from a sodium-potassium alloy and like the previous mentioned solvents subsequently degassed by three freeze−pump−thaw cycles. Germaborenes **1a**, **1b**, **1c**, ^{Me}NHC, sodium tetrakis[3,5-bis(trifluormethyl)phenyl]borate (Na[BAr^F_4]), Ag[Al(OtBu^F)$_4$] and [Cp*Al]$_4$ were prepared according to a literature procedure (Ar^F = 3,5-bis(trifluoromethyl)phenyl; tBu^F = C{CF_3}$_3$). [1-7] Further chemicals were purchased commercially and used as received.

Elemental analysis. Elemental analysis was performed at the Institute of Inorganic Chemistry, University of Tübingen using an *elementar* vario MICRO Cube.

NMR spectroscopy. NMR spectra were recorded with either a Bruker Avance III HD 300 NanoBay spectrometer equipped with a 5 mm BBFO probe head and operating at 300.13 (^{1}H), 96.29 (^{11}B) and 57.23 MHz (^{77}Se), a Bruker AVII+400 NMR spectrometer equipped with Brukers 5 mm QNP (quad nucleus probe) or a 5 mm BBFO probe head operating at 400.11 (^{1}H), 100.61 (^{13}C) and 128.37 (^{11}B) MHz, a Bruker Avance III HDX 600 spectrometer equipped with a 5 mm Prodigy BBO cryo probe head operating at 600.13 (^{1}H) and 150.90 (^{13}C) MHz or a Bruker Avance III HDX 700 NMR spectrometer equipped with a 5 mm TXI probe head operating at 700.29 (^{1}H) and 176.9 (^{13}C) MHz. Chemical shifts are reported in δ values in ppm relative to external H_3PO_4 (^{31}P), 85%, $Al(NO_3)_3$ (^{27}Al), $SiMe_4$ (^{1}H, ^{13}C), $BF_3 \cdot OEt_2$ (^{11}B) or Me_2Se (^{77}Se) using the ^{2}H resonance of the deuterated solvent and using Ξ = 25.145020 % for ^{13}C, Ξ = 32.083 974 % for ^{11}B, Ξ = 94.094011 % for ^{19}F, Ξ = 26.056859 % for ^{29}Al and, Ξ = 40.480742 for ^{31}P, Ξ = 19.071513 for ^{77}Se.[8] The multiplicity of the signals is indicated as s = singlet, d = doublet, t = triplet, q = quartet, sept = septet, m = multiplet or br = broad/unresolved. For the assignment of proton and carbon signals detailed analysis of ^{1}H, ^{13}C{^{1}H}, ^{1}H−^{1}H COSY, ^{1}H−^{13}C HSQC, ^{1}H−^{13}C HMBC, ^{13}C{^{1}H} DEPT 135 and ^{1}H-^{1}H NOESY spectra was done.

Crystallography. X-ray data were collected with a Bruker Smart APEX II diffractometer with graphite-monochromated Mo-K$_\alpha$ radiation. The programs used were Bruker's APEX2 v2011.8-0, including SAINT for data reduction, SADABS for absorption correction, and SHELXS for structure solution, as well as the WinGX suite of programs version 1.70.01 or the GUI ShelXle, including SHELXL for structure refinement.

IR spectroscopy. The IR spectra were recorded as potassium bromide pellets, which were prepared in an MBraun glovebox and measured with a *Bruker* VERTEX 70 IR spectrometer.

Syntheses

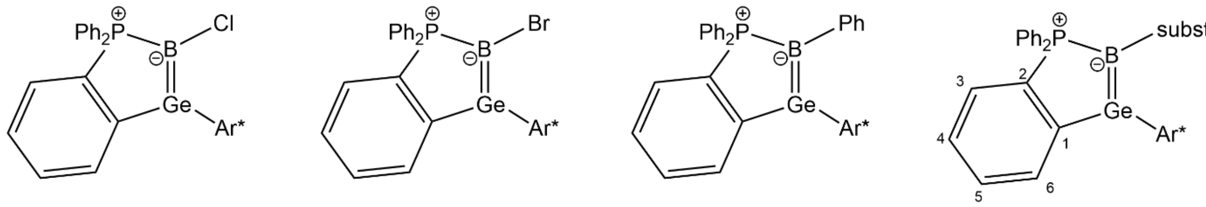

1a **1b** **1c** numbering for NMR

Scheme SI1. Used germaborenes.

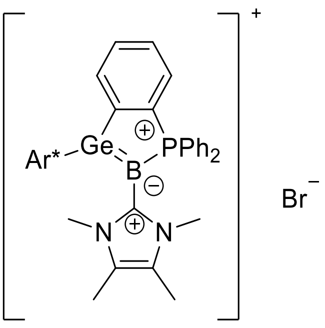

2: **1b** (100 mg, 110 µmol, 1 eq.) was dissolved in benzene (2 mL). ^{Me}NHC (12.8 mg, 103 µmol, 0.93 eq.) was dissolved in benzene (2 mL) and added dropwise to the solution of **1b** and stirred at room temperature. After two hours, the resulting orange solution was cooled to –38 °C and thereby frozen. The product was crystallized by layering pre-cooled *n*-pentane (– 38 °C, 6 mL) on top of the frozen solution and subsequently warming it up to room temperature. The obtained orange crystals were separated from the mother liquor and washed with *n*-pentane (5 mL). After drying under reduced pressure, **2** was isolated as an orange crystalline solid (89.9 mg, 87.2 µmol, 79 %). Single crystals suitable for X-ray diffraction could be obtained by vapor diffusion of *n*-pentane into a solution of **2** in benzene at room temperature. **^{1}H-NMR** (500.13 MHz, 273.2 K, *o*-$C_6D_4Cl_2$): δ (ppm) = 7.80 – 7.73 (m, 1 H, *p*-C_6H_3), 7.65 – 7.61 (m, 2 H, *m*-C_6H_3), 7.61 – 7.57 (m, 1 H, H-6), 7.57 – 7.52 (m, 2 H, *p*-C_6H_5), 7.51 – 7.44 (m, 9 H, H-5, *o*-C_6H_5, *m*-C_6H_5), 7.37 – 7.33 (m, 2 H, *m*-C_6H_2), 7.27 – 7.23 (m, 2 H, *m*-C_6H_2), 7.19 – 7.15 (m, 1 H, H-4, partly overlapping solvent signal), 7.14 – 7.09 (m, 1 H, H-3, partly overlapping solvent signal), 3.29 (sept, $^3J_{H\text{-}H}$ = 6.6 Hz, *o*-C$\underline{H}$(CH$_3$)$_2$), 3.06 (sept, $^3J_{H\text{-}H}$ = 6.9 Hz, *p*-C$\underline{H}$(CH$_3$)$_2$), 3.01 (s, 6 H, N-CH$_3$), 2.57 (sept, $^3J_{H\text{-}H}$ = 6.8 Hz, *o*-C$\underline{H}$(CH$_3$)$_2$), 2.17 (s, 6 H, C-CH$_3$), 1.40 – 1.35 (m, 12 H, *p*-CH(C$\underline{H}_3$)$_2$), 1.32 (d, $^3J_{H\text{-}H}$ = 6.5 Hz, 6 H, *o*-CH(C$\underline{H}_3$)$_2$), 1.14 (d, $^3J_{H\text{-}H}$ = 6.7 Hz, 6 H, *o*-CH(C$\underline{H}_3$)$_2$), 0.91 (d, $^3J_{H\text{-}H}$ = 6.8 Hz, 6 H, *o*-CH(C$\underline{H}_3$)$_2$), 0.86 (d, $^3J_{H\text{-}H}$ = 6.6 Hz, 6 H, *o*-CH(C$\underline{H}_3$)$_2$). **^{13}C{^{1}H}-NMR** (125.76 MHz, 273.2 K, *o*-$C_6D_4Cl_2$): δ (ppm) = 151 – 150.3 (m, C-1, C-carbene), 149.1 (s, *p*-C_6H_2), 147.7 (s, *o*-C_6H_2), 147.4 (s, *o*-C_6H_3), 146.9 (s, *o*-C_6H_2), 142.0 (d, $^3J_{31P\text{-}13C}$ = 19.9 Hz, *i*-C_6H_3), 137.7 (s, *i*-C_6H_2), 133.4 (d, $^1J_{31P\text{-}13C}$ = 87.6 Hz, C-2), 133.0 (d, $^3J_{31P\text{-}13C}$ = 14.2 Hz, C-6), 132.9 (s, C-5), 132.3 (s, *p*-C_6H_5), 131.7 (d, $^{2\text{ or }3}J_{31P\text{-}13C}$ = 10.4 Hz, *o*-C_6H_5 or *m*-C_6H_5), 130.7 (d, $^2J_{31P\text{-}13C}$ = 8.5 Hz, C-3), 129.9 (d, $^{2\text{ or }3}J_{31P\text{-}13C}$ = 11.2 Hz, *o*-C_6H_5 or *m*-C_6H_5), 129.5 (s, *p*-C_6H_3), 129.4 (s, *m*-C_6H_3), 127.8 (d, $^3J_{31P\text{-}13C}$ = 9.2 Hz, C-4), 127.6 (N$\underline{C}$=$\underline{C}$N, overlapping solvent signal, determined by HMBC experiment,), 126.1 (d, $^1J_{31P\text{-}13C}$ = 66.1 Hz, *i*-C_6H_5), 121.5 (s, *m*-C_6H_2), 120.5 (s, *m*-C_6H_2), 34.8 (s, *p*-$\underline{C}$H(CH$_3$)$_2$), 34.6 (s, NCH$_3$), 31.3 (s, *o*-$\underline{C}$H(CH$_3$)$_2$), 30.6 (s, *o*-$\underline{C}$H(CH$_3$)$_2$), 27.1 (s, *o*-CH($\underline{C}$H$_3$)$_2$), 25.7 (s, *o*-CH($\underline{C}$H$_3$)$_2$), 24.5 (s, *p*-CH($\underline{C}$H$_3$)$_2$), 24.4 (s, *p*-CH($\underline{C}$H$_3$)$_2$), 23.0 (s, *o*-CH($\underline{C}$H$_3$)$_2$), 22.7 (s, *o*-CH($\underline{C}$H$_3$)$_2$), 9.3 (s, C-$\underline{C}$H$_3$). **^{11}B{^{1}H}-NMR** (160.46 MHz, 273.2 K, *o*-$C_6D_4Cl_2$): δ (ppm) = 0.1 (d, $^1J_{31P\text{-}11B}$ =131.4 Hz). **^{31}P{^{1}H}-NMR** (202.46 MHz, 273.2 K, *o*-$C_6D_4Cl_2$): δ (ppm) = 17.9 (broad unresolved q). **Elemental analysis:** Calculated (%) for $C_{61}H_{75}BBrGePN_2$: 71.09 C, 7.34 H, 2.72 N Found: 71.45 C, 7.33 H, 2.69 N.

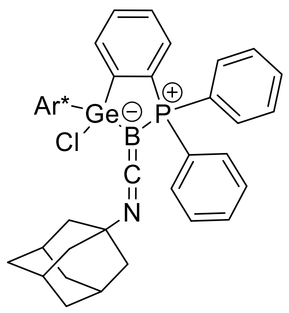

3: A solution of **1a** (100 mg, 116 µmol, 1 eq.) in benzene (2 mL) was added to 1-adamantyl isocyanide (18.7 mg, 116 µmol, 1 eq.) and stirred for 18 h at room temperature and subsequently heated to 60 °C for 5 days. The solution, initially intense red, turned orange during the reaction. The solvent was removed under reduced pressure and residual solvent in the residue was removed by co-evaporation with *n*-pentane (2 mL). The crude product was washed with *n*-pentane (2 × 2 mL, 1 × 4 mL) and after drying under reduced pressure, an orange powder of **3** (85.5 mg, 83.6 µmol, 72%) was obtained. Yellow single crystals suitable for X-ray diffraction were obtained by vapor diffusion of *n*-pentane into a concentrated solution of **3** in benzene at room temperature. **^{1}H-NMR** (400.11 MHz, 299.2 K, C_6D_6): δ (ppm) = 7.89 – 7.78 (m, 2 H, *o*-C_6H_5), 7.36 – 7.31 (m, 2 H, *m*-C_6H_2), 7.18 – 6.97 (m, 12 H, 2 × *o*-C_6H_5, 2 × *m*-C_6H_5, 1 × *p*-C_6H_5, 2 × *m*-C_6H_3, 2 × *m*-C_6H_2, *p*-C_6H_3, H-5, H-3, partly overlapping solvent signal), 6.86 – 6.78 (m, 3 H, 1 × *p*-C_6H_5, H-6, H-4), 6.75 – 6.67 (m, 2 H, 2 × *m*-C_6H_5), 3.36 – 3.08 (m, 4 H, *o*-C$\underline{H}$(CH_3)$_2$), 2.99 (sept, 2 H, $^3J_{H\text{-}H}$ = 6.9 Hz, *p*-C$\underline{H}$(CH_3)$_2$), 1.85 – 1.78 (m, 3 H, Ad-CH), 1.57 – 1.50 (m, 3 H, Ad-CH_2), 1.49 – 1.34 (m, 27 H, 6 × *o*-CH(C$\underline{H}_3$)$_2$, 12 × *p*-CH(C$\underline{H}_3$)$_2$), 9 × Ad-CH_2), 1.18 (d, 6 H, $^3J_{H\text{-}H}$ = 6.8 Hz, *o*-CH(C$\underline{H}_3$)$_2$), 1.03 (d, 6 H, $^3J_{H\text{-}H}$ = 6.8 Hz, *o*-CH(C$\underline{H}_3$)$_2$), 1.01 (d, 6 H, $^3J_{H\text{-}H}$ = 6.8 Hz, *o*-CH(C$\underline{H}_3$)$_2$). **^{13}C{^{1}H}-NMR** (100.62 MHz, 299.2 K, C_6D_6): δ (ppm) = 155.9 (d, $^2J_{31P\text{-}13C}$ = 25.0 Hz, C-1), 147.9 (s, *p*-C_6H_2), 146.9 (s, *o*-C_6H_2), 146.5 (s, *o*-C_6H_2), 146.3 (s, *o*-C_6H_3), 141.6 (d, $^3J_{31P\text{-}13C}$ = 9.1 Hz, *i*-C_6H_3), 139.3 (s, *i*-C_6H_2), 135.9 (d, $^3J_{31P\text{-}13C}$ = 13.1 Hz, C-6), 134.2 (d, $^1J_{31P\text{-}13C}$ = 77.5 Hz, C-2), 133.5 (d, $^1J_{31P\text{-}13C}$ = 66.2 Hz, *i*-C_6H_5), 133.0 (d, $^2J_{31P\text{-}13C}$ = 10.3 Hz, *o*-C_6H_5), 132.8 (d, $^2J_{31P\text{-}13C}$ =10.6 Hz, *o*-C_6H_5), 132.0 (s, m-C_6H_3), 130.9 – 130.7 (m, *p*-C_6H_5, C-3), 130.4 (d, $^1J_{31P\text{-}13C}$ = 67.8 Hz, *i*-C_6H_5), 130.4 (d, $^4J_{31P\text{-}13C}$ = 2.2 Hz, C-5), 129.9 (d, $^4J_{31P\text{-}13C}$ = *p*-C_6H_5), 128.3 (d, $^3J_{31P\text{-}13C}$ = 11.2 Hz, *m*-C_6H_5), 128.2 (d, $^3J_{31P\text{-}13C}$ = 11.2 Hz, *m*-C_6H_5), 127.6 (d, $^3J_{31P\text{-}13C}$ = 8.7 Hz, C-4, overlapping solvent signal), 127.2 (s, *p*-C_6H_3), 121.7 (s, *m*-C_6H_2), 121.0 (s, *m*-C_6H_2), 60.0 (d, $^4J_{31P\text{-}13C}$ = 6.1 Hz, Ad-C_q), 43.4 (s, Ad-CH_2), 36.2 (s, Ad-CH_2), 34.4 (s, *p*-$\underline{C}$H(CH_3)$_2$), 31.2 (s, *o*-$\underline{C}$H(CH_3)$_2$), 30.9 (s, *o*-$\underline{C}$H(CH_3)$_2$), 30.0 (Ad-CH), 25.8 (s, *o*-CH($\underline{C}H_3$)$_2$), 25.4 (s, *o*-CH($\underline{C}H_3$)$_2$), 24.3 (s, *p*-CH($\underline{C}H_3$)$_2$), 24.0 (s, *p*-CH($\underline{C}H_3$)$_2$), 23.4 (*o*-CH($\underline{C}H_3$)$_2$), 22.7 (s, *o*-CH($\underline{C}H_3$)$_2$). GeClB$\underline{C}$NAd not observed. **^{31}P{^{1}H}-NMR** (161.97 MHz, 299.2 K, C_6D_6): δ (ppm) = 27.8 (broad unresolved q). **^{11}B{^{1}H}-NMR** (128.37 MHz, 299.2 K, C_6D_6): δ (ppm) = –21.4 (d, $^1J_{31P\text{-}11B}$ = 115.7 Hz). **Elemental analysis:** Calculated (%) for $C_{67}H_{75}BBrGePN_2$: 76.30 C, 7.68 H, 1.37 N Found: 76.66 C, 7.83 H, 1.36 N. **IR (KBr):** 1893 cm^{-1} (ν C=N).

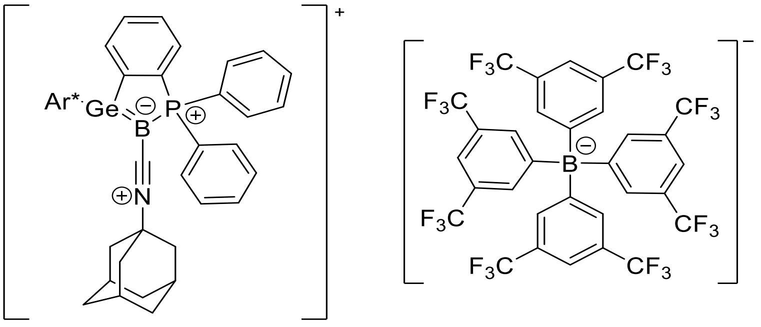

4: **3** (77.1 mg, 75.4 µmol, 1 eq.) and Na[BAr^F$_4$] (66.7 mg, 75.3 µmol, 1 eq.) were combined and stirred thoroughly to ensure a homogeneous distribution of both components. Benzene (1.5 mL) and *o*-difluorobenzene (1.5 mL) were added and the resulting mixture stirred for 5 h at room temperature. The resulting orange suspension was filtered, and the solvent was removed from the filtrate under reduced pressure. Residual solvent was removed by co-evaporation with *n*-pentane (1 × 4 mL, 3 × 2 mL). The orange solid crude product was washed with *n*-pentane (3 × 2 mL) and dried under reduced pressure. The product was obtained as orange solid (113 mg, 61 µmol, 81 %). Orange single crystals suitable for X-Ray diffraction could be obtained from a solution of **4** in a mixture of *n*-pentane (1 ml) and *o*-difluorobenzene (0.1 ml) at room temperature. **^{1}H-NMR** (400.11 MHz, 299.2 K, C_6D_6): δ (ppm) = 8.42 – 8.34 (m, 8 H, *o*-BAr^F$_4$), 7.67 (br s, 4 H, *p*-BAr^F$_4$), 7.40 – 7.29 (m, 3 H, *m*-C_6H_3, *p*-C_6H_3), 7.27 – 7.22 (m, 1 H, H-6), 7.18 – 6.91 (m, 15 H, 4 × *m*-C_6H_2, H-5, 10 × C_6H_5, overlapping solvent signal), 6.89 – 6.82 (m, 1 H, H-3), 6.75 – 6.69 (m, 1 H, H-4), 2.86 (sept, 4 H, $^3J_{H\text{-}H}$ = 6.8 Hz, *o*-C$\underline{H}$(CH_3)$_2$)), 2.66 (sept, 2 H, $^3J_{H\text{-}H}$ = 6.9 Hz, *p*-C$\underline{H}$(CH_3)$_2$), 1.76 – 1.63 (m, 3 H, Ad-CH), 1.63 – 1.54 (m, 6 H, N-Ad-CH_2), 1.37 – 0.88 (m, 42 H,

6 × Ad-CH_2, 24 × o-$CH(C\underline{H}_3)_2$, 12 × p-$CH(C\underline{H}_3)_2$, partly overlapped by *n*-pentane and unidentified impurity). **$^{13}C\{^1H\}$-NMR** (100.62 MHz, 299.2 K, C_6D_6): δ (ppm) = 162.4 (q, $^1J_{13C\text{-}11B}$ = 50.0 Hz, *i*-BAr^F_4), 150.5 (s, *p*-C_6H_2), 147.2 (br, C-1, only found in HMBC experiment), 146.9 (s, *o*-C_6H_2), 146.2 (s, *o*-C_6H_3), 141.6 (br, *i*-C_6H_3), 135.6 (s, *i*-C_6H_2), 135.1 (br, *o*-BAr^F_4), 133.4 (d, $^3J_{31P\text{-}13C}$ = 14.1 Hz, C-6), 133.1 (d, $^4J_{31P\text{-}13C}$ = 2.2 Hz, C-5), 133.0 (d, $^4J_{31P\text{-}13C}$ = 2.2 Hz, *p*-C_6H_5), 132.6 (d, $^1J_{31P\text{-}13C}$ = ca. 90 Hz, C-2), 131.5 (d, $^2J_{31P\text{-}13C}$ = 11.1 Hz, *o*-C_6H_5), 130.7 (d, $^2J_{31P\text{-}13C}$ = 8.8 Hz, C-3), 130.5 (s, *p*-C_6H_3), 130.3 (d, $^3J_{31P\text{-}13C}$ = 9.7 Hz, C-4), 129.7 (s, *m*-C_6H_3), 129.5 (qq, $^2J_{19F\text{-}13C}$ = ca. 31.6 Hz, $^3J_{13C\text{-}11B}$ = ca. 2.9 Hz, *m*-BAr^F_4), 129.4 (d, $^3J_{31P\text{-}13C}$ = 12.0 Hz, *m*-C_6H_2), 124.9 (q, $^1J_{19F\text{-}13C}$ = 273.6 Hz, CF_3), 124.8 (d, $^1J_{31P\text{-}13C}$ = 71.3 Hz, *i*-C_6H_5), 121.0 (s, *m*-C_6H_2), 117.7 (sept., $^4J_{19F\text{-}13C}$ = ca. 3.8 Hz, *p*-BAr^F_4), 61.4 (s, Ad-NC_q), 42.4 (s, Ad-N-$C\underline{C}H_2$), 34.3 (s, *p*-$\underline{C}H(CH_3)_2$), 34.3 (s, Ad-$CH\underline{C}H_2$), 30.7 (s, o-$\underline{C}H(CH_3)_2$), 28.5 (s, Ad-$\underline{C}HCH_2$), 26.0 (br s, *o*-$CH(\underline{C}H_3)_2$), 23.6 (s, *p*-$CH(\underline{C}H_3)_2$), 22.5 (*o*-$CH(\underline{C}H_3)_2$). **$^{11}B\{^1H\}$-NMR** (128.37 MHz, 299.2 K, toluene-d_8): δ (ppm) = –6.0 ppm (s, [$\underline{B}Ar^F_4$]), –13.4 (d, $^1J_{31P\text{-}11B}$ = 149.0 Hz, Ge=$\underline{B}$). **$^{31}P\{^1H\}$-NMR** (161.97 MHz, 299.2 K, toluene-d_8): δ (ppm) = 28.7 (q, $^1J_{31P\text{-}11B}$ = ca. 152.4 Hz, Ge=B-$\underline{P}$). **$^{19}F\{^1H\}$-NMR** (376.48 MHz, 299.2 K, toluene-d_8): δ (ppm) = – 62.2 (s, CF_3). **Elemental analysis:** Calculated (%) for $C_{97}H_{90}B_2F_{24}GeNP$: 62.94 C, 4.90 H, 0.76 N. Found: 63.52 C, 4.66 H, 0.81 N. **IR (KBr):** 2160 cm^{-1} (ν C=N).

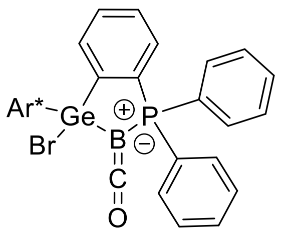

5: **1b** (74.1 mg, 81.8 µmol, 1 eq.) was dissolved in benzene (2 mL) and transferred in a Schlenk tube. By using the „freeze-pump-thaw“-technique, Argon was removed from the atmosphere and replaced by carbon monoxide (1 atm.). The solution, initially intense red, turned orange during the reaction. After stirring for 19h at room temperature, all volatile components were removed under reduced pressure, yielding an orange powder of **5** (74.1 mg, 79.3 µmol, 97 %). Single crystals suitable for X-Ray crystallography could be obtained by slow evaporation of a concentrated solution of **5** in *n*-pentane at –40 °C. **^1H-NMR** (400.11 MHz, 299.2 K, C_6D_6): δ (ppm) = 7.50 – 7.39 (m, 4 H, *o*-C_6H_5), 7.35 – 7.29 (m, 2 H, *m*-C_6H_2), 7.14 – 6.98 (m, 9 H, 2 × *m*-C_6H_5, 2 × *m*-C_6H_3, 2 × *m*-C_6H_2, H-3, *p*-C_6H_3, *p*-C_6H_5), 6.93 – 6.86 (m, 1 H, H-5), 6.81 – 6.75 (m, 1 H, *p*-C_6H_5), 6.74 – 6.65 (m, 3 H, H-4, *m*-C_6H_5), 6.44 – 6.37 (m, 1 H, H-6), 3.42 – 3.24 (m, 2 H, *o*-$C\underline{H}(CH_3)_2$), 3.24 – 3.06 (m, 2 H, *o*-$C\underline{H}(CH_3)_2$), 2.94 (sept, 2 H, $^3J_{H\text{-}H}$ = 6.9 Hz, *p*-$C\underline{H}(CH_3)_2$), 1.37 (d, 12 H, $^3J_{H\text{-}H}$ = 7.0 Hz, *p*-$CH(C\underline{H}_3)_2$), 1.35 – 1.26 (m, 6 H, *o*-$CH(C\underline{H}_3)_2$), 1.26 – 1.07 (m, 12 H, *o*-$CH(C\underline{H}_3)_2$), 1.02 (d, 6 H, $^3J_{H\text{-}H}$ = 6.7 Hz, *o*-$CH(C\underline{H}_3)_2$). **$^{13}C\{^1H\}$-NMR** (100.61 MHz, 299.2 K, C_6D_6): δ (ppm) = 220.4 (br q, B=$\underline{C}$=O, only detectable when using ^{13}CO as reagent), 152.7 (d,$^2J_{31P\text{-}13C}$ = 26.4 Hz, C-1), 148.8 (s, *p*-C_6H_2), 147.3 (s, *o*-C_6H_2), 146.8 (s, *o*-C_6H_2), 145.3 (br, *o*-C_6H_3), 141.3 (d, $^3J_{31P\text{-}13C}$ = 4.9 Hz, *i*-C_6H_3), 139.0 (s, *i*-C_6H_2), 136.6 (d, $^3J_{31P\text{-}13C}$ = 13.6 Hz, C-6), 132.6 (d, $^2J_{31P\text{-}13C}$ = 11.8 Hz, *o*-C_6H_5), 132.1 (s, *m*-$\underline{C}_6H_3$), 131.9 (d, $^2J_{31P\text{-}13C}$ = 10.4 Hz, *o*-C_6H_5), 131.8 (d, $^1J_{31P\text{-}13C}$ = 75.0 Hz, C-2), 131.2 (d, $^4J_{31P\text{-}13C}$ = 2.2 Hz, *p*-$\underline{C}_6H_5$), 131.0 (d, $^1J_{31P\text{-}13C}$ = 58.9 Hz, *i*-C_6H_5), 130.9 (d, $^4J_{31P\text{-}13C}$ = 2.0 Hz, C-5), 130.6 (d, $^4J_{31P\text{-}13C}$ = 1.9 Hz, *p*-C_6H_5), 130.4 (d, $^1J_{31P\text{-}13C}$ = 57.3 Hz, *i*-C_6H_5), 130.0 (d, J = 7.6 Hz, C-3), 128.7 (d, $^3J_{31P\text{-}13C}$ = 12.1 Hz, *m*-C_6H_5), 128.5 (d, $^3J_{31P\text{-}13C}$ = 11.8 Hz, *m*-C_6H_5), 127.8 (d, $^3J_{31P\text{-}13C}$ = 9.4 Hz, C-4, overlapping solvent signal), 127.6 (s, *p*-C_6H_3, overlapping solvent signal), 121.8 (s, *m*-C_6H_2), 121.6 (s, *m*-C_6H_2), 34.6 (s, *p*-$\underline{C}H(CH_3)_2$), 31.1 (s, *o*-$\underline{C}H(CH_3)_2$), 30.9 (s, *o*-$\underline{C}H(CH_3)_2$), 25.6 (s, *o*-$CH(\underline{C}H_3)_2$), 25.2 (s, *o*-$CH(\underline{C}H_3)_2$), 24.2 (s, *p*-$CH(\underline{C}H_3)_2$), 24.0 (s, *p*-$CH(\underline{C}H_3)_2$), 23.6 – 23.1 (m, *o*-$CH(\underline{C}H_3)_2$). **$^{11}B\{^1H\}$-NMR** (128.37 MHz, 299.2 K, C_6D_6): δ (ppm) = –39.7 (d, $^1J_{31P\text{-}11B}$ = 121.3 Hz). **$^{31}P\{^1H\}$-NMR** (161.97 MHz, 299.2 K, C_6D_6): δ (ppm) = 40.1 (broad unresolved q). **Elemental analysis:** Calculated (%) for $C_{55}H_{63}BBrGePO$: 70.70 C, 6.80 H. Found: 71.21 C, 6.83 H. **IR (KBr):** 1984 cm^{-1} (ν CO).

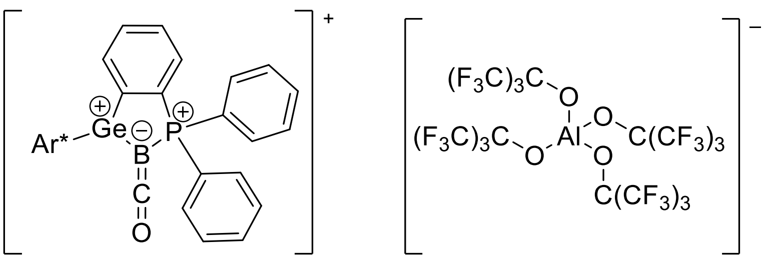

6: **5** (70.9 mg, 75.9 µmol, 1 eq.) was dissolved in benzene (5 mL). To this solution, a suspension of Ag[Al(OtBu^F)$_4$] (81.6 mg, 75.9 µmol, 1 eq.) in *o*-difluorobenzene (1.5 mL) was added. The orange reaction mixture turns considerably darker over the course of the reaction. After stirring for ten minutes at room temperature, the reaction mixture was filtered. The solvent was removed from the filtrate under reduced pressure and residual solvent was removed from the obtained dark orange oil by co-evaporation with *n*-pentane (4 mL). The solid orange crude product was dissolved in *o*-difluorobenzene (1.5 mL) and concentrated to ca. 0.3 ml and cooled to –40 °C. Cold *n*-pentane (3 mL, –40 °C) was layered on top of the cold solution and the mixture was stored at –40 °C for crystallization. After four days, the mother liquor was removed from the orange crystals and the crystals were washed with *n*-pentane (2 × 1 mL). A second crystallization was performed by dissolving the crystalline solid in *o*-difluorobenzene (0.4 mL), concentrating the solution almost to dryness, and layering *n*-pentane (3.4 mL) on top of the solution and storing the mixture at –40 °C for one day. The mother liquor was removed and the crystals were washed with *o*-difluorobenzene (0.5 mL) and *n*-pentane (2 × 2 mL) and dried under reduced pressure. Residual solvent was removed by co-evaporation with *n*-pentane (1 mL). The product [GeBCO][Al(OtBu^F)$_4$] (48.8 mg, 26.8 µmol, 35.3 %) was obtained as an orange crystalline solid. The mother liquor from the second crystallization was combined with the washing solution and stored at –40 °C for two months. From this solution, further product crystallized and after removing the mother liquor and drying under reduced pressure **6** was isolated as an orange crystalline solid (combined yield: 96.1 mg, 52.8 µmol, 69.5 %). Orange single crystals from the first crystallization were suitable for X-Ray diffraction. **^{1}H-NMR** (400.11 MHz, 299.2 K, C_6D_6 + *o*-$C_6H_4F_2$): δ (ppm) = 7.46 – 7.37 (m, 3 H, *m*-C_6H_3, *p*-C_6H_3), 7.26 – 6.89 (m, 14 H, PCH_{ar}, overlapping solvent signal), 6.88 (s, 4 H, *m*-C_6H_2), 2.81 (sept, $^3J_{H\text{-}H}$ = 6.8 Hz, 4 H, *o*-C$\underline{H}$(CH$_3$)$_2$), 2.51 (sept, $^3J_{H\text{-}H}$ = 6.9 Hz, 2 H, *p*-C$\underline{H}$(CH$_3$)$_2$), 1.00 (d, $^3J_{H\text{-}H}$ = 6.7 Hz, 12 H, *o*-CH(C$\underline{H}_3$)$_2$), 0.96 (d, $^3J_{H\text{-}H}$ = 6.8 Hz, 12 H, *o*-CH(C$\underline{H}_3$)$_2$), 0.89 (d, $^3J_{H\text{-}H}$ = 7.0 Hz, 12 H, *p*-CH(C$\underline{H}_3$)$_2$). **^{13}C{^{1}H}-NMR** (100.61 MHz, 299.2 K, C_6D_6 + *o*-$C_6H_4F_2$): δ (ppm) = 203.4 (q, $^1J_{13C\text{-}11B}$ = 111.6 Hz, B$\underline{C}$O, only detectable when using ^{13}C-labelled CO), 151.7 (s, *p*-C_6H_2), 147.0 (s, *o*-C_6H_2), 145.5 (s, *o*-C_6H_3), 144.3 (br, $PC_{ar,q}$), 142.7 (d, $^3J_{31P\text{-}13C}$ = 8.7 Hz, *i*-C_6H_3), 135.2 (s, *i*-C_6H_2), 133.8 (d, $J_{31P\text{-}13C}$ = 2.3 Hz, PC_{ar}), 133.5 (d, $J_{31P\text{-}13C}$ = 13.2 Hz, PC_{ar}), 133.3 (d, $J_{31P\text{-}13C}$ = 2.0 Hz, PC_{ar}), 132.9 (d, $J_{31P\text{-}13C}$ = 10.1 Hz, PC_{ar}), 131.9 (s, *p*-C_6H_3), 131.8 – 131.5 (m, PC_{ar}), 129.8 (d, $J_{31P\text{-}13C}$ = 12.7 Hz, PC_{ar}), 129.7 (s, *m*-C_6H_3), 122.9 (d, $J_{31P\text{-}13C}$ = 75.8 Hz, $PC_{ar,q}$), 122.1 (s, m-C_6H_2), 121.9 (q, $^1J_{19F\text{-}13C}$ = 292.8 Hz, CF_3), 79.6 (br, $\underline{C}$(CF$_3$)$_3$), 34.2 (s, *p*-$\underline{C}$H(CH$_3$)$_2$), 30.8 (s, *o*-$\underline{C}$H(CH$_3$)$_2$), 26.2 (s, *o*-CH($\underline{C}$H$_3$)$_2$), 23.2 (s, *p*-CH($\underline{C}$H$_3$)$_2$), 21.2 (*o*-CH($\underline{C}$H$_3$)$_2$). **^{11}B{^{1}H}-NMR** (128.37 MHz, 299.2 K, C_6D_6 + *o*-$C_6H_4F_2$): δ (ppm) = –24.8 (d, $^1J_{31P\text{-}11B}$ = 168.2 Hz); ^{13}CO labelled sample: –24.8 (dd, $^1J_{31P\text{-}11B}$ = 160.1 Hz, $^1J_{13C\text{-}11B}$ = 119.3 Hz);. **^{19}F{^{1}H}-NMR** (376.48 MHz, 299.2 K, C_6D_6 + *o*-$C_6H_4F_2$): δ (ppm) = –74.9 (s, C$\underline{F}_3$). **^{27}Al{^{1}H]-NMR** (104.26 MHz, 299.2 K, C_6D_6 + *o*-$C_6H_4F_2$) = 35.3 (s). **^{31}P{^{1}H}-NMR** (161.97 MHz, 299.2 K, C_6D_6 + *o*-$C_6H_4F_2$): δ (ppm) = 32.3 (q, $^1J_{31P\text{-}11B}$ = 166.1 Hz). **Elemental analysis:** Calculated (%) for $C_{71}H_{90}AlBBrGeN_2P \cdot C_7H_8$: 46.82 C, 3.49 H. Found: 47.10 C, 3.755 H. **IR (KBr):** 2024 cm^{-1} (ν CO).

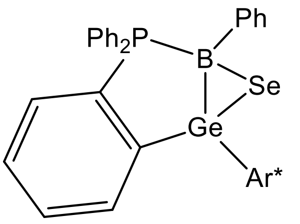

7: **1c** (70.7 mg, 78.2 µmol, 1 eq.) and selenium (6.5 mg, 82.3 µmol, 1.05 eq.) were combined. The mixture was suspended in benzene (2 mL) and stirred for 21 h at room temperature. After filtration of the dark suspension, the solvent was removed from the pale-yellow filtrate by evaporation under reduced pressure and residual solvent was removed by co-evaporation with *n*-pentane (4 mL). After crystallization from toluene at room temperature and washing the obtained crystals with *n*-pentane (0.5 mL), a pale yellow crystalline solid of **7** (29.6 mg, 30.1 µmol, 39%) was obtained. The obtained crystals were suitable for X-Ray diffraction. **^{1}H-NMR** (600.13 MHz, 253.0 K, toluene-d$_8$): δ (ppm) = 7.74 – 7.64 (m, 1 H, H-6), 7.47 – 7.32 (m, 6 H, 2 × *m*-C$_6$H$_3$, 2 × *o*-PC$_6$H$_5$, 2 × *o*-BC$_6$H$_5$), 7.31 – 7.25 (m, 2 H, *m*-C$_6$H$_2$, *p*-C$_6$H$_3$), 7.18 – 7.05 (m, 7 H, 3 × *m*-C$_6$H$_2$, *p*-PC$_6$H$_5$, 2 × *m*-BC$_6$H$_5$, *p*-BC$_6$H$_5$, partly overlapping solvent signal), 7.05 – 6.98 (m, 5 H, H-5, 2× *m*-PC$_6$H$_5$, 2 × *o*-PC$_6$H$_5$, partly overlapping solvent signal), 6.94 – 6.90 (m, 1 H, H-3), 6.90 – 6.86 (m, 1 H, *p*-PC$_6$H$_5$), 6.82 – 6.77 (m, 1 H, H-4), 6.75 – 6.66 (m, 2 H, *m*-PC$_6$H$_5$), 3.49 (sept, $^3J_{H-H}$ = 6.6 Hz, 1 H, *o*-C$\underline{H}$(CH$_3$)$_2$), 3.27 (sept, $^3J_{H-H}$ = 6.7 Hz, 1H, *o*-C$\underline{H}$(CH$_3$)$_2$), 3.00 – 2.86 (m, 2 H, *p*-C$\underline{H}$(CH$_3$)$_2$), 2.83 (sept, $^3J_{H-H}$ = 6.8 Hz, 1 H, *o*-C$\underline{H}$(CH$_3$)$_2$), 2.58 (sept, $^3J_{H-H}$ = 6.8 Hz, 1 H, *o*-C$\underline{H}$(CH$_3$)$_2$), 1.57 (d, $^3J_{H-H}$ = 6.6 Hz, 3 H, *o*-CH(C$\underline{H}_3$)$_2$), 1.43 (d, $^3J_{H-H}$ = 6.6 Hz, 3 H, *o*-CH(C$\underline{H}_3$)$_2$), 1.40 – 1.32 (m, 15 H, 3 × *o*-CH(C$\underline{H}_3$)$_2$, 12 × *p*-CH(C$\underline{H}_3$)$_2$), 1.17 (d, $^3J_{H-H}$ = 6.6 Hz, 3 H, *o*-CH(C$\underline{H}_3$)$_2$), 1.10 (d, $^3J_{H-H}$ = 6.7 Hz, 3 H, *o*-CH(C$\underline{H}_3$)$_2$), 0.96 (d, $^3J_{H-H}$ = 6.7 Hz, 3 H, *o*-CH(C$\underline{H}_3$)$_2$), 0.58 (d, $^3J_{H-H}$ = 6.6 Hz, 3 H, *o*-CH(C$\underline{H}_3$)$_2$), 0.44 (d, $^3J_{H-H}$ = 6.8 Hz, 3 H, *o*-CH(C$\underline{H}_3$)$_2$). **^{13}C{^{1}H}-NMR** (150.90 MHz, 253.0 K, toluene-d$_8$): δ (ppm) = 156.7 (d, $^2J_{31P-13C}$ = 20.0 Hz, C-1), 148.7 (s, *o*-C$_6$H$_3$), 147.8 (s, *o*-C$_6$H$_2$), 147.1 (s, *p*-C$_6$H$_2$), 147.0 (s, *p*-C$_6$H$_2$), 146.9 (s, *o*-C$_6$H$_2$), 146.2 (s, *o*-C$_6$H$_3$), 144.5 (s, *o*-C$_6$H$_2$), 144.4 (s, *o*-C$_6$H$_2$), 138.6 (s, *i*-C$_6$H$_2$), 137.7 (br, *i*-BC$_6$H$_5$) 137.3 (s, *i*-C$_6$H$_2$), 136.8 (d, $^3J_{31P-13C}$ = 4.6 Hz, *o*-BC$_6$H$_5$), 135.5 (d, $^3J_{31P-13C}$ = 5.9 Hz, *i*-C$_6$H$_3$), 133.5 (d, $^3J_{31P-13C}$ = 12.5 Hz, C-6), 133.1 (d, $^3J_{31P-13C}$ = 4.6 Hz, C-4), 132.7 (d, $^2J_{31P-13C}$ = 7.4 Hz, *o*-PC$_6$H$_5$), 132.2 (d, $^2J_{31P-13C}$ = 9.2 Hz, *o*-PC$_6$H$_5$), 131.6 (d, $^1J_{31P-13C}$ = 82.5 Hz, C-2), 129.9 (s, *p*-PC$_6$H$_5$), 129.7 (s, *m*-C$_6$H$_3$), 129.5 (s, *p*-PC$_6$H$_5$), 128.8 (s, C-5), 128.7 (d, $^1J_{31P-13C}$ = 45.9 Hz, *i*-PC$_6$H$_5$), 128.1 (s, *m*-C$_6$H$_3$), 127.7 (s, *p*-C$_6$H$_3$, overlapping solvent signal, found and identified by HMBC and ^{13}C-DEPT experiments), 127.4 (d, $^3J_{31P-13C}$ = 9.4 Hz, *m*-PC$_6$H$_5$), 127.3 – 127.1 (m, *m*-PC$_6$H$_5$), 126.8 (d, $^2J_{31P-13C}$ = 8.7 Hz, C-3, partly overlapping solvent signal), 126.0 (s, *m*-BC$_6$H$_5$), 125.0 (s, *p*-BC$_6$H$_5$), 123.7 (d, $^1J_{31P-13C}$ = 67.9 Hz, *i*-PC$_6$H$_5$, partly overlapping solvent signal), 120.4 (s, *m*-C$_6$H$_2$), 120.3 (s, *m*-C$_6$H$_2$), 119.8 (s, *m*-C$_6$H$_2$), 119.4 (s, *m*-C$_6$H$_2$), 34.3 (s, *p*-$\underline{C}$H(CH$_3$)$_2$), 34.0 (s, *p*-$\underline{C}$H(CH$_3$)$_2$), 30.5 (s, *o*-$\underline{C}$H(CH$_3$)$_2$), 30.5 (s, *o*-$\underline{C}$H(CH$_3$)$_2$), 30.0 (s, *o*-$\underline{C}$H(CH$_3$)$_2$), 29.8 (*o*-$\underline{C}$H(CH$_3$)$_2$), 26.6 (s, *o*-CH($\underline{C}$H$_3$)$_2$), 25.6 (s, *o*-CH($\underline{C}$H$_3$)$_2$), 25.1 (s, *p*-CH($\underline{C}$H$_3$)$_2$), 25.0 (s, *o*-CH($\underline{C}$H$_3$)$_2$), 24.3 (s, *o*-CH($\underline{C}$H$_3$)$_2$), 24.1 (s, *o*-CH($\underline{C}$H$_3$)$_2$), 23.7 (s, *p*-CH($\underline{C}$H$_3$)$_2$), 23.4 (s, *p*-CH($\underline{C}$H$_3$)$_2$), 22.0 (s, *p*-CH($\underline{C}$H$_3$)$_2$), 21.9 (s, *o*-CH($\underline{C}$H$_3$)$_2$), 21.8 (s, *o*-CH($\underline{C}$H$_3$)$_2$), 19.9 (s, *o*-CH($\underline{C}$H$_3$)$_2$). **^{11}B{^{1}H}-NMR** (192.55 MHz, 253.0 K, toluene-d$_8$): δ (ppm) = –18.1 (br). **^{31}P{^{1}H}-NMR** (242.94 MHz, 253.0 K, toluene-d$_8$): δ (ppm) = 6.5 (s).**^{77}Se{^{1}H}-NMR** (57.23 MHz, 298.0 K, C$_6$D$_6$): δ (ppm) = –386.7 (br). **Elemental analysis:** Calculated (%) for C$_{60}$H$_{68}$BGePSe: 73.35 C, 6.98 H. Found: 73.94 C, 7.35 H.

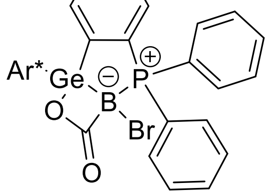

8: **1b** (37.5 mg, 41.4 µmol, 1 eq.) was dissolved in C$_6$D$_6$ (0.6 mL) and transferred to a J. Young NMR tube. Using the "freeze-pump-thaw"-technique, the Argon atmosphere was removed and then replaced by CO$_2$. (1 atm.) Full conversion was confirmed by NMR spectroscopy after 1.5 h. During this time, the color of the solution changed from intense red to colorless. The solution was transferred to a vial and frozen by cooling to –40 °C. The solvent was removed by sublimation under reduced pressure and a colorless powder of **8** (39.0 mg, 41.0 µmol, 99 %) was obtained. Single crystals suitable for X-Ray diffraction were obtained from a concentrated solution of **8** in benzene at room temperature. **^{1}H-NMR** (400.11 MHz, 300.2 K, C$_6$D$_6$): δ (ppm) = 7.76 – 7.68 (m, 1 H, H-6), 7.38 – 7.30 (m, 4 H, 2 × *m*-C$_6$H$_3$, 2 × *o*-C$_6$H$_5$), 7.26 – 7.21 (m, 3 H, *m*-C$_6$H$_2$, *p*-C$_6$H$_3$), 7.14 – 7.12 (m, 2 H, *m*-C$_6$H$_2$, partly overlapped by solvent signal), 7.09 – 7.04 (m, 1 H, H-5), 7.04 – 6.92 (m, 6 H, H-3, *p*-C$_6$H$_5$, 2 × *m*-C$_6$H$_5$, 2 × *o*-C$_6$H$_5$), 6.88 – 6.82 (m, 1 H, *p*-C$_6$H$_5$), 6.82 – 6.75 (m, 1 H, H-4), 6.72 – 6.64 (m, 2 H, *m*-C$_6$H$_5$), 3.25 – 3.08 (m, 4 H, *o*-C$\underline{H}$(CH$_3$)$_2$), 2.78 (sept, 2 H,

$^{3}J_{H\text{-}H}$ = 6.9 Hz, *p*-C$\underline{H}$(CH$_3$)$_2$), 1.47 (d, 6 H, $^{3}J_{H\text{-}H}$ = 6.7 Hz, *o*-CH(C$\underline{H}_3$)$_2$), 1.26 – 1.21 (m, 12 H, *o*-CH(C$\underline{H}_3$)$_2$, *p*-CH(C$\underline{H}_3$)$_2$), 1.19 (d, 6 H, $^{3}J_{H\text{-}H}$ = 6.9 Hz, *p*-CH(C$\underline{H}_3$)$_2$), 1.10 (d, 6 H, $^{3}J_{H\text{-}H}$ = 6.7 Hz, *o*-CH(C$\underline{H}_3$)$_2$), 0.97 (d, 6 H, $^{3}J_{H\text{-}H}$ = 6.8 Hz, *o*-CH(C$\underline{H}_3$)$_2$). **^{13}C{^{1}H}-NMR** (100.62 MHz, 299.2 K, C_6D_6): δ (ppm) = 184.2 (br, O-C=O, only detectable when using $^{13}CO_2$ as reagent), 153.6 (d, $^{2}J_{31P\text{-}13C}$ = 25.5 Hz, C-1), 148.4 (s, *p*-C_6H_2), 147.4 (s, *o*-C_6H_3), 146.7 (s, *o*-C_6H_2), 146.5 (s, *o*-C_6H_2), 137.1 (d, $^{3}J_{31P\text{-}13C}$ = 8.2 Hz, *i*-C_6H_3), 136.6 (s, *i*-C_6H_2), 134.3 (d, $^{3}J_{31P\text{-}13C}$ = 14.3 Hz, C-6), 133.8 (d, $^{3}J_{31P\text{-}13C}$ = 9.3 Hz, *m*-C_6H_5), 133.7 (d, $^{2}J_{31P\text{-}13C}$ = 6.7 Hz, C-3), 133.4 (d, $^{2}J_{31P\text{-}13C}$ = 9.7 Hz, *o*-C_6H_5), 133.1 (d, $^{1}J_{31P\text{-}13C}$ = 71.9 Hz, C-2), 131.9 (d, $^{4}J_{31P\text{-}13C}$ = 2.3 Hz, *p*-C_6H_5), 131.5 – 131.2 (m, C-5, *p*-C_6H_5), 130.5 (s, *m*-C_6H_3), 129.8 (d, $^{3}J_{31P\text{-}13C}$ = 4.4 Hz, C-4), 128.9 (d, $^{3}J_{31P\text{-}13C}$ = 11.5 Hz, *m*-C_6H_5), 128.8 (s, *p*-C_6H_3), 128.3 (d, $^{2}J_{31P\text{-}13C}$ = 11.5 Hz, *o*-C_6H_5), 126.7 (d, $^{1}J_{31P\text{-}13C}$ = 66.8 Hz, *i*-C_6H_5), 121.4 (s, *m*-C_6H_2), 121.1 (d, $^{1}J_{31P\text{-}13C}$ = 63.8 Hz, *i*-C_6H_5), 120.9 (s, *m*-C_6H_2), 34.5 (s, *p*-$\underline{C}$H(CH$_3$)$_2$), 31.3 (s, *o*-$\underline{C}$H(CH$_3$)$_2$), 31.1 (s, *o*-$\underline{C}$H(CH$_3$)$_2$), 26.2 (s, *p*-CH($\underline{C}$H$_3$)$_2$), 26.0 (s, *o*-CH($\underline{C}$H$_3$)$_2$), 24.5 (s, *o*-CH($\underline{C}$H$_3$)$_2$), 24.0 (s, *o*-CH($\underline{C}$H$_3$)$_2$), 23.3 (s, *p*-CH($\underline{C}$H$_3$)$_2$), 21.9 (s, *o*-CH($\underline{C}$H$_3$)$_2$). **^{11}B{^{1}H}-NMR** (128.37 MHz, 299.2 K, C_6D_6): δ (ppm) = –12.9 (br). **^{31}P{^{1}H}-NMR** (161.97 MHz, 299.2 K, C_6D_6): δ (ppm) = 14.5 (br). **Elemental analysis:** Calculated (%) for $C_{55}H_{63}BBrGePO_2$: 71.24 C, 6.76 H. Found: 71.23 C, 6.90 H. **IR (KBr):** 1700 cm^{-1} (ν C=O).

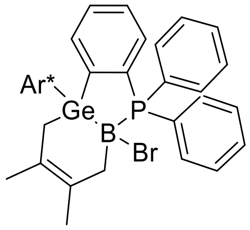

9: 2,3-Dimethylbuta-1,3-diene (0.02 mL, 178 µmol, 2.87 eq.) was added to a solution of **1b** (56.2 mg, 62 µmol, 1 eq.) in benzene (2 mL). The reaction mixture was stirred at room temperature for 27.5 h. During the reaction, the intense red solution decolorizes. The solvent was removed under reduced pressure. The crude product was subsequently purified by precipitation from a cooled solution in *n*-pentane (2 mL, –40 °C). The mother liquor was removed by using a syringe and the colorless precipitate was dried under reduced pressure. A colorless solid of **9** was obtained. (38.9 mg, 39.4 µmol, 64 %). Slow evaporation of a solution of **9** in *n*-pentane afforded at room temperature single crystals suitable for X-Ray diffraction. **^{1}H-NMR** (400.11 MHz, 299.2 K, C_6D_6): 7.46 – 7.33 (m, 3 H, H-6, *o*-C$_6\underline{H}_5$), 7.27 – 7.15 (m, 8 H, *m*-C$_6\underline{H}_2$, *m*-C$_6\underline{H}_3$, *o*-C$_6\underline{H}_5$), 7.13 – 7.08 (m, 1 H, *p*-C$_6\underline{H}_3$), 7.08 – 6.99 (m, 4 H, H-5, *p*-C$_6\underline{H}_5$, *m*-C$_6\underline{H}_5$), 6.99 – 6.94 (m, 1 H, H-3), 6.92 – 6.77 (m, 4 H, H-4, *p*-C$_6\underline{H}_5$, *m*-C$_6\underline{H}_5$), 3.35 (sept, 2 H, $^{3}J_{H\text{-}H}$ = 6.8 Hz, *o*-C$\underline{H}$(CH$_3$)$_2$), 3.11 (sept, 2 H, $^{3}J_{H\text{-}H}$ = 6.8 Hz, *o*-C$\underline{H}$(CH$_3$)$_2$), 2.90 (sept, 2 H, $^{3}J_{H\text{-}H}$ = 6.9 Hz, *p*-C$\underline{H}$(CH$_3$)$_2$), 2.76 – 2.53 (m, 1 H, BC$\underline{H}_2$), 2.17 – 1.98 (m, 1 H, BC$\underline{H}_2$), 1.38 (d, 6 H, $^{3}J_{H\text{-}H}$ = 6.9 Hz, *o*-CH(C$\underline{H}_3$)$_2$), 1.35 – 1.23 (m, 13 H, *p*-CH(C$\underline{H}_3$)$_2$), GeC$\underline{H}_2$), 1.22 – 1.12 (m, 12 H, *o*-CH(C$\underline{H}_3$)$_2$); 1.12 – 0.90 (m, 10 H, *o*-CH(C$\underline{H}_3$)$_2$, GeC$\underline{H}_2$, GeCH$_2$-C$\underline{H}_3$), 0.86 (s, 3 H, BCH$_2$C$\underline{H}_3$). **^{13}C{^{1}H}-NMR** (100.61 MHz, 299.2 K, C_6D_6): δ (ppm) = 159.9 (d, $^{2}J_{31P\text{-}13C}$ = 32.4 Hz, C-1), 148.3 (s, *o*-$\underline{C}_6$H$_3$), 147.8 (s, *p*-$\underline{C}_6$H$_2$), 146.7 (s, *o*-$\underline{C}_6$H$_2$), 146.2 (s, *o*-$\underline{C}_6$H$_2$), 141.3 (d, $^{3}J_{31P\text{-}13C}$ = 13.5 Hz, *i*-$\underline{C}_6$H$_3$), 140.9 (s, *i*-$\underline{C}_6$H$_2$), 135.3 (d, $^{3}J_{31P\text{-}13C}$ = 14.6 Hz, C-6), 134.9 (d, $^{2}J_{31P\text{-}13C}$ = 8.0 Hz, *o*-$\underline{C}_6$H$_5$), 133.4 (d, $^{3}J_{31P\text{-}13C}$ = 8.5 Hz, *m*-C_6H_5), 132.9 (d, $^{2}J_{31P\text{-}13C}$ = 5.3 Hz, C-3), 132.3 (d, $^{1}J_{31P\text{-}13C}$ = 70.9 Hz, C-1), 131.8 (s, *m*-C_6H_3), 130.5 (d, $^{4}J_{31P\text{-}13C}$ = 2.4 Hz, C-5), 130.4 (br s, *p*-C_6H_5), 129.6 (d, $^{1}J_{P\text{-}C}$ = 56.4 Hz, *i*-C_6H_5), 128.5 (s, C=C), 127.7 (d, $^{2}J_{31P\text{-}13C}$ = 9.8 Hz, *o*-C_6H_5, overlapping solvent signal), 127.6 (d, $^{3}J_{31P\text{-}13C}$ = 10.4 Hz, *m*-C_6H_5, overlapping solvent signal), 127.4 (d, $^{3}J_{31P\text{-}13C}$ = 7.9 Hz, C-4, overlapping solvent signal), 126.4 (s, *p*-C_6H_3), 126.3 (d, $^{1}J_{31P\text{-}13C}$ = 50.9 Hz, *i*-C_6H_5), 126.0 (s, C=C), 121.4 (s, *m*-C_6H_2), 121.4 (s, *m*-C_6H_2), 34.6 (s, *p*-$\underline{C}$H(CH$_3$)$_2$), 34.4 (br, BCH$_2$), 31.1 (s, o-$\underline{C}$H(CH$_3$)$_2$), 31.0 (s, *o*-$\underline{C}$H(CH$_3$)$_2$), 27.1 (Ge-CH$_2$), 26.0 (s, *o*-CH($\underline{C}$H$_3$)$_2$), 25.7 (s, *o*-CH($\underline{C}$H$_3$)$_2$), 24.5 (*p*-CH($\underline{C}$H$_3$)$_2$), 23.9 (s, *p*-CH($\underline{C}$H$_3$)$_2$), 23.5 (s, *o*-CH($\underline{C}$H$_3$)$_2$), 22.9 (s, GeCH$_2\underline{C}$H$_3$), 22.7 (s, o-CH($\underline{C}$H$_3$)$_2$), 22.4 (BCH$_2\underline{C}$H$_3$). **^{11}B{^{1}H}-NMR** (128.37 MHz, 299.2 K, C_6D_6): δ (ppm) = –9.7 (br). **^{31}P{^{1}H}-NMR** (161.97 MHz, 299.2 K, C_6D_6) δ (ppm) = 14.6 (s). **Elemental analysis:** Calculated (%) for $C_{60}H_{73}BBrGeP$: 72.90 C, 7.44 H. Found: 73.18 C, 7.55 H.

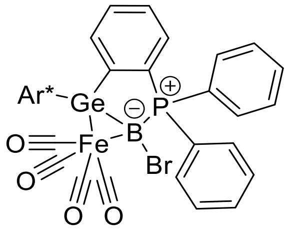

10: **1b** (92 mg, 102 µmol, 1 eq.) was dissolved in toluene (2 mL) and added to $Fe_2(CO)_9$ (36.9 mg, 102 µmol, 1 eq.). The resulting solution was stirred for four hours at room temperature. During the reaction, the color of the solution changed from intense red to red-brown. The crude product was obtained in the form of yellow crystals after filtration and subsequent concentration of the filtrate almost to dryness. The mother liquor was removed, and the crystals were washed with *n*-pentane (3 × 1 mL). To remove residual amounts of solvent, the crystals were dissolved in benzene (4.5 mL), cooled to –40 °C and the frozen solvent was removed by sublimation under reduced pressure. A yellow powder of **10** (81.8 mg, 76.1 µmol, 75 %) was obtained. Single crystals suitable for X-Ray diffraction were obtained from a concentrated solution of **10** in toluene at room temperature. **^{1}H-NMR** (400.11 MHz, 300.2 K, C_6D_6): δ (ppm) = 7.72 – 7.63 (m, 2 H, *o*-C_6H_5), 7.58 – 7.52 (m, 1 H, *m*-C_6H_2), 7.52 – 7.46 (m, 1 H, H-6), 7.32 – 7.27 (m, 2 H, *m*-C_6H_2), 7.26 – 7.22 (m, 2 H, *m*-C_6H_3), 7.17 – 7.13 (m, 1 H, *m*-C_6H_2, overlapping solvent signal), 7.13 – 7.06 (m, 4 H, *p*-C_6H_3, *p*-C_6H_5, *m*-C_6H_5), 7.06 – 6.94 (m, 5 H, *p*-C_6H_5, *o*-C_6H_5, *m*-C_6H_5), 6.93-6.85 (m, 2 H, H-3, H-5), 6.74 – 6.67 (m, 1 H, H-4), 3.67 – 3.52 (m, 2 H, *o*-C$\underline{H}$(CH_3)$_2$), 3.19 (sept, 1 H, $^3J_{H\text{-}H}$ = 6.6 Hz, *o*-C$\underline{H}$(CH_3)$_2$), 3.03 (sept, 1 H, $^3J_{H\text{-}H}$ = 6.9 Hz, *p*-C$\underline{H}$(CH_3)$_2$), 2.91 (sept, 1 H, $^3J_{H\text{-}H}$ = 6.9 Hz, *p*-C$\underline{H}$(CH_3)$_2$), 2.83 (sept, 1 H, $^3J_{H\text{-}H}$ = 6.8 Hz, *o*-C$\underline{H}$(CH_3)$_2$), 1.78 (d, 3 H, $^3J_{H\text{-}H}$ = 6.7 Hz, *o*-CH(C$\underline{H}_3$)$_2$), 1.54 (d, 3 H, $^3J_{H\text{-}H}$ = 6.7 Hz, *o*-CH(C$\underline{H}_3$)$_2$), 1.43 (d, 3 H, $^3J_{H\text{-}H}$ = 6.9 Hz, *p*-CH(C$\underline{H}_3$)$_2$), 1.38 – 1.31 (m, 4 H, 3 × *p*-CH(C$\underline{H}_3$)$_2$, 1 × *o*-CH(C$\underline{H}_3$)$_2$), 1.29 (d, 3 H, $^3J_{H\text{-}H}$ = 6.7 Hz, *o*-CH(C$\underline{H}_3$)$_2$), 1.23 (d, 3 H, $^3J_{H\text{-}H}$ = 6.7 Hz, *o*-CH(C$\underline{H}_3$)$_2$), 1.03 (d, 3 H, $^3J_{H\text{-}H}$ = 6.6 Hz, *o*-CH(C$\underline{H}_3$)$_2$), 0.95 (d, 3 H, $^3J_{H\text{-}H}$ = 6.7 Hz, *o*-CH(C$\underline{H}_3$)$_2$), –0.09 (d, 3 H, $^3J_{H\text{-}H}$ = 6.8 Hz, *o*-CH(C$\underline{H}_3$)$_2$). **^{13}C{^{1}H}-NMR** (100.62 MHz, 299.2 K, C_6D_6): δ (ppm) = 212.5 (d, *J* =4.0 Hz, CO), 210.6 (br q, CO), 209.7 (br s, CO), 208.6 (br s, CO), 156.8 (d, $^3J_{31P\text{-}13C}$ = 24.4 Hz, C-1), 148.8 (s, *o*-C_6H_3), 148.3 (s, *p*-C_6H_2), 147.8 (s, *o*-C_6H_2), 147.6 (s, *p*-C_6H_2), 147.0 (s, *o*-C_6H_3), 146.9 (s, *o*-C_6H_2), 146.1 (s, *o*-C_6H_2), 145.7 (s, *o*-C_6H_2), 140.3 (s, *i*-C_6H_2), 138.6 (s, *i*-C_6H_2), 137.8 (d, $^3J_{31P\text{-}13C}$ = 8.7 Hz, *i*-C_6H_3), 136.5 (d, $^3J_{31P\text{-}13C}$ = 13.5 Hz, C-6), 134.2 (d, $^2J_{31P\text{-}13C}$ = 9.4 Hz, *o*-C_6H_5), 133.8 (s, *m*-C_6H_3), 133.2 – 132.8 (m, C-3, *o*-C_6H_5), 131.7 – 131.4 (m, *m*-C_6H_3, *p*-C_6H_5), 131.2 (d, $^4J_{31P\text{-}13C}$ = 2.1 Hz,C-5), 130.9 (d, $^4J_{31P\text{-}13C}$ = 2.0 Hz, *p*-C_6H_5), 129.2 (d, $^1J_{31P\text{-}13C}$ = 62.0 Hz, C-2), 128.6 (d, $^1J_{31P\text{-}13C}$ = 61.6 Hz, *i*-C_6H_5), 128.4 (d, $^3J_{31P\text{-}13C}$ = 10.9 Hz, *m*-C_6H_5), 127.7 (d, $^3J_{31P\text{-}13C}$ = 8.5 Hz, C-4 overlapping solvent signal), 127.0 (s, *p*-C_6H_3), 125.2 (d, $^1J_{31P\text{-}13C}$ = 67.4 Hz, *i*-C_6H_5), 122.7 (s, *m*-C_6H_2), 121.7 (s, *m*-C_6H_2), 121.4 (s, *m*-C_6H_2), 120.8 (s, *m*-C_6H_2), 35.0 (s, *p*-$\underline{C}$H(CH_3)$_2$), 34.3 (s, *p*-$\underline{C}$H(CH_3)$_2$), 31.4 (s, *o*-$\underline{C}$H(CH_3)$_2$), 31.2 (s, *o*-$\underline{C}$H(CH_3)$_2$), 30.7 (s, *o*-$\underline{C}$H(CH_3)$_2$), 2 × 26.6 (s, *o*-CH($\underline{C}H_3$)$_2$), 25.9 (s, *o*-CH($\underline{C}H_3$)$_2$), 25.7 (s, *p*-CH($\underline{C}H_3$)$_2$), 25.2 (s, *o*-CH($\underline{C}H_3$)$_2$), 24.0 (s, *o*-CH($\underline{C}H_3$)$_2$), 23.9 (s, *p*-CH($\underline{C}H_3$)$_2$), 23.6 (s, *p*-CH($\underline{C}H_3$)$_2$), 23.4 (s, *o*-CH($\underline{C}H_3$)$_2$), 22.9 (s, *o*-CH($\underline{C}H_3$)$_2$), 22.5 (s, *o*-CH($\underline{C}H_3$)$_2$), 22.4 (*p*-CH($\underline{C}H_3$)$_2$). **^{31}P{^{1}H}-NMR** (161.97 MHz, 300.2 K, C_6D_6): δ (ppm) = 18.6 (broad unresolved q). **^{11}B{^{1}H}-NMR** (128.37 MHz, 300.2 K, C_6D_6): δ (ppm) = –13.5 (d, $^1J_{31P\text{-}11B}$ = 86.5 Hz). **Elemental analysis:** Calculated (%) for $C_{58}H_{63}BBrFeO_4GeP{\cdot}C_7H_8$: 66.93 C, 6.14 H. Found: 66.37 C, 6.03 H. **IR (KBr):** 2051 cm^1, 1983 cm^{-1}, 1960 cm^{-1} (ν C=O).

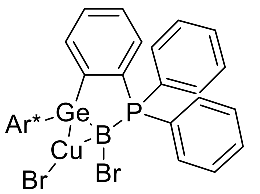

11: **1b** (79.2 mg, 87.4 µmol, 1 eq.) and $CuBr{\cdot}SMe_2$ (18.0 mg, 87.4 µmol, 1 eq.) were combined and stirred thoroughly to ensure good homogeneity. The resulting solid mixture was suspended in benzene (2 mL) and stirred at room temperature. During the reaction, color of the mixture changes from intense red to yellow. After 1 h, the reaction mixture was filtered, and the solvent was removed from the filtrate under reduced pressure. Residual solvent was removed by co-evaporation with *n*-pentane (2 × 2 mL). The crude product was dissolved in *o*-difluorobenzene (1 mL) and crystallized by vapor diffusion of *n*-pentane (3 mL) into the solution at room temperature. After 2 days, the mother liquor was removed, and the resulting orange crystals of **11** were washed with *n*-pentane (2 × 2 mL). After removal of residual solvent under reduced pressure, an orange, crystalline solid of **11** (49.0 mg, 46.7 µmol, 53.4 %) was obtained. Additional product was obtained by crystallization from the mixture of mother liquor and the wash solution (total yield: 71.5 mg, 68.1 µmol, 78 %). The crystals obtained from the first crystallization were suitable for X-Ray

diffraction. **^{1}H-NMR** (400.11 MHz, 300.2 K, C_6D_6): δ (ppm) = 7.81 – 7.71 (m, 2 H, *o*-$PC_6\underline{H}_5$), 7.50 – 7.42 (m, 1 H, H-6), 7.28 – 7.24 (m, 2 H, *m*-$C_6\underline{H}_2$), 7.24 – 7.15 (m, 6 H, *m*-$C_6\underline{H}_2$, *m*-$C_6\underline{H}_3$, *o*-$C_6\underline{H}_5$), 7.14 – 7.09 (m, 1 H, *p*-$C_6\underline{H}_3$), 7.09 – 7.03 (m, 1 H, *p*-$PC_6\underline{H}_5$), 7.03 – 6.95 (m, 6 H, H-5, *p*-$C_6\underline{H}_5$, *m*-$C_6\underline{H}_5$), 6.92 – 6.85 (m, 1 H, H-3), 6.73 – 6.65 (m, 1 H, H-4), 3.19 (sept, 2 H, $^{3}J_{H\text{-}H}$ = 6.8 Hz, *o*-$C\underline{H}(CH_3)_2$), 3.09 (sept, 2 H, $^{3}J_{H\text{-}H}$ = 6.8 Hz, *o*-$C\underline{H}(CH_3)_2$), 3.03 (sept., 2 H, $^{3}J_{H\text{-}H}$ = 6.8 Hz, *p*-$C\underline{H}(CH_3)_2$), 1.44 – 1.31 (m, 18 H, 12 × *p*-$CH(C\underline{H}_3)_2$, 6 × *o*-$CH(C\underline{H}_3)_2$), 1.22 – 1.03 (m, 18 H, *o*-$CH(C\underline{H}_3)_2$). **^{13}C{^{1}H}-NMR** (100.62 MHz, 300.2 K, C_6D_6): δ (ppm) = 149.8 (br, C-1), 149.6 (s, *p*-C_6H_2), 147.4 (s, *o*-C_6H_3), 146.7 (s, *o*-C_6H_2), 145.9 (s, *o*-C_6H_2), 138.3 (d, $^{3}J_{31P\text{-}13C}$ = 18.3 Hz, *i*-C_6H_3), 137.0 (s, *i*-C_6H_2), 134.4 (d, $^{2}J_{31P\text{-}13C}$ = 9.8 Hz, *o*-PC_6H_5), 133.7 (d, $^{3}J_{31P\text{-}13C}$ = 13.0 Hz, C-6), 132.7 – 132.4 (m, *o*-PC_6H_5, C-5), 132.4 (d, $^{1}J_{31P\text{-}13C}$ = 80.9 Hz, C-2), 132.3 (d, $^{4}J_{31P\text{-}13C}$ = 2.5 Hz, *p*-PC_6H_5), 131.4 (d, $^{4}J_{31P\text{-}13C}$ = 2.2 Hz, *p*-PC_6H_5), 131.1 (d, $^{2}J_{31P\text{-}13C}$ = 6.7 Hz, C-3), 130.5 (s, *m*-C_6H_3), 129.2 – 128.9 (m, *m*-C_6H_5, *p*-C_6H_3), 128.7 (d, $^{3}J_{31P\text{-}13C}$ = 11.5 Hz, *m*-C_6H_5), 127.7 (d, $^{2}J_{31P\text{-}13C}$ = 9.2 Hz, C-4, overlapping solvent signal), 127.2 (d, $^{1}J_{31P\text{-}13C}$ = 71.9 Hz, *i*-PC_6H_5, overlapping solvent signal), 122.3 (d, $^{1}J_{31P\text{-}13C}$ = 69.9 Hz, *i*-PC_6H_5), 122.1 (s, *m*-C_6H_2), 122.0 (s, *m*-C_6H_2), 34.2 (s, *p*-$\underline{C}H(CH_3)_2$), 31.1 (s, *o*-$\underline{C}H(CH_3)_2$), 31.0 (s, *o*-$\underline{C}H(CH_3)_2$), 25.9 (s, *o*-$CH(\underline{C}H_3)_2$), 25.7 (s, *o*-$CH(\underline{C}H_3)_2$), 24.3 (s, *o*-$CH(\underline{C}H_3)_2$), 24.1 (s, *p*-$CH(\underline{C}H_3)_2$), 23.8 (*p*-$CH(\underline{C}H_3)_2$), 23.6 (*o*-$CH(\underline{C}H_3)_2$). **^{31}P{^{1}H}-NMR** (161.97 MHz, 300.2 K, C_6D_6): δ (ppm) = 4.2 (broad unresolved q). **^{11}B{^{1}H}-NMR** (128.37 MHz, 300.2 K, C_6D_6): δ (ppm) = 2.5 (d, $^{1}J_{31P\text{-}11B}$ = 120.3 Hz). **Elemental analysis:** Calculated (%) for $C_{54}H_{63}BBr_2CuGeP\cdot C_5H_{12}$: 63.16 C, 6.74 H, found: 62.98 C, 6.45 H.

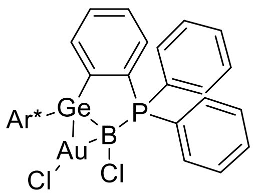

12: **1a** (39.5 mg, 45.8 µmol, 1 eq) was dissolved in a mixture of benzene (1.5 mL) and *n*-pentane (5 mL). A suspension of $AuCl\cdot S(CH_3)_2$ (13.5 mg, 45.8 µmol, 1 eq) in diethyl ether (5 mL) and benzene (1.5 mL) was added dropwise, and the resulting mixture was stirred for two hours at room temperature. During the reaction, the color of the reaction mixture changed from intense red to green and a dark colored solid precipitated. The reaction mixture was filtered and the solvent from the light-yellow filtrate was evaporated under reduced pressure. The light-yellow residue was dissolved in benzene (2 mL) and concentrated to 0.3 mL and cooled down to – 40 °C. Cold *n*-pentane (5 mL, –40 °C) was layered on top of the resulting frozen solution and the mixture was slowly warmed to room temperature. After two days, yellow crystals were obtained. The mother liquor was removed, and the crystals were washed with *n*-pentane (2 mL) and dried under reduced pressure. The product was obtained in the form of yellow crystals (32.8 mg, 30.0 µmol, 65 %). By vapor diffusion of *n*-pentane in a concentrated solution of **12** in THF at –40 °C, single crystals suitable for X-Ray diffraction were obtained. **^{1}H-NMR** (400.11 MHz, 300.2 K, C_6D_6): δ (ppm) = 7.74 – 7.64 (m, 2 H, *o*-C_6H_5), 7.49 – 7.43 (m, 1 H, H-6), 7.26 – 7.21 (m, 2 H, *m*-C_6H_2), 7.18 – 7.10 (m, 7 H, *m*-C_6H_3, *p*-C_6H_3, *m*-C_6H_2, *o*-C_6H_5, overlapping solvent signal), 7.09 – 6.94 (m, 4 H, H-5, *m*-C_6H_5, *p*-C_6H_5), 6.93 – 6.87 (m, 3 H, *m*-C_6H_5, *p*-C_6H_5), 6.84 – 6.78 (m, 1 H, H-3), 6.73 – 6.66 (m, 1 H, H-4), 3.22 (sept., 2 H, $^{3}J_{H\text{-}H}$ = 6.8 Hz, *o*-$C\underline{H}(CH_3)_2$), 3.12 – 2.96 (m, 4 H, *p*-$C\underline{H}(CH_3)_2$, *o*-$C\underline{H}(CH_3)_2$), 1.41 – 1.33 (m, 18 H, *o*-$CH(C\underline{H}_3)_2$), 1.18 – 1.05 (m, 18 H, 12 × *p*-$CH(C\underline{H}_3)_2$, 6 × *o*-$CH(C\underline{H}_3)_2$). **^{13}C{^{1}H}-NMR** (100.61 MHz, 300.2 K, C_6D_6): δ (ppm) = 149.5 (s, *o*-C_6H_2), 148.5 (br, C-1), 147.8 (s, *p*-C_6H_3), 146.6 (s, *o*-C_6H_2), 146.2 (s, *p*-C_6H_2), 137.0 (s, *i*-C_6H_2), 134.4 (d, $^{3}J_{31P\text{-}13C}$ =12.6 Hz, C-6), 134.0 – 133.6 (m, *o*-C_6H_5 + *i*-C_6H_3), 132.9 (d, $^{2}J_{31P\text{-}13C}$ = 9.7 Hz, *o*-C_6H_5), 132.3 – 132.1 (m, C-5 + *p*-C_6H_5), 131.6 (d, $^{1}J_{31P\text{-}13C}$ = 81.3 Hz, C-2), 131.4 (d, $^{4}J_{31P\text{-}13C}$ = 2.4 Hz, *p*-C_6H_5), 131.3 (d, $^{2}J_{31P\text{-}13C}$ = 6.3 Hz, C-3), 130.5 (s, *m*-C_6H_2), 129.2 (d, $^{3}J_{31P\text{-}13C}$ = 11.7 Hz, *m*-C_6H_5), 129.0 (s, *p*-C_6H_3), 128.7 (d, $^{3}J_{31P\text{-}13C}$ = 11.2 Hz, *m*-C_6H_5), 128.4 (d, $^{3}J_{31P\text{-}13C}$ = 9.4 Hz, C-4), 125.9 (d, $^{1}J_{31P\text{-}13C}$ = 63.4 Hz, *i*-C_6H_5), 123.9 (d, $^{1}J_{31P\text{-}13C}$ = 67.3 Hz, *i*-C_6H_5), 121.7 (s, *m*-C_6H_2), 121.6 (s, *m*-C_6H_2), 34.2 (s, *p*-$\underline{C}H(CH_3)_2$), 31.1 (s, *o*-$\underline{C}H(CH_3)_2$), 31.0 (s, *o*-$\underline{C}H(CH_3)_2$), 26.0 (s, *p*-$CH(\underline{C}H_3)_2$), 25.6 (s, *o*-$CH(\underline{C}H_3)_2$), 24.2 (s, *o*-$CH(\underline{C}H_3)_2$), 24.1 (s, *o*-$CH(\underline{C}H_3)_2$), 23.8 (s, *o*-$CH(\underline{C}H_3)_2$), 23.4 (s, *p*-$CH(\underline{C}H_3)_2$). **^{31}P{^{1}H}-NMR** (161.97 MHz, 300.2 K, C_6D_6): δ (ppm) = 1.0 (broad unresolved q). **^{11}B{^{1}H}-NMR** (128.37 MHz, 300.2 K, C_6D_6): δ (ppm) = 0.4 (d, $^{1}J_{31P\text{-}11B}$ = 122.8 Hz). **Elemental analysis:** Calculated (%) for $C_{54}H_{63}BAuCl_2GeP$: 59.27 C, 5.80 H. Found: 60.72 C, 6.25 H (elemental analyses of crystalline material was repeated several times leading always to large deviations).

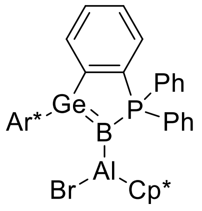

13: A solution of **1b** (131.7 mg, 145 µmol, 1 eq.) in benzene (3 mL) was added to $[Cp^{*}Al]_4$ (23.6 mg, 36.3 µmol, 0.25 eq.) followed by stirring and heating the mixture at 60 °C for 21 hours. The intense red solution changed color to dark orange during the reaction. After filtration, the solvent was evaporated under reduced pressure. The brownish residue was dissolved in *n*-pentane (2 mL). Slow evaporation of the solvent afforded red-orange crystals. Removing the mother liquor followed by washing the crystals with *n*-pentane (3 × 1 mL) and subsequently removing all residual solvent under reduced pressure afforded **13** (81.3 mg, 76.1 µmol, 52 %) in the form of a red-orange crystalline solid. Single crystals suitable for X-ray diffraction were obtained from a concentrated solution of **13** in toluene at room temperature. **^{1}H-NMR** (400.11 MHz, 299.2 K, C_6D_6): δ (ppm) = 7.55 – 7.45 (m, 4 H, *o*-C_6H_5), 7.41 – 7.35 (m, 2 H, *m*-C_6H_3), 7.35 – 7.30 (m, 2 H, *m*-C_6H_2), 7.24 – 7.18 (m, 1 H, *p*-C_6H_3), 7.10 – 7.00 (m, 9 H, *m*-C_6H_2, 3-$C_6\underline{H}_4$, *m*-C_6H_5, *p*-C_6H_5, H-3), 6.84 – 6.79 (m, 1 H, H-6), 6.78 – 6.72 (m, 1 H, H-5), 6.69 – 6.62 (m, 1 H, H-4), 3.27 (sept, $^{3}J_{H\text{-}H}$ = 6.7 Hz, 2 H, *o*-$C\underline{H}(CH_3)_2$), 3.07 (sept, $^{3}J_{H\text{-}H}$ = 6.8 Hz, 2 H, *o*-$C\underline{H}(CH_3)_2$), 2.94 (sept, $^{3}J_{H\text{-}H}$ = 6.9 Hz, 2 H, *p*-$C\underline{H}(CH_3)_2$), 1.67 (d, $^{3}J_{H\text{-}H}$ = 6.9 Hz, 6 H, *o*-$CH(C\underline{H}_3)_2$), 1.55 (s, 15 H, $C_5(C\underline{H}_3)_5$), 1.39 (d, $^{3}J_{H\text{-}H}$ = 6.9 Hz, 6 H, *p*-$CH(C\underline{H}_3)_2$), 1.30 (d, $^{3}J_{H\text{-}H}$ = 6.9 Hz, 6 H, *p*-$CH(C\underline{H}_3)_2$), 1.23 (d, $^{3}J_{H\text{-}H}$ = 6.8 Hz, 6 H, *o*-$CH(C\underline{H}_3)_2$), 1.18 (d, $^{3}J_{H\text{-}H}$ = 6.8 Hz, 6 H, *o*-$CH(C\underline{H}_3)_2$), 0.45 (d, $^{3}J_{H\text{-}H}$ = 6.7 Hz, 6 H, *o*-$CH(C\underline{H}_3)_2$). **^{13}C{^{1}H}-NMR** (100.62 MHz, 300.2 K, C_6D_6): δ (ppm) = 157.0 (d, $^{2}J_{31P\text{-}13C}$ = 40.8 Hz, C-1), 148.0 (s, *p*-C_6H_2), 147.8 (s, *o*-C_6H_2), 147.6 (d, $^{3}J_{31P\text{-}13C}$ = 23.7 Hz, *i*-C_6H_3), 147.0 (s, *o*-C_6H_3), 146.2 (s, *o*-C_6H_2), 138.7 (d, $^{1}J_{31P\text{-}13C}$ = 73.6 Hz, C-2), 138.7 (s, *i*-C_6H_2), 132.8 (d, $^{2}J_{31P\text{-}13C}$ = 10.0 Hz, *o*-C_6H_5), 131.5 (d, $^{1}J_{31P\text{-}13C}$ = 64.8 Hz, *i*-C_6H_5), 130.6 (d, $^{3}J_{31P\text{-}13C}$ = 17.6 Hz, C-6), 130.4 (d, $^{4}J_{31P\text{-}13C}$ = 1.7 Hz, *p*-C_6H_5), 130.1 (d, $^{4}J_{31P\text{-}13C}$ = 1.0 Hz, C-5), 129.9 (s, *m*-C_6H_3), 128.2 (d, $^{3}J_{31P\text{-}13C}$ = 10.8 Hz, *m*-C_6H_5), 127.1 (s, *p*-C_6H_3), 124.8 (d, $^{3}J_{31P\text{-}13C}$ = 7.0 Hz, C-4), 121.2 (s, m-C_6H_2), 115.3 (s, $\underline{C}_5(CH_3)_5$), 34.4 (s, *p*-$\underline{C}H(CH_3)_2$), 31.1 (s, *o*-$\underline{C}H(CH_3)_2$), 30.4 (s, *o*-$\underline{C}H(CH_3)_2$), 26.1 (*o*-$CH(\underline{C}H_3)_2$), 25.8 (s, *o*-$CH(\underline{C}H_3)_2$), 24.6 (s, *p*-$CH(\underline{C}H_3)_2$), 24.0 (s, *o*-$CH(\underline{C}H_3)_2$), 23.5 (s, *p*-$CH(\underline{C}H_3)_2$), 22.1 (s, *o*-$CH(\underline{C}H_3)_2$), 11.0 (s, $C_5(\underline{C}H_3)_5$). **^{11}B{^{1}H}-NMR** (128.37 MHz, 299.2 K, C_6D_6): δ (ppm) = 0.1 (s). **^{31}P{^{1}H}-NMR** (161.97 MHz, 299.2 K, C_6D_6) 36.6 (br). **Elemental analysis:** Calculated (%) for $C_{64}H_{78}AlBBrGeP$: 71.94 C, 7.36 H. Found: 72.07 C, 7.66 H.

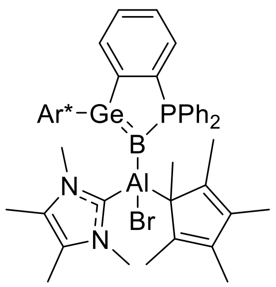

14: A solution of ^{Me}NHC (6.5 mg, 52.3 µmol, 1 eq.) in toluene (2 mL) was added dropwise to a solution of **13** (55.7 mg, 52.1 µmol, 1 eq.) in toluene (1 mL) and stirred for 80 minutes at room temperature. Removing the solvent under reduced pressure followed by co-evaporation of residual solvent with *n*-pentane (4 mL) afforded a red-orange powder. The product was extracted from the powder with toluene (7 mL) and filtered, followed by concentrating the filtrate to approx. 50 % of the initial volume under reduced pressure. Storage of the concentrated solution at –40 °C for four days afforded orange crystals. Washing the crystals with *n*-pentane (2 mL) and removing residual amounts of toluene by co-evaporation with *n*-pentane (2 mL) afforded **14** (40.5 mg, 34.0 µmol, 65.1 %) in the form of an orange crystalline solid. Single crystals suitable for X-Ray diffraction were obtained from a concentrated solution of **14** in toluene at room temperature. **^{1}H-NMR** (400.11 MHz, 299.2 K, THF-d_8): δ (ppm) = 7.66 – 7.52 (m, 4 H, *o*-C_6H_5), 7.49 – 7.42 (m, 1 H, *p*-C_6H_3), 7.36 – 7.28 (m, 3 H, *m*-C_6H_3, *p*-C_6H_5), 7.19 – 7.17 (m, 1 H, *m*-C_6H_2), 7.17 – 7.09 (m, 5 H, H-3, *m*-C_6H_2, *p*-C_6H_5, 2 × *m*-C_6H_5), 7.08 – 7.02 (m, 1 H, H-5), 7.00 – 6.93 (m, 3 H, *m*-C_6H_2, 2 × *m*-C_6H_5), 6.92 – 6.89 (m ,1 H, *m*-C_6H_2), 6.86 – 6.78 (m, 1 H, H-4), 6.54 – 6.47 (m, 1 H, H-6), 3.27 (sept, $^{3}J_{H\text{-}H}$ = 6.7 Hz, 1 H, *o*-$C\underline{H}(CH_3)_2$), 3.23 – 3.11 (m, 7 H, N-CH_3, *o*-$C\underline{H}(CH_3)_2$), 3.07 (sept, $^{3}J_{H\text{-}H}$ = 6.9 Hz, 1 H, *p*-$C\underline{H}(CH_3)_2$), 3.03 – 2.89 (m, 2 H, *o*-$C\underline{H}(CH_3)_2$, *p*-$C\underline{H}(CH_3)_2$), 2.65 (sept, $^{3}J_{H\text{-}H}$ = 6.8 Hz, 1 H, *o*-$C\underline{H}(CH_3)_2$), 1.84 (s, 6 H, C=C-$C\underline{H}_3$), 1.50 (s, 15 H, Cp*-$C\underline{H}_3$), 1.44 (d, $^{3}J_{H\text{-}H}$ = 6.9 Hz, 3 H, *p*-$CH(C\underline{H}_3)_2$), 1.41 (d, $^{3}J_{H\text{-}H}$ = 6.9 Hz, 3 H, *p*-$CH(C\underline{H}_3)_2$), 1.39 – 1.34 (m, 9 H, 3 × *o*-$CH(C\underline{H}_3)_2$, 6 × *p*-$CH(C\underline{H}_3)_2$), 1.28 (d, $^{3}J_{H\text{-}H}$ = 6.8 Hz, 3 H, *o*-$CH(C\underline{H}_3)_2$), 1.06 – 0.98 (m, 9 H, *o*-$CH(C\underline{H}_3)_2$), 0.86 (d, $^{3}J_{H\text{-}H}$ = 6.7 Hz, 3 H, *o*-$CH(C\underline{H}_3)_2$), 0.48 (d, $^{3}J_{H\text{-}H}$ = 6.7 Hz, 3 H, *o*-$CH(C\underline{H}_3)_2$), –0.04 (d, $^{3}J_{H\text{-}H}$ = 6.7 Hz, 3 H, *o*-$CH(C\underline{H}_3)_2$). **^{13}C{^{1}H}-NMR** (100.61 MHz, 299.2 K, THF-d_8): δ (ppm) = 162.8 (br, N$\underline{C}$N), 159.6 (d, $^{2}J_{31P\text{-}13C}$ = 43.2 Hz, C-1), 148.9 (d,

$^3J_{31P-13C}$ = 26.4 Hz, *i*-C_6H_3), 147.7 (s, *o*-C_6H_2), 147.4 (s, *o*-C_6H_3), 147.3 (s, *p*-C_6H_2), 147.2 (s, *o*-C_6H_2), 147.0 (s, *o*-C_6H_3), 147.0 (s, *p*-C_6H_2), 146.8 (s, *o*-C_6H_2), 146.2 (s, *o*-C_6H_2), 140.2 (d, $^1J_{31P-13C}$ = 68.9 Hz, C-2), 139.3 (s, *i*-C_6H_2), 132.8 (d, $^2J_{31P-13C}$ = 10.4 Hz, *o*-C_6H_5), 132.7 (d, $^2J_{31P-13C}$ = 9.9 Hz, *o*-C_6H_5), 132.6 (d, $^1J_{31P-13C}$ = 62.9 Hz, *i*-C_6H_5), 130.9 (d, $^1J_{31P-13C}$ = 61.9 Hz, *i*-C_6H_5), 130.5 – 130.1 (m, C-6, 2 × *m*-C_6H_3), 129.7 (d, $^4J_{31P13C}$ = 2.0 Hz, *p*-C_6H_5), 129.3 (br d, C-5), 129.2 (br d, *p*-C_6H_5), 127.5 (d, $^2J_{31P-13C}$ = 6.7 Hz, C-3), 127.1 (d, $^3J_{31P-13C}$ = 11.0 Hz, *m*-C_6H_5), 126.5 (d, $^3J_{31P-13C}$ = 10.3 Hz, *m*-C_6H_5), 125.8 (s, *p*-C_6H_3), 124.2 (s, $\underline{C}$=$\underline{C}$-CH_3), 123.9 (d, $^3J_{31P-13C}$ = 6.4 Hz, C-4), 121.1 (s, *m*-C_6H_2), 120.6 (s, *m*-C_6H_2), 120.3 (s, *m*-C_6H_2), 119.8 (s, *m*-C_6H_2), 116.7 (s, Cp*-C_q), 34.4 (s, *p*-$\underline{C}H(CH_3)_2$), 34.3 (s, *p*-$\underline{C}H(CH_3)_2$), 34.2 (s, N-$\underline{C}H_3$), 31.0 (s, *o*-$\underline{C}H(CH_3)_2$), 30.7 (s, *o*-$\underline{C}H(CH_3)_2$), 30.2 (s, *o*-$\underline{C}H(CH_3)_2$), 30.1 (s, *o*-$\underline{C}H(CH_3)_2$), 25.8 (s, 2 × *o*-CH($\underline{C}H(CH_3)_2$), 25.0 (s, *o*-CH($\underline{C}H_3)_2$), 24.8 (s, *o*-CH($\underline{C}H_3)_2$), 23.9 (s, *p*-CH($\underline{C}H_3)_2$), 23.7 (s, *p*-CH($\underline{C}H_3)_2$), 23.7 (s, *p*-CH($\underline{C}H_3)_2$), 23.6 (s, *p*-CH($\underline{C}H_3)_2$), 23.1 (s, *o*-CH($\underline{C}H_3)_2$), 22.6 (s, *o*-CH($\underline{C}H_3)_2$), 21.5 (s, *o*-CH($\underline{C}H_3)_2$),21.5 (s, *o*-CH($\underline{C}H_3)_2$), 12.4 (s, Cp*-$\underline{C}H_3$), 7.3 (s, C=C-$\underline{C}H_3$). **^{11}B{^{1}H}-NMR** (128.37 MHz, 299.2 K, THF-d_8): δ (ppm) = 3.6 (br). **^{31}P{^{1}H}-NMR** (161.97 MHz, 299.2 K, THF-d_8): δ (ppm) = 35.2 (br). **Elemental analysis:** Calculated (%) for $C_{71}H_{90}AlBBrGeN_2P \cdot C_7H_8$: 72.91 C, 7.61 H, found: 73.35 C, 7.826 H.

Data of crystal structure analyses

Table SI1. Data of compounds **2**-**7**.

	(**2**)	(**3**)	(**4**)	(**5**)	(**6**)	(**7**)
Empirical formula	$C_{73}H_{91.03}BBrGeN_2P$	$C_{70}H_{90}BClGeNP$	$C_{65}H_{78}BGeNP$, $C_{32}H_{12}BF_{24}$	$C_{55}H_{63}BBrGeOP$	$C_{71}H_{63}AlBF_{36}GeO_5P$	$C_{60}H_{68}BGePSe$
M_r / g mol^{-1}	1190.78	1095.24	1850.90	934.33	1821.56	982.47
λ / Å	0.71073	0.71073	0.71073	0.71073	0.71073	0.71073
T / K	100(2)	100(2)	100(2)	100(2)	120(2)	100(2)
Crystal system	monoclinic	monoclinic	triclinic	monoclinic	monoclinic	triclinic
Space group	$P2_1/c$	P 21/n	P -1	P 21/c	P 21/n	P -1
Z	4	4	2	4	4	2
a / Å	14.6697(2)	18.2700(6)	12.8385(5)	10.5731(4)	17.2296(5)	11.7124(4)
b / Å	25.6742(4)	17.3395(5)	16.5703(6)	28.1523(9)	13.1098(4)	13.1092(4)
c / Å	17.2849(2)	19.7561(6)	21.3396(8)	16.5798(6)	34.3624(11)	18.2445(6)
α / °	90	90	91.049(2)	90	90	107.1810(10)
β / °	92.4820(10)	100.445(2)	98.702(2)	90.754(2)	94.446(2)	98.0640(10)
γ / °	90	90	93.377(2)	90	90	100.1540(10)
V / Å^3	6503.95(15)	6154.9(3)	4478.0(3)	4934.7(3)	7738.3(4)	2578.65(15)
D_c / g cm^{-3}	1.216	1.182	1.373	1.258	1.564	1.265
μ / mm^{-1}	1.152	0.608	0.457	1.500	0.563	1.368
F(000)	2520	2344	1904	1952	3672	1028
Crystal size / mm	0.253 × 0.198 × 0.186	0.332 × 0.188 × 0.173	0.366 × 0.281 × 0.198	0.348×0.316×0.296	0.378 × 0.352 × 0.212	0.32×0.28×0.24
θ range / °	2.109 - 27.900	3.556 - 30.556	2.60 - 25.88	2.41 - 27.06	1.663 - 30.534	2.55 - 31.58
Limiting indices	-19 < h < 19	-26 < h < 26	-15 < h < 16	-13 < h < 13	-24 < h < 24	-17 < h < 17
	-32 < k < 33	-24 < k < 24	-21 < k < 21	-36 < k < 36	-18 < k < 18	-19 < k < 19
	-22 < l < 22	-28 < l < 28	-27 < l < 27	-21 < l < 21	-48 < l < 49	-26 < l < 26
Reflects. collect.	110665	152898	84154	108423	217520	69710
Indepdnt Reflects	15484	18782	19540	11002	23614	16891
R_{int}	0.0713	0.0622	0.0652	0.0667	0.0426	0.0302
Completeness	0.994	0.995	0.982	0.994	0.997	0.975
Absorp. Corr.	numerical	multi-scan	multi-scan	multi-scan	multi-scan	multi-scan
Trans.(max., min.)	1.0000, 0.9257	0.7461, 0.7072	0.7455, 0.6893	0.7455, 0.6739	0.7461, 0.6883	0.7463, 0.7095
Parameters/restraints	793	720	1163	553	1436	589
R_1, ωR_2 [$I > 2\sigma(I)$]	0.0406, 0.0879	0.0424, 0.1001	0.0501, 0.1034	0.0328, 0.0671	0.0535, 0.1336	0.0350, 0.0902
R_1, ωR_2 (all data)	0.0659, 0.0981	0.0658, 0.1110	0.0839, 0.1163	0.0505, 0.0727	0.0718, 0.1449	0.0438, 0.0945
GooF on F^2	1.008	1.026	1.015	1.037	1.020	1.053
$\Delta\rho_{max,min}$ / e·Å^{-3}	1.530, -0.669	0.951, -0.682	1.547, -0.857	0.765, -0.327	1.575, -0.931	0.947, -1.348
CCDC	2350052	2350060	2350062	2350063	2350059	2350055

Table SI2. Data of compounds **8**-**14**.

	(**8**)	(**9**)	(**10**)	(**11**)	(**12**)	(**13**)	(**14**)
Empirical formula	$C_{61}H_{69}BBrGeO_2P$	$C_{60}H_{74}BBrGeP$	$C_{58}H_{63}BBrFeGe O_4P$	2(C_{54}H63BBr_2CuGeP), C_5H_{12}	$C_{58}H_{71}AuBCl_2GeOP$	$C_{64}H_{78}AlBBrGeP$	$C_{85}H_{106}AlBBrGeN_2P$
M_r / g mol^{-1}	1028.45	989.47	1074.23	2171.72	1166.38	1068.52	1376.99
λ / Å	0.71073	0.71073	0.71073	0.71073	0.71073	0.71073	0.71073
T / K	100(2)	100(2)	106(2)	100(2)	100(2)	100(2)	100(2)
Crystal system	triclinic	triclinic	trigonal	monoclinic	monoclinic	monoclinic	triclinic
Space group	P -1	P -1	P 32 2 1	P 21/c	P 21/c	P 21/c	P -1
Z	2	2	6	2	4	8	2
a / Å	9.6665(2)	10.8284(3)	20.0501(4)	19.4738(4)	19.4927(4)	23.3641(5)	13.5727(5)
b / Å	16.5723(4)	11.5846(3)	20.0501(4)	15.6084(4)	15.5941(3)	17.9056(4)	14.9983(6)
c / Å	16.9238(4)	22.5224(6)	27.7768(6)	18.1663(4)	18.0318(3)	28.2604(6)	19.1435(7)
α / °	84.989(2)	88.3060(10)	90	90	90	90	91.740(2)
β / °	79.9240(10)	81.9350(10)	90	104.3940(10)	104.0510(10)	107.7390(10)	103.407(2)
γ / °	86.3450(10)	70.6680(10)	120	90	90	90	93.221(2)
V / Å^3	2655.86(11)	2639.17(12)	9670.4(4)	5348.4(2)	5317.15(18)	11260.6(4)	3781.0(2)
D_c / g cm^{-3}	1.286	1.245	1.107	1.349	1.457	1.261	1.209
μ / mm^{-1}	1.401	1.404	1.373	2.518	3.490	1.336	1.011
F(000)	1076	1042	3336	2236	2368	4496	1460
Crystal size / mm	0.27 × 0.25 × 0.23	0.24 × 0.22 × 0.20	0.214 × 0.189 × 0.127	0.283 × 0.192 × 0.147	0.22 × 0.17 × 0.15	0.221 × 0.216 × 0.188	0.387 × 0.365 × 0.218
θ range / °	2.914 - 29.171	3.163 - 27.946	3.189 - 28.340	2.61 - 31.99	1.693 - 27.210	3.27 - 29.32	2.526 - 33.767
Limiting indices	-12 < h < 13	-14 < h < 14	-21 < h < 26	-29 < h < 29	-25 < h < 24	-35 < h < 35	-21 < h < 21
	-22 < k < 22	-15 < k < 15	-26 < k < 26	-23 < k < 23	-20 < k < 20	-26 < k < 24	-23 < k < 23
	0 < l < 23	-29 < l < 29	-37 < l < 37	-25 < l < 27	-23 < l < 22	-42 < l < 42	-29 < l < 29
Reflects. collect.	32620	113470	161290	107763	86214	272027	165739
Indepdnt Reflects	32620	12591	16049	18602	11780	40215	29908
R_{int}		0.0421	0.0622	0.0416	0.0780	0.0828	0.0404
Completeness	0.991	0.993	0.995	0.997	0.995	0.996	0.987
Absorp. Corr.	multi-scan	multi-scan	multi-scan	multi-scan	multi-scan	multi-scan	multi-scan
Trans.(max., min.)	0.745, 0.657	0.7456, 0.6990	0.7457, 0.6808	0.7463, 0.6404	0.7455, 0.6805	0.7464, 0.6731	0.810, 0.696
Parameters/restraints	616	591	634	606	632	1277	1052
R_1, ωR_2 [$I > 2\sigma(I)$]	0.0421, 0.0906	0.0280, 0.0707	0.0321, 0.0836	0.0320, 0.0742	0.0368, 0.0732	0.0443, 0.0929	0.0303, 0.0760
R_1, ωR_2 (all data)	0.0658, 0.0996	0.0328, 0.0729	0.0400, 0.0866	0.0468, 0.0798	0.0601, 0.0804	0.0842, 0.1065	0.0437, 0.0820
GooF on F^2	1.007	1.042	1.038	1.018	1.022	1.007	1.017
$\Delta\rho_{max,min}$ / e·Å^{-3}	0.500, -0.411	0.864, -1.034	0.367, -0.337	1.000, -1.005	1.223, -0.853	1.436, -1.255	0.594, -0.518
CCDC	2350051	2350056	2350058	2350054	2350053	2350057	2350061

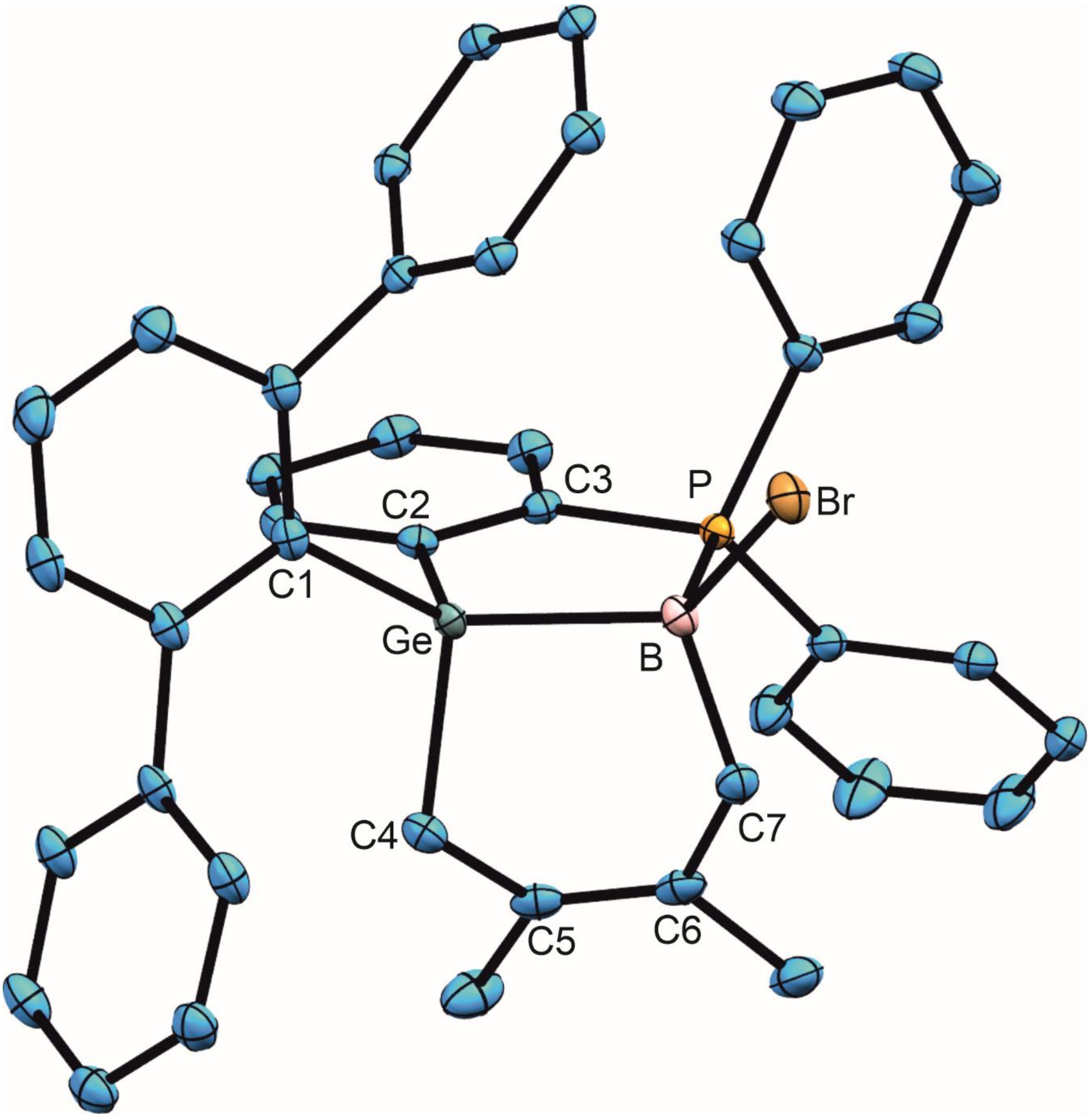

Figure SI1. Ortep of the molecular structure of (**9**). Thermal ellipsoids are shown at 50 % probability level. Hydrogen atoms and *i*Pr groups have been omitted. Interatomic distances in Å and angles in (°): Ge-B 2.1089(17), B-C7 1.626(2), C6-C7 1.521(2), C5-C6 1.342(2), C4-C5 1.507(2), Ge-C4 1.9842(15).

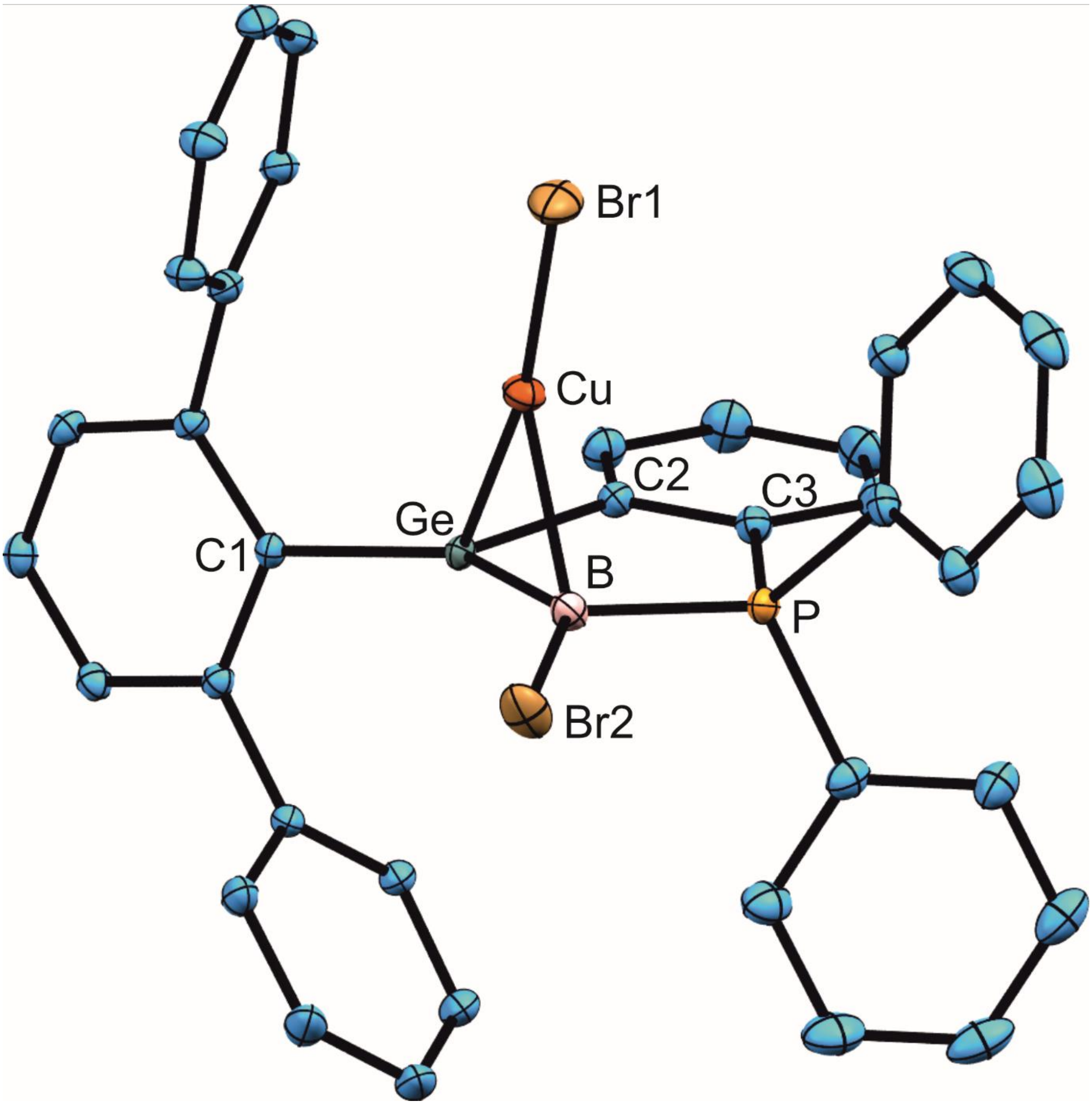

Figure SI2. Ortep of the molecular structure of (**11**). Thermal ellipsoids are shown at 50 % probability level. Hydrogen atoms and *i*Pr groups have been omitted. Interatomic distances in Å and angles in (°): Ge-B 1.9266(18), Cu-B 2.1049(18), Ge-Cu 2.4627(3), B-P 1.9112(17), Cu-Br1 2.2632(3), B-Br2 1.9421(18), B-Ge-Cu 55.7(1), B-Cu-Ge 49.1(1), Cu-B-Ge 75.2(1), Br2-B-Cu 106.9(1), Br1-Cu-Ge 164.7(1).

NMR spectroscopy

NMR spectra of compound **2**.

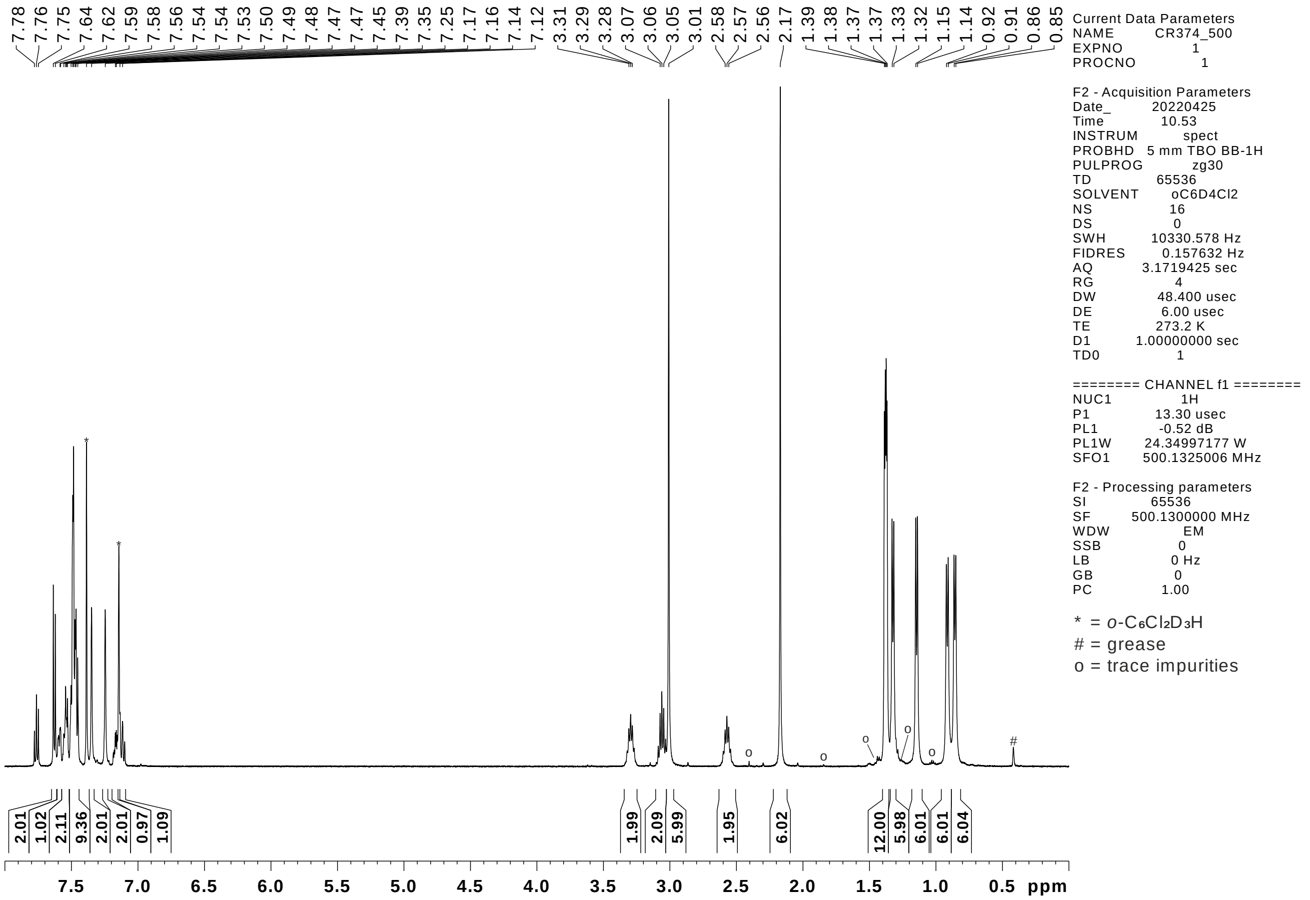

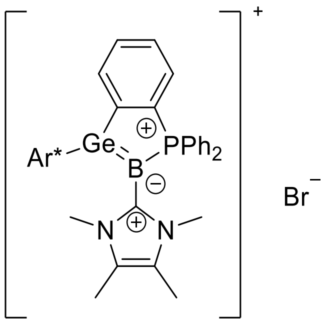

Figure SI3. ^{1}H NMR spectrum of compound **2**.

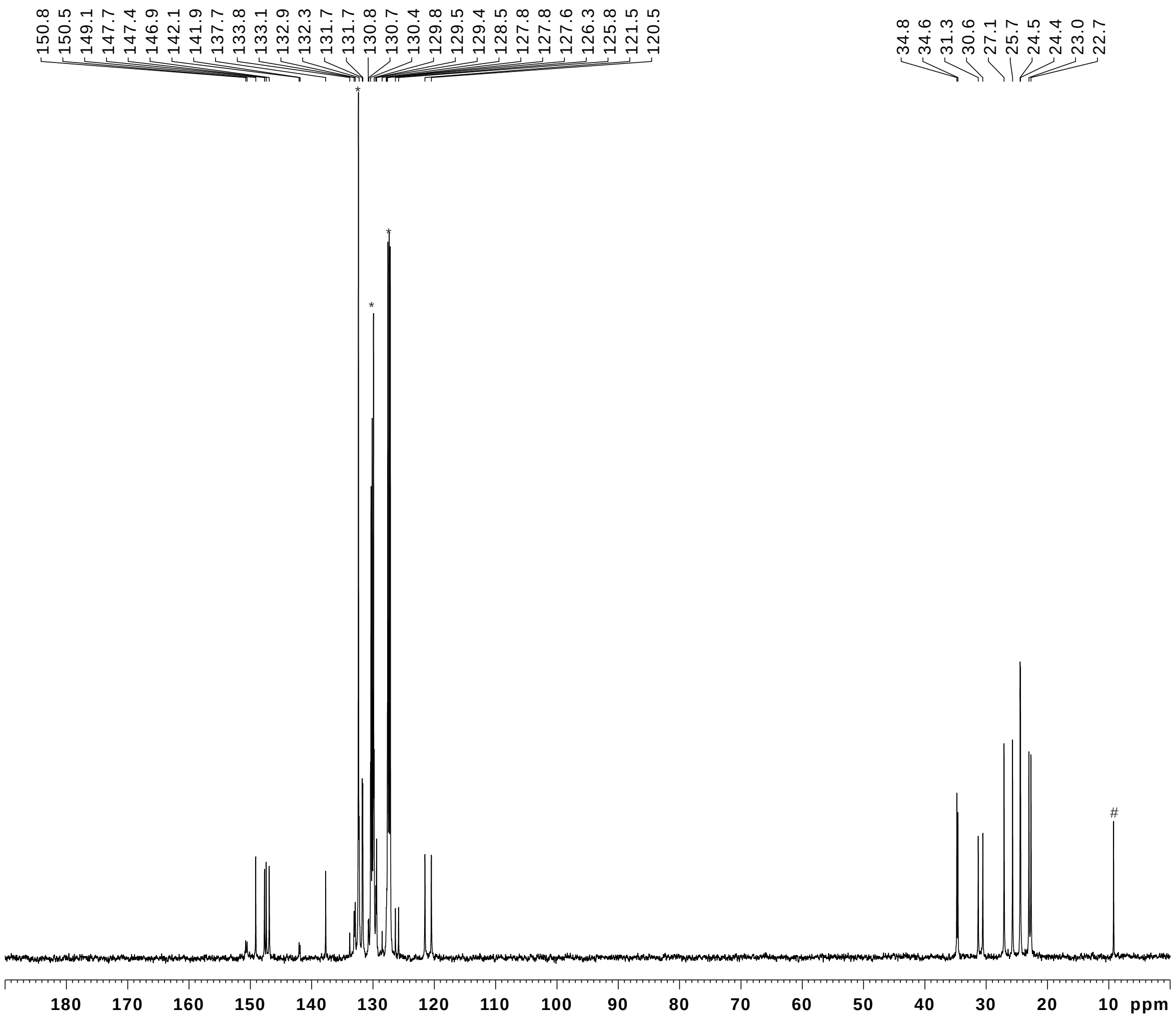


```
Current Data Parameters
NAME          CR374_500
EXPNO                 6
PROCNO                1

F2 - Acquisition Parameters
Date_          20220425
Time              17.07
INSTRUM           spect
PROBHD   5 mm TBO BB-1H
PULPROG           udeft
TD                27268
SOLVENT        oC6D4Cl2
NS                 1200
DS                    0
SWH        37878.789 Hz
FIDRES      1.389130 Hz
AQ        0.3599376 sec
RG                 2050
DW           13.200 usec
DE             6.00 usec
TE              273.2 K
D1       1.00000000 sec
D11      0.03000000 sec
D12      0.00002000 sec
D20    100.00000000 sec
TD0                   1

======== CHANNEL f1 ========
NUC1                13C
P1             8.32 usec
P13         2000.00 usec
P26          500.00 usec
PL1             0.40 dB
PL1W      76.51497650 W
SFO1     125.7728799 MHz
SP8            10.16 dB
SP13           10.16 dB
SPNAM[8] Crp60,0.5,20.1
SPNAM[13   Crp60comp.4
SPOAL8            0.500
SPOAL13           0.500
SPOFFS8            0 Hz
SPOFFS13           0 Hz

======== CHANNEL f2 ========
CPDPRG[2        waltz16
NUC2                 1H
PCPD2          80.00 usec
PL2            -0.52 dB
PL12           15.06 dB
PL2W      24.34997177 W
PL12W      0.67374945 W
SFO2     500.1325006 MHz

F2 - Processing parameters
SI               262144
SF       125.7577890 MHz
WDW                  EM
SSB                   0
LB              5.00 Hz
GB                    0
PC                 1.40
```

* = *o*-$C_6Cl_2D_4$
# = grease

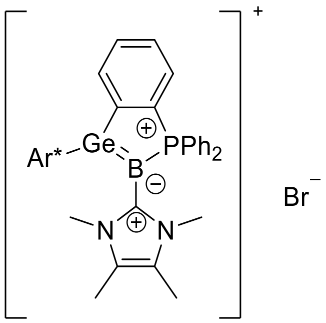

Figure SI4. $^{13}C\{^{1}H\}$ NMR spectrum of compound **2**.

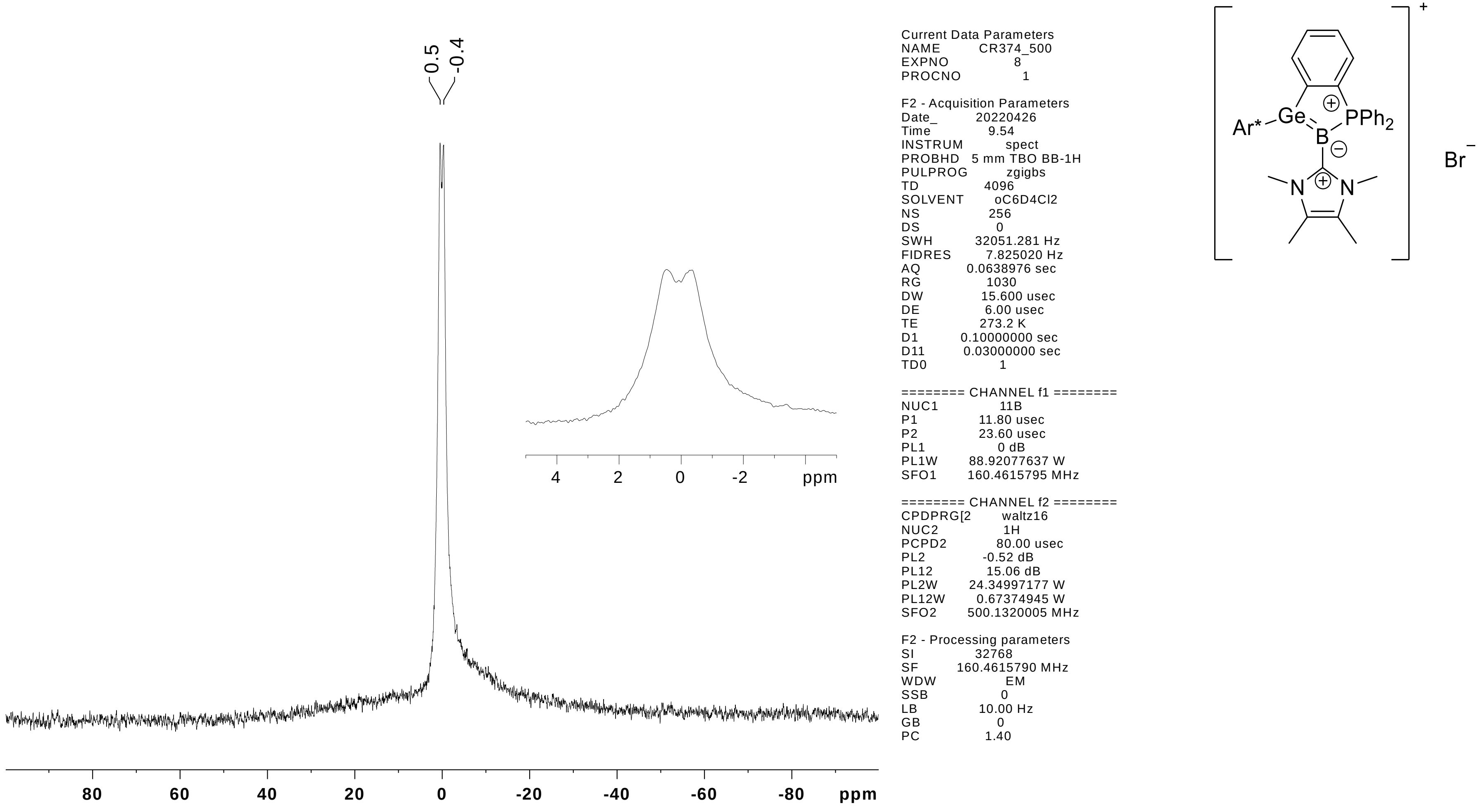

Figure SI5. $^{11}B\{^{1}H\}$ NMR spectrum of compound **2**.

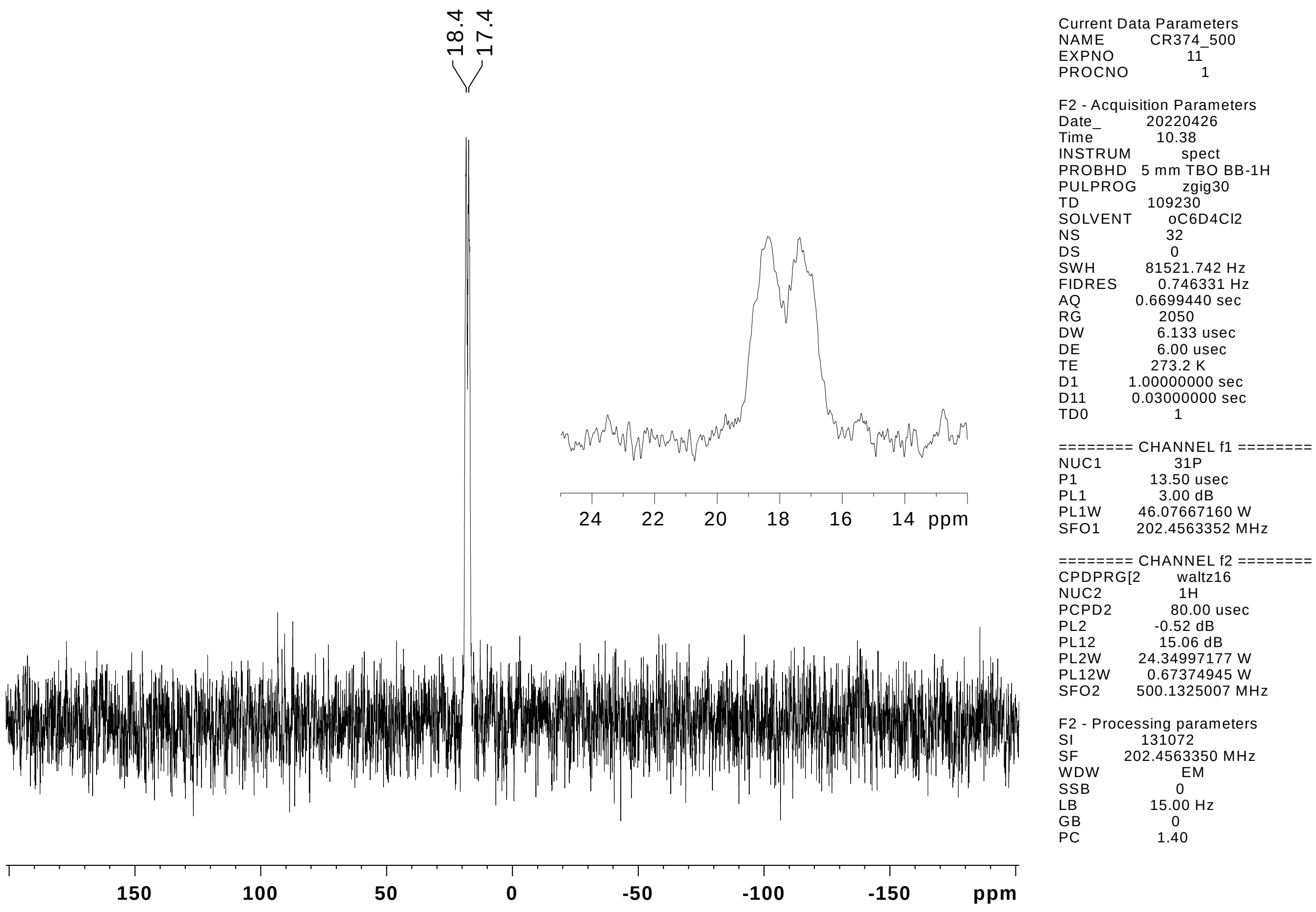

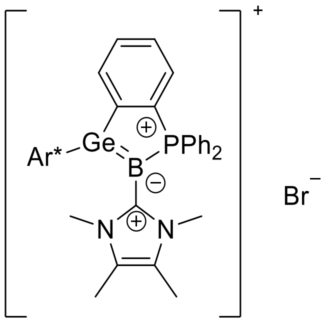

Figure SI6. $^{31}P\{^{1}H\}$ NMR spectrum of compound **2**.

NMR spectra of compound **3**.

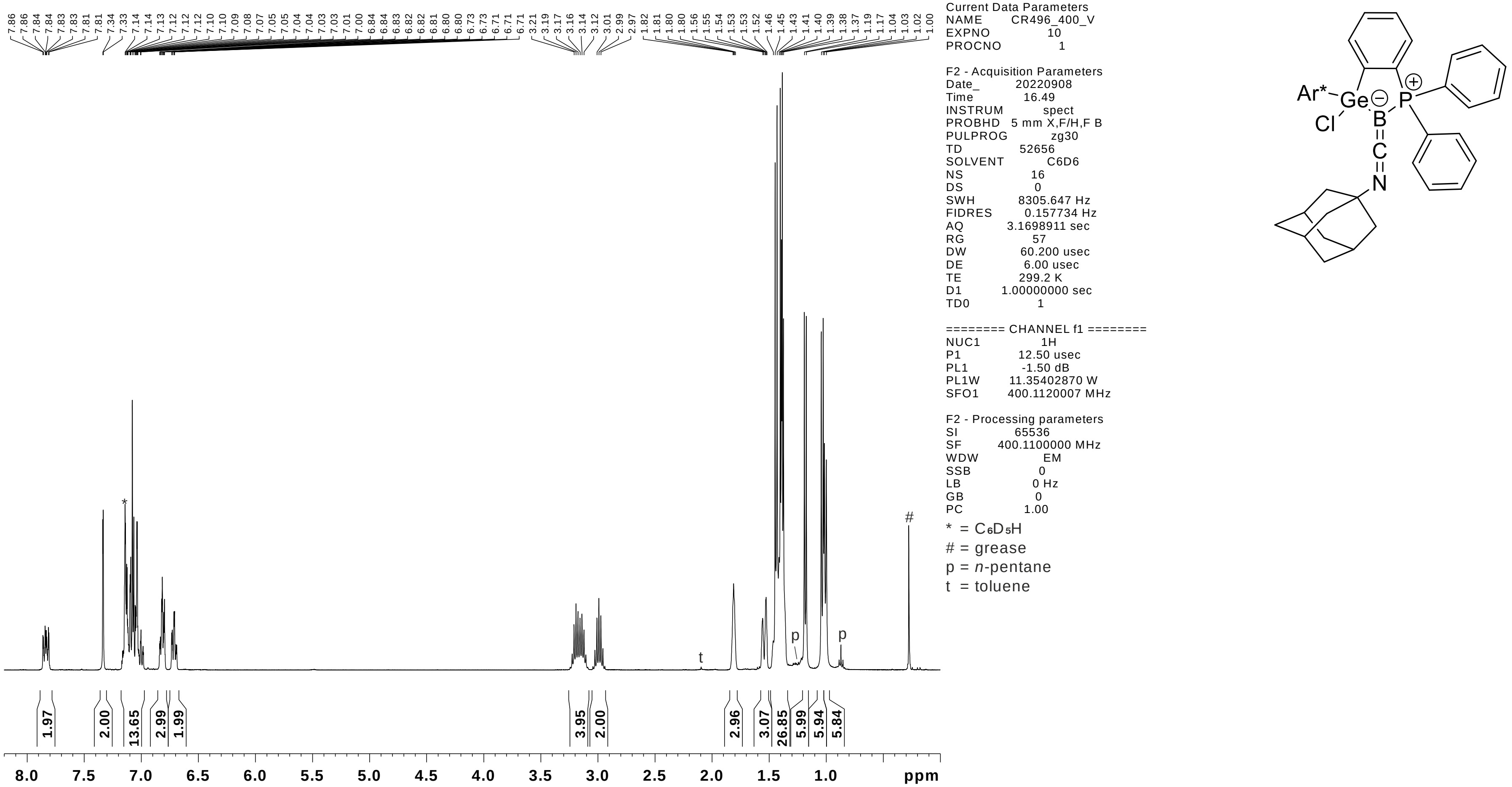

Figure SI7. ^{1}H NMR spectrum of compound **3**.

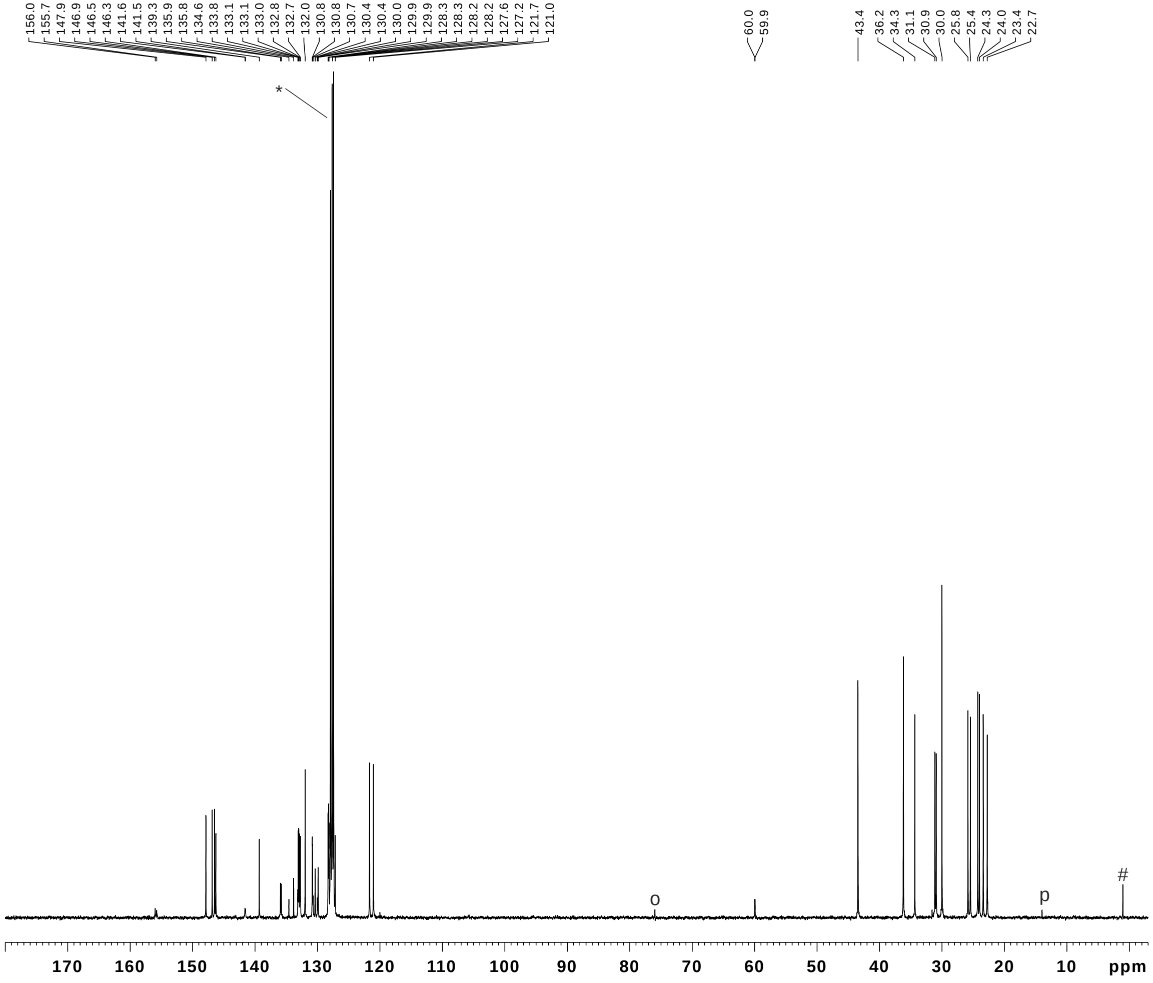

Current Data Parameters
NAME CR496_400_V
EXPNO 15
PROCNO 1

F2 - Acquisition Parameters
Date_ 20220909
Time 1.59
INSTRUM spect
PROBHD 5 mm X,F/H,F B
PULPROG udeft
TD 22218
SOLVENT C6D6
NS 5700
DS 0
SWH 30864.197 Hz
FIDRES 1.389153 Hz
AQ 0.3599316 sec
RG 32800
DW 16.200 usec
DE 6.00 usec
TE 299.2 K
D1 3.00000000 sec
D11 0.03000000 sec
D12 0.00002000 sec
D20 100.00000000 sec
TD0 1

======== CHANNEL f1 ========
NUC1 13C
P1 14.40 usec
P13 2000.00 usec
P26 500.00 usec
PL1 -5.90 dB
PL1W 117.26847076 W
SFO1 100.6198135 MHz
SP8 -0.91 dB
SP13 1.39 dB
SPNAM[8] Crp60,0.5,20.1
SPNAM[13 Crp60comp.4
SPOAL8 0.500
SPOAL13 0.500
SPOFFS8 0 Hz
SPOFFS13 0 Hz

======== CHANNEL f2 ========
CPDPRG[2 waltz16
NUC2 1H
PCPD2 90.00 usec
PL2 -1.50 dB
PL12 15.65 dB
PL2W 11.35402870 W
PL12W 0.21885175 W
SFO2 400.1120007 MHz

F2 - Processing parameters
SI 131072
SF 100.6077400 MHz
WDW EM
SSB 0
LB 2.00 Hz
GB 0
PC 1.40

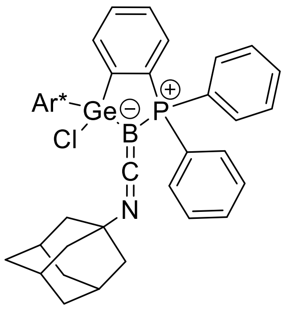

p = *n*-pentane

# = grease

o = trace impurity

* = C_6D_6

Figure SI8. $^{13}C\{^1H\}$ NMR spectrum of compound **3**.

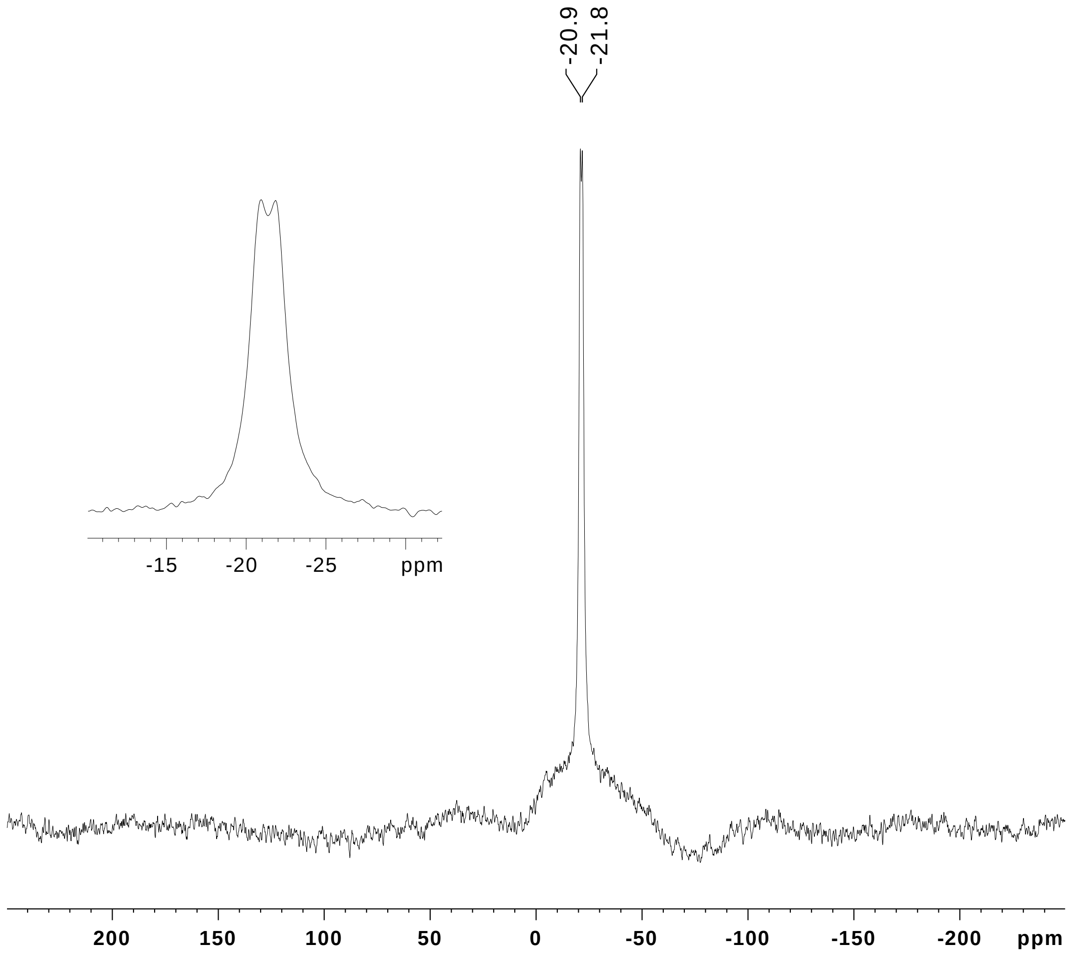

Current Data Parameters
NAME CR496_400_V
EXPNO 12
PROCNO 1

F2 - Acquisition Parameters
Date_ 20220908
Time 16.54
INSTRUM spect
PROBHD 5 mm X,F/H,F B
PULPROG zgbsig
TD 8192
SOLVENT C6D6
NS 1024
DS 0
SWH 64102.562 Hz
FIDRES 7.825020 Hz
AQ 0.0638976 sec
RG 1820
DW 7.800 usec
DE 6.00 usec
TE 299.2 K
D1 0.10000000 sec
D11 0.03000000 sec
TD0 1

======== CHANNEL f1 ========
NUC1 11B
P1 15.00 usec
P2 30.00 usec
PL1 -3.00 dB
SFO1 128.3711884 MHz

======== CHANNEL f2 ========
CPDPRG[2 garp
NUC2 1H
PCPD2 90.00 usec
PL2 -1.50 dB
PL12 15.65 dB
PL2W 11.35402870 W
PL12W 0.21885175 W
SFO2 400.1108002 MHz

F2 - Processing parameters
SI 8192
SF 128.3711880 MHz
WDW EM
SSB 0
LB 50.00 Hz
GB 0
PC 1.40

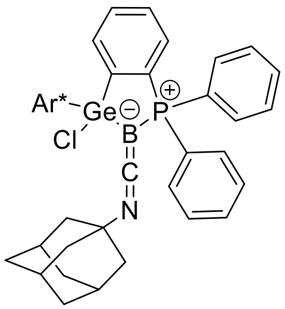

Figure SI9. $^{11}B\{^{1}H\}$ NMR spectrum of compound **3**.

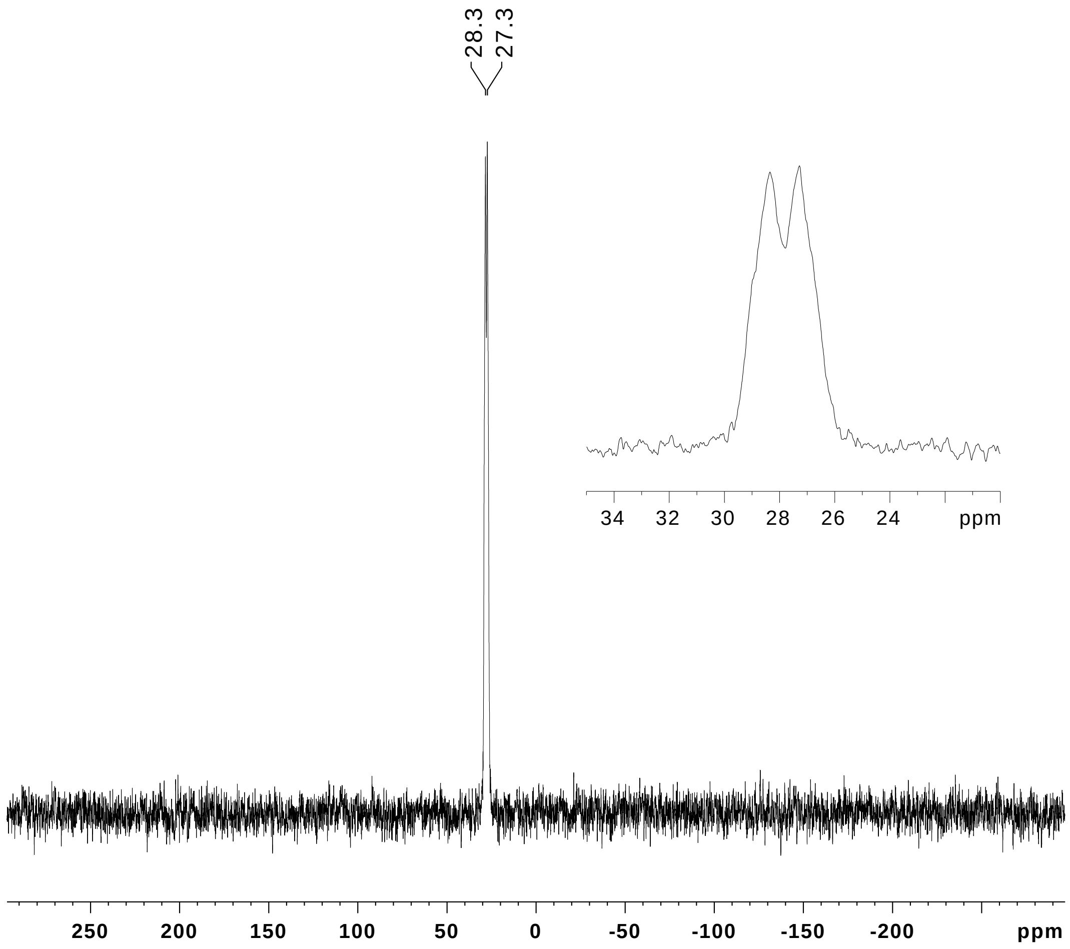

Current Data Parameters
NAME CR496_400_V
EXPNO 13
PROCNO 1

F2 - Acquisition Parameters
Date_ 20220908
Time 17.01
INSTRUM spect
PROBHD 5 mm X,F/H,F B
PULPROG zgig30b
TD 88150
SOLVENT C6D6
NS 256
DS 0
SWH 96153.844 Hz
FIDRES 1.090798 Hz
AQ 0.4583800 sec
RG 23100
DW 5.200 usec
DE 6.00 usec
TE 299.2 K
D1 1.00000000 sec
D11 0.03000000 sec
TD0 1

======== CHANNEL f1 ========
NUC1 31P
P1 14.50 usec
PL1 -4.00 dB
PL1W 56.78615952 W
SFO1 161.9674970 MHz

======== CHANNEL f2 ========
CPDPRG[2 waltz16
NUC2 1H
PCPD2 90.00 usec
PL2 -1.50 dB
PL12 15.65 dB
PL2W 11.35402870 W
PL12W 0.21885175 W
SFO2 400.1120007 MHz

F2 - Processing parameters
SI 131072
SF 161.9674970 MHz
WDW EM
SSB 0
LB 3.00 Hz
GB 0
PC 1.40

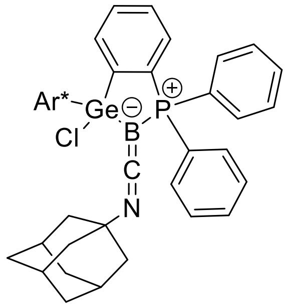

Figure SI10. $^{31}P\{^1H\}$ NMR spectrum of compound **3**.

NMR spectra of compound **4**.

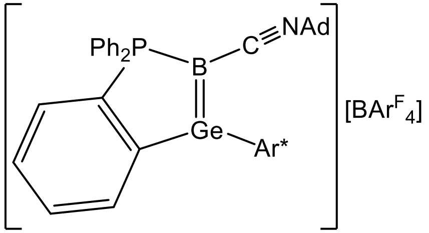

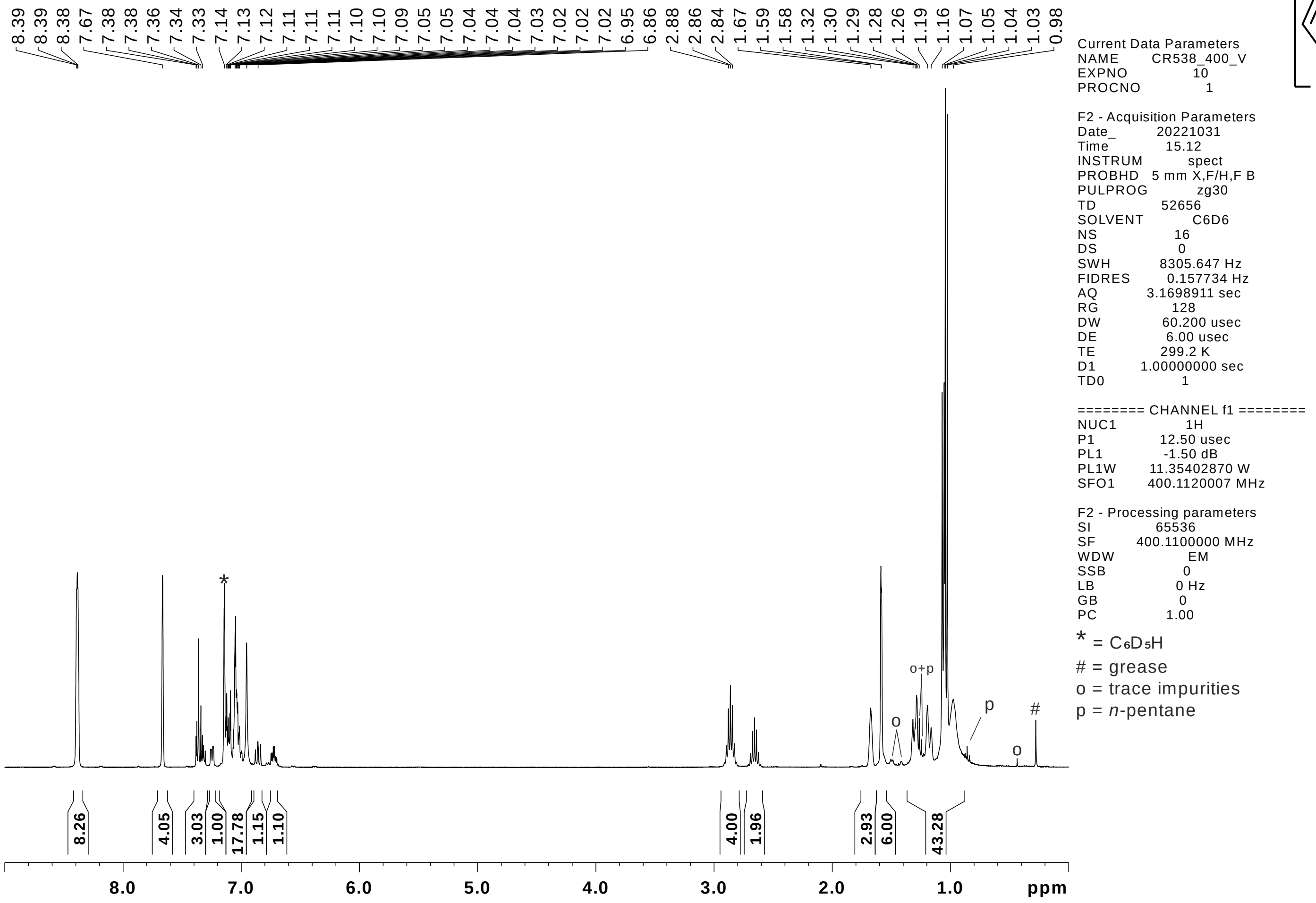

Figure SI11. ^{1}H NMR spectrum of compound **4**.

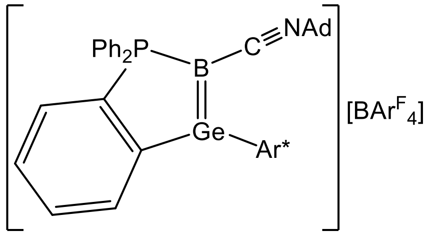

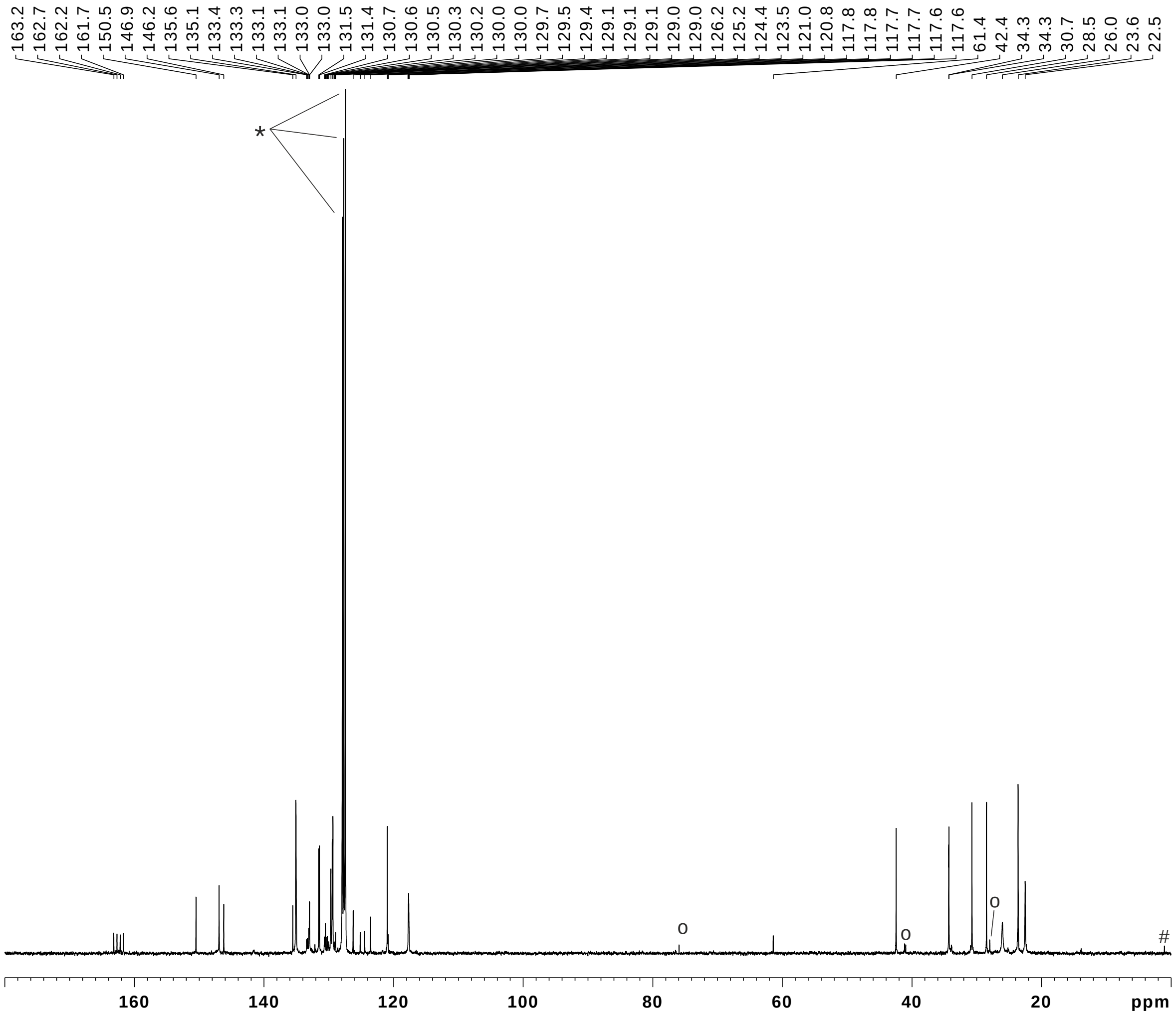

Current Data Parameters
NAME CR538_400_V
EXPNO 14
PROCNO 1

F2 - Acquisition Parameters
Date_ 20221101
Time 1.59
INSTRUM spect
PROBHD 5 mm X,F/H,F B
PULPROG udeft
TD 22218
SOLVENT C6D6
NS 5700
DS 0
SWH 30864.197 Hz
FIDRES 1.389153 Hz
AQ 0.3599316 sec
RG 32800
DW 16.200 usec
DE 6.00 usec
TE 299.2 K
D1 3.00000000 sec
D11 0.03000000 sec
D12 0.00002000 sec
D20 100.00000000 sec
TD0 1

======== CHANNEL f1 ========
NUC1 13C
P1 14.40 usec
P13 2000.00 usec
P26 500.00 usec
PL1 -5.90 dB
PL1W 117.26847076 W
SFO1 100.6198135 MHz
SP8 -0.91 dB
SP13 1.39 dB
SPNAM[8] Crp60,0.5,20.1
SPNAM[13 Crp60comp.4
SPOAL8 0.500
SPOAL13 0.500
SPOFFS8 0 Hz
SPOFFS13 0 Hz

======== CHANNEL f2 ========
CPDPRG[2 waltz16
NUC2 1H
PCPD2 90.00 usec
PL2 -1.50 dB
PL12 15.65 dB
PL2W 11.35402870 W
PL12W 0.21885175 W
SFO2 400.1120007 MHz

F2 - Processing parameters
SI 131072
SF 100.6077400 MHz
WDW EM
SSB 0
LB 2.00 Hz
GB 0
PC 1.40

# = grease
o = trace impurity
* = C_6D_6

Figure SI12. $^{13}C\{^1H\}$ NMR spectrum of compound **4**.

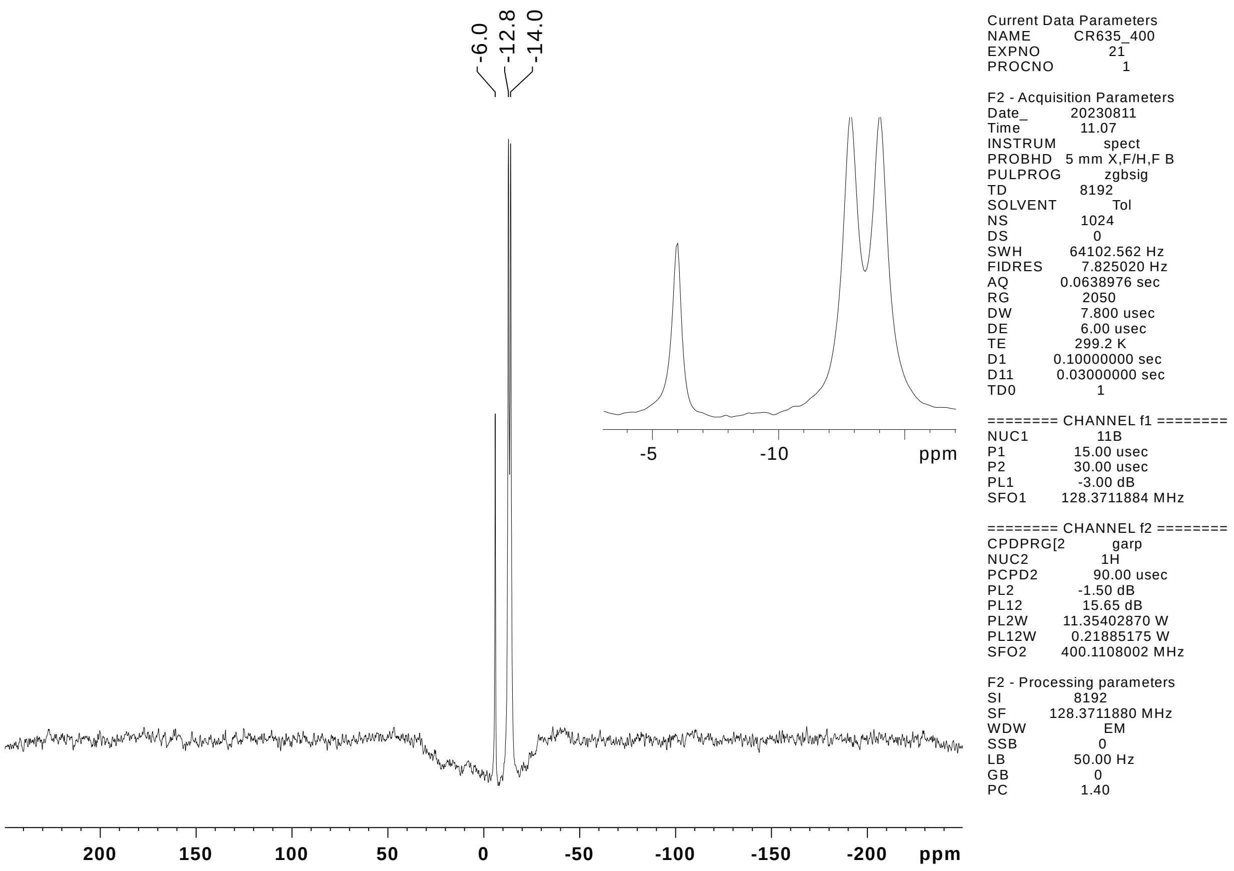

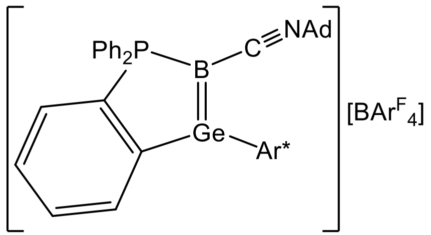

Figure SI13. $^{11}B\{^{1}H\}$ NMR spectrum of compound **4**.

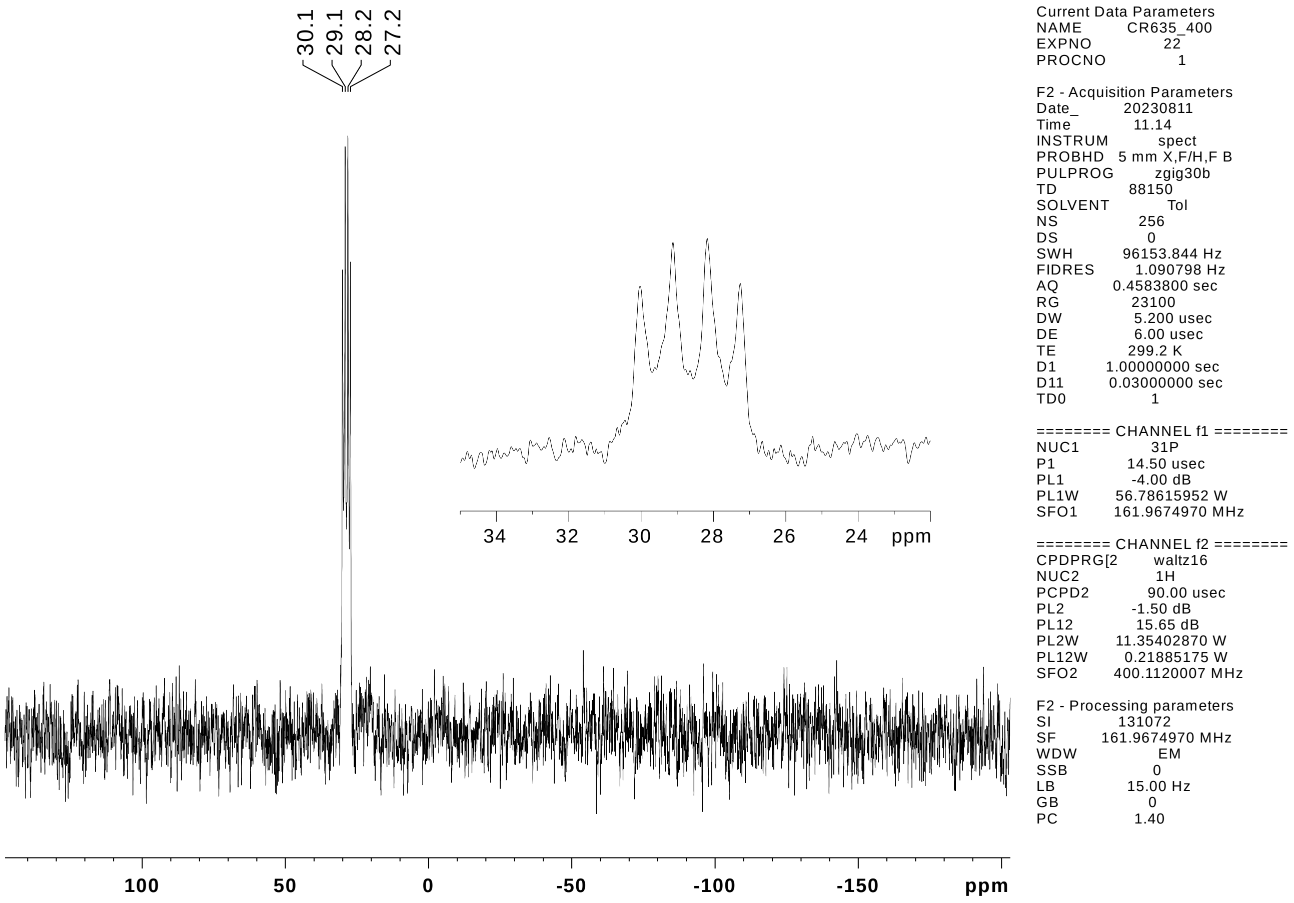

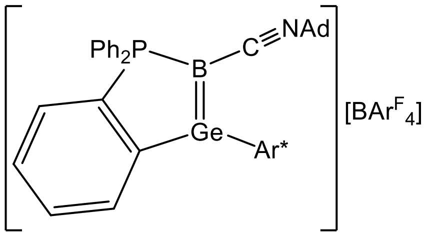

Figure SI14. $^{31}P\{^{1}H\}$ NMR spectrum of compound **4**.

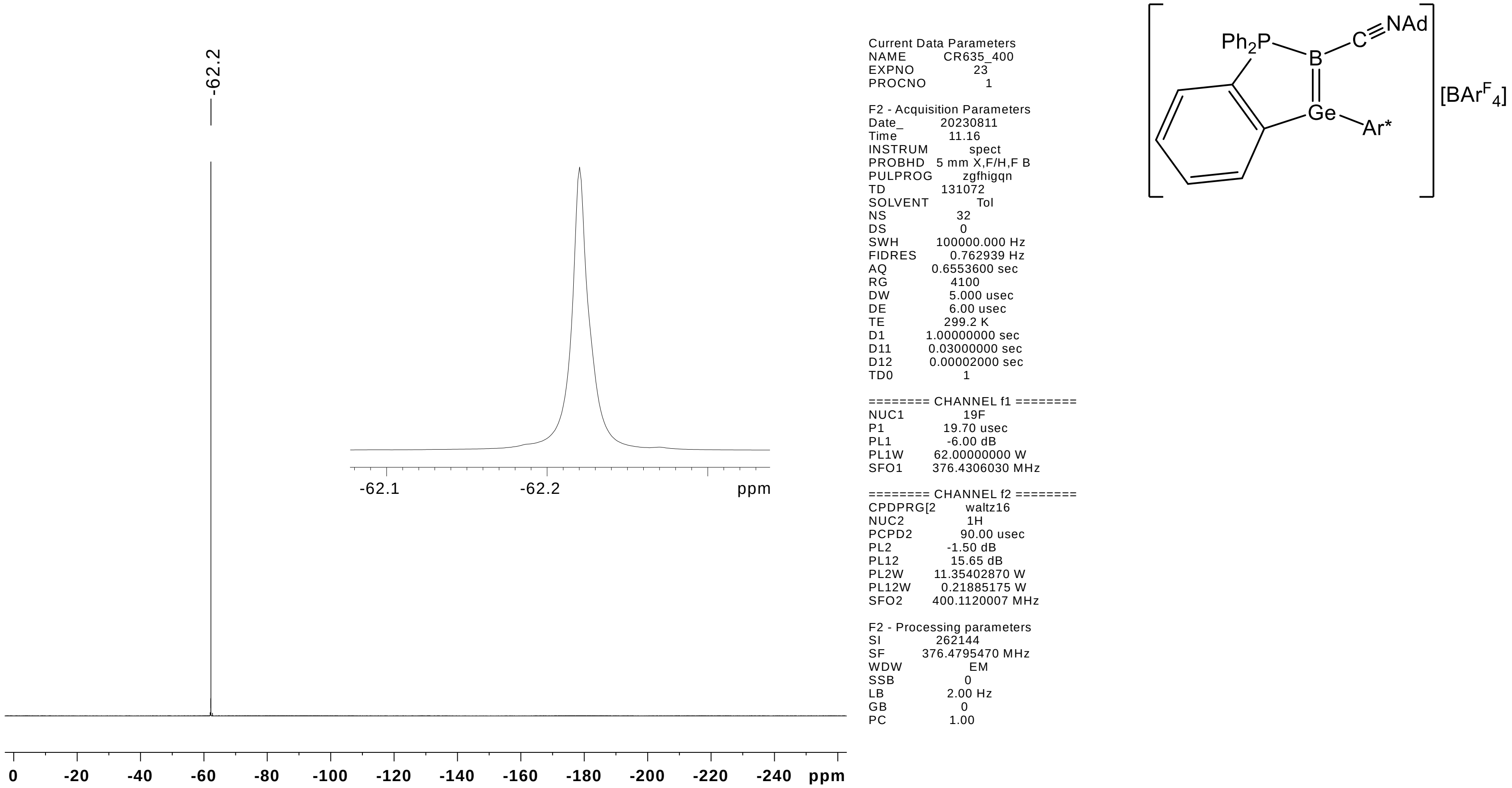

Figure SI15. $^{19}F\{^{1}H\}$ NMR spectrum of compound **4**.

NMR spectra of compound **5**.

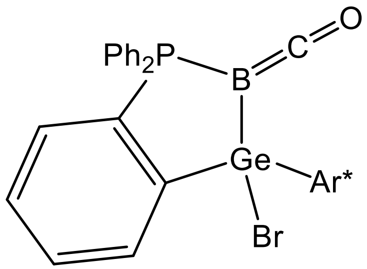

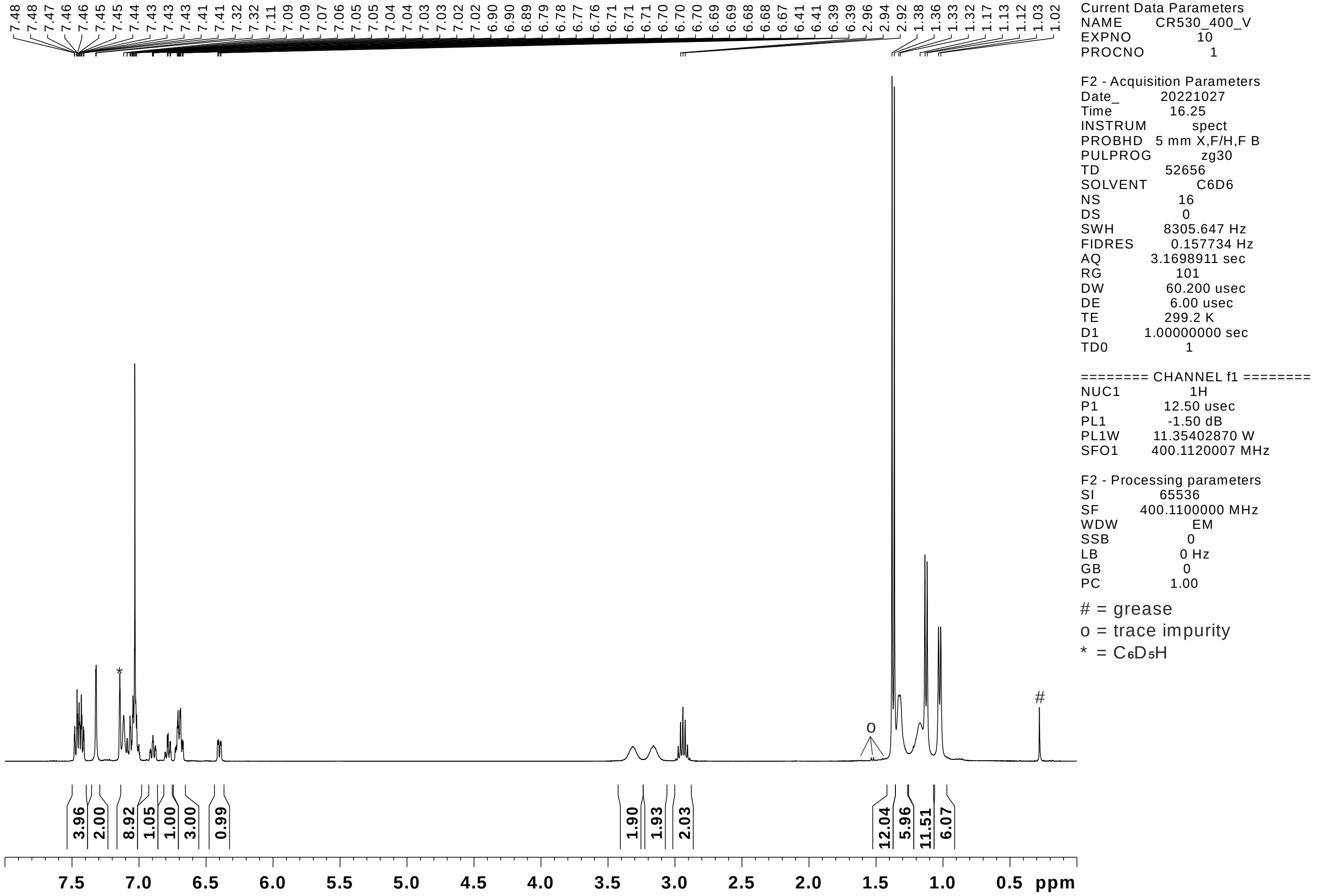

Figure SI16. ^{1}H NMR spectrum of compound **5**.

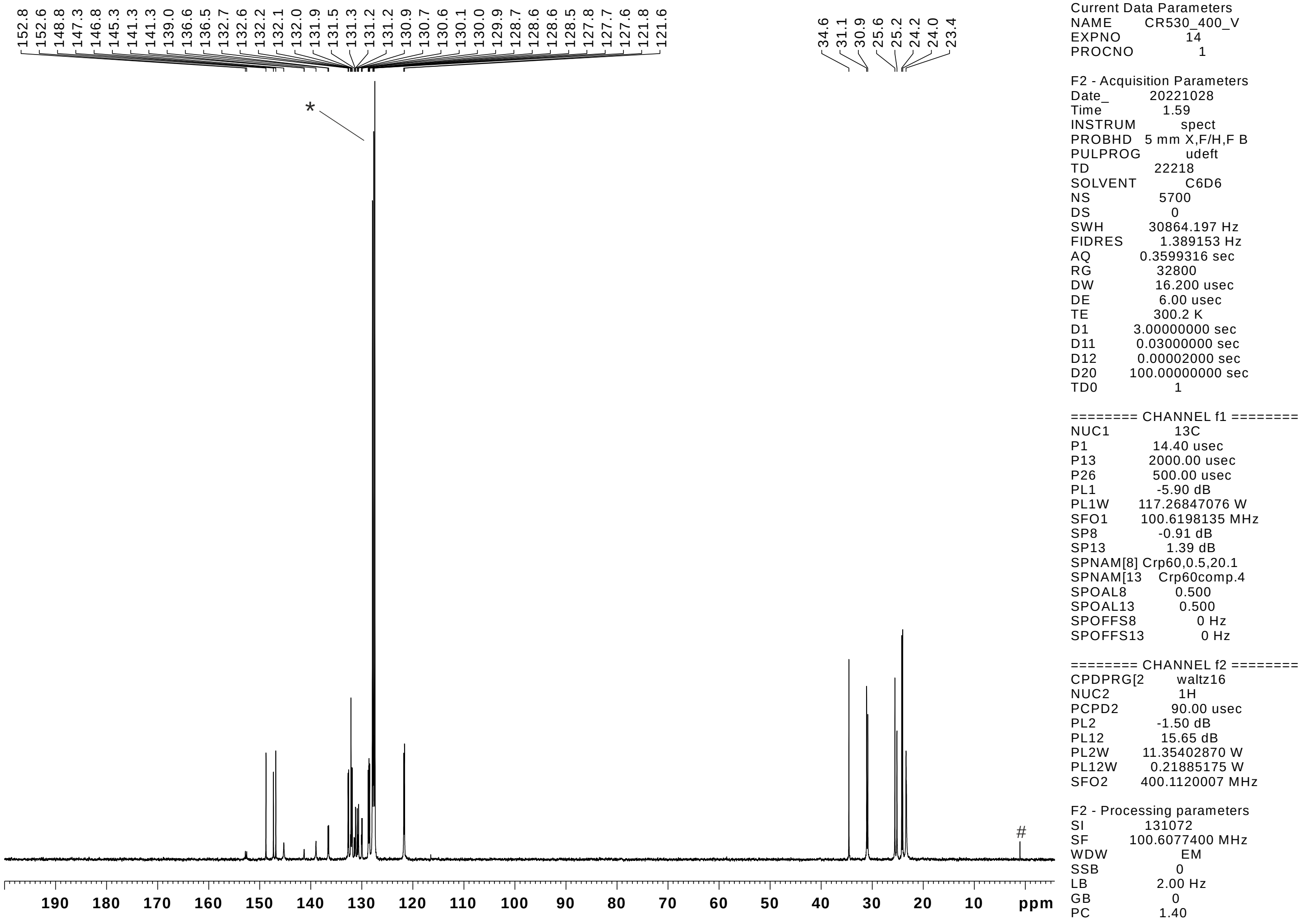

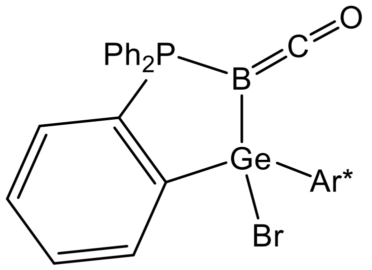

# = grease

* = C_6D_6

Figure SI17. $^{13}C\{^1H\}$ NMR spectrum of compound **5**.

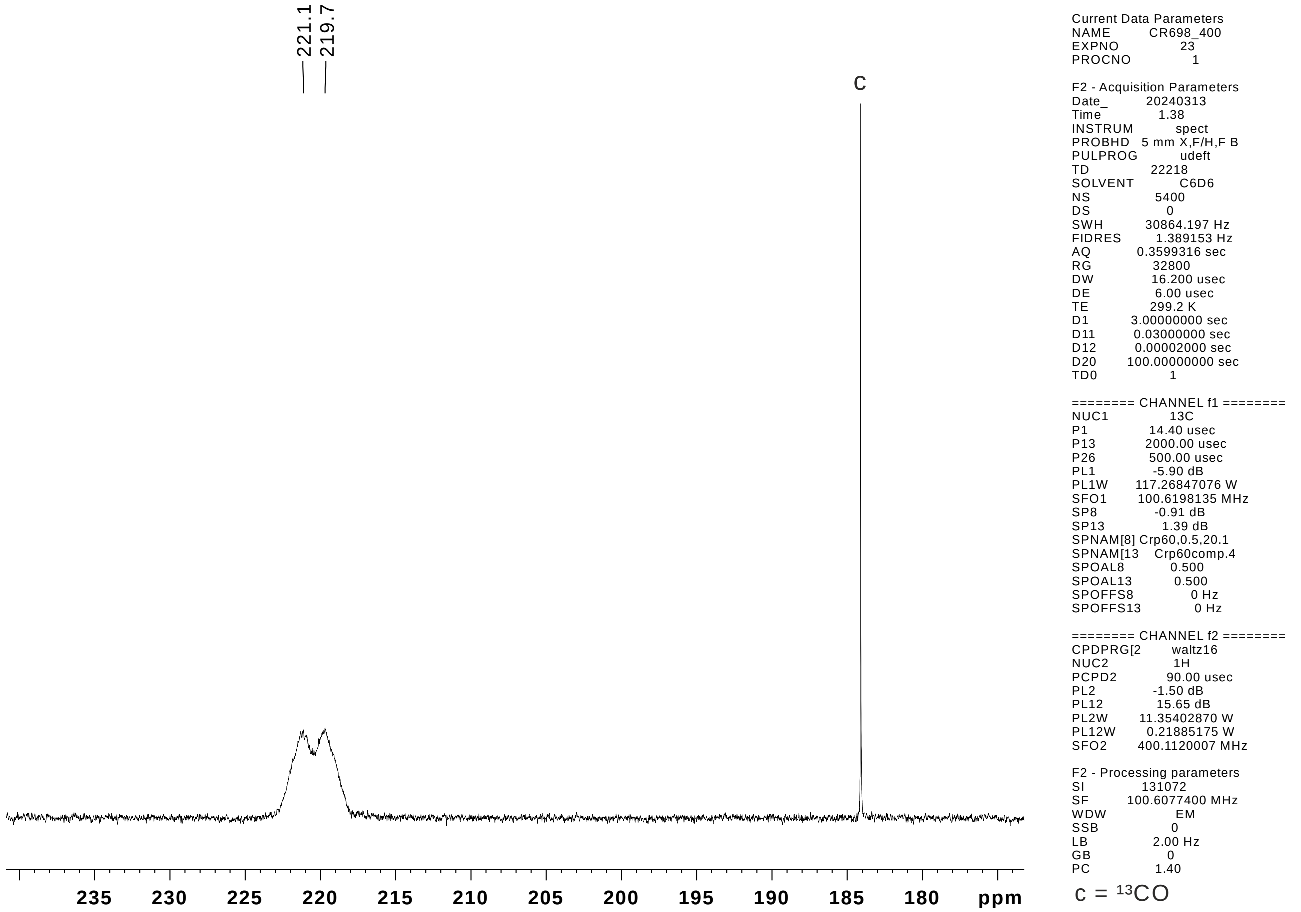

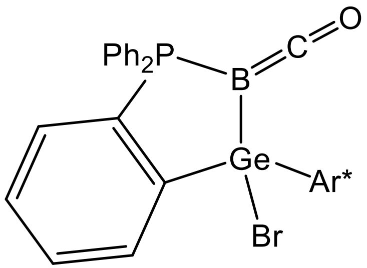

Figure SI18. $^{13}C\{^{1}H\}$ NMR spectrum of compound **5** synthesized with (99.5%) ^{13}CO.

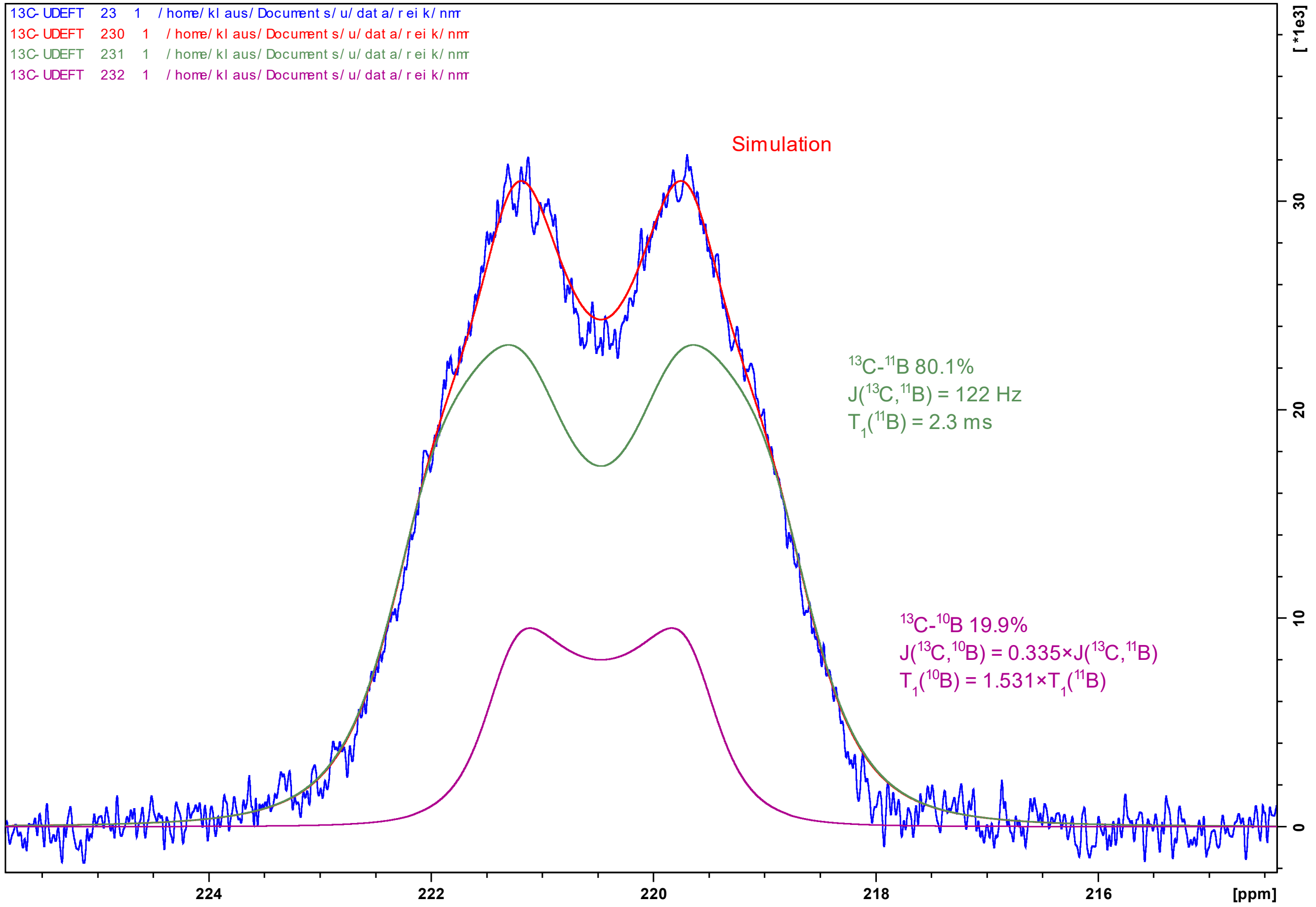

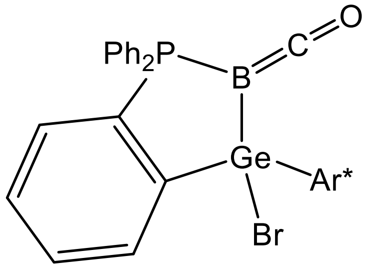

Figure SI19. Simulation of $^{13}C\{^{1}H\}$ NMR signal at 221 ppm using the parameters given in the figure for the two isotopologues, ^{13}C-^{11}B and ^{13}C-^{10}B, in the software WSolids.[9]

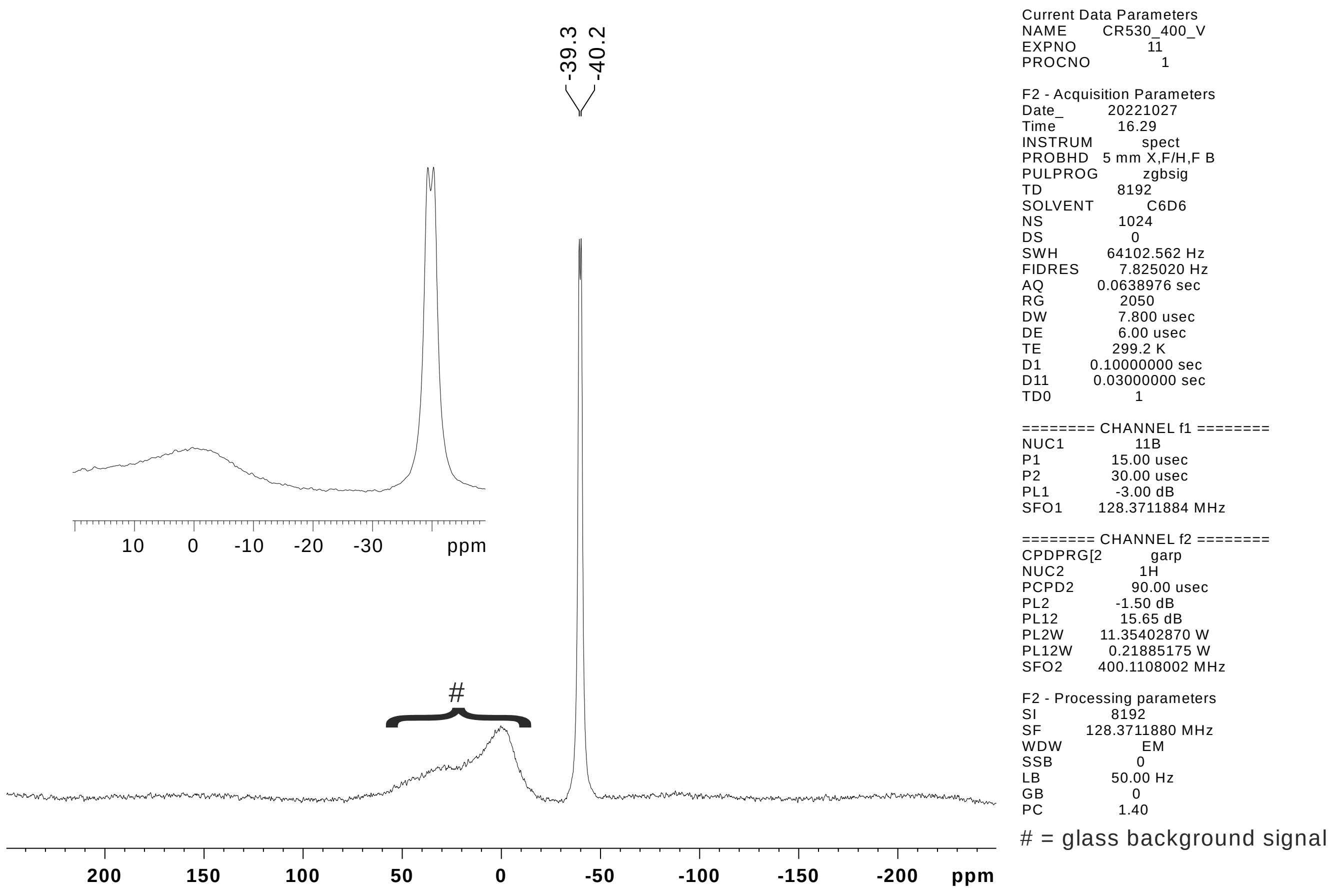

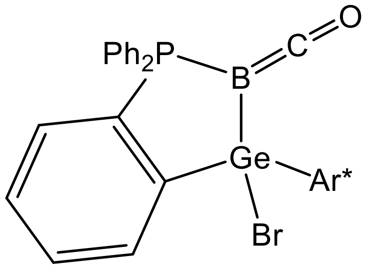

Figure SI20. $^{11}B\{^{1}H\}$ NMR spectrum of compound **5**.

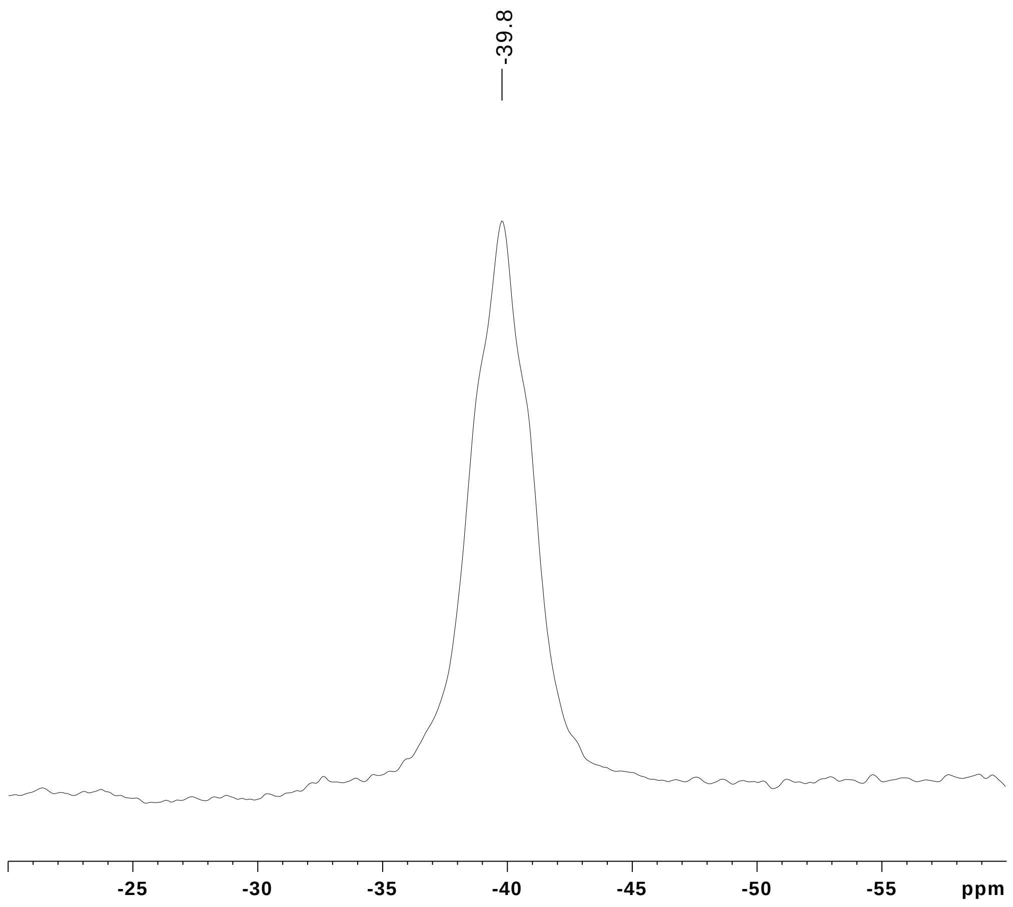

Current Data Parameters
NAME CR698_400
EXPNO 21
PROCNO 1

F2 - Acquisition Parameters
Date_ 20240312
Time 19.06
INSTRUM spect
PROBHD 5 mm X,F/H,F B
PULPROG zgbsig
TD 8192
SOLVENT C6D6
NS 1024
DS 0
SWH 64102.562 Hz
FIDRES 7.825020 Hz
AQ 0.0638976 sec
RG 2050
DW 7.800 usec
DE 6.00 usec
TE 299.2 K
D1 0.10000000 sec
D11 0.03000000 sec
TD0 1

======== CHANNEL f1 ========
NUC1 11B
P1 15.00 usec
P2 30.00 usec
PL1 -3.00 dB
SFO1 128.3711884 MHz

======== CHANNEL f2 ========
CPDPRG[2 garp
NUC2 1H
PCPD2 90.00 usec
PL2 -1.50 dB
PL12 15.65 dB
PL2W 11.35402870 W
PL12W 0.21885175 W
SFO2 400.1108002 MHz

F2 - Processing parameters
SI 8192
SF 128.3711880 MHz
WDW EM
SSB 0
LB 50.00 Hz
GB 0
PC 1.40

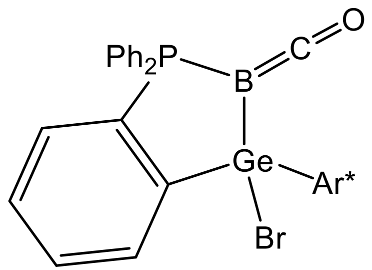

Figure SI21. $^{11}B\{^{1}H\}$ NMR spectrum of compound **5** synthesized with (> 99%) ^{13}CO.

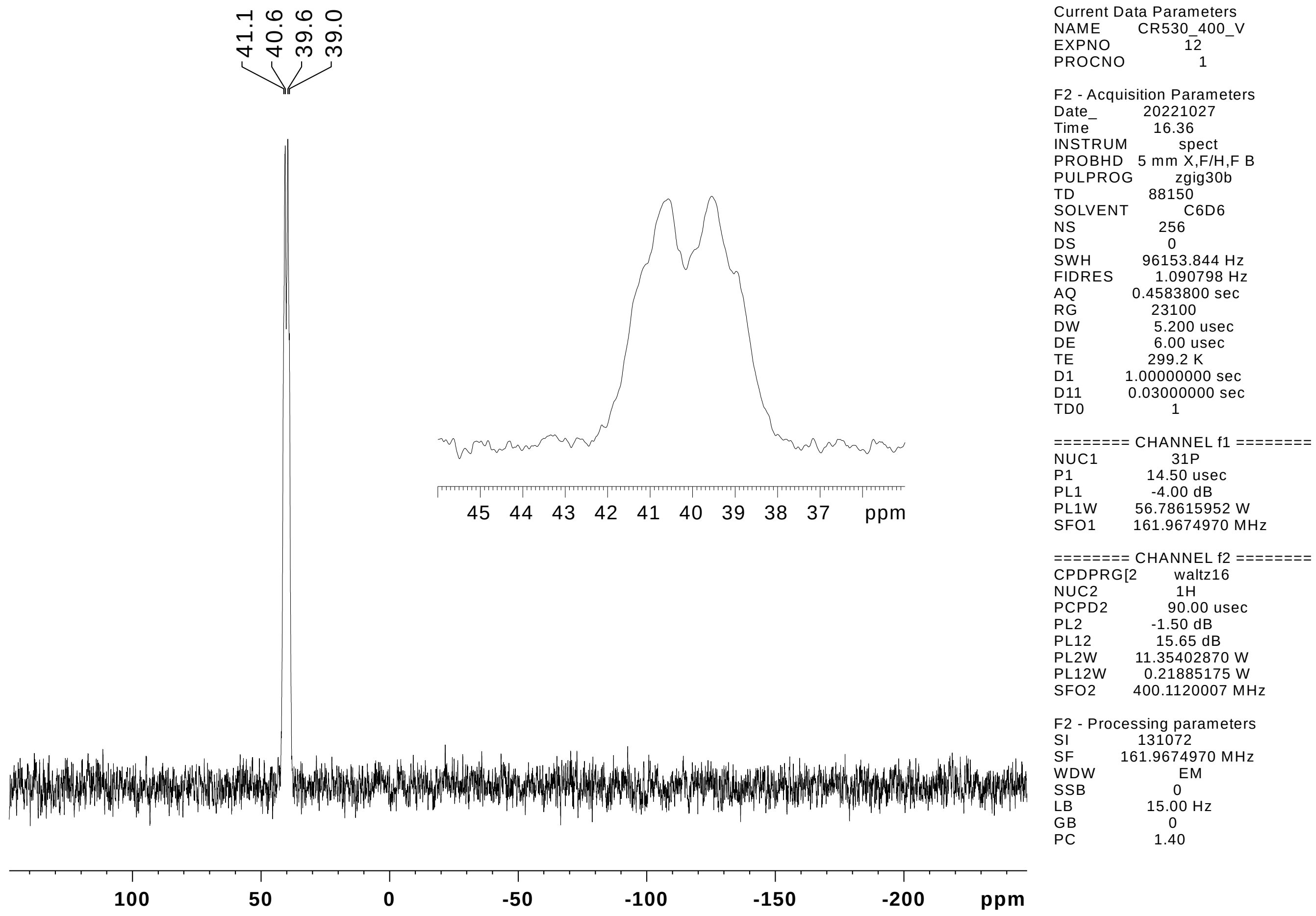

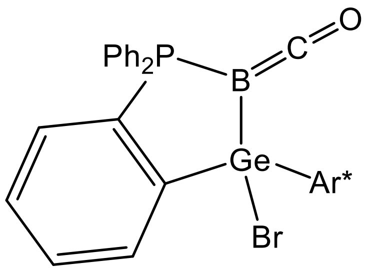

Figure SI22. $^{31}P\{^{1}H\}$ NMR spectrum of compound **5**.

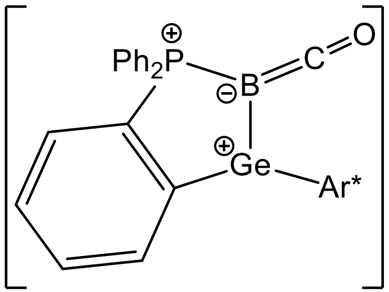

NMR spectra of compound **6**.

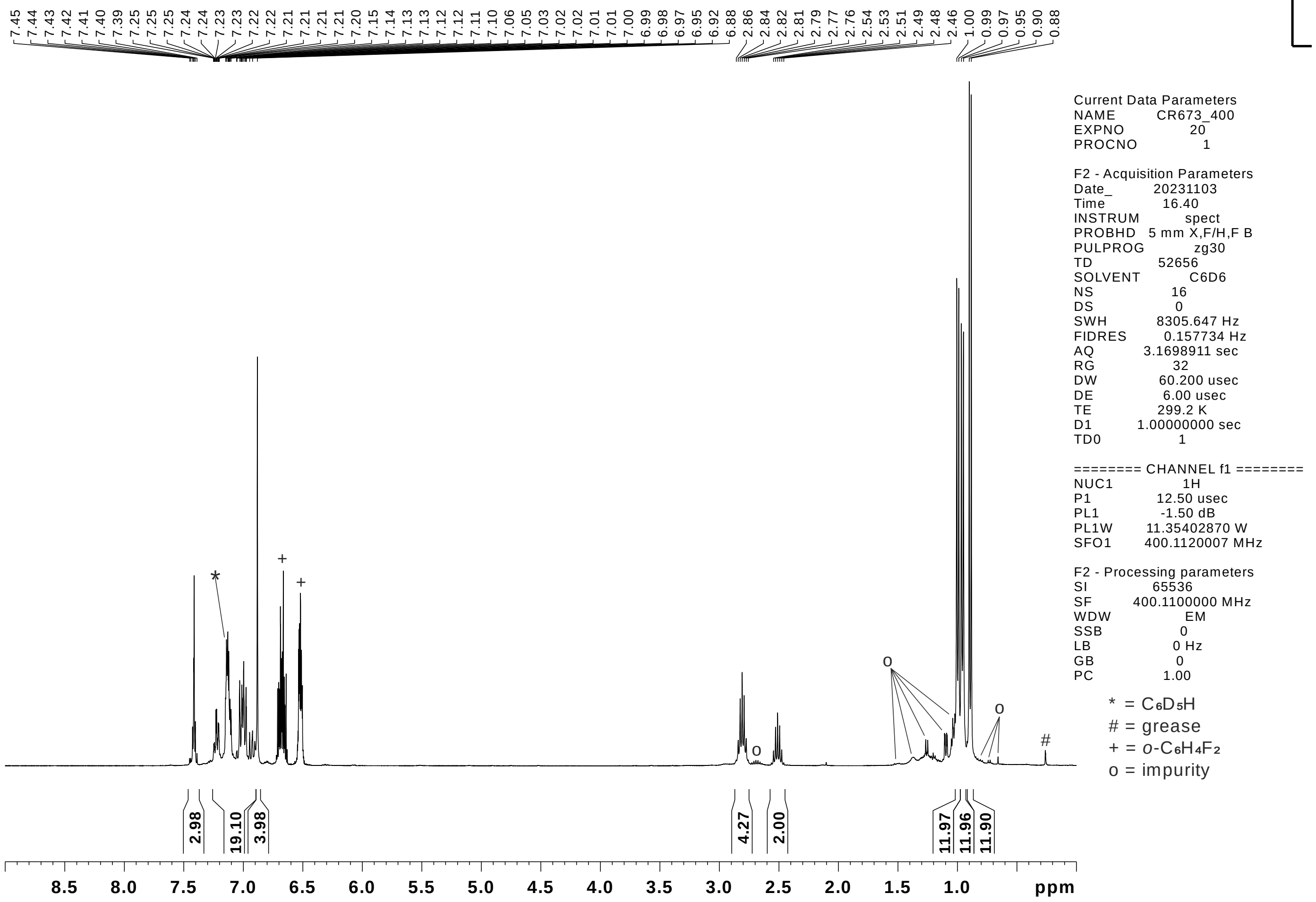

Figure SI23. ^{1}H NMR spectrum of compound **6**.

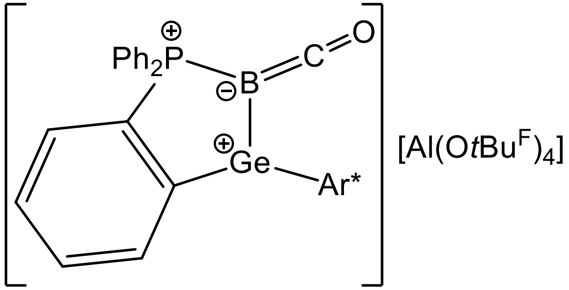

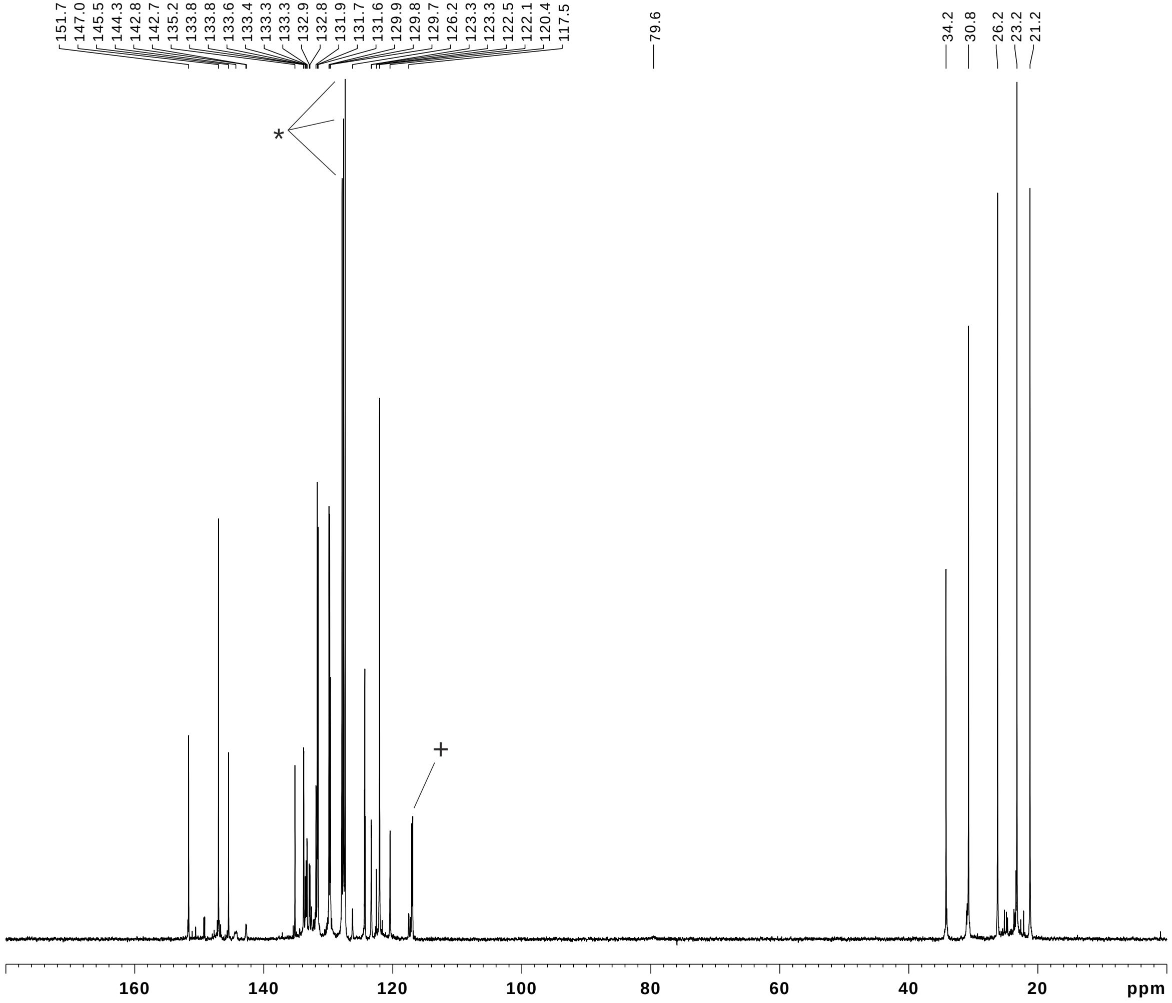

Current Data Parameters
NAME CR673_400
EXPNO 24
PROCNO 1

F2 - Acquisition Parameters
Date_ 20231104
Time 1.57
INSTRUM spect
PROBHD 5 mm X,F/H,F B
PULPROG udeft
TD 22218
SOLVENT C6D6
NS 5700
DS 0
SWH 30864.197 Hz
FIDRES 1.389153 Hz
AQ 0.3599316 sec
RG 32800
DW 16.200 usec
DE 6.00 usec
TE 299.2 K
D1 3.00000000 sec
D11 0.03000000 sec
D12 0.00002000 sec
D20 100.00000000 sec
TD0 1

======== CHANNEL f1 ========
NUC1 13C
P1 14.40 usec
P13 2000.00 usec
P26 500.00 usec
PL1 -5.90 dB
PL1W 117.26847076 W
SFO1 100.6198135 MHz
SP8 -0.91 dB
SP13 1.39 dB
SPNAM[8] Crp60,0.5,20.1
SPNAM[13 Crp60comp.4
SPOAL8 0.500
SPOAL13 0.500
SPOFFS8 0 Hz
SPOFFS13 0 Hz

======== CHANNEL f2 ========
CPDPRG[2 waltz16
NUC2 1H
PCPD2 90.00 usec
PL2 -1.50 dB
PL12 15.65 dB
PL2W 11.35402870 W
PL12W 0.21885175 W
SFO2 400.1120007 MHz

F2 - Processing parameters
SI 131072
SF 100.6077400 MHz
WDW EM
SSB 0
LB 2.00 Hz
GB 0
PC 1.40

+ = *o*-$C_6H_4F_2$

* = C_6D_6

Figure SI24. $^{13}C\{^1H\}$ NMR spectrum of compound **6**.

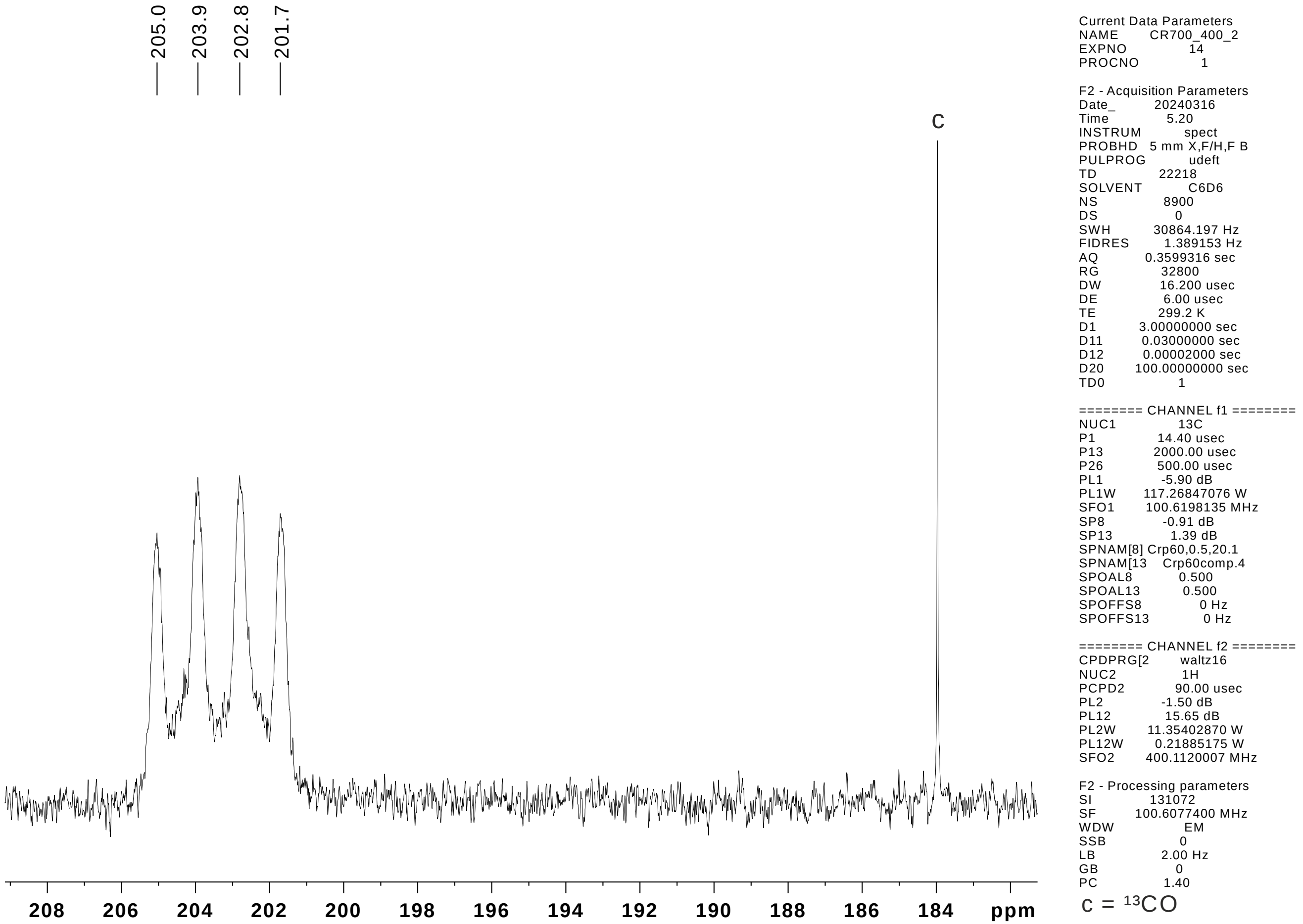

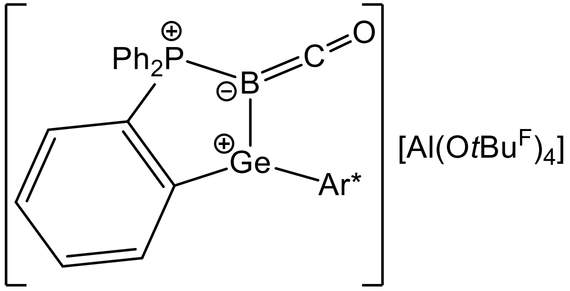

Figure SI25. $^{13}C\{^{1}H\}$ NMR spectrum of compound **6** synthesized with (> 99%) ^{13}CO.

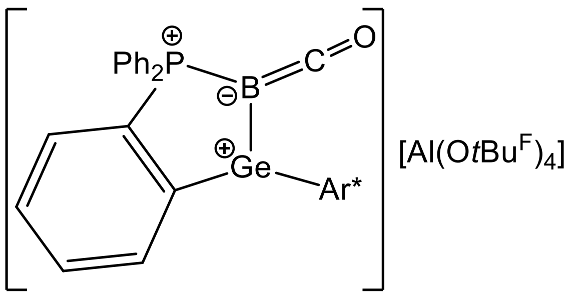

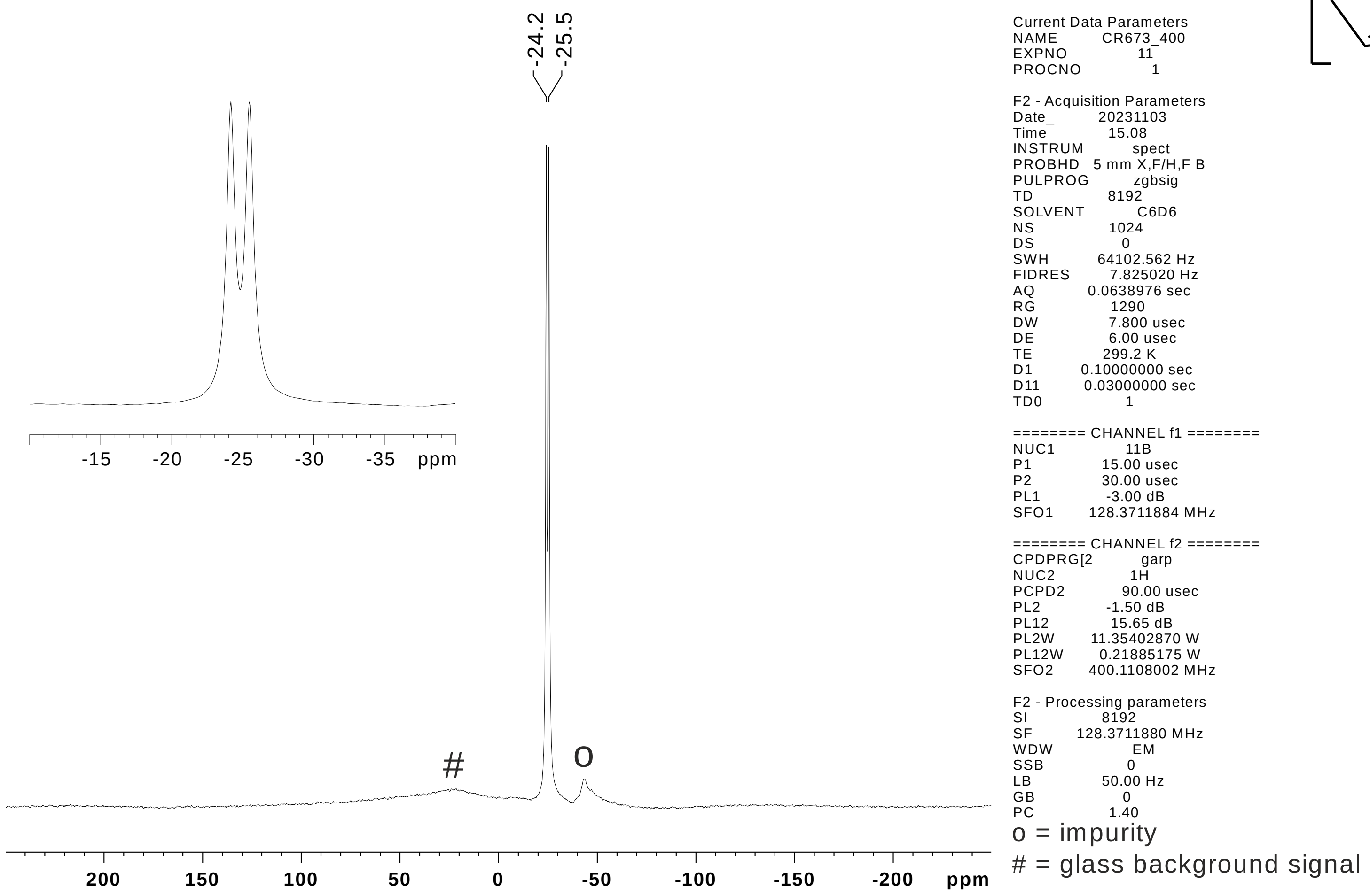

Figure SI26. $^{11}B\{^1H\}$ NMR spectrum of compound **6**.

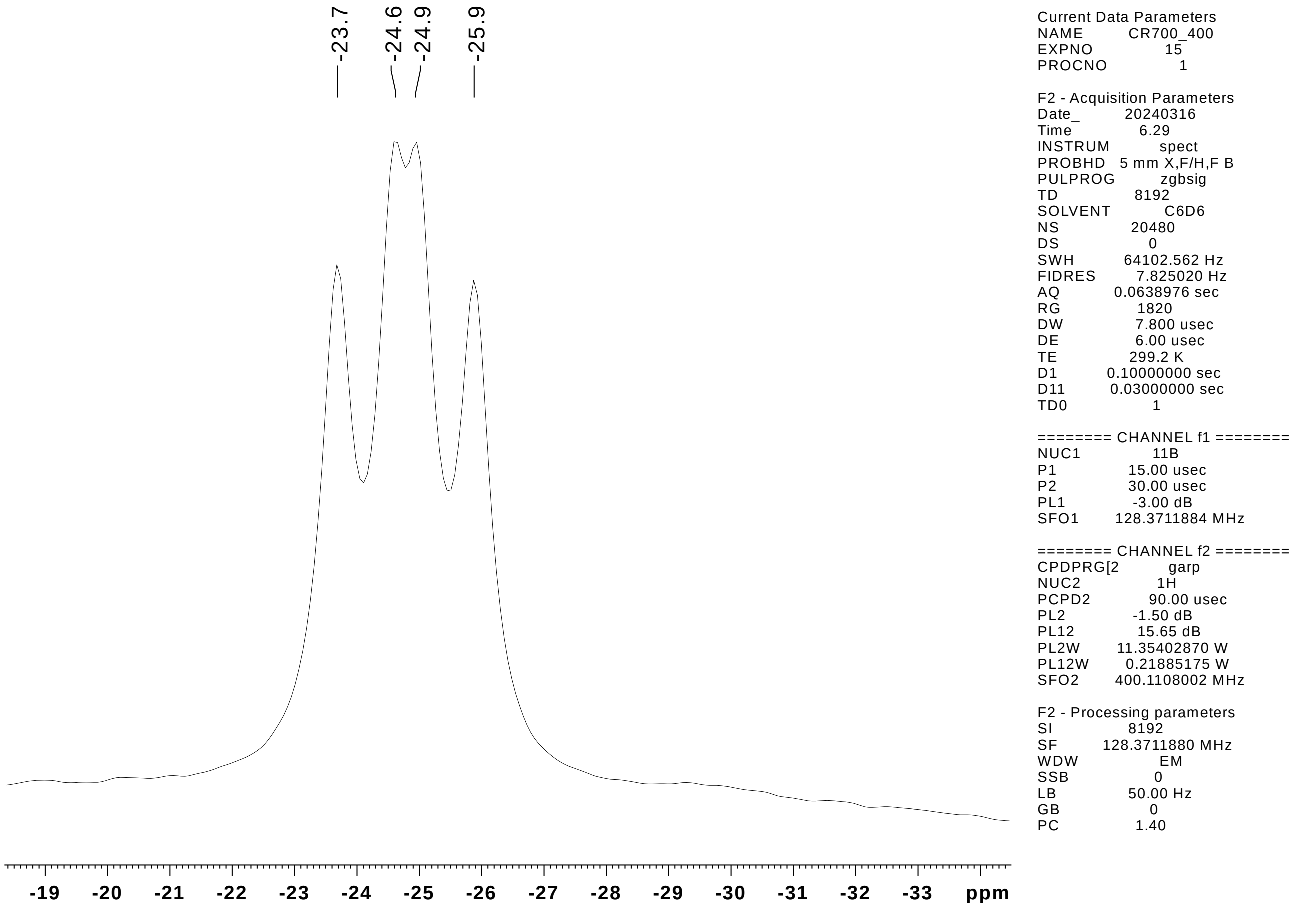

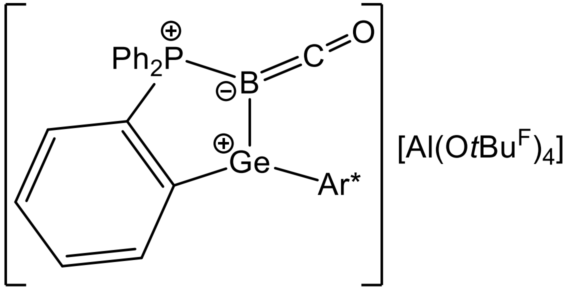

Figure SI27. $^{11}B\{^1H\}$ NMR spectrum of compound **6** synthesized with (> 99%) ^{13}CO.

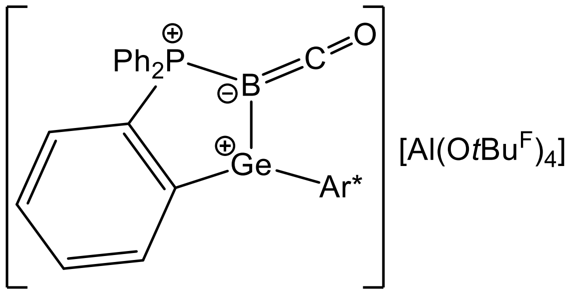

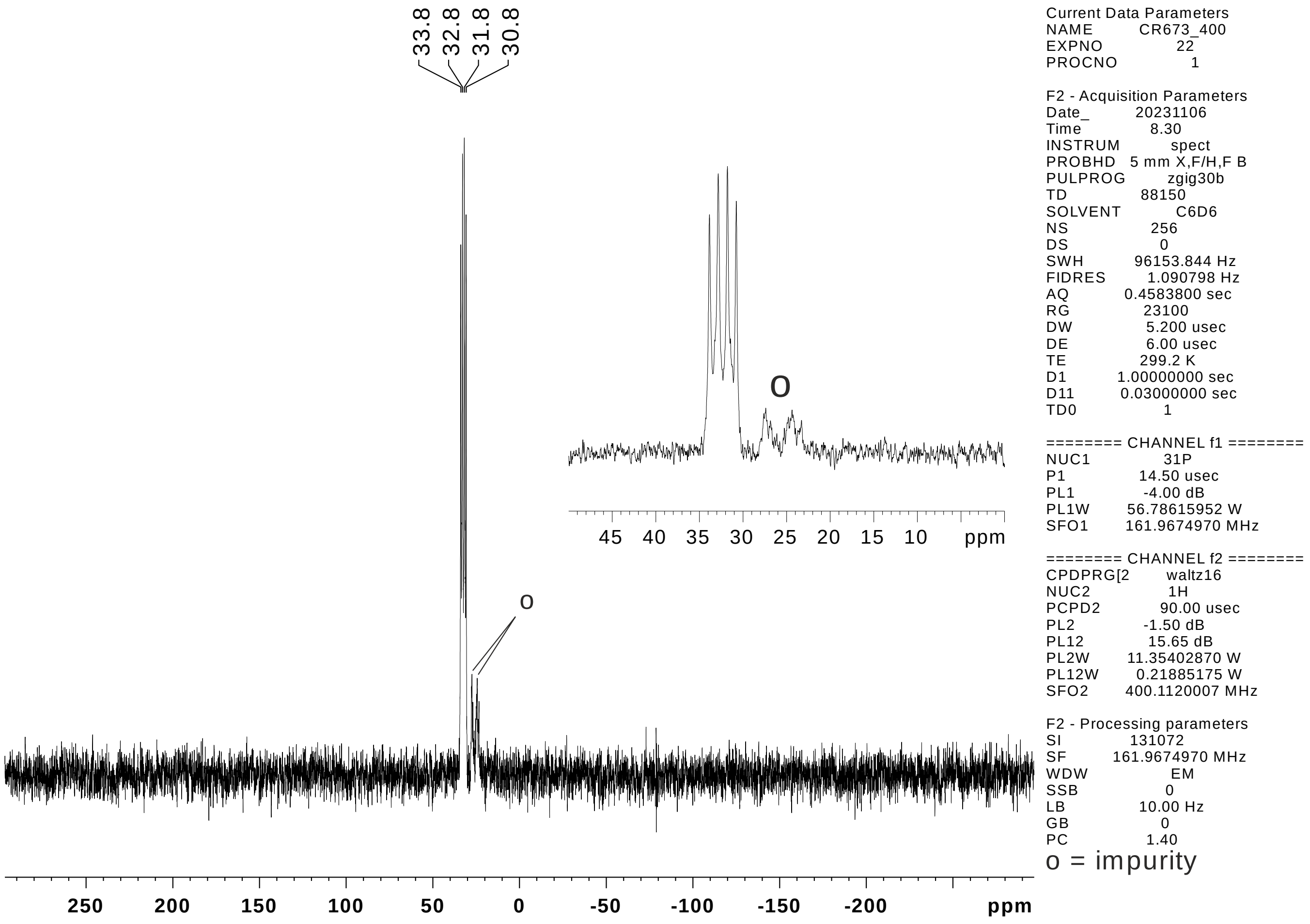

Figure SI28. $^{31}P\{^{1}H\}$ NMR spectrum of compound **6**.

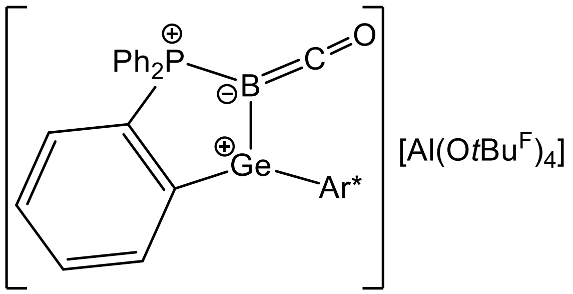

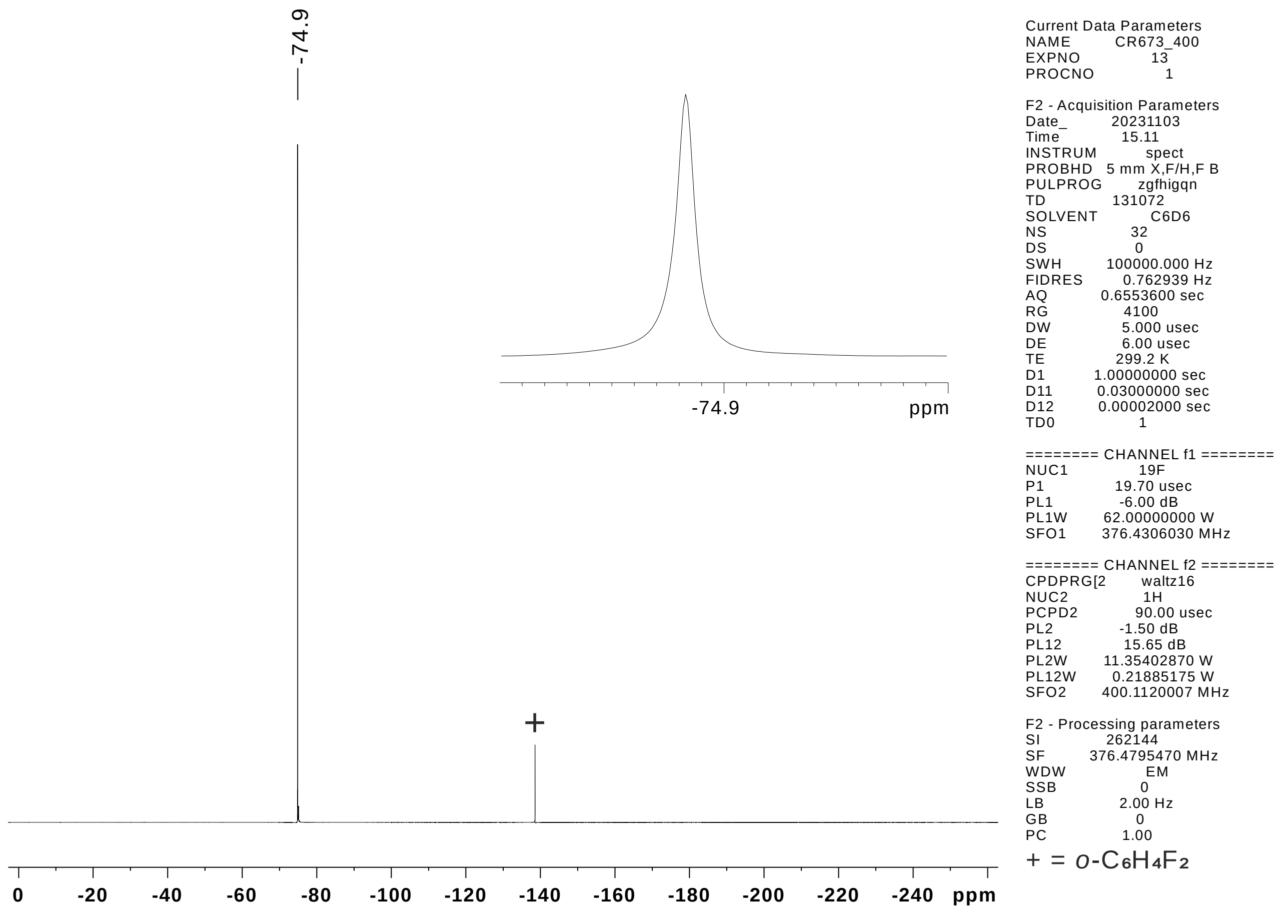

Figure SI29. $^{19}F\{^{1}H\}$ NMR spectrum of compound **6**.

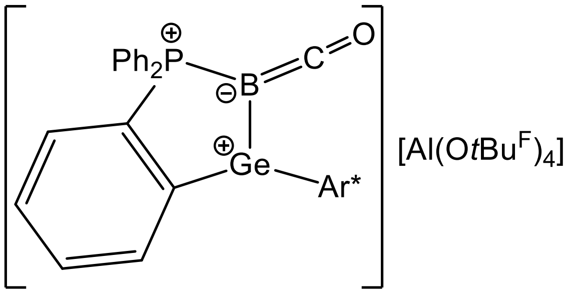

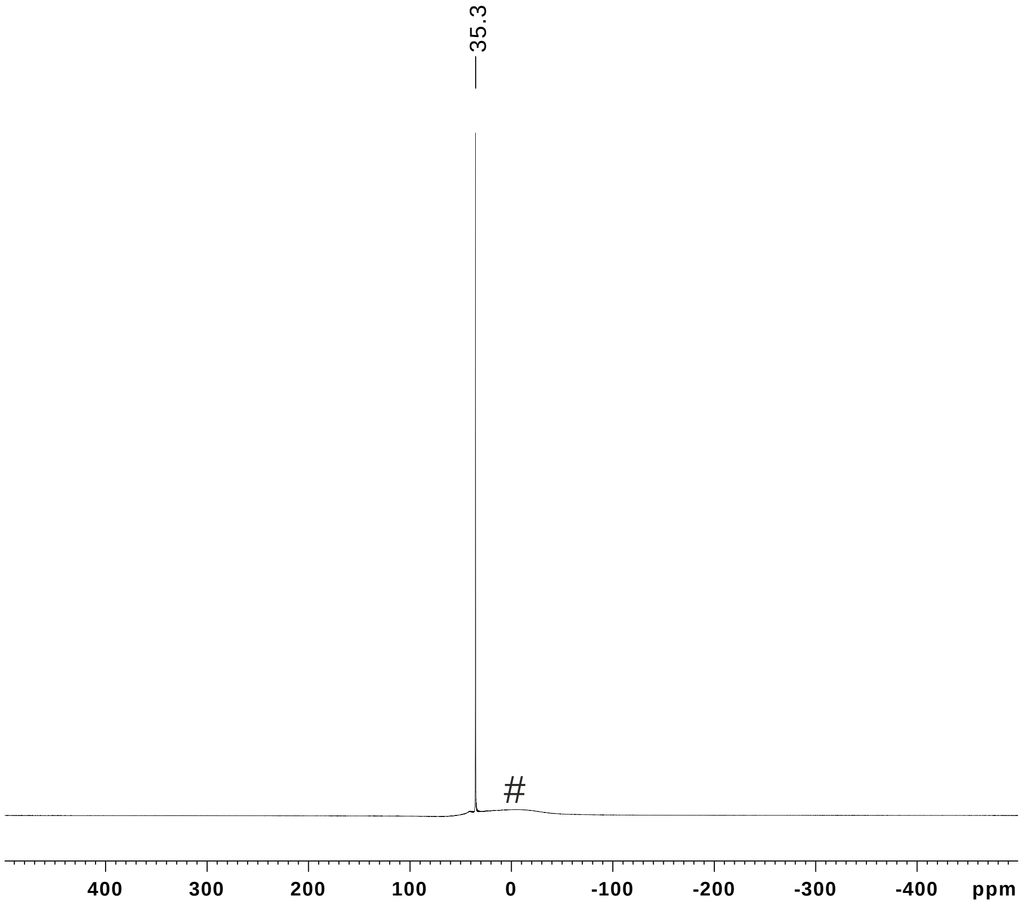

Current Data Parameters
NAME CR673_400
EXPNO 15
PROCNO 1

F2 - Acquisition Parameters
Date_ 20231103
Time 15.19
INSTRUM spect
PROBHD 5 mm X,F/H,F B
PULPROG zgbsig
TD 16384
SOLVENT C6D6
NS 2000
DS 0
SWH 104166.664 Hz
FIDRES 6.357829 Hz
AQ 0.0786432 sec
RG 575
DW 4.800 usec
DE 6.00 usec
TE 299.2 K
D1 0.10000000 sec
D11 0.03000000 sec
TD0 1

======== CHANNEL f1 ========
NUC1 27Al
P1 11.13 usec
P2 22.26 usec
PL1 -3.00 dB
SFO1 104.2560990 MHz

======== CHANNEL f2 ========
CPDPRG[2 garp
NUC2 1H
PCPD2 90.00 usec
PL2 -1.50 dB
PL12 15.65 dB
PL2W 11.35402870 W
PL12W 0.21885175 W
SFO2 400.1108002 MHz

F2 - Processing parameters
SI 16384
SF 104.2560990 MHz
WDW EM
SSB 0
LB 10.00 Hz
GB 0
PC 1.40

# = background signal

Figure SI30. ^{27}Al NMR spectrum of compound **6**.

NMR spectra of compound **7**.

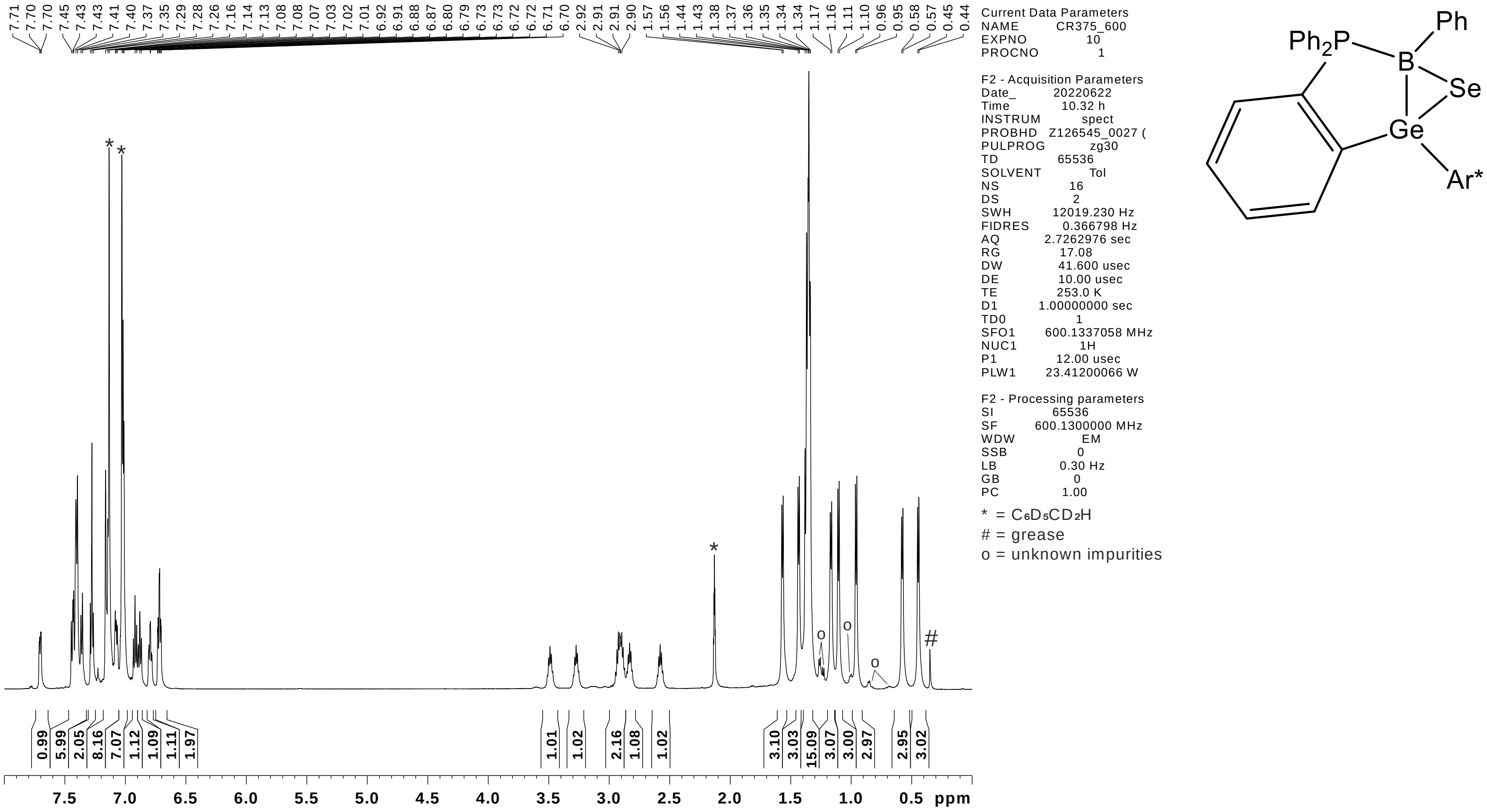

Figure SI31. ^{1}H NMR spectrum of compound **7**.

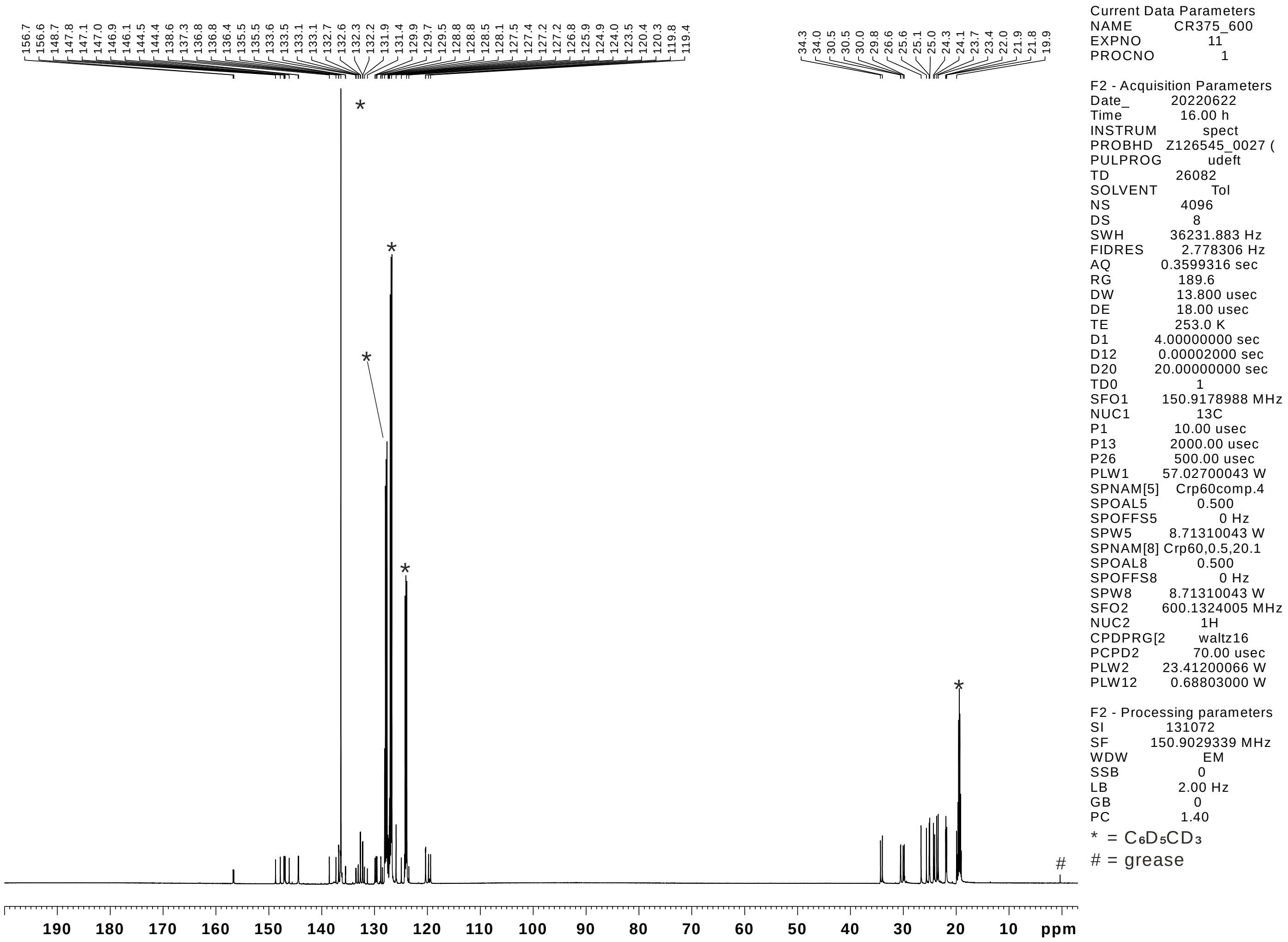

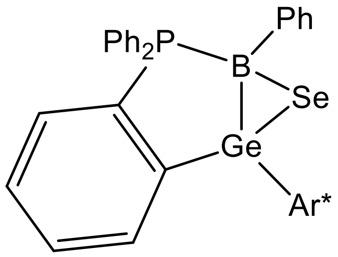

Figure SI32. $^{13}C\{^{1}H\}$ NMR spectrum of compound **7**.

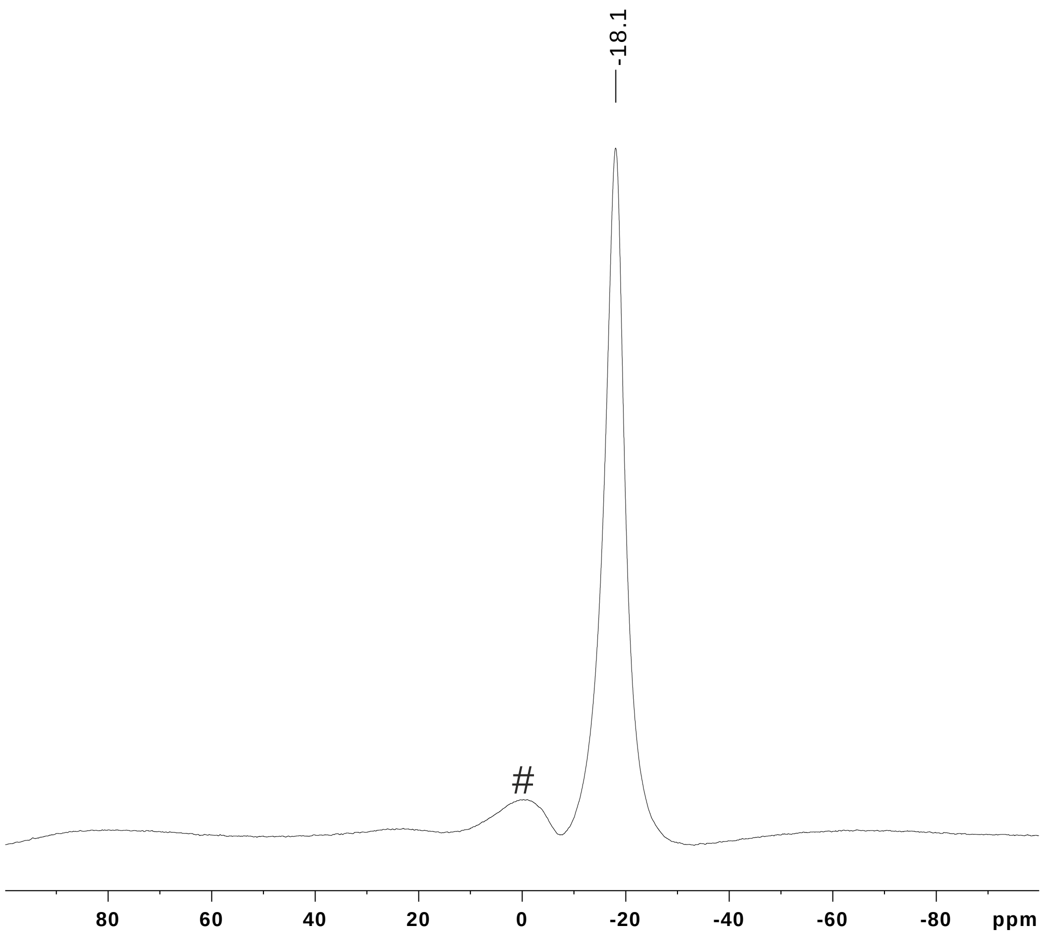

Current Data Parameters
NAME CR375_600
EXPNO 14
PROCNO 1

F2 - Acquisition Parameters
Date_ 20220622
Time 17.28 h
INSTRUM spect
PROBHD Z126545_0027 (
PULPROG zgbsig
TD 16384
SOLVENT Tol
NS 4096
DS 4
SWH 38461.539 Hz
FIDRES 4.695012 Hz
AQ 0.2129920 sec
RG 189.6
DW 13.000 usec
DE 18.00 usec
TE 253.0 K
D1 0.10000000 sec
D11 0.03000000 sec
TD0 1
SFO1 192.5455530 MHz
NUC1 11B
P1 19.80 usec
P2 39.60 usec
PLW1 50.00000000 W
SFO2 600.1328206 MHz
NUC2 1H
CPDPRG[2 waltz16
PCPD2 70.00 usec
PLW2 23.41200066 W
PLW12 0.68803000 W

F2 - Processing parameters
SI 16384
SF 192.5455530 MHz
WDW EM
SSB 0
LB 50.00 Hz
GB 0
PC 1.40

# = glass background signal

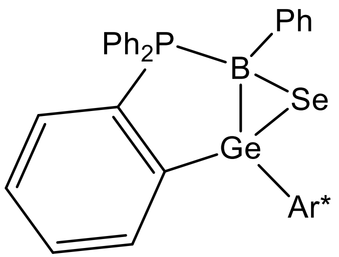

Figure SI33. $^{11}B\{^{1}H\}$ NMR spectrum of compound **7**.

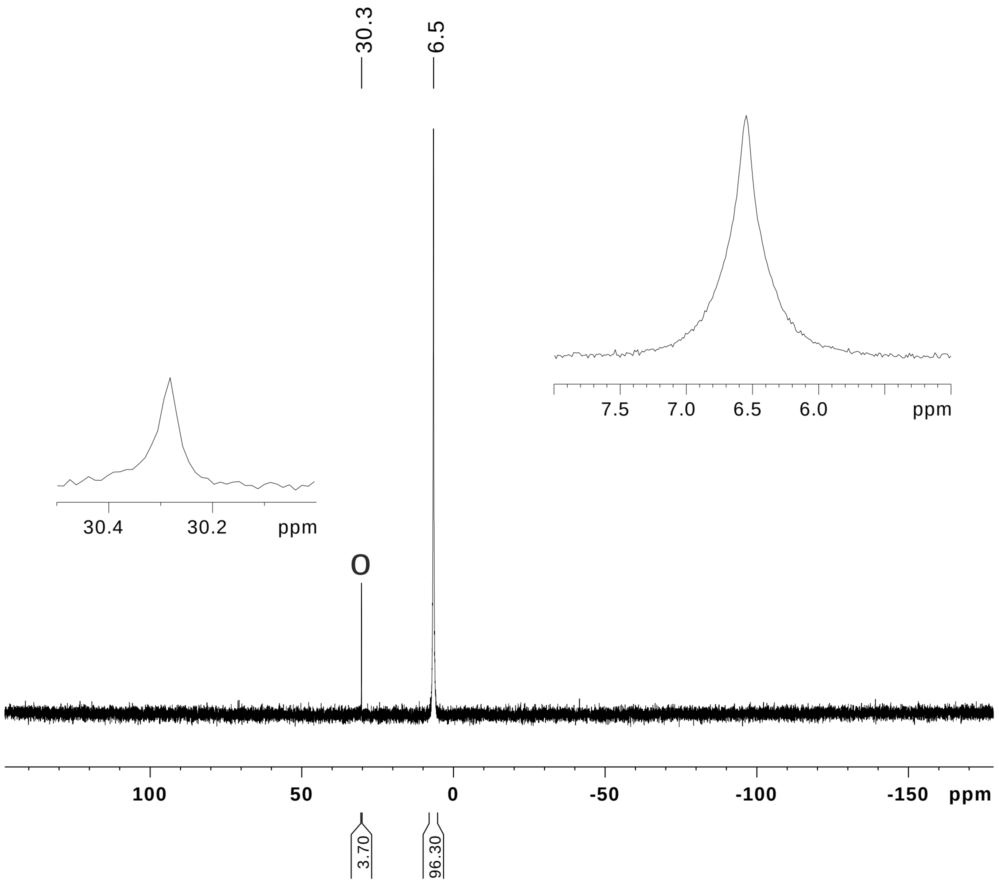

Current Data Parameters
NAME CR375_600
EXPNO 13
PROCNO 1

F2 - Acquisition Parameters
Date_ 20220622
Time 17.02 h
INSTRUM spect
PROBHD Z126545_0027 (
PULPROG zgpg30
TD 65536
SOLVENT Tol
NS 16
DS 4
SWH 96153.844 Hz
FIDRES 2.934382 Hz
AQ 0.3407872 sec
RG 189.6
DW 5.200 usec
DE 18.00 usec
TE 253.0 K
D1 2.00000000 sec
D11 0.03000000 sec
TD0 1
SFO1 242.9249301 MHz
NUC1 31P
P1 12.00 usec
PLW1 52.43000031 W
SFO2 600.1324005 MHz
NUC2 1H
CPDPRG[2 waltz16
PCPD2 70.00 usec
PLW2 23.41200066 W
PLW12 0.68803000 W
PLW13 0.34606999 W

F2 - Processing parameters
SI 32768
SF 242.9370770 MHz
WDW EM
SSB 0
LB 1.00 Hz
GB 0
PC 1.40

o = unknown impurity

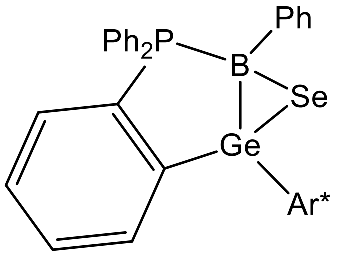

Figure SI34. $^{31}P\{^{1}H\}$ NMR spectrum of compound **7**.

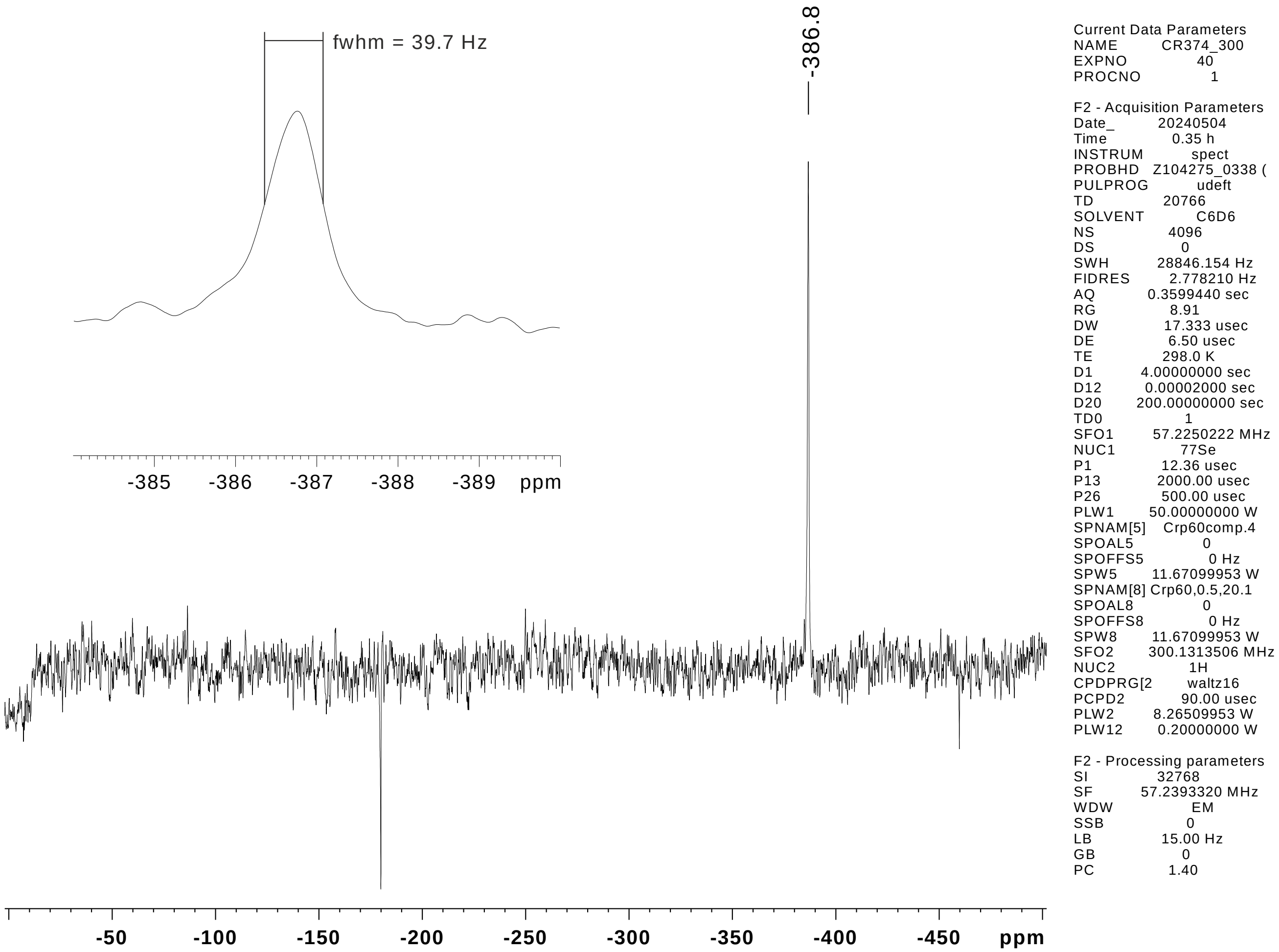

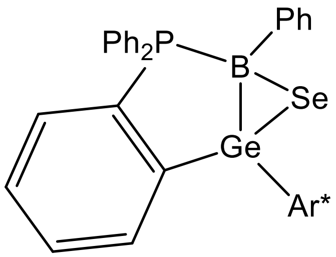

Figure SI35. $^{77}Se\{^{1}H\}$ NMR spectrum of compound **7**.

NMR spectra of compound **8**.

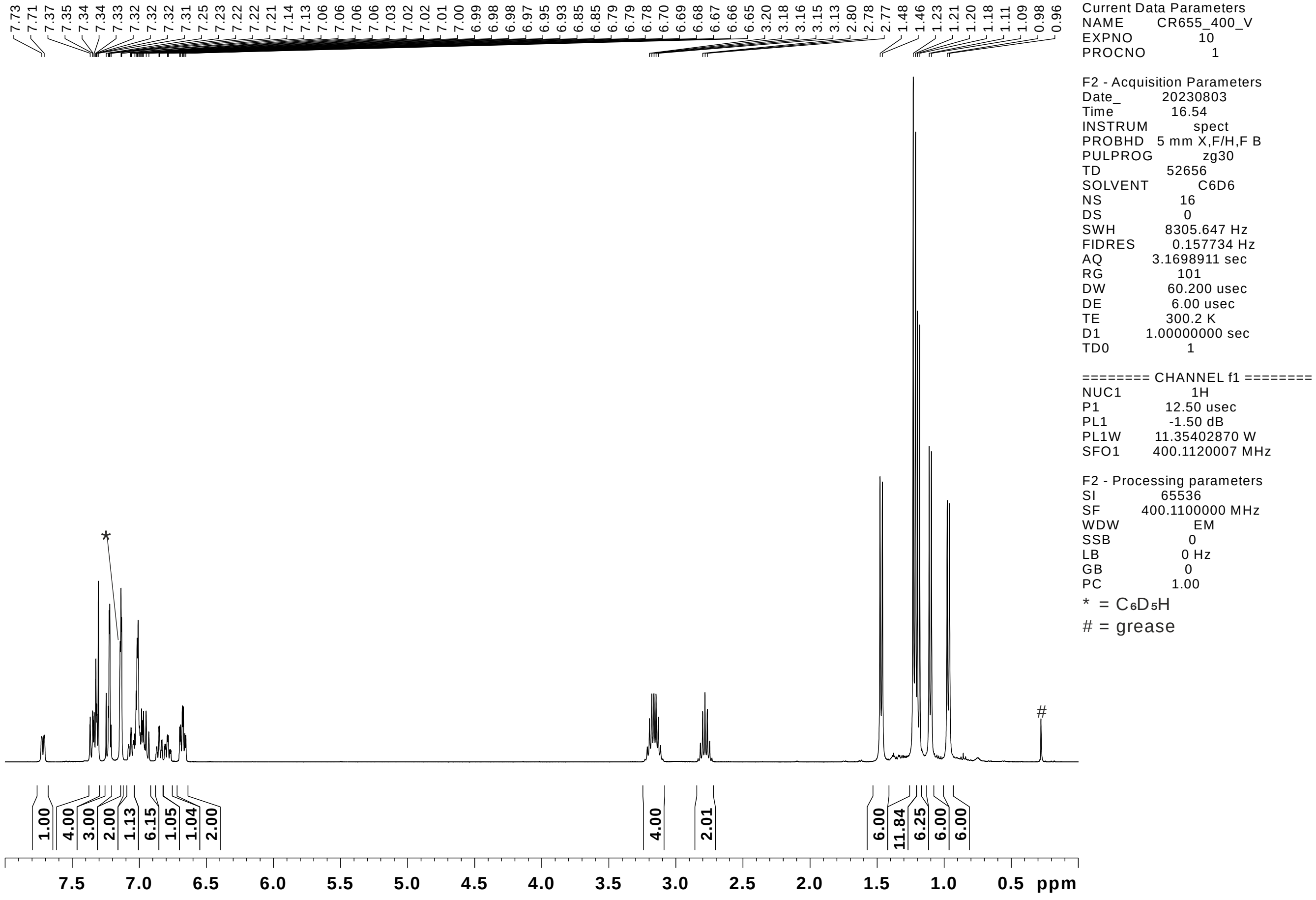

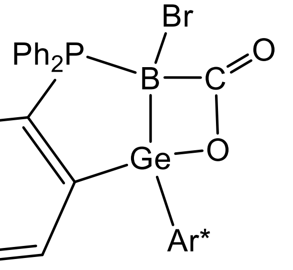

Figure SI36. ^{1}H NMR spectrum of compound **8**.

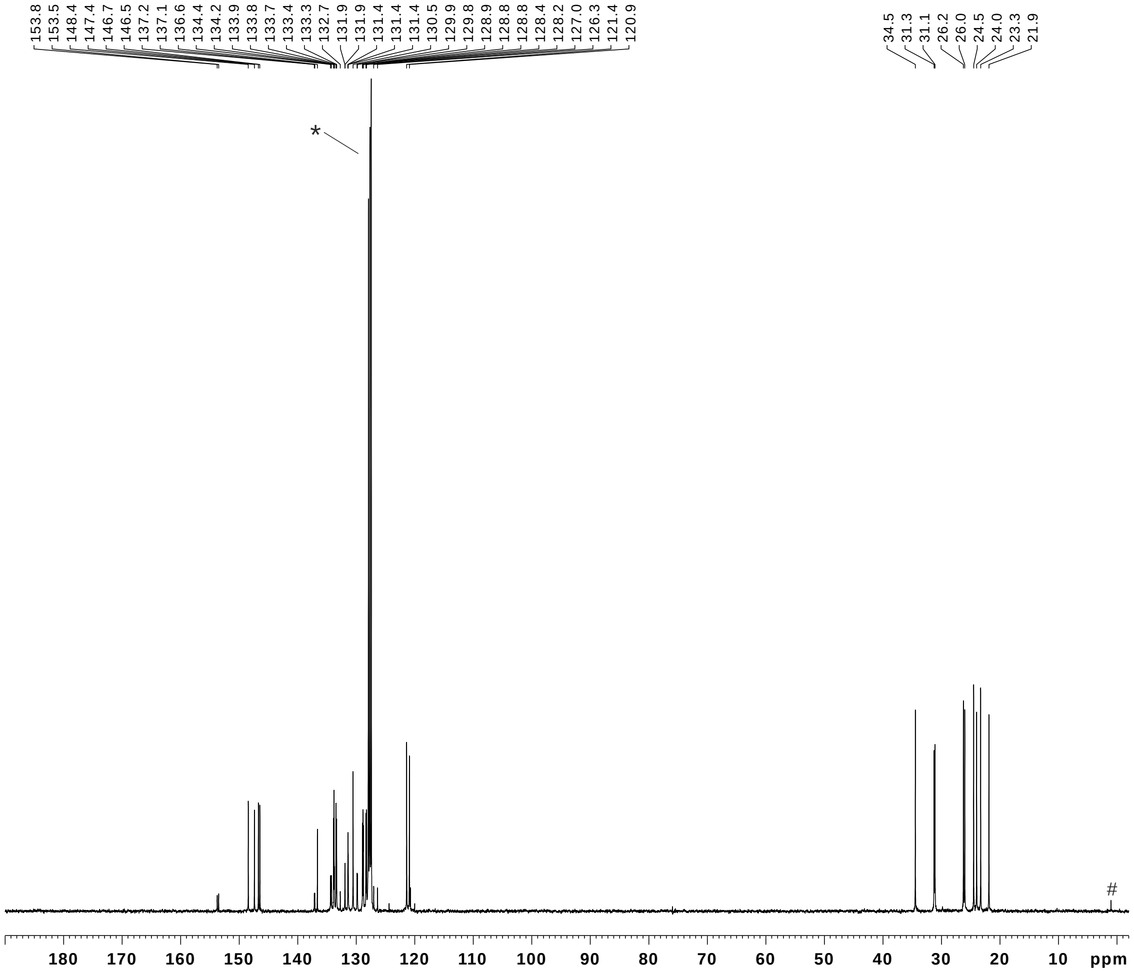

Current Data Parameters
NAME CR655_400_V
EXPNO 12
PROCNO 1

F2 - Acquisition Parameters
Date_ 20230804
Time 2.01
INSTRUM spect
PROBHD 5 mm X,F/H,F B
PULPROG udeft
TD 22218
SOLVENT C6D6
NS 5700
DS 0
SWH 30864.197 Hz
FIDRES 1.389153 Hz
AQ 0.3599316 sec
RG 32800
DW 16.200 usec
DE 6.00 usec
TE 300.2 K
D1 3.00000000 sec
D11 0.03000000 sec
D12 0.00002000 sec
D20 100.00000000 sec
TD0 1

======== CHANNEL f1 ========
NUC1 13C
P1 14.40 usec
P13 2000.00 usec
P26 500.00 usec
PL1 -5.90 dB
PL1W 117.26847076 W
SFO1 100.6198135 MHz
SP8 -0.91 dB
SP13 1.39 dB
SPNAM[8] Crp60,0.5,20.1
SPNAM[13 Crp60comp.4
SPOAL8 0.500
SPOAL13 0.500
SPOFFS8 0 Hz
SPOFFS13 0 Hz

======== CHANNEL f2 ========
CPDPRG[2 waltz16
NUC2 1H
PCPD2 90.00 usec
PL2 -1.50 dB
PL12 15.65 dB
PL2W 11.35402870 W
PL12W 0.21885175 W
SFO2 400.1120007 MHz

F2 - Processing parameters
SI 131072
SF 100.6077400 MHz
WDW EM
SSB 0
LB 2.00 Hz
GB 0
PC 1.40

# = grease
* = C_6D_6

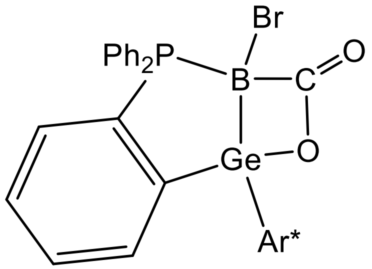

Figure SI37. $^{13}C\{^1H\}$ NMR spectrum of compound **8**.

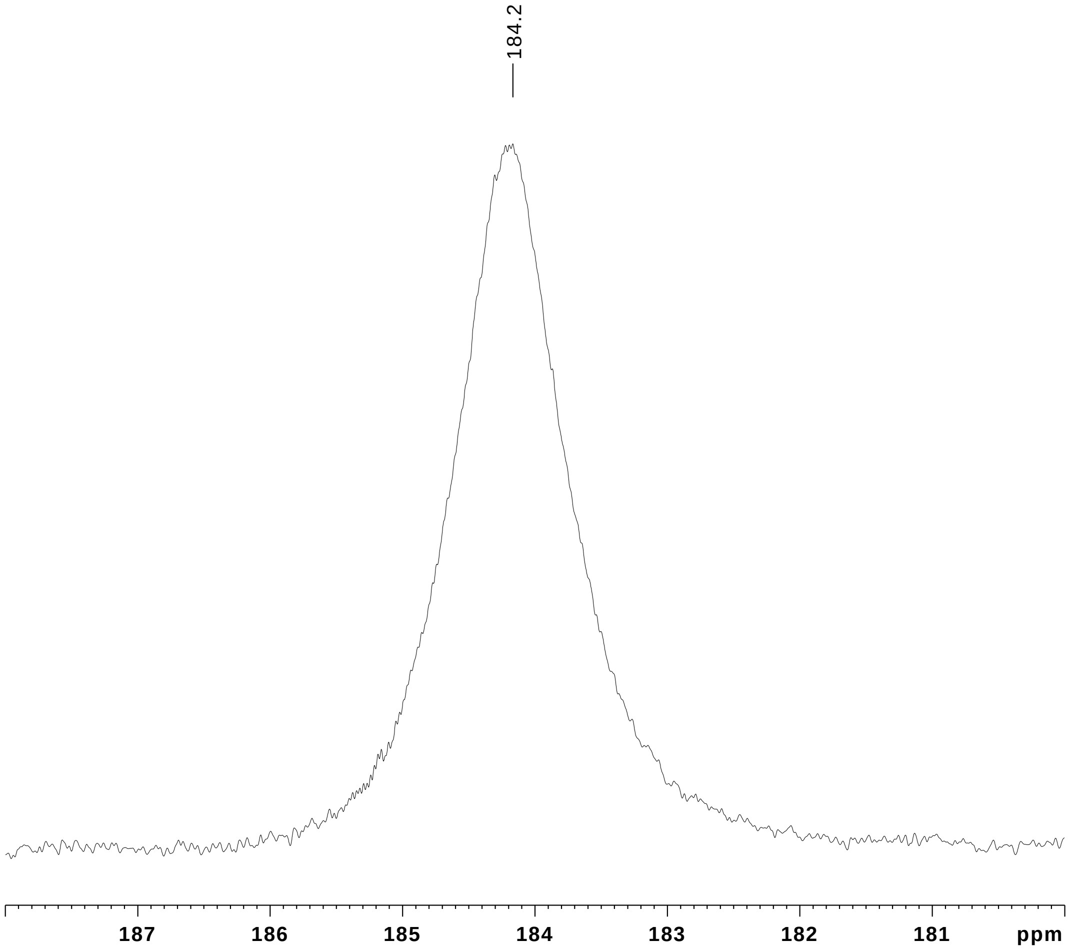

Current Data Parameters
NAME CR699_400_2
EXPNO 24
PROCNO 1

F2 - Acquisition Parameters
Date_ 20240313
Time 7.19
INSTRUM spect
PROBHD 5 mm X,F/H,F B
PULPROG udeft
TD 22218
SOLVENT C6D6
NS 5400
DS 0
SWH 30864.197 Hz
FIDRES 1.389153 Hz
AQ 0.3599316 sec
RG 32800
DW 16.200 usec
DE 6.00 usec
TE 299.2 K
D1 3.00000000 sec
D11 0.03000000 sec
D12 0.00002000 sec
D20 100.00000000 sec
TD0 1

======== CHANNEL f1 ========
NUC1 13C
P1 14.40 usec
P13 2000.00 usec
P26 500.00 usec
PL1 -5.90 dB
PL1W 117.26847076 W
SFO1 100.6198135 MHz
SP8 -0.91 dB
SP13 1.39 dB
SPNAM[8] Crp60,0.5,20.1
SPNAM[13 Crp60comp.4
SPOAL8 0.500
SPOAL13 0.500
SPOFFS8 0 Hz
SPOFFS13 0 Hz

======== CHANNEL f2 ========
CPDPRG[2 waltz16
NUC2 1H
PCPD2 90.00 usec
PL2 -1.50 dB
PL12 15.65 dB
PL2W 11.35402870 W
PL12W 0.21885175 W
SFO2 400.1120007 MHz

F2 - Processing parameters
SI 131072
SF 100.6077400 MHz
WDW EM
SSB 0
LB 2.00 Hz
GB 0
PC 1.40

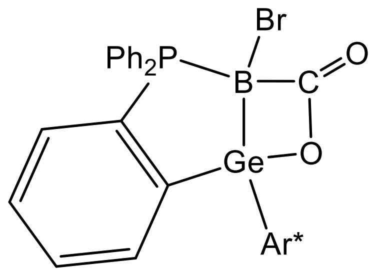

Figure SI38. $^{13}C\{^1H\}$ NMR spectrum of compound **8** synthesized with (99.0 %) $^{13}CO_2$.

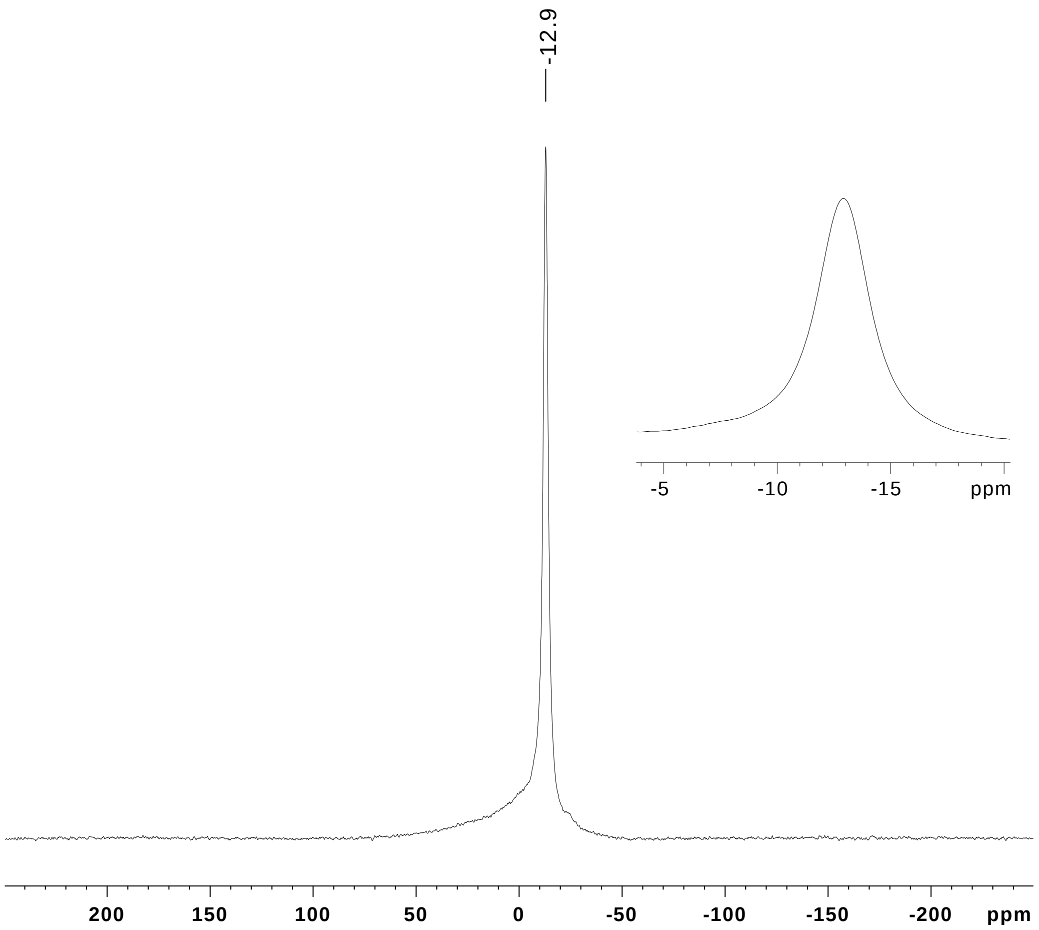

Current Data Parameters
NAME CR445_400
EXPNO 11
PROCNO 1

F2 - Acquisition Parameters
Date_ 20220609
Time 16.47
INSTRUM spect
PROBHD 5 mm X,F/H,F B
PULPROG zgbsig
TD 8192
SOLVENT C6D6
NS 1024
DS 0
SWH 64102.562 Hz
FIDRES 7.825020 Hz
AQ 0.0638976 sec
RG 2050
DW 7.800 usec
DE 6.00 usec
TE 299.2 K
D1 0.10000000 sec
D11 0.03000000 sec
TD0 1

======== CHANNEL f1 ========
NUC1 11B
P1 15.00 usec
P2 30.00 usec
PL1 -3.00 dB
SFO1 128.3711884 MHz

======== CHANNEL f2 ========
CPDPRG[2 garp
NUC2 1H
PCPD2 90.00 usec
PL2 -1.50 dB
PL12 15.65 dB
PL2W 11.35402870 W
PL12W 0.21885175 W
SFO2 400.1108002 MHz

F2 - Processing parameters
SI 8192
SF 128.3711880 MHz
WDW EM
SSB 0
LB 50.00 Hz
GB 0
PC 1.40

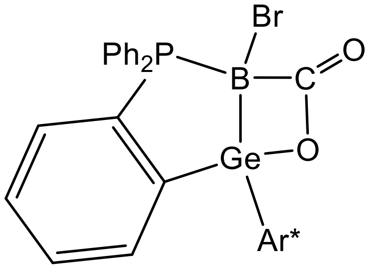

Figure SI39. $^{11}B\{^1H\}$ NMR spectrum of compound **8**.

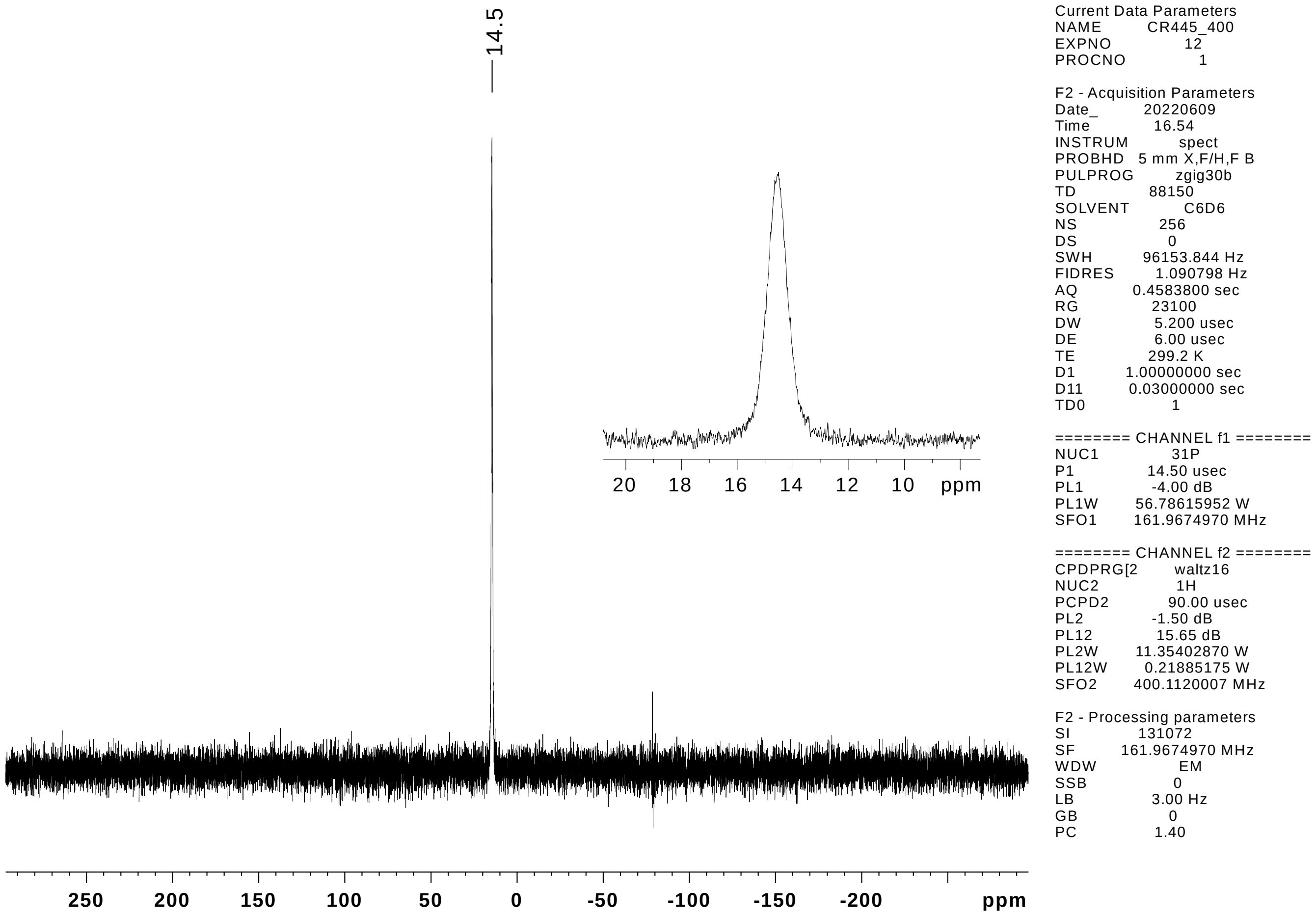

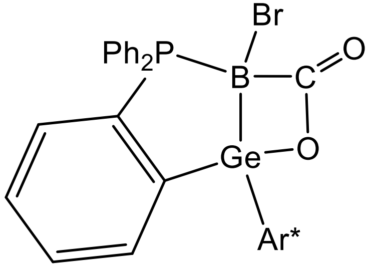

Figure SI40. $^{31}P\{^{1}H\}$ NMR spectrum of compound **8**.

NMR spectra of compound **9**.

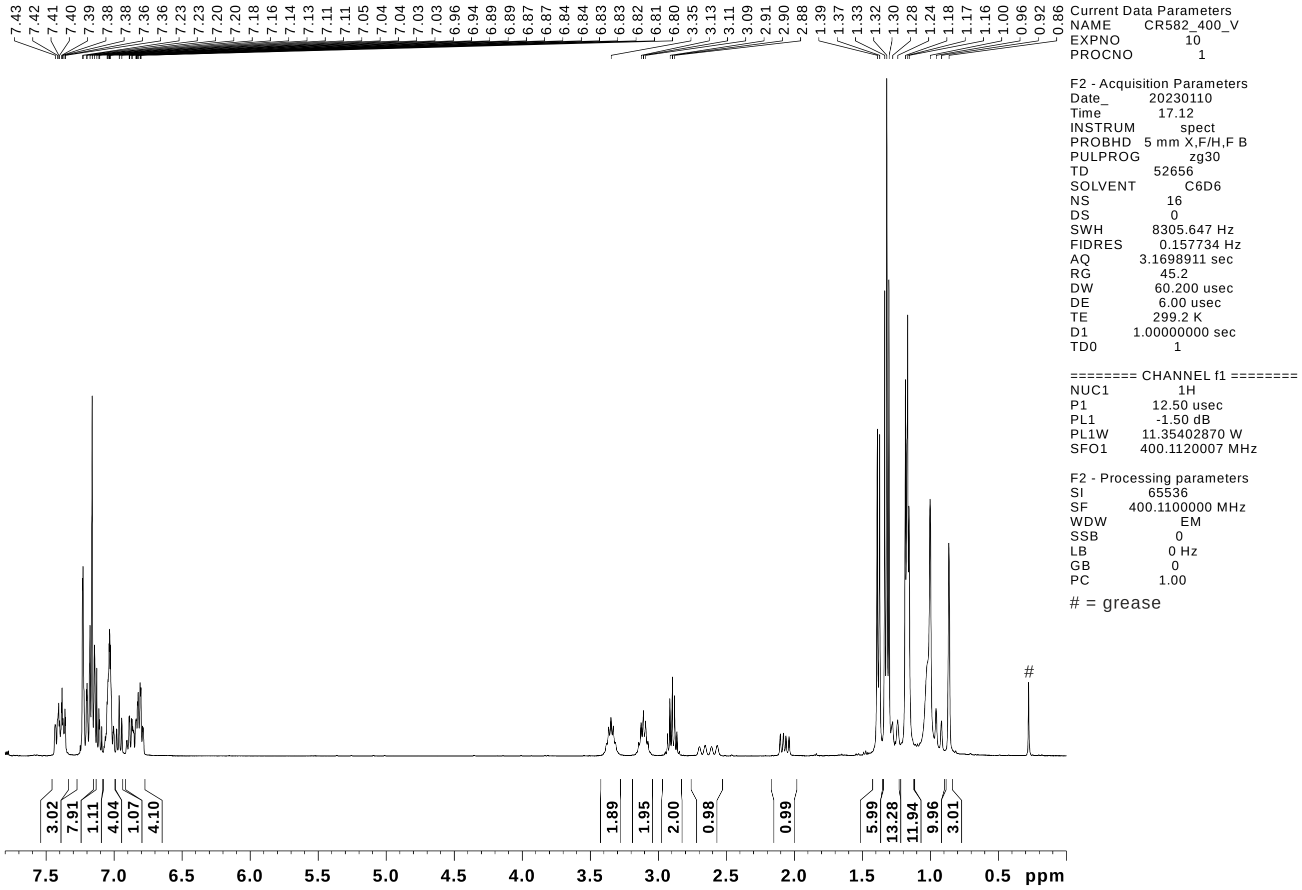

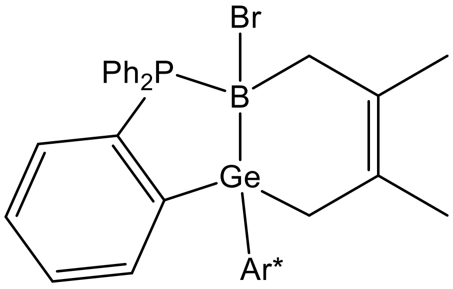

Figure SI41. ^{1}H NMR spectrum of compound **9**.

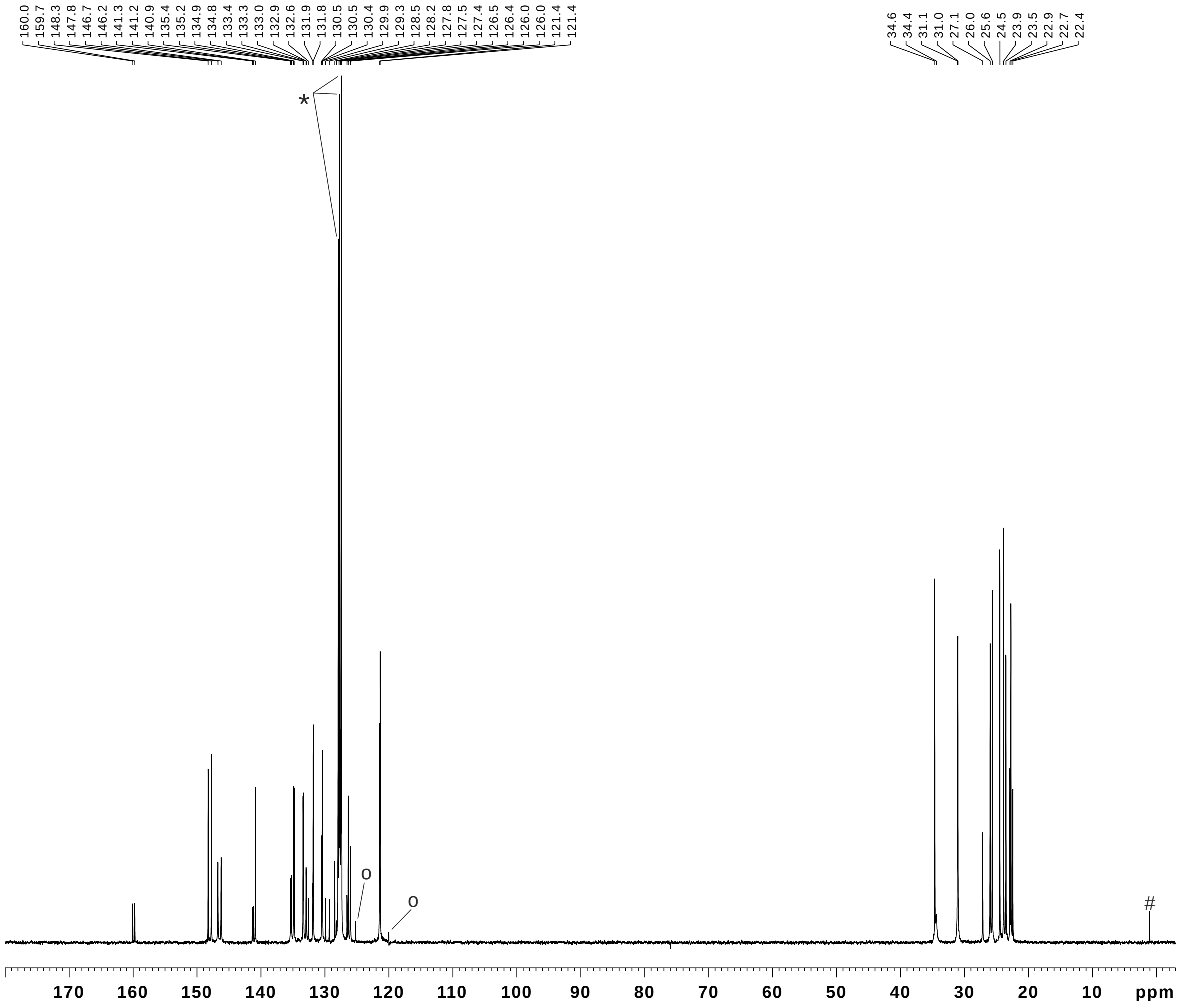

Current Data Parameters
NAME CR582_400_V
EXPNO 13
PROCNO 1

F2 - Acquisition Parameters
Date_ 20230111
Time 1.57
INSTRUM spect
PROBHD 5 mm X,F/H,F B
PULPROG udeft
TD 22218
SOLVENT C6D6
NS 5700
DS 0
SWH 30864.197 Hz
FIDRES 1.389153 Hz
AQ 0.3599316 sec
RG 32800
DW 16.200 usec
DE 6.00 usec
TE 299.2 K
D1 3.00000000 sec
D11 0.03000000 sec
D12 0.00002000 sec
D20 100.00000000 sec
TD0 1

======== CHANNEL f1 ========
NUC1 13C
P1 14.40 usec
P13 2000.00 usec
P26 500.00 usec
PL1 -5.90 dB
PL1W 117.26847076 W
SFO1 100.6198135 MHz
SP8 -0.91 dB
SP13 1.39 dB
SPNAM[8] Crp60,0.5,20.1
SPNAM[13 Crp60comp.4
SPOAL8 0.500
SPOAL13 0.500
SPOFFS8 0 Hz
SPOFFS13 0 Hz

======== CHANNEL f2 ========
CPDPRG[2 waltz16
NUC2 1H
PCPD2 90.00 usec
PL2 -1.50 dB
PL12 15.65 dB
PL2W 11.35402870 W
PL12W 0.21885175 W
SFO2 400.1120007 MHz

F2 - Processing parameters
SI 131072
SF 100.6077400 MHz
WDW EM
SSB 0
LB 2.00 Hz
GB 0
PC 1.40

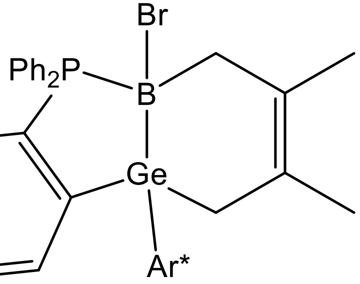

# = grease
o = trace impurities
* = C_6D_6

Figure SI42. $^{13}C\{^1H\}$ NMR spectrum of compound **9**.

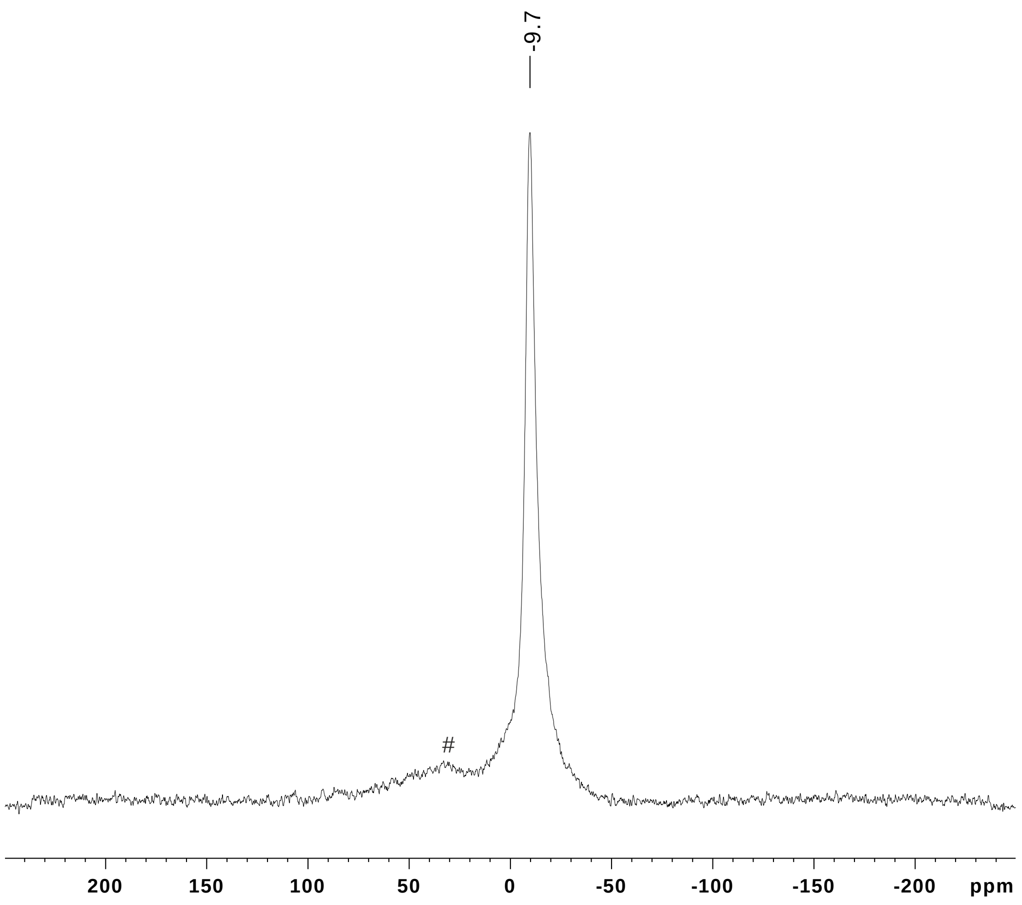

Current Data Parameters
NAME CR582_400
EXPNO 11
PROCNO 1

F2 - Acquisition Parameters
Date_ 20230109
Time 14.31
INSTRUM spect
PROBHD 5 mm X,F/H,F B
PULPROG zgbsig
TD 8192
SOLVENT C6D6
NS 1024
DS 0
SWH 64102.562 Hz
FIDRES 7.825020 Hz
AQ 0.0638976 sec
RG 2050
DW 7.800 usec
DE 6.00 usec
TE 299.2 K
D1 0.10000000 sec
D11 0.03000000 sec
TD0 1

======== CHANNEL f1 ========
NUC1 11B
P1 15.00 usec
P2 30.00 usec
PL1 -3.00 dB
SFO1 128.3711884 MHz

======== CHANNEL f2 ========
CPDPRG[2 garp
NUC2 1H
PCPD2 90.00 usec
PL2 -1.50 dB
PL12 15.65 dB
PL2W 11.35402870 W
PL12W 0.21885175 W
SFO2 400.1108002 MHz

F2 - Processing parameters
SI 8192
SF 128.3711880 MHz
WDW EM
SSB 0
LB 50.00 Hz
GB 0
PC 1.40

# = glass background signal

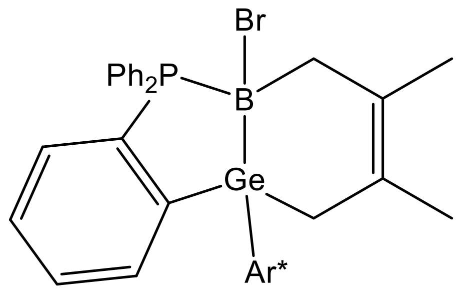

Figure SI43. $^{11}B\{^1H\}$ NMR spectrum of compound **9**.

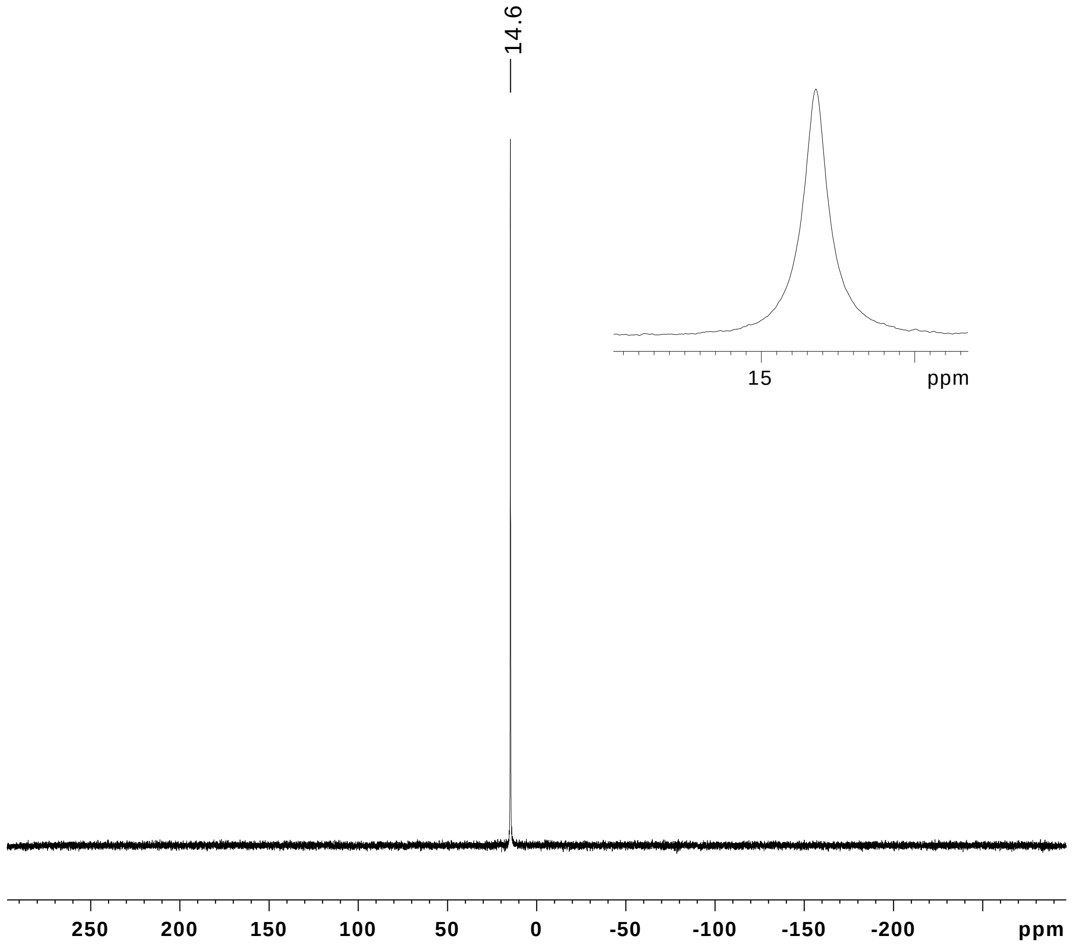

Current Data Parameters
NAME CR582_400
EXPNO 12
PROCNO 1

F2 - Acquisition Parameters
Date_ 20230109
Time 14.38
INSTRUM spect
PROBHD 5 mm X,F/H,F B
PULPROG zgig30b
TD 88150
SOLVENT C6D6
NS 256
DS 0
SWH 96153.844 Hz
FIDRES 1.090798 Hz
AQ 0.4583800 sec
RG 23100
DW 5.200 usec
DE 6.00 usec
TE 299.2 K
D1 1.00000000 sec
D11 0.03000000 sec
TD0 1

======== CHANNEL f1 ========
NUC1 31P
P1 14.50 usec
PL1 -4.00 dB
PL1W 56.78615952 W
SFO1 161.9674970 MHz

======== CHANNEL f2 ========
CPDPRG[2 waltz16
NUC2 1H
PCPD2 90.00 usec
PL2 -1.50 dB
PL12 15.65 dB
PL2W 11.35402870 W
PL12W 0.21885175 W
SFO2 400.1120007 MHz

F2 - Processing parameters
SI 131072
SF 161.9674970 MHz
WDW EM
SSB 0
LB 3.00 Hz
GB 0
PC 1.40

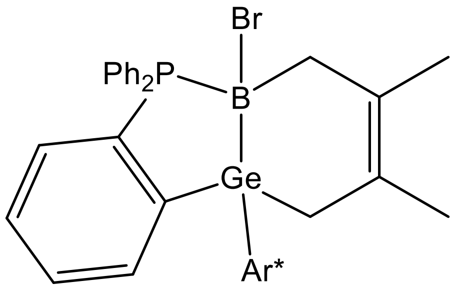

Figure SI44. $^{31}P\{^{1}H\}$ NMR spectrum of compound **9**.

NMR spectra of compound **10**.

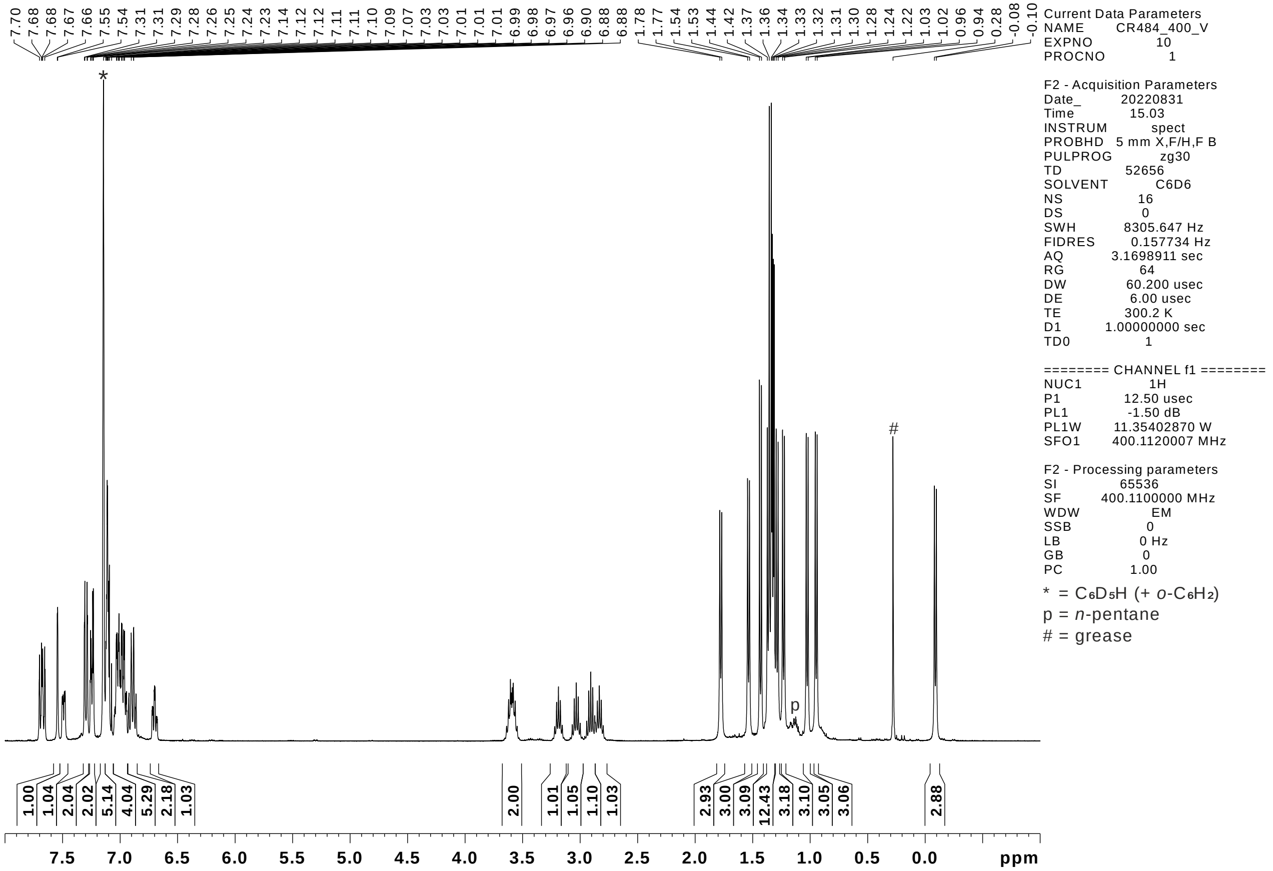

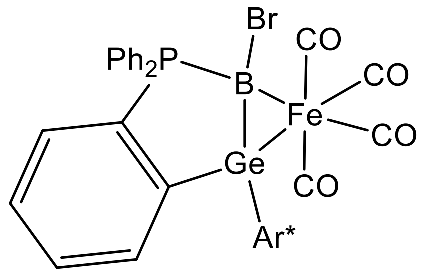

Figure SI45. ^{1}H NMR spectrum of compound **10**.

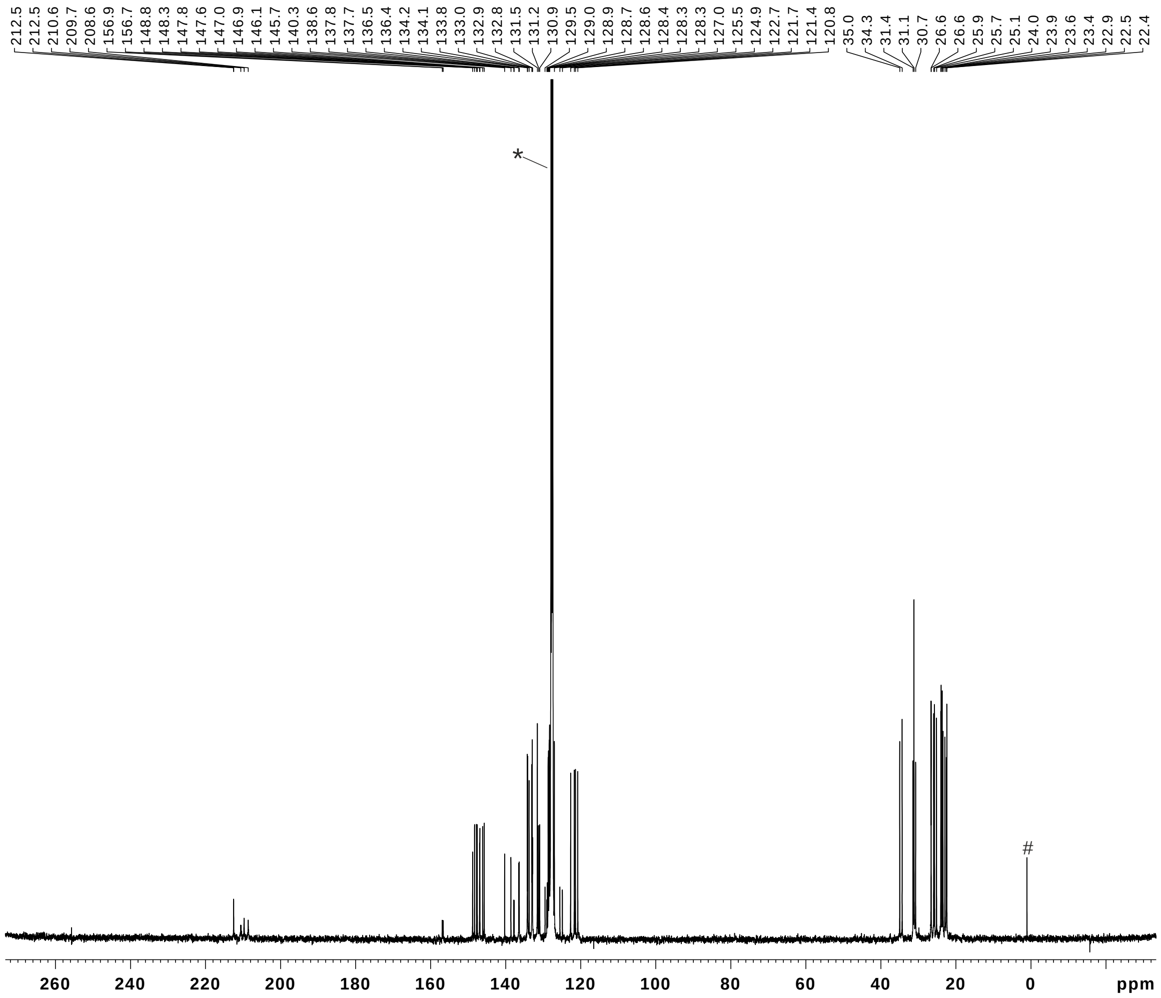

Current Data Parameters
NAME CR484_400_V_2
EXPNO 25
PROCNO 1

F2 - Acquisition Parameters
Date_ 20220901
Time 7.26
INSTRUM spect
PROBHD 5 mm X,F/H,F B
PULPROG udeft
TD 22218
SOLVENT C6D6
NS 5400
DS 0
SWH 30864.197 Hz
FIDRES 1.389153 Hz
AQ 0.3599316 sec
RG 32800
DW 16.200 usec
DE 6.00 usec
TE 299.2 K
D1 3.00000000 sec
D11 0.03000000 sec
D12 0.00002000 sec
D20 100.00000000 sec
TD0 1

======== CHANNEL f1 ========
NUC1 13C
P1 14.40 usec
P13 2000.00 usec
P26 500.00 usec
PL1 -5.90 dB
PL1W 117.26847076 W
SFO1 100.6198135 MHz
SP8 -0.91 dB
SP13 1.39 dB
SPNAM[8] Crp60,0.5,20.1
SPNAM[13 Crp60comp.4
SPOAL8 0.500
SPOAL13 0.500
SPOFFS8 0 Hz
SPOFFS13 0 Hz

======== CHANNEL f2 ========
CPDPRG[2 waltz16
NUC2 1H
PCPD2 90.00 usec
PL2 -1.50 dB
PL12 15.65 dB
PL2W 11.35402870 W
PL12W 0.21885175 W
SFO2 400.1120007 MHz

F2 - Processing parameters
SI 131072
SF 100.6077400 MHz
WDW EM
SSB 0
LB 2.00 Hz
GB 0
PC 1.40

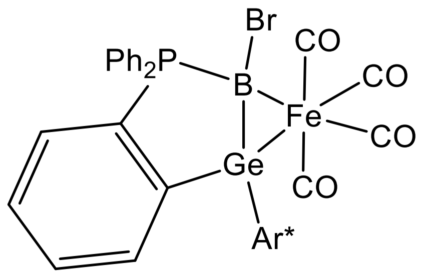

# = grease
* = C_6D_6

Figure SI46. $^{13}C\{^1H\}$ NMR spectrum of compound **10**.

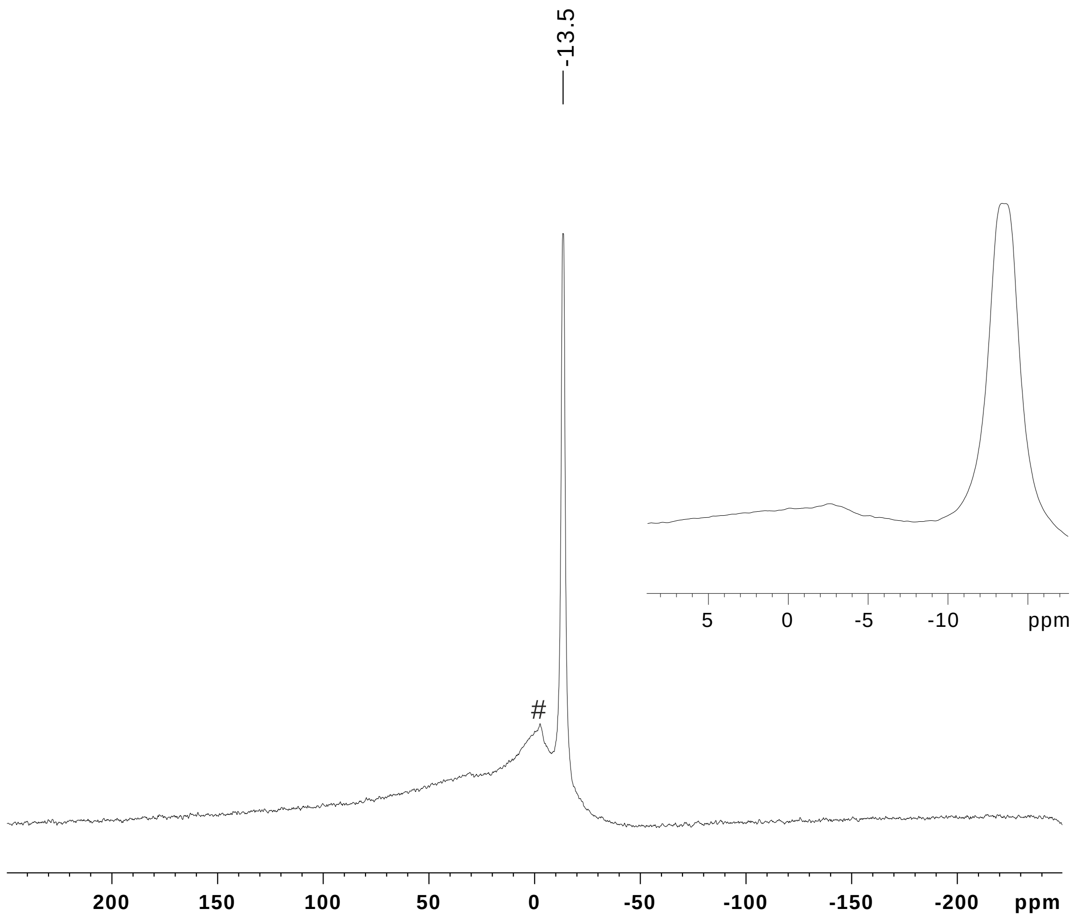

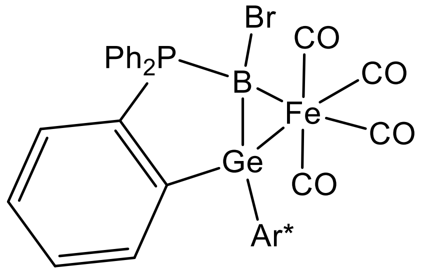

Figure SI47. $^{11}B\{^{1}H\}$ NMR spectrum of compound **10**.

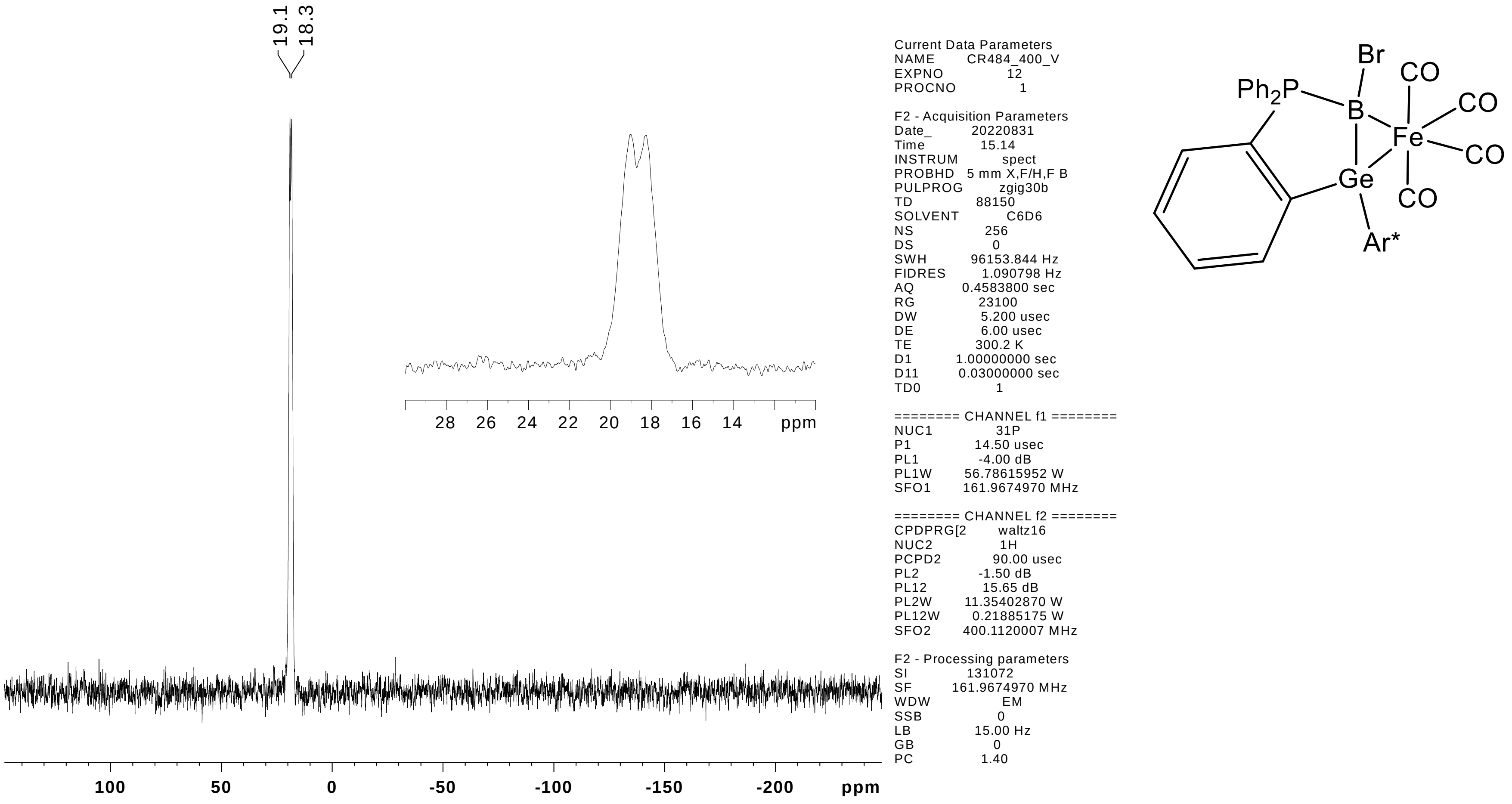

Figure SI48. $^{31}P\{^{1}H\}$ NMR spectrum of compound **10**.

NMR spectra of compound **11**.

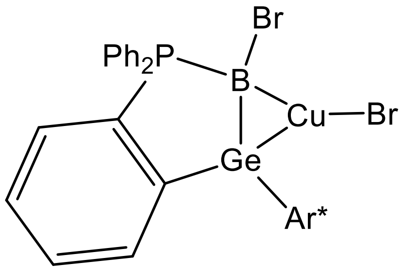

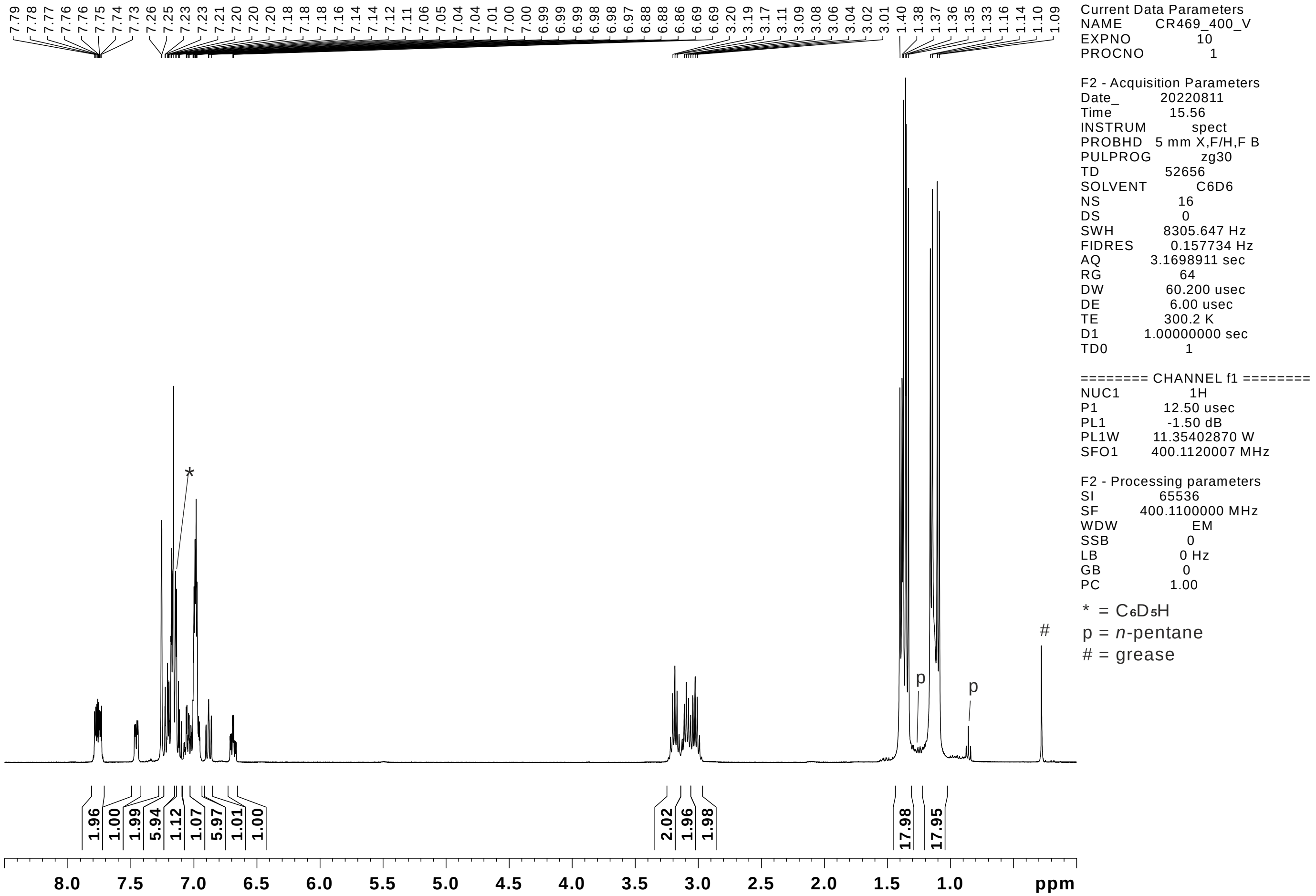

Figure SI49. ^{1}H NMR spectrum of compound **11**.

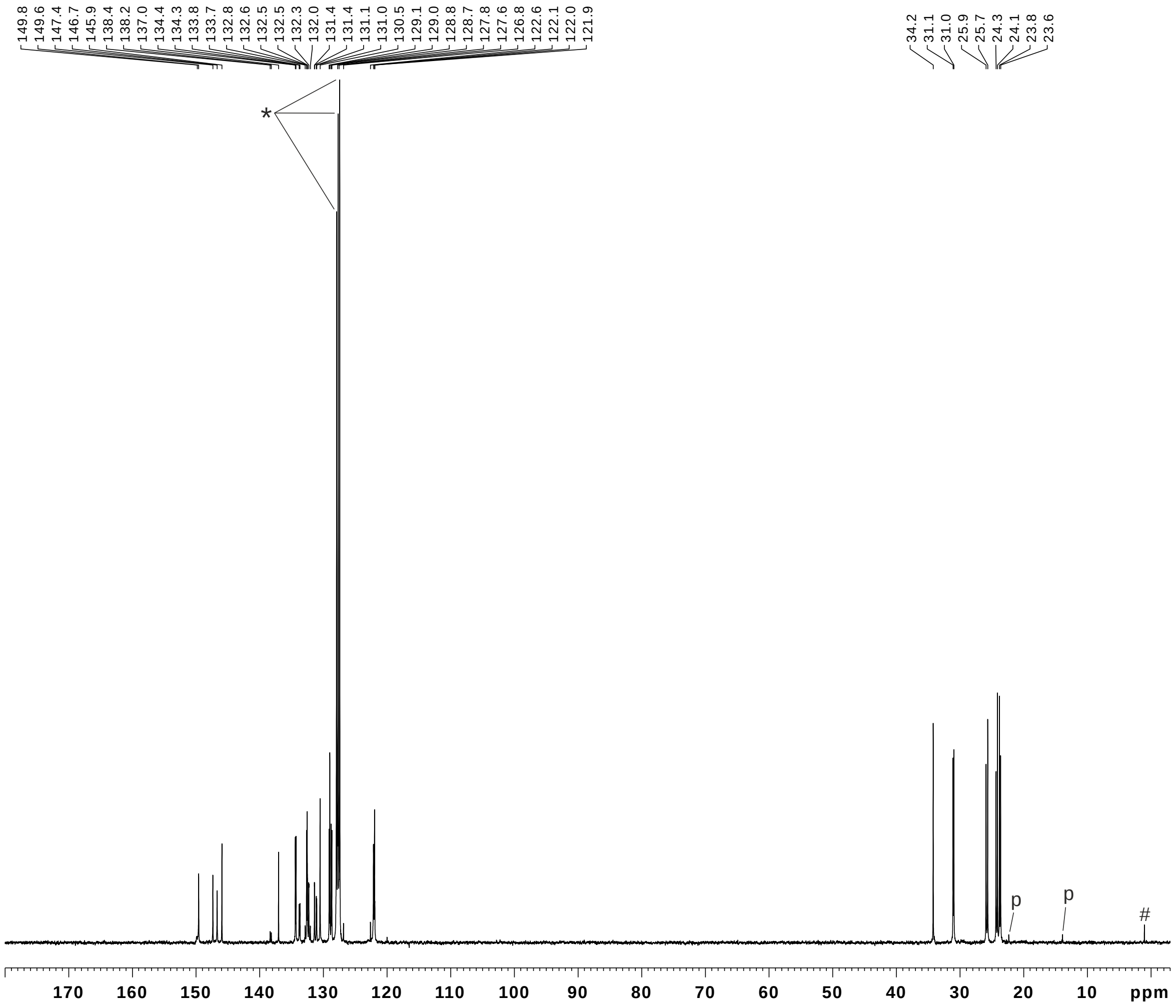

Current Data Parameters
NAME CR469_400_V
EXPNO 14
PROCNO 1

F2 - Acquisition Parameters
Date_ 20220812
Time 1.59
INSTRUM spect
PROBHD 5 mm X,F/H,F B
PULPROG udeft
TD 22218
SOLVENT C6D6
NS 5700
DS 0
SWH 30864.197 Hz
FIDRES 1.389153 Hz
AQ 0.3599316 sec
RG 32800
DW 16.200 usec
DE 6.00 usec
TE 300.2 K
D1 3.00000000 sec
D11 0.03000000 sec
D12 0.00002000 sec
D20 100.00000000 sec
TD0 1

======== CHANNEL f1 ========
NUC1 13C
P1 14.40 usec
P13 2000.00 usec
P26 500.00 usec
PL1 -5.90 dB
PL1W 117.26847076 W
SFO1 100.6198135 MHz
SP8 -0.91 dB
SP13 1.39 dB
SPNAM[8] Crp60,0.5,20.1
SPNAM[13 Crp60comp.4
SPOAL8 0.500
SPOAL13 0.500
SPOFFS8 0 Hz
SPOFFS13 0 Hz

======== CHANNEL f2 ========
CPDPRG[2 waltz16
NUC2 1H
PCPD2 90.00 usec
PL2 -1.50 dB
PL12 15.65 dB
PL2W 11.35402870 W
PL12W 0.21885175 W
SFO2 400.1120007 MHz

F2 - Processing parameters
SI 131072
SF 100.6077400 MHz
WDW EM
SSB 0
LB 2.00 Hz
GB 0
PC 1.40

p = *n*-pentane
# = grease
* = C_6D_6

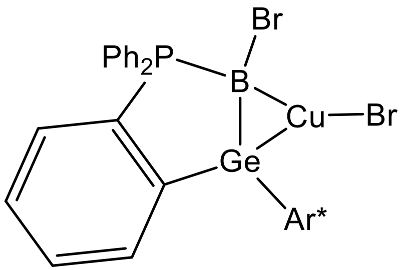

Figure SI50. $^{13}C\{^1H\}$ NMR spectrum of compound **11**.

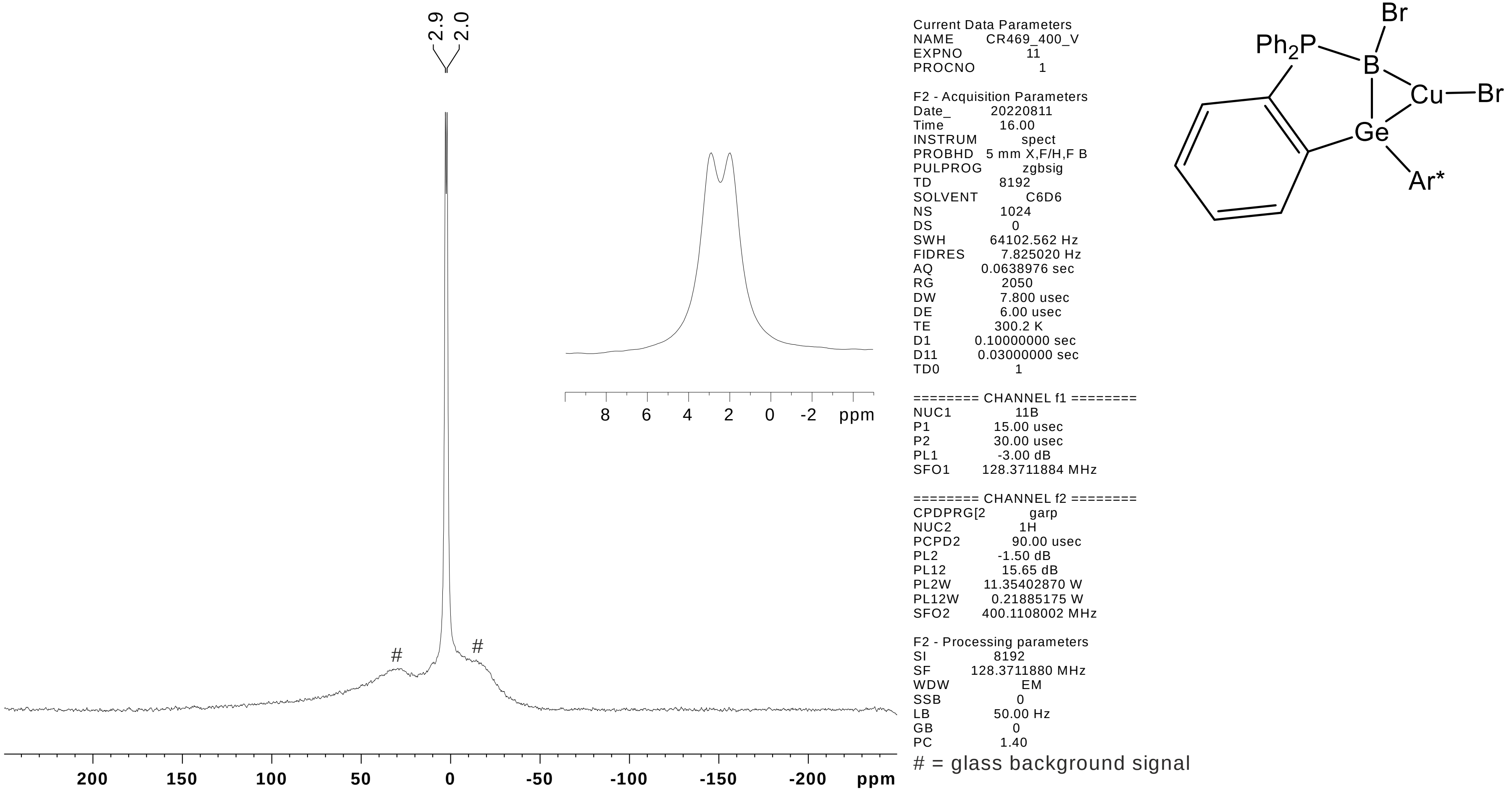

Figure SI51. $^{11}B\{^{1}H\}$ NMR spectrum of compound **11**.

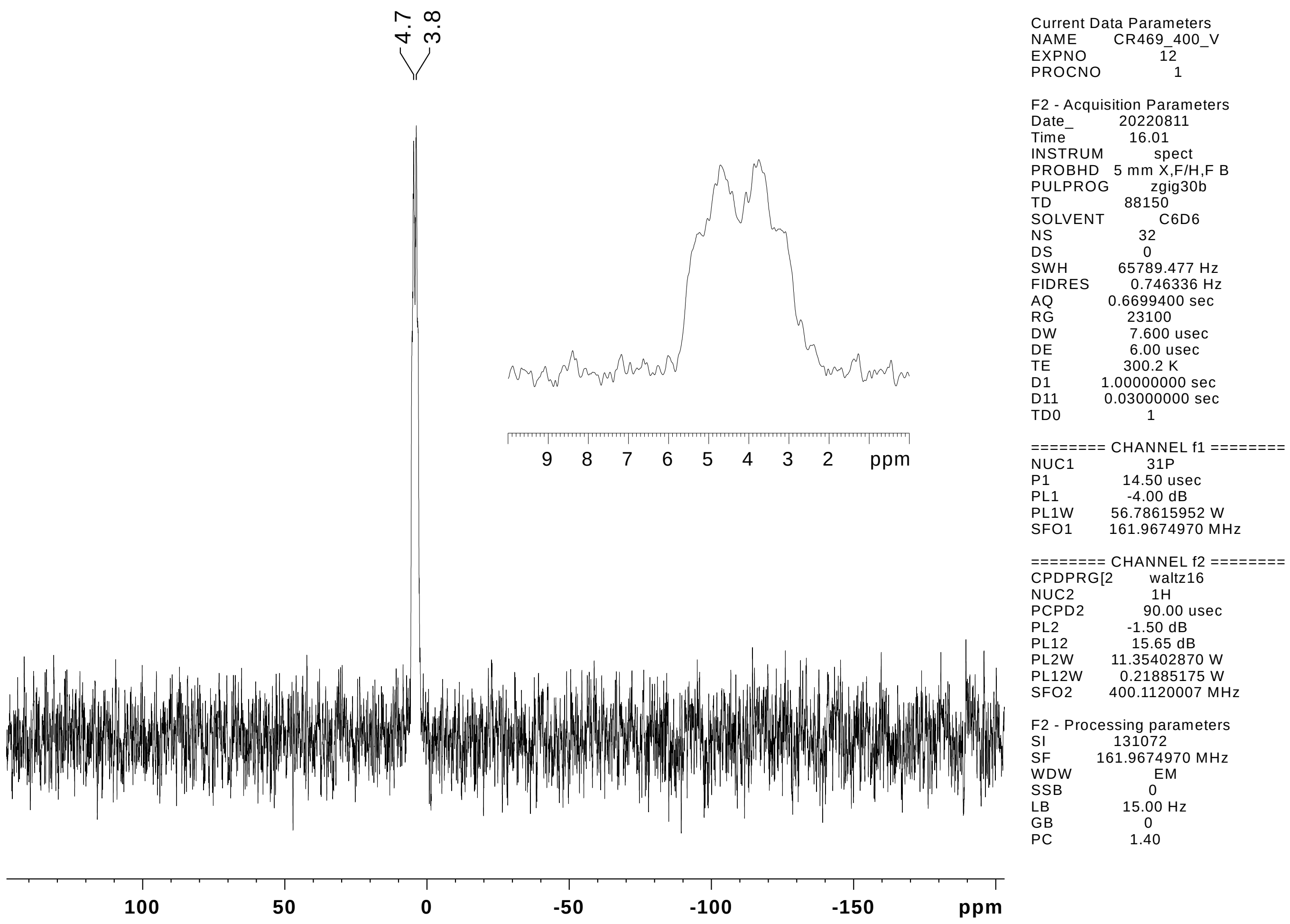

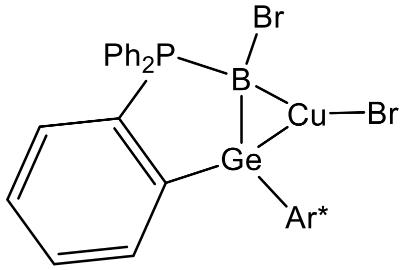

Figure SI52. $^{31}P\{^{1}H\}$ NMR spectrum of compound **11**.

NMR spectra of compound **12**.

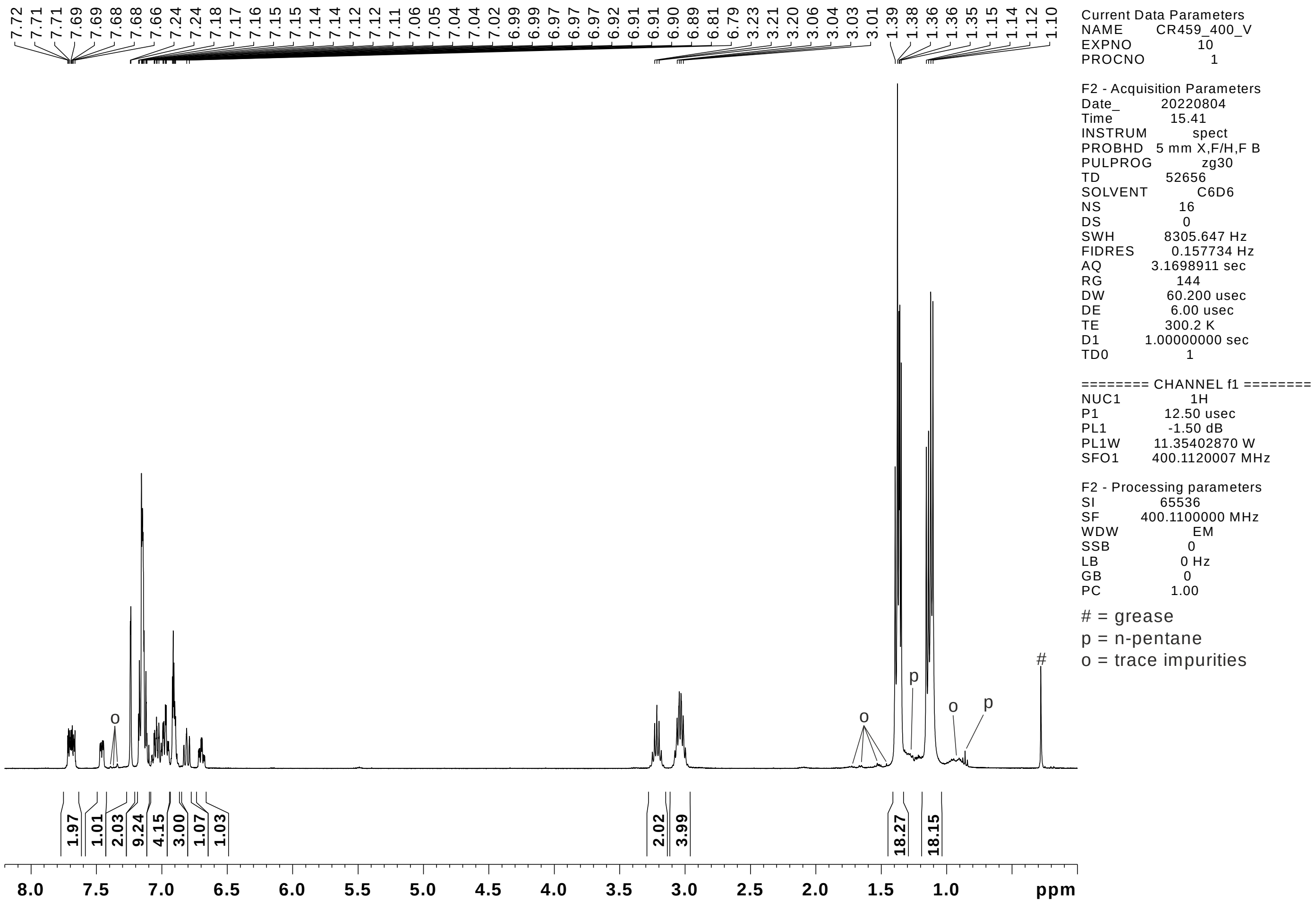

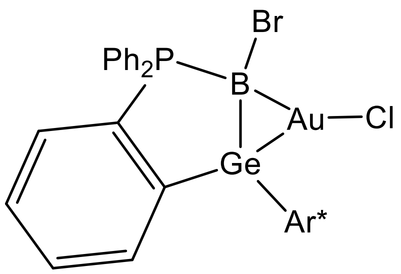

Figure SI53. ^{1}H NMR spectrum of compound **12**.

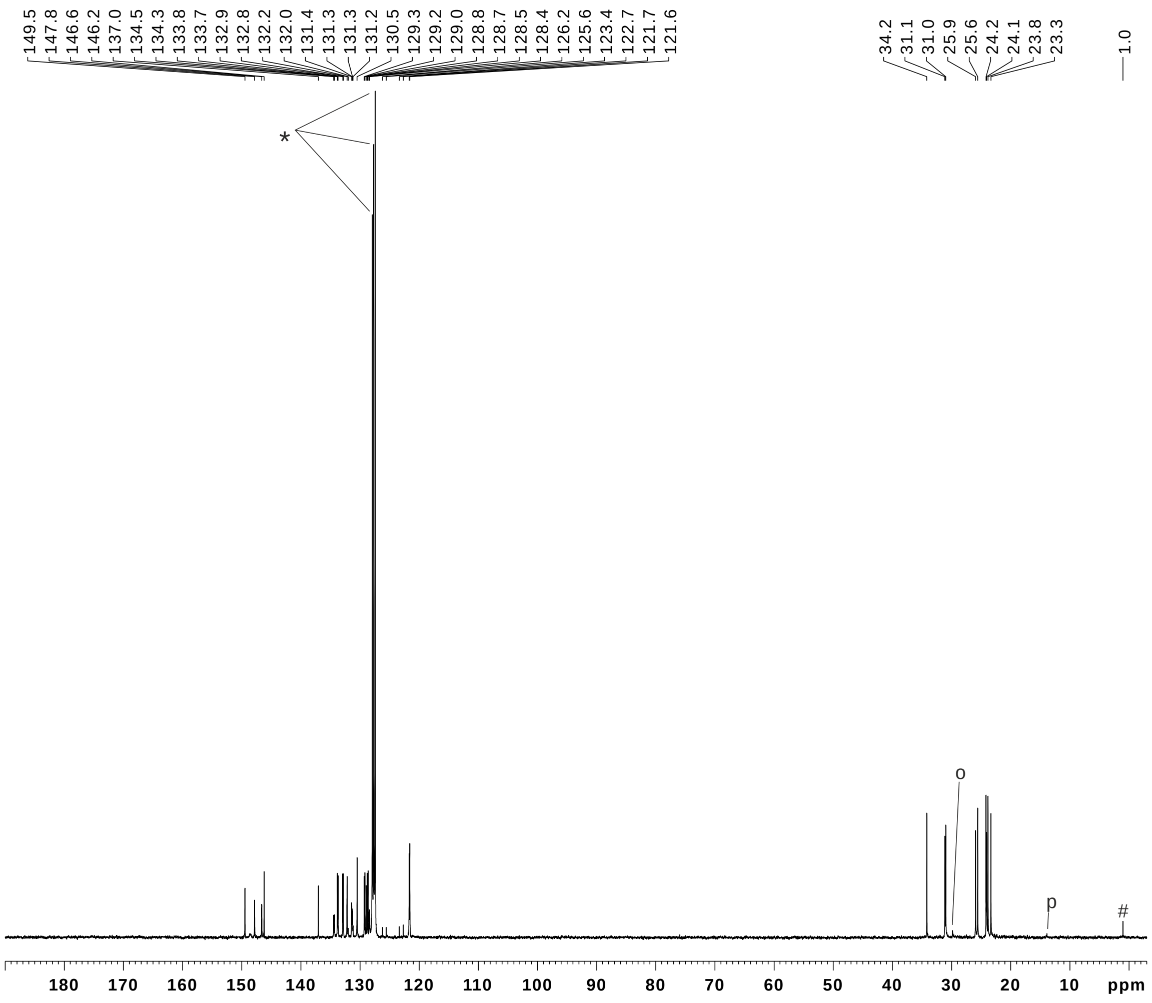

Current Data Parameters
NAME CR459_400_V_2
EXPNO 17
PROCNO 1

F2 - Acquisition Parameters
Date_ 20220805
Time 7.44
INSTRUM spect
PROBHD 5 mm X,F/H,F B
PULPROG udeft
TD 22218
SOLVENT C6D6
NS 5700
DS 0
SWH 30864.197 Hz
FIDRES 1.389153 Hz
AQ 0.3599316 sec
RG 32800
DW 16.200 usec
DE 6.00 usec
TE 300.2 K
D1 3.00000000 sec
D11 0.03000000 sec
D12 0.00002000 sec
D20 100.00000000 sec
TD0 1

======== CHANNEL f1 ========
NUC1 13C
P1 14.40 usec
P13 2000.00 usec
P26 500.00 usec
PL1 -5.90 dB
PL1W 117.26847076 W
SFO1 100.6198135 MHz
SP8 -0.91 dB
SP13 1.39 dB
SPNAM[8] Crp60,0.5,20.1
SPNAM[13 Crp60comp.4
SPOAL8 0.500
SPOAL13 0.500
SPOFFS8 0 Hz
SPOFFS13 0 Hz

======== CHANNEL f2 ========
CPDPRG[2 waltz16
NUC2 1H
PCPD2 90.00 usec
PL2 -1.50 dB
PL12 15.65 dB
PL2W 11.35402870 W
PL12W 0.21885175 W
SFO2 400.1120007 MHz

F2 - Processing parameters
SI 131072
SF 100.6077400 MHz
WDW EM
SSB 0
LB 2.00 Hz
GB 0
PC 1.40

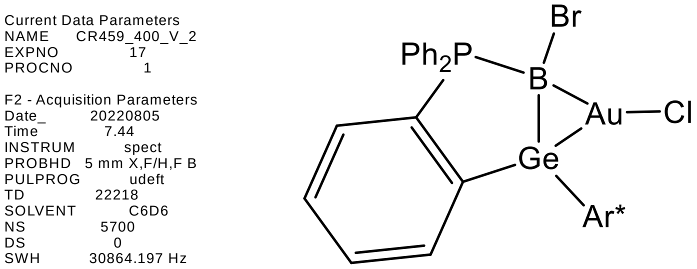

p = *n*-pentane
# = grease
o = trace impurity
* = C_6D_6

Figure SI54. $^{13}C\{^1H\}$ NMR spectrum of compound **12**.

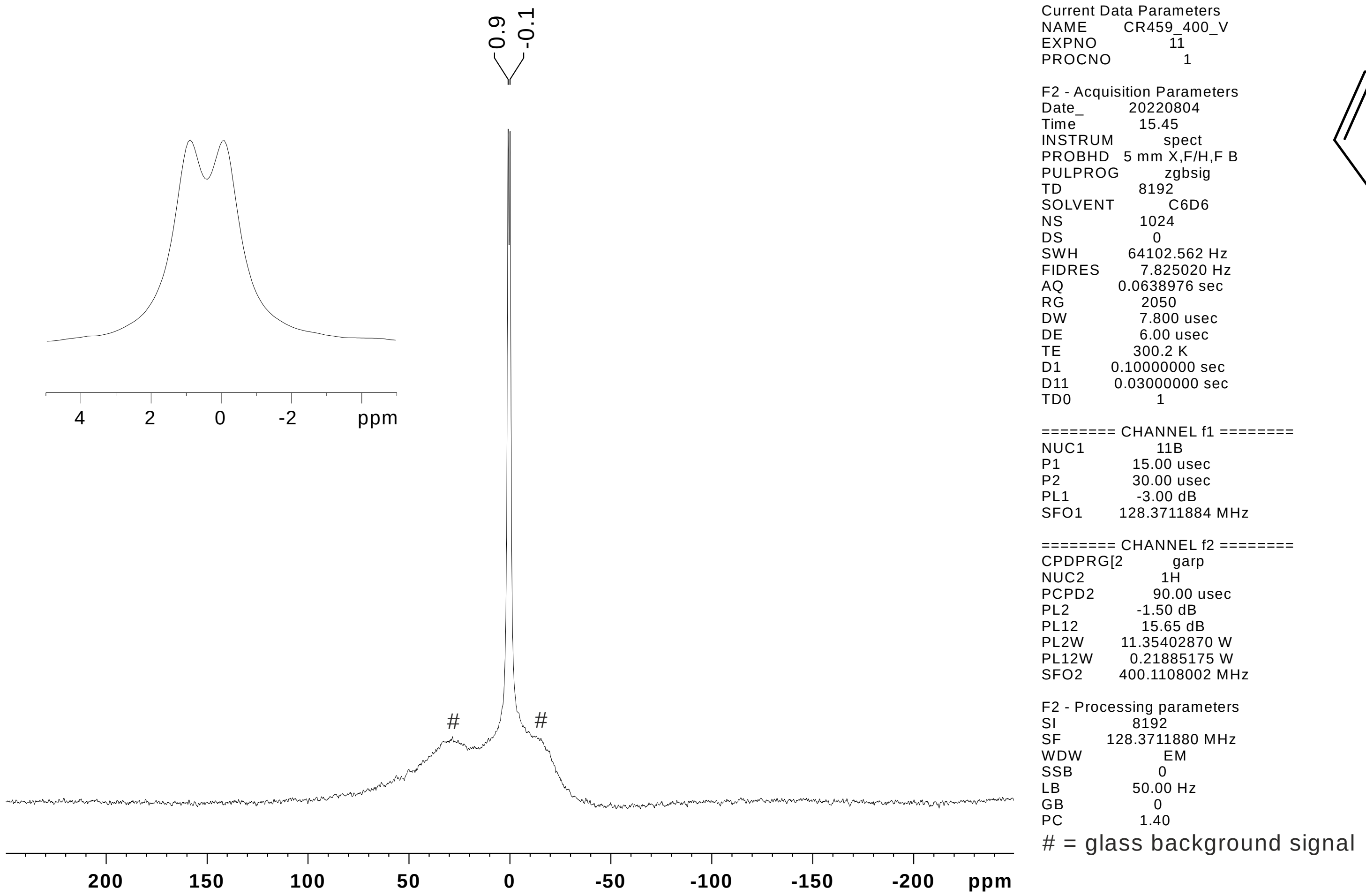

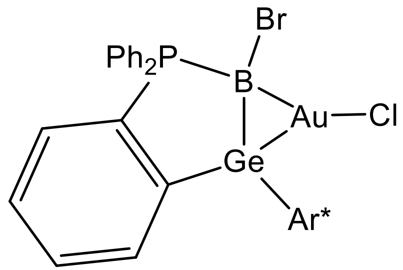

Figure SI55. $^{11}B\{^{1}H\}$ NMR spectrum of compound **12**.

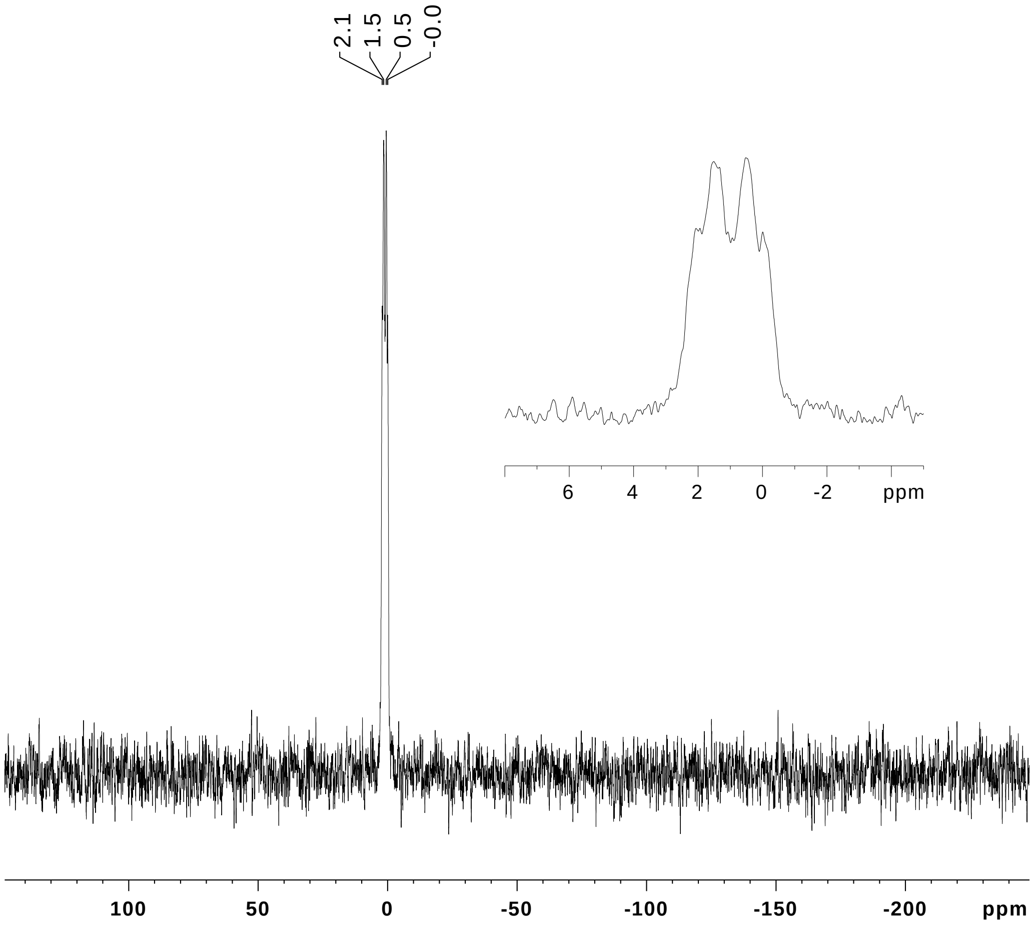

Current Data Parameters
NAME CR459_400_V
EXPNO 12
PROCNO 1

F2 - Acquisition Parameters
Date_ 20220804
Time 15.52
INSTRUM spect
PROBHD 5 mm X,F/H,F B
PULPROG zgig30b
TD 88150
SOLVENT C6D6
NS 256
DS 0
SWH 96153.844 Hz
FIDRES 1.090798 Hz
AQ 0.4583800 sec
RG 23100
DW 5.200 usec
DE 6.00 usec
TE 300.2 K
D1 1.00000000 sec
D11 0.03000000 sec
TD0 1

======== CHANNEL f1 ========
NUC1 31P
P1 14.50 usec
PL1 -4.00 dB
PL1W 56.78615952 W
SFO1 161.9674970 MHz

======== CHANNEL f2 ========
CPDPRG[2 waltz16
NUC2 1H
PCPD2 90.00 usec
PL2 -1.50 dB
PL12 15.65 dB
PL2W 11.35402870 W
PL12W 0.21885175 W
SFO2 400.1120007 MHz

F2 - Processing parameters
SI 131072
SF 161.9674970 MHz
WDW EM
SSB 0
LB 15.00 Hz
GB 0
PC 1.40

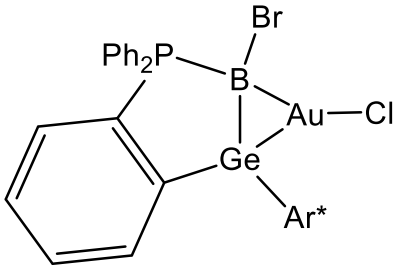

Figure SI56. $^{31}P\{^1H\}$ NMR spectrum of compound **12**.

NMR spectra of compound **13**.

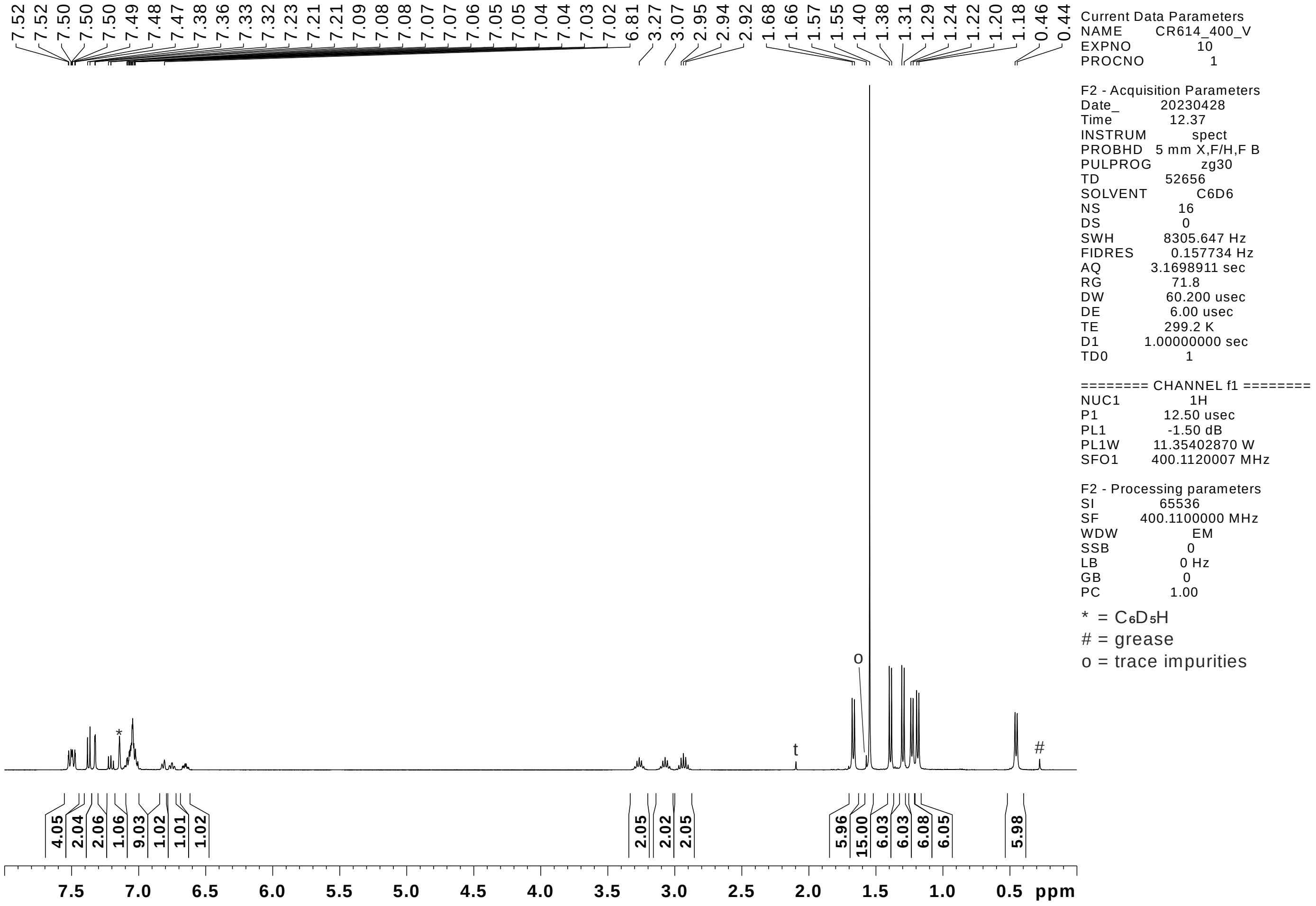

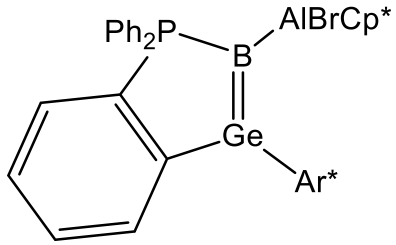

Figure SI57. ^{1}H NMR spectrum of compound **13**.

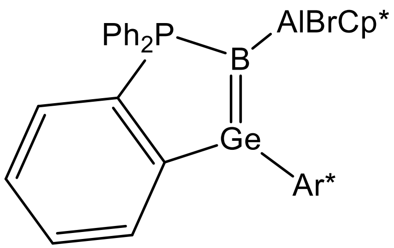

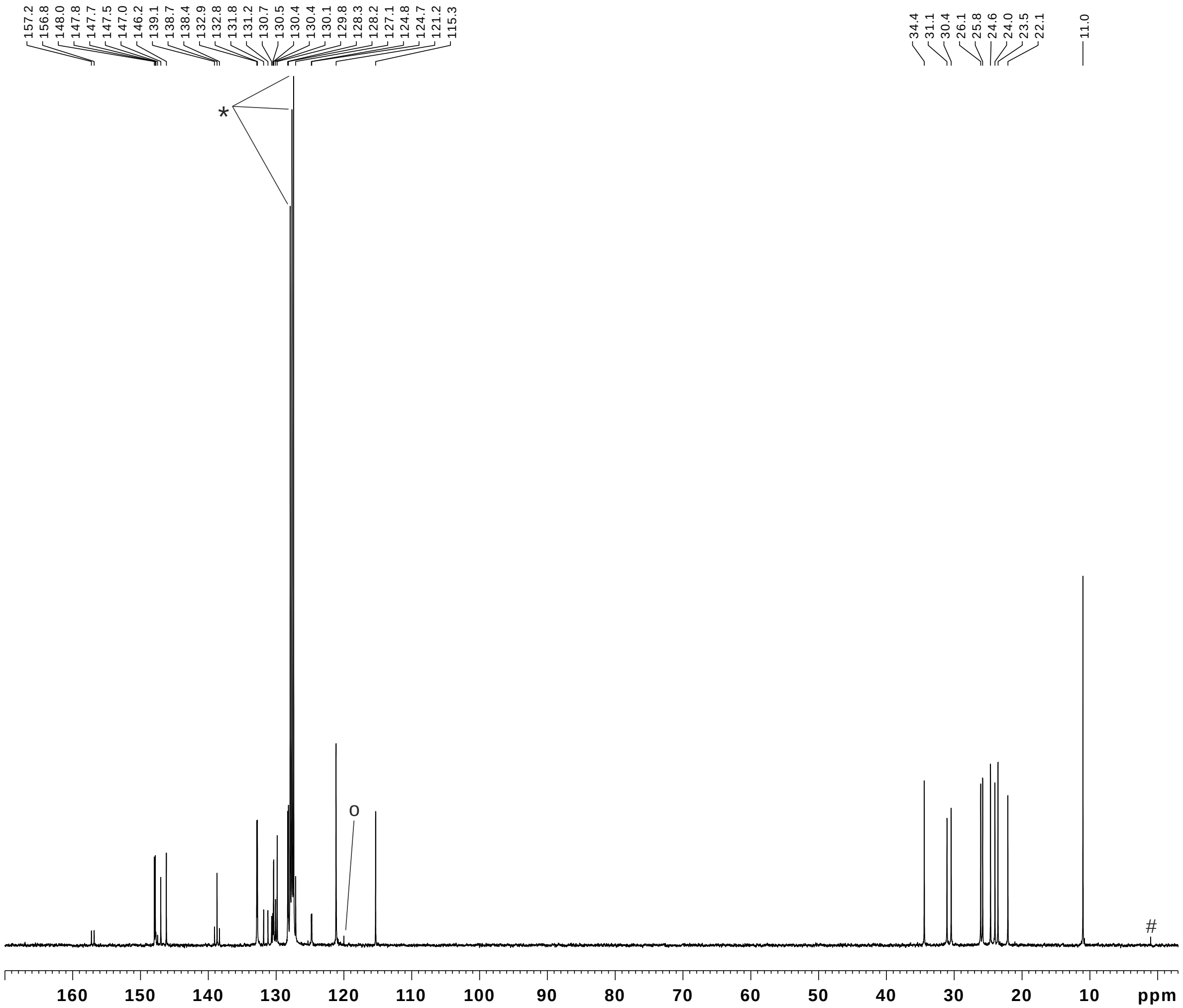

Current Data Parameters
NAME CR614_400_V
EXPNO 12
PROCNO 1

F2 - Acquisition Parameters
Date_ 20230430
Time 1.40
INSTRUM spect
PROBHD 5 mm X,F/H,F B
PULPROG udeft
TD 22218
SOLVENT C6D6
NS 5400
DS 0
SWH 30864.197 Hz
FIDRES 1.389153 Hz
AQ 0.3599316 sec
RG 32800
DW 16.200 usec
DE 6.00 usec
TE 300.2 K
D1 3.00000000 sec
D11 0.03000000 sec
D12 0.00002000 sec
D20 100.00000000 sec
TD0 1

======== CHANNEL f1 ========
NUC1 13C
P1 14.40 usec
P13 2000.00 usec
P26 500.00 usec
PL1 -5.90 dB
PL1W 117.26847076 W
SFO1 100.6198135 MHz
SP8 -0.91 dB
SP13 1.39 dB
SPNAM[8] Crp60,0.5,20.1
SPNAM[13 Crp60comp.4
SPOAL8 0.500
SPOAL13 0.500
SPOFFS8 0 Hz
SPOFFS13 0 Hz

======== CHANNEL f2 ========
CPDPRG[2 waltz16
NUC2 1H
PCPD2 90.00 usec
PL2 -1.50 dB
PL12 15.65 dB
PL2W 11.35402870 W
PL12W 0.21885175 W
SFO2 400.1120007 MHz

F2 - Processing parameters
SI 131072
SF 100.6077400 MHz
WDW EM
SSB 0
LB 2.00 Hz
GB 0
PC 1.40

# = grease
o = trace impurity
* = C_6D_6

Figure SI58. $^{13}C\{^1H\}$ NMR spectrum of compound **13**.

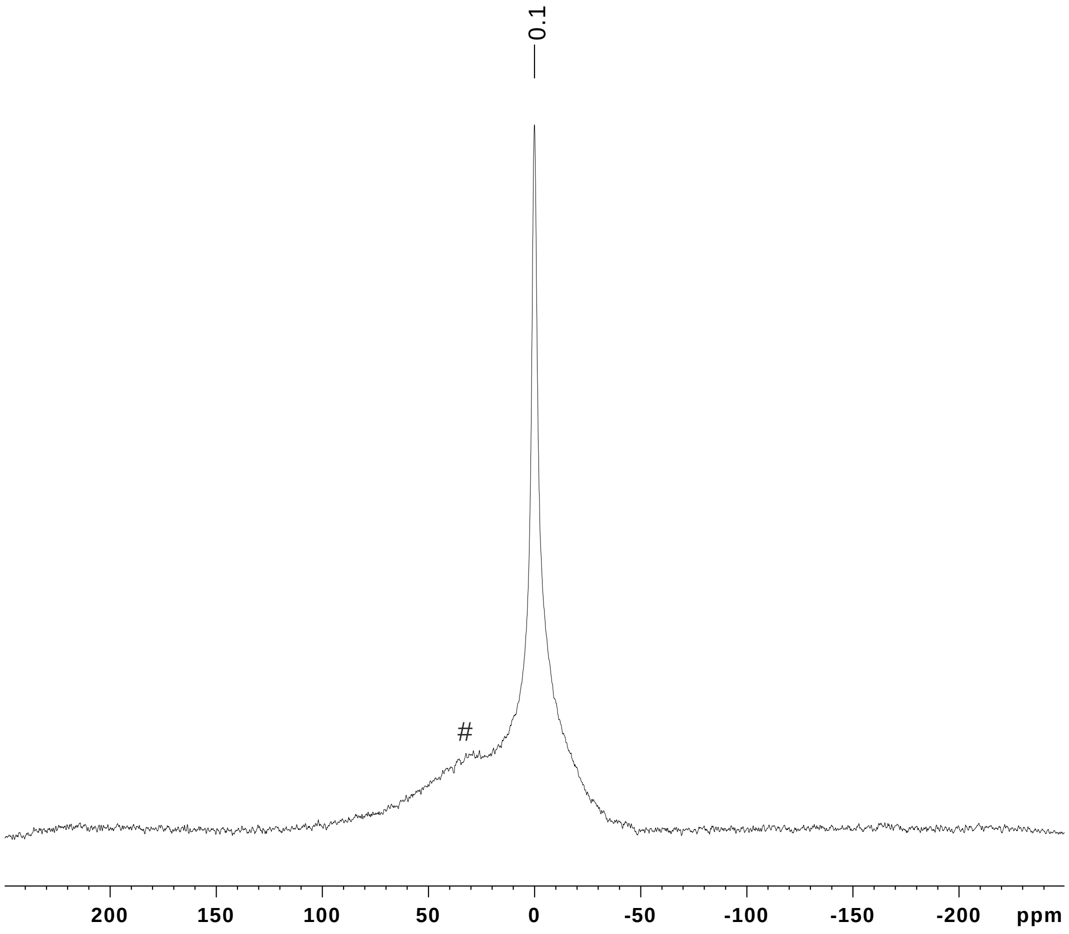

Current Data Parameters
NAME CR614_400
EXPNO 21
PROCNO 1

F2 - Acquisition Parameters
Date_ 20230427
Time 17.09
INSTRUM spect
PROBHD 5 mm X,F/H,F B
PULPROG zgbsig
TD 8192
SOLVENT C6D6
NS 1024
DS 0
SWH 64102.562 Hz
FIDRES 7.825020 Hz
AQ 0.0638976 sec
RG 2050
DW 7.800 usec
DE 6.00 usec
TE 299.2 K
D1 0.10000000 sec
D11 0.03000000 sec
TD0 1

======== CHANNEL f1 ========
NUC1 11B
P1 15.00 usec
P2 30.00 usec
PL1 -3.00 dB
SFO1 128.3711884 MHz

======== CHANNEL f2 ========
CPDPRG[2 garp
NUC2 1H
PCPD2 90.00 usec
PL2 -1.50 dB
PL12 15.65 dB
PL2W 11.35402870 W
PL12W 0.21885175 W
SFO2 400.1108002 MHz

F2 - Processing parameters
SI 8192
SF 128.3711880 MHz
WDW EM
SSB 0
LB 50.00 Hz
GB 0
PC 1.40

# = glass background signal

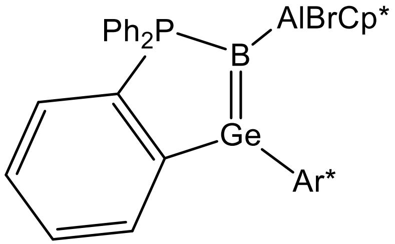

Figure SI59. $^{11}B\{^{1}H\}$ NMR spectrum of compound **13**.

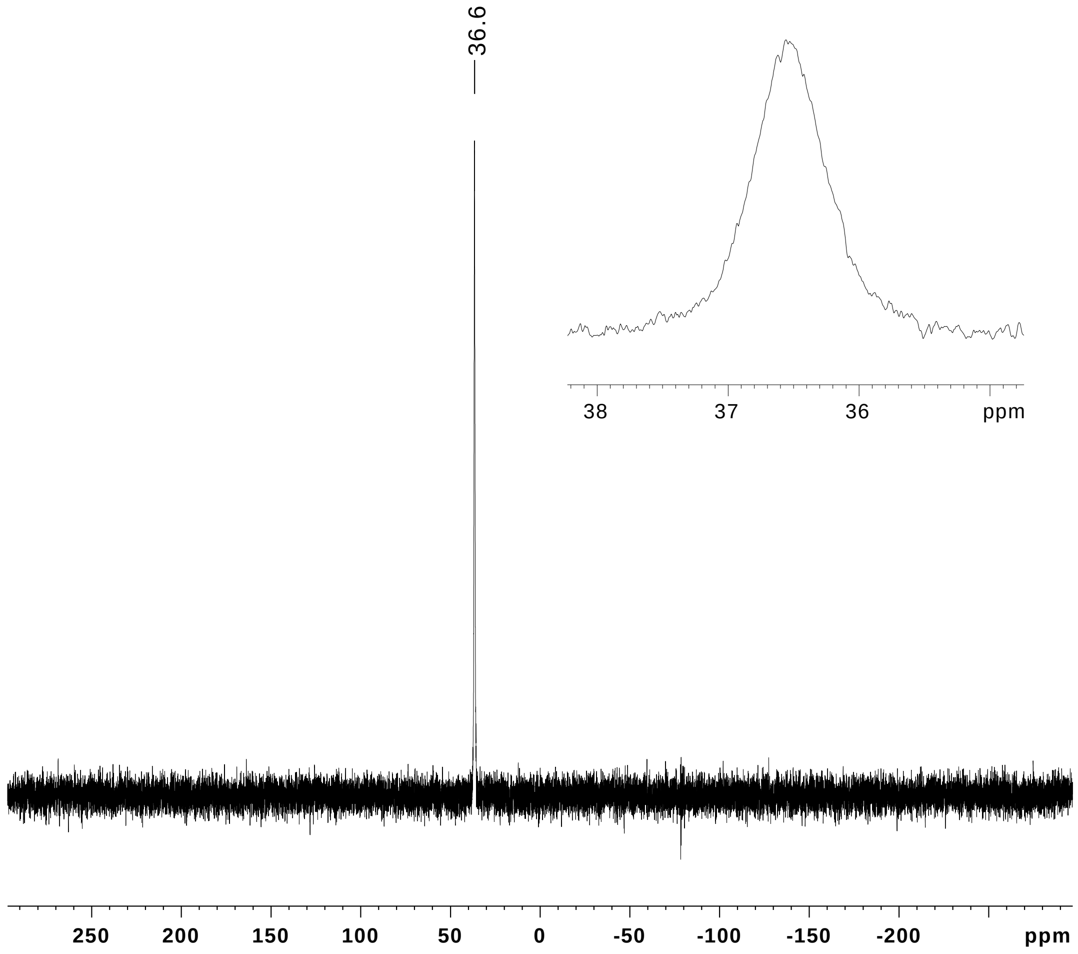

Current Data Parameters
NAME CR614_400
EXPNO 22
PROCNO 1

F2 - Acquisition Parameters
Date_ 20230427
Time 17.16
INSTRUM spect
PROBHD 5 mm X,F/H,F B
PULPROG zgig30b
TD 88150
SOLVENT C6D6
NS 256
DS 0
SWH 96153.844 Hz
FIDRES 1.090798 Hz
AQ 0.4583800 sec
RG 23100
DW 5.200 usec
DE 6.00 usec
TE 299.2 K
D1 1.00000000 sec
D11 0.03000000 sec
TD0 1

======== CHANNEL f1 ========
NUC1 31P
P1 14.50 usec
PL1 -4.00 dB
PL1W 56.78615952 W
SFO1 161.9674970 MHz

======== CHANNEL f2 ========
CPDPRG[2 waltz16
NUC2 1H
PCPD2 90.00 usec
PL2 -1.50 dB
PL12 15.65 dB
PL2W 11.35402870 W
PL12W 0.21885175 W
SFO2 400.1120007 MHz

F2 - Processing parameters
SI 131072
SF 161.9674970 MHz
WDW EM
SSB 0
LB 3.00 Hz
GB 0
PC 1.40

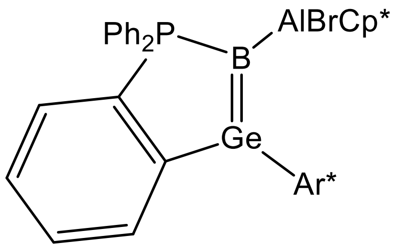

Figure SI60. $^{31}P\{^{1}H\}$ NMR spectrum of compound **13**.

NMR spectra of compound **14**.

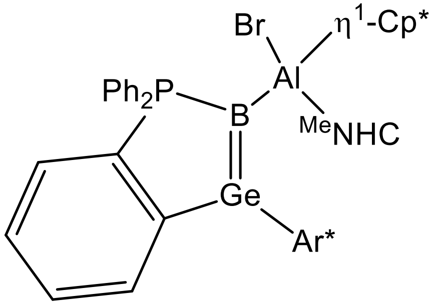

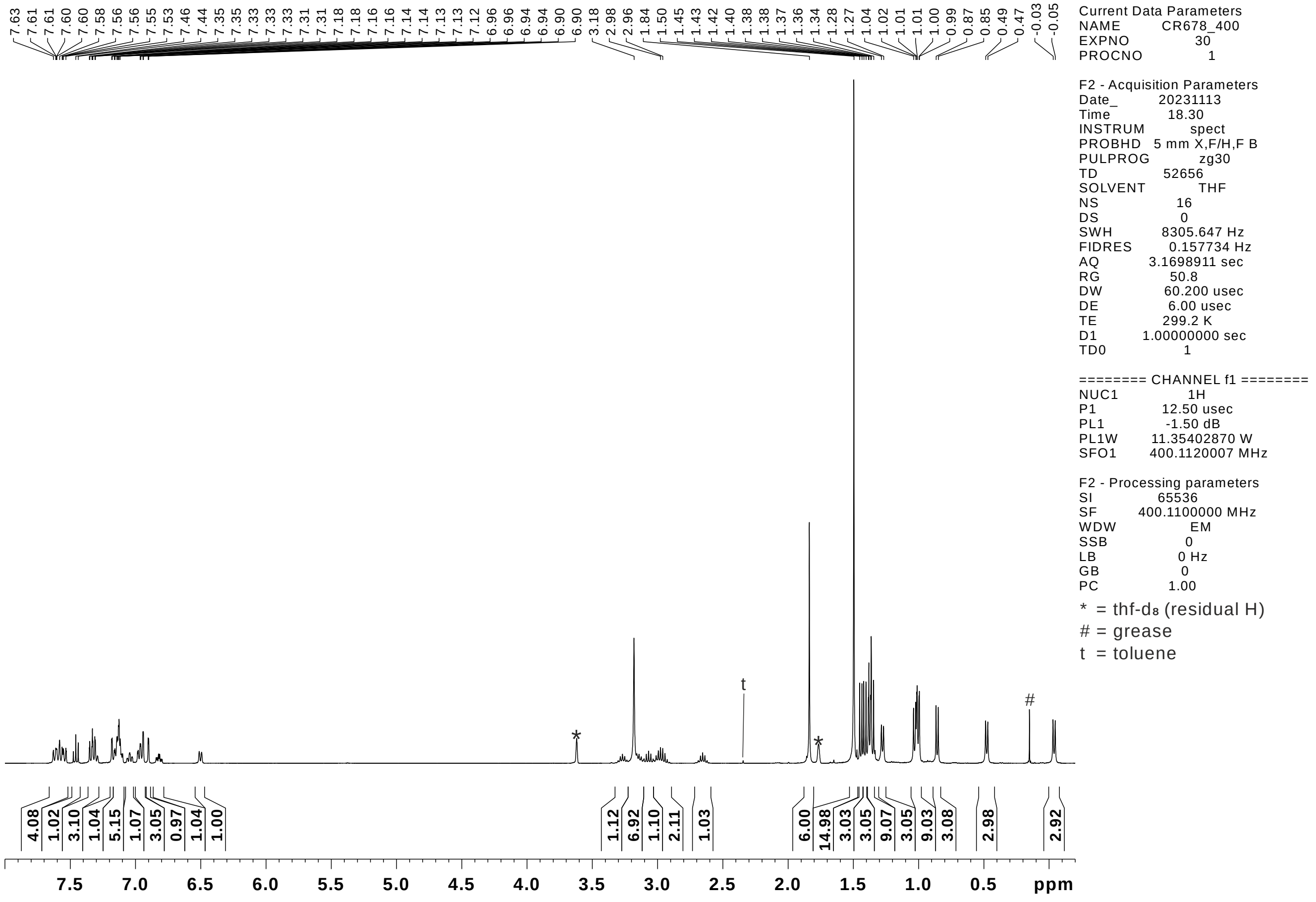

Figure SI61. ^{1}H NMR spectrum of compound **14**.

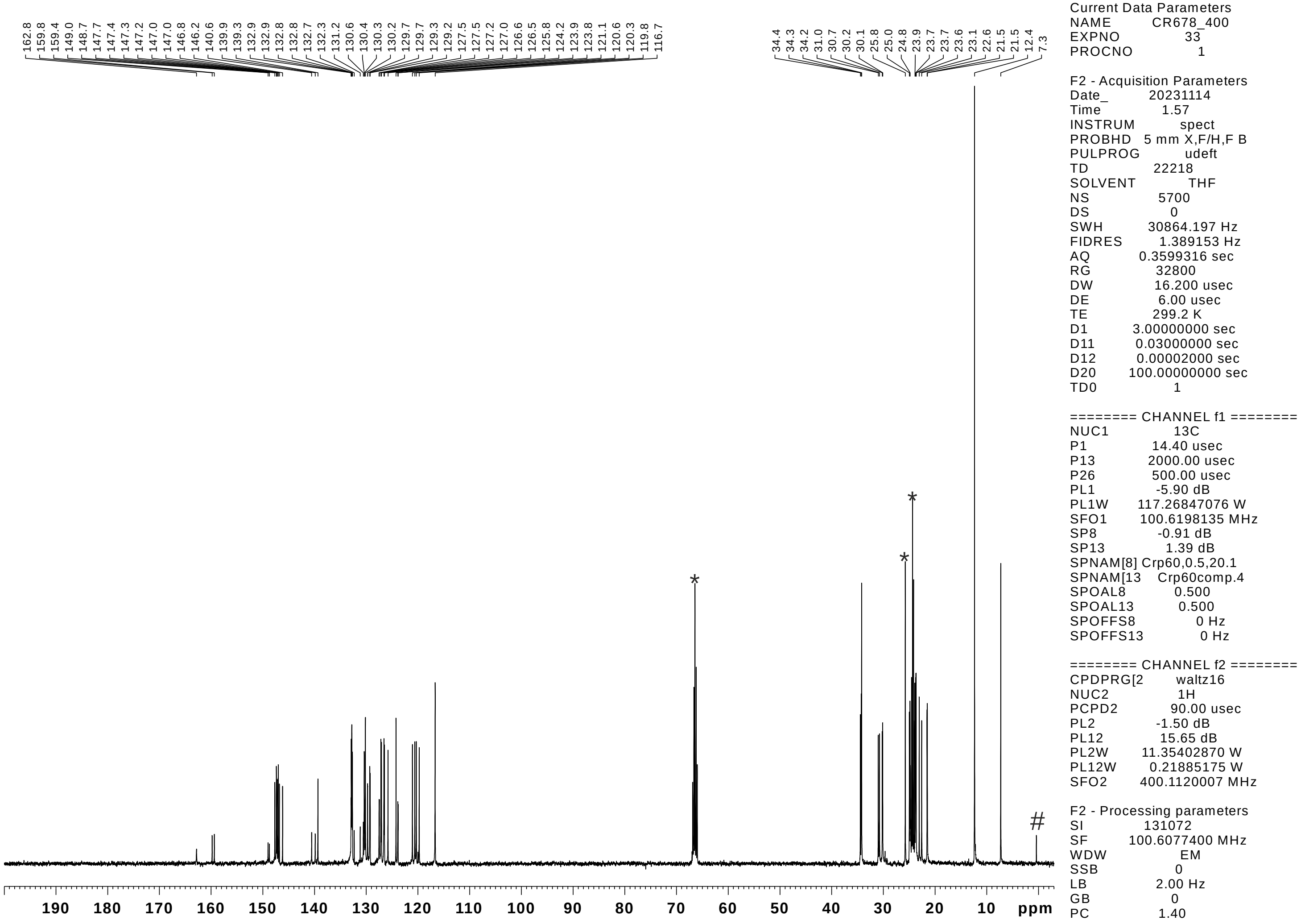

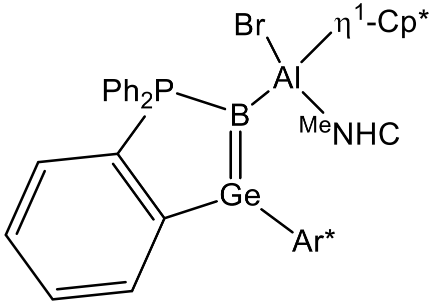

# = grease
* = thf-d8

Figure SI62. $^{13}C\{^{1}H\}$ NMR spectrum of compound **14**.

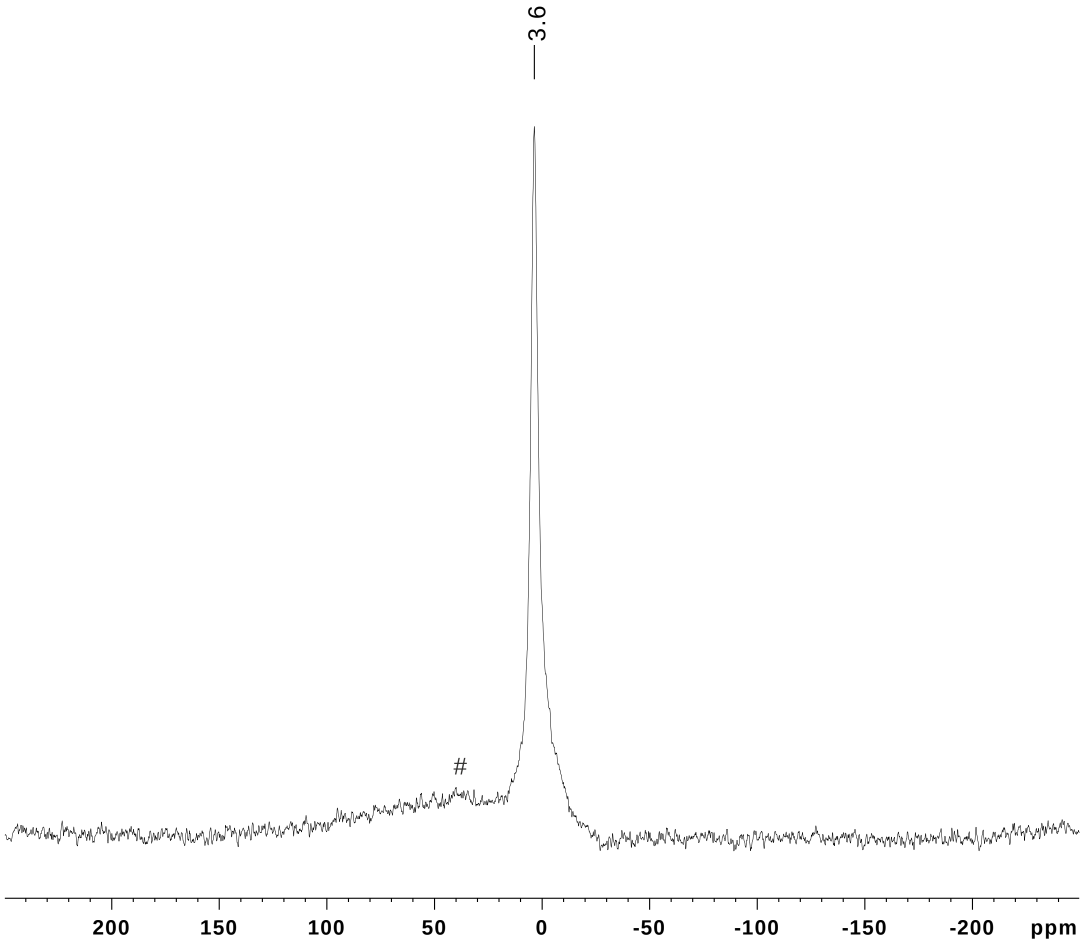

Current Data Parameters
NAME CR678_400
EXPNO 21
PROCNO 1

F2 - Acquisition Parameters
Date_ 20231113
Time 16.01
INSTRUM spect
PROBHD 5 mm X,F/H,F B
PULPROG zgbsig
TD 8192
SOLVENT THF
NS 1024
DS 0
SWH 64102.562 Hz
FIDRES 7.825020 Hz
AQ 0.0638976 sec
RG 2050
DW 7.800 usec
DE 6.00 usec
TE 299.2 K
D1 0.10000000 sec
D11 0.03000000 sec
TD0 1

======== CHANNEL f1 ========
NUC1 11B
P1 15.00 usec
P2 30.00 usec
PL1 -3.00 dB
SFO1 128.3711884 MHz

======== CHANNEL f2 ========
CPDPRG[2 garp
NUC2 1H
PCPD2 90.00 usec
PL2 -1.50 dB
PL12 15.65 dB
PL2W 11.35402870 W
PL12W 0.21885175 W
SFO2 400.1108002 MHz

F2 - Processing parameters
SI 8192
SF 128.3711880 MHz
WDW EM
SSB 0
LB 50.00 Hz
GB 0
PC 1.40

# = glass background signal

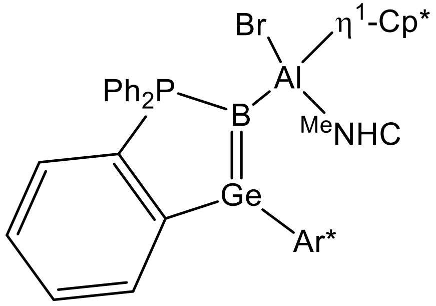

Figure SI63. $^{11}B\{^1H\}$ NMR spectrum of compound **14**.

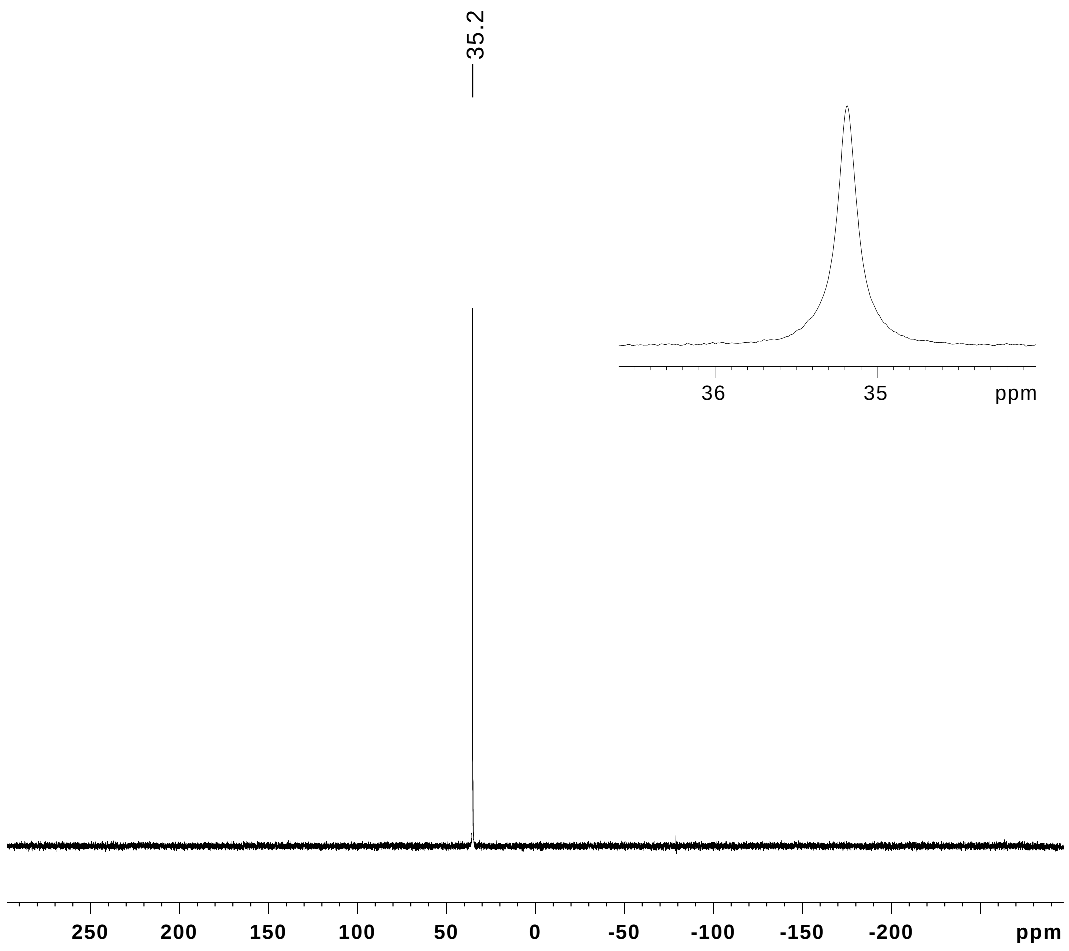

Current Data Parameters
NAME CR678_400
EXPNO 22
PROCNO 1

F2 - Acquisition Parameters
Date_ 20231113
Time 16.08
INSTRUM spect
PROBHD 5 mm X,F/H,F B
PULPROG zgig30b
TD 88150
SOLVENT THF
NS 256
DS 0
SWH 96153.844 Hz
FIDRES 1.090798 Hz
AQ 0.4583800 sec
RG 23100
DW 5.200 usec
DE 6.00 usec
TE 299.2 K
D1 1.00000000 sec
D11 0.03000000 sec
TD0 1

======== CHANNEL f1 ========
NUC1 31P
P1 14.50 usec
PL1 -4.00 dB
PL1W 56.78615952 W
SFO1 161.9674970 MHz

======== CHANNEL f2 ========
CPDPRG[2 waltz16
NUC2 1H
PCPD2 90.00 usec
PL2 -1.50 dB
PL12 15.65 dB
PL2W 11.35402870 W
PL12W 0.21885175 W
SFO2 400.1120007 MHz

F2 - Processing parameters
SI 131072
SF 161.9674970 MHz
WDW EM
SSB 0
LB 3.00 Hz
GB 0
PC 1.40

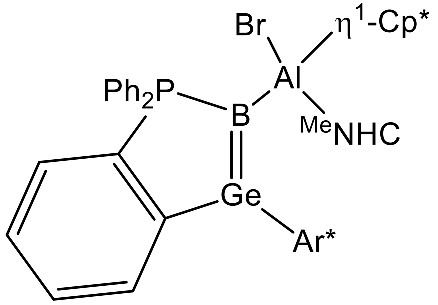

Figure SI64. $^{31}P\{^1H\}$ NMR spectrum of compound **14**.

Quantum chemical calculations

On the basis of the molecular structures of **2**-**6** and **10-14** determined in the solid state, the structures of the compounds were optimized using the programme Orca5.0.3[10-12] along with BP86,[13, 14] Grimme´s dispersion correction and Becke-Johnson damping (D3BJ) and ωB97X-D3BJ with suitable RI approximations (RI/ RIJCOSX).[15] The basis sets employed were def2-TZVP for Ge, B, P, Fe, Cu, Au, Al as implemented in ORCA5.0.3, and def2-SVP on all other elements.[16-19] For all calculations, tight or very tight convergence criteria were applied for optimisations and SCF convergence, respectively. All optimized structures were obtained without imaginary frequencies. With the BP86 functional for the structure of **4** the C-N-C angle shows a deviation in comparison to the solid-state structure. With the wB97X-D3 functional the optimization of **4** results to give a structure closely related to the solid-state structure. Plots were generated using ChemCraft.[20]

Table SI3. Selected results of DFT calculations of compounds **10**-**12**.

	**10** Ge-B-Fe	**11** Ge-B-Cu	**12** Ge-B-Au
Ge-B [Å]	2.02490	1.95526	1.97512
Ge-M [Å]	2.48139	2.45643	2.53527
B-M [Å]	2.30915	2.07624	2.18856
q [*e*] Ge, B, M	1.11, -0.57, -0.63	1.04, -0.87, 0.69	1.04, -0.54, 0.43
σ-donor, occ.	0.6423	1.6544	1.8953
Ge, B, M %	18.1, 32.3, 16,1, (12.6 C5)	32.0, 50.8, 9.0	28.0, 41.9, 25.0
π-acceptor, occ.	1.4992	1.8669	1.8291
Ge, B, M %	4.7, 4.0, 75.0	3.4, 2.3, 93.4	4.5, 3.2, 91.5

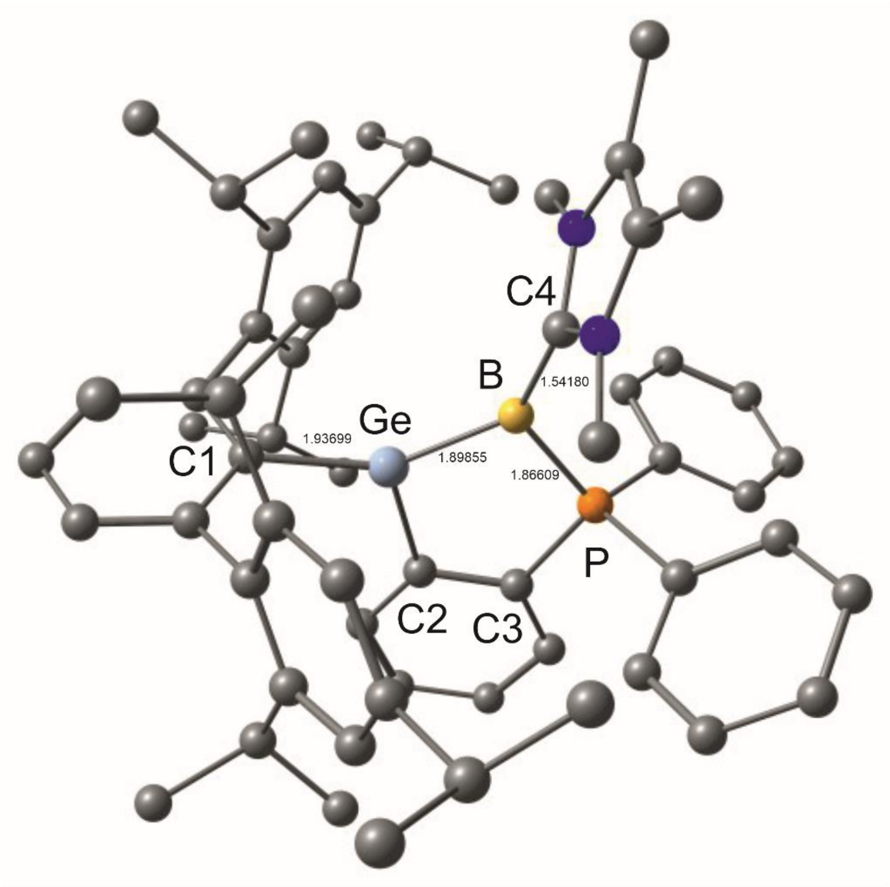

Figure SI65. Optimized structure of compound **2**.

Coordinates of compound **2**.

141

Coordinates from ORCA-job bp86optfreq_hnumac

Ge	4.81296271332917	9.67459191240911	13.48539842017036
P	3.94393637839571	12.41336042288949	12.88988018449563
N	4.37832081630372	10.68364823908215	9.56544462342950
N	6.42107223555539	11.23397515966275	10.04679695130622
C	6.67669842764679	6.20334778741139	14.96496928827131
H	7.66890006342324	5.92456454205708	15.35052478294876
C	5.63036145351135	5.26584360017566	14.95637770368393
H	5.79826600457670	4.25768623064214	15.36436890417652
C	6.95511793737933	15.08230229236177	13.55192601275232
H	8.02749174070505	15.01843394079294	13.78786762949373
C	8.07147308303895	7.39037735010474	11.99609185662951
H	7.13442320247242	6.86252519998196	12.26531023204369
C	5.20977910090201	10.96169160116614	10.60932130880219
C	6.32175206071096	16.33577201319463	13.47430787457993
H	6.90167316395792	17.25596211931428	13.64101258474763
C	7.13866807370936	9.48474321811163	16.58936698388287
H	6.15741990736304	8.99243551092652	16.42872776848602
C	6.36303879707374	11.11977967494406	8.65736453222708
C	5.05948379947672	10.77020874133779	8.35065125097317

C 2.36827909419313 13.54041622086284 10.85805193080990

H 3.26666944539007 14.09865784092584 10.55428965038007

C 4.38669884731200 5.58638314011263 14.38608451960241

H 3.59619686063653 4.82369868818575 14.32182707603316

C 3.55904088509389 11.93823091651502 14.59781235094903

C 6.21979843219027 13.90921332548631 13.33202165634609

H 6.70053479551189 12.92059467133504 13.40569123597968

C 6.47152431180209 7.49814267407802 14.44875983423397

C 5.19085855064375 7.83194632037905 13.94777241159950

C 6.87735417346272 10.89101541185028 17.14815975585713

H 6.37859241372765 11.54075546622501 16.40185910915077

H 7.81690129342957 11.38753583403022 17.46736786805096

H 6.22181497343451 10.83748767912161 18.03972156490375

C 3.97844005107343 10.63956823782514 14.97475479113440

C 4.15206018211332 6.87334985722155 13.86093831105814

C 2.97459538204015 10.32444058065571 9.70561579269997

H 2.32330435605625 11.17102996769301 9.41603067251477

H 2.78998696382188 10.06090920843789 10.76256234849942

H 2.74833575934312 9.44891025538415 9.07391307237985

C 4.84133219594085 13.98662627546734 13.03706136795019

C 2.38918561938305 12.73735668282304 12.01886898860499

C 1.19880643855221 13.61939622224981 10.08349828062496

H 1.18184764225927 14.25183772385977 9.18297821920721

C 7.85488630370842 9.49077643618219 15.24569685404187

C 7.55094324313237 11.33189692791493 7.78272872456383

H 7.26797783700650 11.23299072523182 6.71883516723254

H 8.34771355174448 10.58583397414628 7.98684957561250

H 7.99349770245105 12.33986179372671 7.92185434593881

C 10.67290544036327 11.47722680411420 13.52059276164160

H 11.07500988806917 11.27809307783401 12.50245550073906

C 8.84253578361629 10.45313306369916 14.97273538190191

H 9.04712473978618 11.22959944349040 15.72444891746384

C 9.58878365878263 10.43990862353905 13.77992483181094

C 4.40177173721225 10.48732156396149 7.04375923771607

H 5.10462918683216 10.66796567888435 6.21005343699728

H 3.51224446754241 11.13072606217887 6.88327765194786

H 4.06521604215171 9.43136242232005 6.97556012219062

C 4.94767602057961 16.41422602630978 13.19221321006769

H 4.44961597398606 17.39413770111006 13.14146320582177

C 2.73053381343973 12.37730209669124 16.83036774596268

H 2.24508408485244 13.04742686735707 17.55504397744589

C 11.83701285409978 11.33213079215794 14.51902996290581

H 12.25981317559379 10.30817575662649 14.49499049225545

H 11.50016187998116 11.53227170890154 15.55738329779612

H 12.65140428134980 12.04815806991186 14.28581801121896

C	9.31760036362257	9.42869325549919	12.84456604468465
H	9.90973688074999	9.39747605428805	11.91533023050649
C	7.58235500492848	8.49104097870314	14.27171230721181
C	2.94301139349389	12.81018017562946	15.50805889669187
H	2.63578918710618	13.82058655102409	15.19774523053559
C	8.31652700497450	8.46198676444022	13.05497449481821
C	9.20362670408759	6.34623696970311	12.00879728307716
H	9.00597365400868	5.53787777145730	11.27515949159090
H	9.30683449613434	5.88338934533919	13.01033219109660
H	10.17942374555016	6.80894062734357	11.75241841321059
C	4.16857421316249	6.39582809762820	10.98086514741868
H	5.04036379824569	6.54322064140692	11.65055348457703
C	4.20250913805998	15.24283259967165	12.97992720190155
H	3.12433725975849	15.30884503507049	12.77344846075998
C	3.14835799663327	11.09483818016128	17.22266041334805
H	2.98987972596399	10.76247453716652	18.25984074896038
C	4.52259577350694	7.04740830903420	9.63570009743691
H	5.42586613849278	6.57282869004855	9.20428451540425
H	4.73690965579297	8.12688188226528	9.75747529937206
H	3.71118854866336	6.93312891599991	8.88720624761996
C	7.60068809698010	11.61452322366276	10.80881244321752
H	8.48793042978241	11.08317852914669	10.42220802530295
H	7.43907949166938	11.31628218143602	11.86072804693704
H	7.76780795353075	12.70824465728874	10.74979397006801
C	7.86232177832197	7.98609090335791	10.59448505951059
H	8.76150523292592	8.52988573801833	10.23666376463270
H	7.00495675450186	8.68805715195714	10.58339009362621
H	7.65262747041415	7.18523071262243	9.85792520622391
C	2.91645393901832	7.21106947236450	13.07614888870768
C	3.76825176853923	10.23050009637327	16.30183723864760
H	4.08885189534700	9.22944255135485	16.62688403165037
C	7.91674581277375	8.61743389311365	17.60005037763107
H	8.04339172601555	7.58077906568954	17.23077235265674
H	7.38563228681610	8.57223555407261	18.57299327175388
H	8.92815142828234	9.03715367205110	17.77968790852760
C	0.05296856490605	12.89718021170300	10.45911326743745
H	-0.86242841166151	12.96272193313736	9.85232601152010
B	4.84929666129270	10.98099182404729	12.10825969413009
C	2.95428159663899	7.02384110235333	11.66319435285481
C	0.07732088835220	12.08985816003139	11.61184724940005
H	-0.81615015189856	11.52002328900601	11.90690111818357
C	1.24041947231719	12.00435102733005	12.38829952798799
H	1.26765974996610	11.35222716872938	13.27407019043095
C	1.81651638509003	7.34822189222565	10.90420922468588
H	1.83332793248258	7.16598964708973	9.81883128451362

C 10.10460152746003 12.90857201362591 13.52741916320007

H 10.89331915836751 13.65127902792881 13.28987531498203

H 9.68928524949871 13.16936702388854 14.52324753615559

H 9.29085527173476 13.02479484942314 12.78327783355699

C 3.95988069196451 4.87641026180086 10.82560652432330

H 3.09065487081546 4.66235770512138 10.16952944618800

H 3.76630358967722 4.39684341646295 11.80488903812679

H 4.85496580383060 4.39616595347979 10.37949880543181

C 0.65594538626697 7.88604368397492 11.48851399916644

C 1.74656275919982 7.73071112272074 13.69180343515236

C 0.64942186865548 8.07634440442842 12.87973524552968

H -0.25974015277424 8.48200563798906 13.34918438655434

C 1.63512528523092 7.83629586563819 15.20700600805726

H 2.66449145325496 7.96392080116557 15.59984309915090

C 0.79423378866151 9.03267796087317 15.67588845134524

H 1.13832971860442 9.98050674517114 15.21618489369815

H 0.86700035643126 9.14986993238118 16.77490104733580

H -0.28054700322571 8.89930687634671 15.43556814235336

C 1.08370622534122 6.51879920148781 15.79075536998518

H 0.06289760329950 6.31667387131785 15.40553610457291

H 1.02927238944926 6.57053808811650 16.89758537609067

H 1.72217046653549 5.65555385303894 15.52045104633912

C -0.58965123495112 8.17823209372935 10.66172935047009

C -0.30455306799139 9.05663055074050 9.43231410525139

H 0.15906565854398 10.01955852146043 9.72367241476110

H 0.37284855832294 8.54654545390297 8.71568888465071

H -1.24285803554056 9.28233010786976 8.88591778844343

C -1.28667480654135 6.86292880855682 10.25992809532756

H -0.63380534253022 6.25598394439900 9.59837904215280

H -1.52580115362479 6.24753879302187 11.14983908595170

H -2.23087878707117 7.06357434109430 9.71326005182157

H -1.28728530146451 8.74013218521307 11.32171827846039

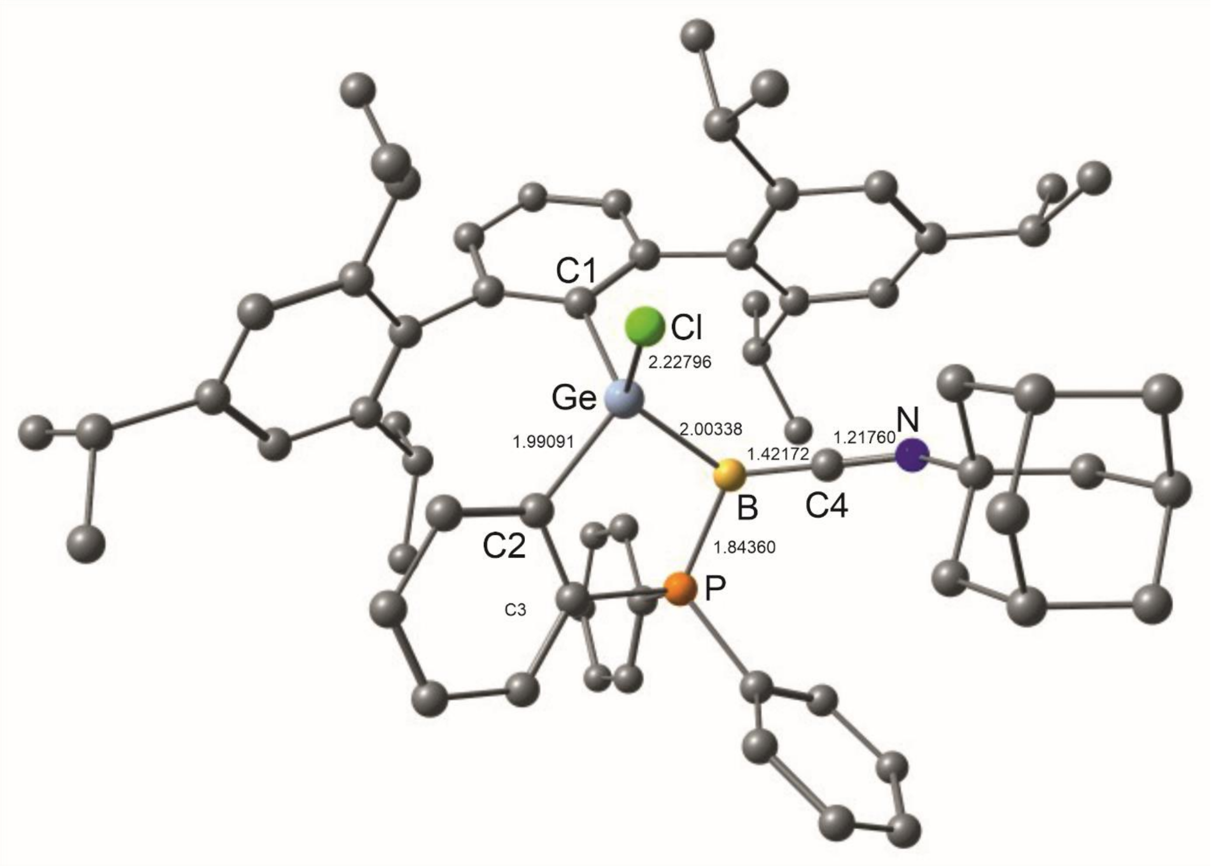

Figure SI66. Optimized structure of compound **3**.

Coordinates of compound **3**.

148

Coordinates from ORCA-job bp86optfreq_hnumac

Cl 11.28815623112800 15.64567799688683 10.12326257241064

P 11.29240499594519 13.40514472572996 14.31024992484482

N 14.82176660808500 13.79553546930149 12.67378797777196

C 10.37743650192260 12.37486147950015 10.05740732405916

B 12.22040929858400 13.35333543659121 12.71808712397143

C 11.42075099410796 11.55518844257550 9.53685496838258

C 11.08764753950178 10.42467232142005 8.75955318986702

H 11.90399268619874 9.81368708285945 8.34678601114629

C 9.75490461908366 10.09486227517327 8.48787028030159

H 9.51413728537688 9.20919301277230 7.88021972625028

C 8.73394364124933 10.92471356685001 8.96449399785388

H 7.68209990411021 10.71138497617084 8.72090160594932

C 9.02936287949222 12.06829928199872 9.73890383919072

C 12.88323243042096 11.85789405404958 9.69054290873481

C 13.49053101872653 12.81469104859633 8.83291235323245

C 14.88654067970443 13.00111570214418 8.90267613561528

H 15.34840935458172 13.73841579922338 8.23345408434736

C 15.69465744674418 12.26635817131688 9.78217999832648

C 15.06875452788181 11.32598299570257 10.61987081068454

H 15.68657327552863 10.76097067473591 11.33460508402787

C	17.74639741610493	13.66569871585188	9.12123081248654
H	17.59166963403315	13.58821429887291	8.02474033411807
H	18.83607555526086	13.77680353615773	9.29362081857875
H	17.25295336285297	14.59623989093997	9.46579580723068
C	17.20959146047149	12.43004250014867	9.85356232177479
H	17.45591453497835	12.54348470266921	10.93462649763373
C	13.62704127706174	10.03293722550315	12.91966497156614
H	14.69917350560015	9.75060173311179	12.95391114167252
H	13.07493746233745	9.30771256234197	13.55131726701075
H	13.52115170583284	11.04069340179413	13.36533238844350
C	13.27420898315875	8.61945826708026	10.85783572430880
H	12.82364814511200	8.55184575533377	9.84917524209863
H	12.81081785752492	7.83558847919058	11.49304805325313
H	14.35441144065570	8.38369704623977	10.75905218670485
C	6.29187033759225	14.66389282443633	9.39549276587815
H	5.99095119146149	15.45467498580536	8.69011438726106
C	7.50565504621461	13.97943320344897	9.18616323896523
C	7.86273683234952	12.94092551156661	10.08606071597975
C	12.68385002757335	13.54880945442809	7.76205055634465
H	11.66201877187056	13.70456637695924	8.16370975220286
C	13.08181128911989	10.01666032025424	11.48217501831338
H	11.98968195269633	10.21369424243965	11.52791771344490
C	13.68546730277706	11.09997959533960	10.59102929755662
C	4.09427488689844	16.00666514472712	11.85476154281798
H	4.87951221447849	16.78455863009491	11.76866467589323
H	4.27878658131321	15.44046897482845	12.79154669444046
H	3.11590601283317	16.52078604551824	11.95655112100337
C	2.93041778864873	14.07694952630728	10.67893863816326
H	1.95817149559053	14.61097513829165	10.71298913649060
H	2.98711402934778	13.43065946472058	11.57959628964976
H	2.93054989258158	13.41379039663199	9.79065273146163
C	8.38482711536148	14.31966219910696	7.98464700054559
H	9.40111118220500	13.93298094253287	8.20336913171506
C	7.88144158218586	13.60032964798807	6.71833851293944
H	8.54107981714405	13.81896994516706	5.85302483333089
H	6.85321632885844	13.92959822946367	6.45846276846957
H	7.86022725325466	12.50203342705246	6.86167510689307
Ge	10.86401920130892	13.81310176653331	11.31724636752192
C	8.52380724609550	15.83219732451769	7.75341570962608
H	9.25652556346628	16.02707520739420	6.94389641059556
H	8.89152371217796	16.34198916863819	8.66530784256392
H	7.56439159523260	16.29924846019957	7.44721230814438
C	9.49137154686371	14.53752725185425	12.56415714949472
C	8.38438023988243	15.33713148806675	12.23164478299999
H	8.13884471034170	15.51997124300298	11.17619431261787
C	7.58803772101306	15.90956056927400	13.23675947863617
H	6.73181468202901	16.53868816196984	12.95564692488441

C 7.86528244973337 15.67034284348786 14.59416485840724

H 7.22589940315747 16.10712686721013 15.37636630646150

C 8.96574464344551 14.87651636183352 14.95006236431755

H 9.19273470448650 14.68527724986257 16.00988477708120

C 9.78801419293415 14.34524457058856 13.93662411068459

C 10.66987758089893 11.80966209965015 14.93900502695864

C 10.26414372553622 11.62515306399573 16.27620247742239

H 10.40363761965953 12.43259183092393 17.01112591061743

C 9.68982946381495 10.40585189640056 16.66801657919433

H 9.37524414400603 10.25967061158647 17.71267552911439

C 9.51666827706319 9.37216057454793 15.72957700373863

H 9.06620429798732 8.41754229009697 16.04150815067944

C 9.92214364710040 9.55617607288056 14.39633261624906

H 9.79322072095978 8.74964484605304 13.65901487773280

C 10.49784391402547 10.77280592501899 14.00298231194171

H 10.82812958595273 10.93339806292715 12.96494899276239

C 12.19230711706551 14.17843824276108 15.67248551642244

C 13.18055797167639 13.41797940102599 16.33536229625561

H 13.28456160516944 12.34423491931791 16.11730235608629

C 14.03600547842602 14.03573430738225 17.26017226320972

H 14.81035738656342 13.44140097463783 17.76840945876321

C 13.90625003966208 15.40823174898163 17.53404644022450

H 14.57805156602603 15.89061362152511 18.26009919682878

C 13.24664731525480 14.93308586628928 7.40832447252716

H 14.20075813741792 14.86080718175632 6.84499421386321

H 13.41907056520022 15.54107266342812 8.31676027443311

H 12.52925325814430 15.48104814418189 6.76426679409217

C 12.92210339946797 16.16609800904137 16.87484685450399

H 12.82314577130919 17.24235107522761 17.08198537263652

C 12.07241885946481 15.55831102425705 15.93940163246173

H 11.32391650570580 16.15702631411206 15.40027772183999

C 13.62425975595819 13.57776304708527 12.70695408108658

C 15.38684266585667 15.16886773650559 12.68059244092474

C 16.90816335169148 15.10143224926036 12.89347611819903

H 17.36194098788780 14.47459789740084 12.09790286650261

H 17.12062808265754 14.60351798277141 13.86354791288886

C 17.49601040796496 16.52834398619656 12.87021249397199

H 18.59526117052328 16.47517970918659 13.02322875326479

C 7.05523703765122 12.66210786532852 11.22101316193947

C 12.55604272976377 12.68017234505923 6.49365581250780

H 11.95442910033328 13.20244535455520 5.72044396630553

H 12.06354175360415 11.71253183570210 6.70964897886134

H 13.55696610970753 12.46548414564909 6.06369335959989

C 16.85424067684555 17.36647463274227 13.99712963795818

H 17.28798429992342 18.39004807438968 14.00551486162539

H 17.07862000505514 16.91052387713655 14.98601522954231

C 15.32734499165656 17.43657456878830 13.78338132485903

H	14.86112018330464	18.03079327782537	14.59901794109302
C	14.74555536355808	16.01061185970971	13.80199911600566
H	14.92889679170263	15.52106778862150	14.78125196323301
H	13.64733251566842	16.02951005700575	13.64489465549301
C	15.07623256948061	15.83110601927386	11.31971198213232
H	13.97634453408718	15.85219123355191	11.17165399998644
H	15.49443971822329	15.20583872909631	10.50559001402885
C	15.02382176961252	18.09183709416904	12.41915800561584
H	15.41798151799400	19.13112221414698	12.39719455312934
H	13.92552908213562	18.15449359728322	12.26305251600889
C	15.66551574609093	17.25463579882811	11.29234807570931
H	15.44410635775849	17.71963654679691	10.30790304669805
C	17.19248297340096	17.18468610902830	11.50622130649655
H	17.66548099769606	16.59819243744842	10.68926535103481
H	17.63238503579790	18.20489597234394	11.46762737652247
C	7.09557012960500	11.98783764574651	13.68723683947355
H	7.37126213981471	13.02870071450310	13.93661178238151
H	7.60653835386223	11.31626845149133	14.40580292027873
H	6.00342806556643	11.87358734623433	13.85097600342335
C	7.49484207426452	11.62309242211352	12.24973829567727
H	8.60519839121249	11.59465278054863	12.21272818770327
C	6.98823947582193	10.21142652905341	11.89678012830603
H	5.87855716986203	10.19178466156704	11.86854306278348
H	7.32324698029134	9.47852281442695	12.66000160023988
H	7.36186786936586	9.87463506998288	10.91119472286146
C	4.10382213949163	15.07306906350973	10.63038120313147
H	3.96892512757225	15.70700355486027	9.72631439896101
C	5.85594224717877	13.37462949713689	11.38750310858940
H	5.22812801315384	13.15928960723413	12.26494538937379
C	5.43947195510357	14.35691162254362	10.46883011844569
C	17.91684179183518	11.15541664991705	9.34761038279477
H	17.69889736679020	10.99385434793749	8.27098030721958
H	17.57694869115326	10.25404274904444	9.89514964305256
H	19.01754397701253	11.23494427086722	9.46749084386233

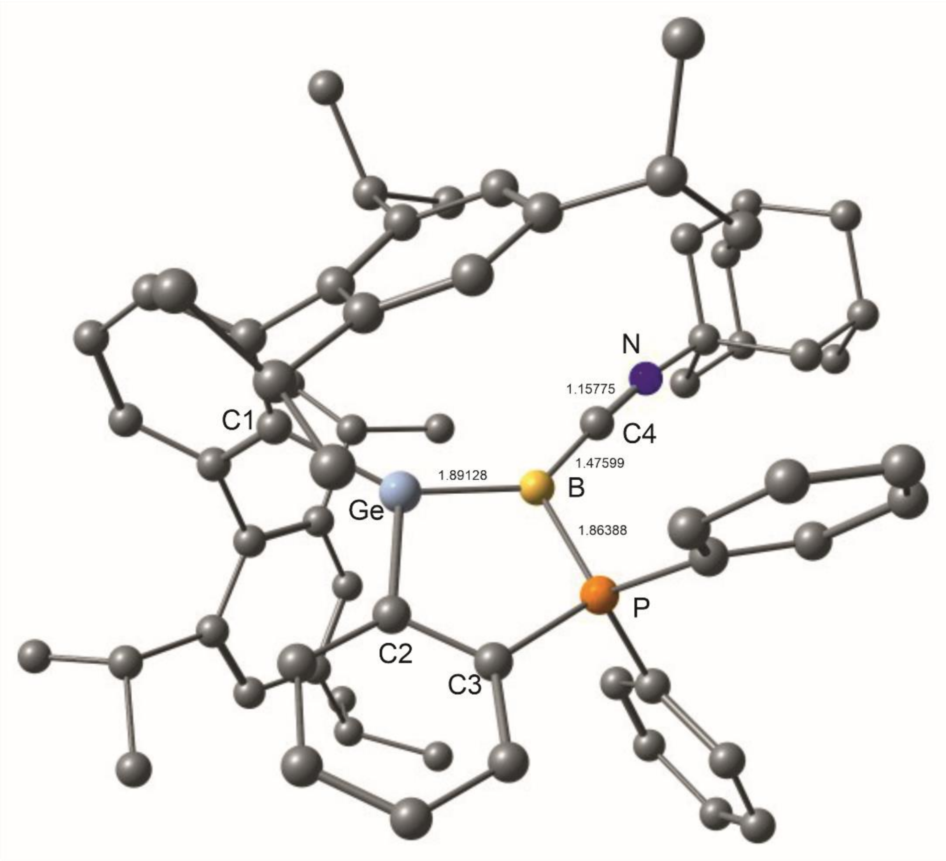

Figure SI67. Optimized structure of compound **4**.

Coordinates of compound **4**.

147

Coordinates from ORCA-job wB97X

P 6.75829247497397 4.51672478647336 3.96116488771189

N 4.15795408552511 4.16094640130417 6.78675009215106

C 6.47609144315289 9.02244762795816 5.86682985456499

C 7.44804410833268 9.54431995872885 6.73323067870845

C 7.34503761482506 10.89119441316666 7.09903385766034

H 8.07290941133848 11.31591415120792 7.79381051172127

C 6.31353395404589 11.68605553082897 6.60227775069044

H 6.25640348318286 12.73765788542597 6.89290879321638

C 5.32995123629647 11.14231793919378 5.77612274306094

H 4.49282868223014 11.75854960947760 5.43908048652660

C 5.38926913393100 9.79389972102438 5.42115971415278

C 4.29564571222537 9.03993447347602 4.72383110340205

C 3.30160093579143 8.44522739434899 5.53414277273639

C 2.43229338888700 7.52270555923495 4.95500697117607

H 1.67345173390530 7.04563350912635 5.58127469968643

C 2.51341326653895 7.17461791508158 3.60508763502384

C 3.45224908298679 7.83186200779641 2.81128639203831

H 3.48916712625376 7.59416291499830 1.74532477364078

Ge 6.61284403682527 7.21644860686775 5.20232572171708

C 4.34858349797975 8.76741220102707 3.34039416819336

C	5.31099800302612	9.52932679852089	2.43708100489405
H	6.21939467271196	9.73760517823946	3.02652148889680
C	5.74134736234009	8.76107600355022	1.18731496373043
H	4.91083322008770	8.62855717560887	0.47651941324716
H	6.14457527372154	7.76801263607924	1.43715398764194
H	6.53330292999122	9.31170042708440	0.65830175808774
C	4.69926690909905	10.88419470941480	2.05543770897515
H	5.39210192024062	11.46663816991192	1.42904466687920
H	4.46288425800236	11.48454506390256	2.94546137410286
H	3.76473778257466	10.74240861241312	1.49022495739275
C	1.60080672188524	6.10887724775440	3.02509294326579
H	1.88937908854270	5.98005786210517	1.96788417844349
C	0.13055750816903	6.53648182288708	3.05983293229529
H	-0.50874454223304	5.78235333569237	2.57576794636001
H	-0.01822603738673	7.49393132039170	2.53948892624575
H	-0.22669958360856	6.66022468008101	4.09480612189225
C	1.81691444701267	4.76292449493645	3.72438019009881
H	1.23208200204732	3.96648613594288	3.23901780055376
H	1.50446082964493	4.80865145686535	4.78052498967899
H	2.87789509445764	4.47105768715638	3.70135498369321
C	3.14380730976422	8.81599163673135	7.00511589680578
H	4.08641460276705	9.27019265242851	7.34493445637997
C	2.04582498727826	9.87468411989308	7.16592909177198
H	2.26847012154293	10.77429646058628	6.57355623293564
H	1.94297170168191	10.17800874172218	8.21911070187701
H	1.07271764112137	9.48635959215977	6.82502370921704
B	5.95709742844919	5.44966673721818	5.36179459796221
C	2.88665333448884	7.61080533983641	7.90911806478106
H	1.89947152840486	7.15791911361095	7.72843670232859
H	2.91432377447157	7.91336166063184	8.96721657969644
H	3.64952199011712	6.83166204026667	7.75861721108631
C	8.49742569162531	8.62771712403381	7.29084932299008
C	8.11670129328165	7.64580116640216	8.24317471103380
C	9.08688737466126	6.76529468416485	8.72099326269804
H	8.80013143317736	6.01242259214835	9.45770673046102
C	10.41799529354744	6.82430778395539	8.30294222852164
C	10.77006811968382	7.80630741375958	7.38236768754160
H	11.81159397303322	7.86447175716558	7.05512043262183
C	9.83980528714956	8.71363989710238	6.86495282394872
C	10.31842989437353	9.78635889691481	5.89438143939740
H	9.43445400102087	10.16349886352328	5.35393465119840
C	10.93155663080933	10.96950183927424	6.65579550342793
H	11.24014671022837	11.76433595589821	5.95976157675399
H	11.82077200206830	10.64955171786457	7.22159274104899
H	10.22494922851729	11.40574451339844	7.37535743731603
C	11.31118667114689	9.25454957221006	4.85471837138861
H	10.95536910661246	8.32596566497546	4.38545082881213

H	12.29652556602673	9.04818653731051	5.30010750270050
H	11.47179258146813	10.00282250630495	4.06312177795601
C	11.44777613093778	5.83368204600264	8.81694090261782
H	12.42731419412288	6.16632801191413	8.43389617379851
C	11.53004883787577	5.81534550729673	10.34596208925621
H	12.34991189683943	5.16257835470565	10.68214481700616
H	10.60081035350250	5.43213440179984	10.79754386010613
H	11.70932560437234	6.82261316123574	10.74999758607439
C	11.17934175342517	4.43489509759354	8.25117076892315
H	11.14280469519302	4.44860646094027	7.15113869333444
H	10.21329854101513	4.04790764925102	8.61652428937775
H	11.96213280191313	3.72467732031705	8.55959891268247
C	6.70348642668559	7.59130703412897	8.82192570054839
H	5.99758075825027	7.86346081388102	8.02239838641499
C	6.53902653539727	8.63886774559023	9.93067493611392
H	6.74933415908998	9.65294917555934	9.56143007568796
H	7.22761392408372	8.43467592837539	10.76551110236960
H	5.51108722572668	8.63012510534442	10.32544838636537
C	6.29109208634620	6.20527019518242	9.32030470704133
H	6.45918445907407	5.43249233256430	8.55350842875981
H	5.22076552313886	6.20616273596198	9.57833230669245
H	6.83715244936135	5.90877613635970	10.22917389400009
C	7.79689596060178	7.03784322317034	3.66977739871830
C	7.79525841370860	5.74450207922200	3.11889293843229
C	8.55211890235358	5.44566029452218	1.98639243666547
H	8.54815985531670	4.43999159167757	1.55830131419010
C	9.31571173486298	6.44990253371711	1.38988376316954
H	9.90708700815104	6.22478417047196	0.49976696063921
C	9.32508227769281	7.73570423194779	1.92876834608988
H	9.92552811447951	8.51819127652709	1.45878453576446
C	8.56905496051893	8.02924008246870	3.06509013893557
H	8.57764193289484	9.04206043948551	3.47716973892431
C	7.82475611876889	3.13747906554937	4.44567498510886
C	8.20207027189726	2.14313668521441	3.53179270582694
H	7.80649964078857	2.14858870119894	2.51273761112611
C	9.07462381606700	1.13205768198174	3.92323621320575
H	9.36704896367828	0.36028137792301	3.20782483507272
C	9.57100809155936	1.10386081695553	5.22703084388259
H	10.25364685576805	0.30757475054899	5.53288313448690
C	9.19787162471614	2.08943264674209	6.13870924374157
H	9.58806922486278	2.06860706338409	7.15831969958488
C	8.32937227065576	3.10796677770911	5.75086387899215
H	8.04572607432531	3.89262976530597	6.45836515189232
C	5.57796488745198	3.86104279230947	2.75666018329373
C	5.15478530441579	4.66277617448918	1.69026599759746
H	5.59608691200258	5.64982304727276	1.53834402370789
C	4.16792035137988	4.20514888102045	0.82138091980315

H	3.85142262228967	4.83253468464421	-0.01511602910572
C	3.59141740286319	2.95044061552957	1.01594960345115
H	2.82055921397126	2.59210034872269	0.32991854198828
C	4.00061868115677	2.15261771264710	2.08400169176524
H	3.54921411256061	1.16996707273586	2.23797720219475
C	4.98940448216326	2.60505082203461	2.95506682193812
H	5.30799293560825	1.97058941947060	3.78663728283742
C	4.94430668213609	4.76351051944753	6.18762730079111
C	3.35999390322946	3.28908797325792	7.59995662546212
C	2.07863473988820	4.01785459011193	8.03578357834569
H	1.52754450365571	4.35375047780532	7.14255615649076
H	2.34685469961158	4.91460075591715	8.61491612430134
C	1.22519779856386	3.06475253725930	8.88386202566557
H	0.30948061988084	3.59238504502333	9.19318598297535
C	2.02396298320759	2.63816178756643	10.12475589263979
H	2.27498169129290	3.51970709431500	10.73837175631848
H	1.41147045483803	1.97653456426092	10.75818874552315
C	3.30477329515422	1.91154183780653	9.68762172305704
H	3.88310232871392	1.60809834712513	10.57437610121774
C	4.16506730387866	2.86080169693227	8.83988930556799
H	4.44837488568388	3.75467137671808	9.41794231026417
H	5.09765252463055	2.36826517318385	8.51868526163609
C	2.99685141827832	2.05019748582922	6.76029287858968
H	3.92352000218783	1.55042369927081	6.43298916698264
H	2.45565991266506	2.36775578174354	5.85425084485175
C	2.14035153718995	1.10372138247109	7.61493365034077
H	1.88267263683987	0.21933063646064	7.01125627293846
C	2.93723319130073	0.67325928091460	8.85604996111325
H	3.84829564225913	0.12858735029772	8.55691954711474
H	2.33804228432664	-0.02334260689024	9.46425322067699
C	0.85793600615785	1.82856154885234	8.05026208110283
H	0.22367108132644	1.14962257603384	8.64266165036775
H	0.26677094914325	2.12469104090042	7.16752248575499

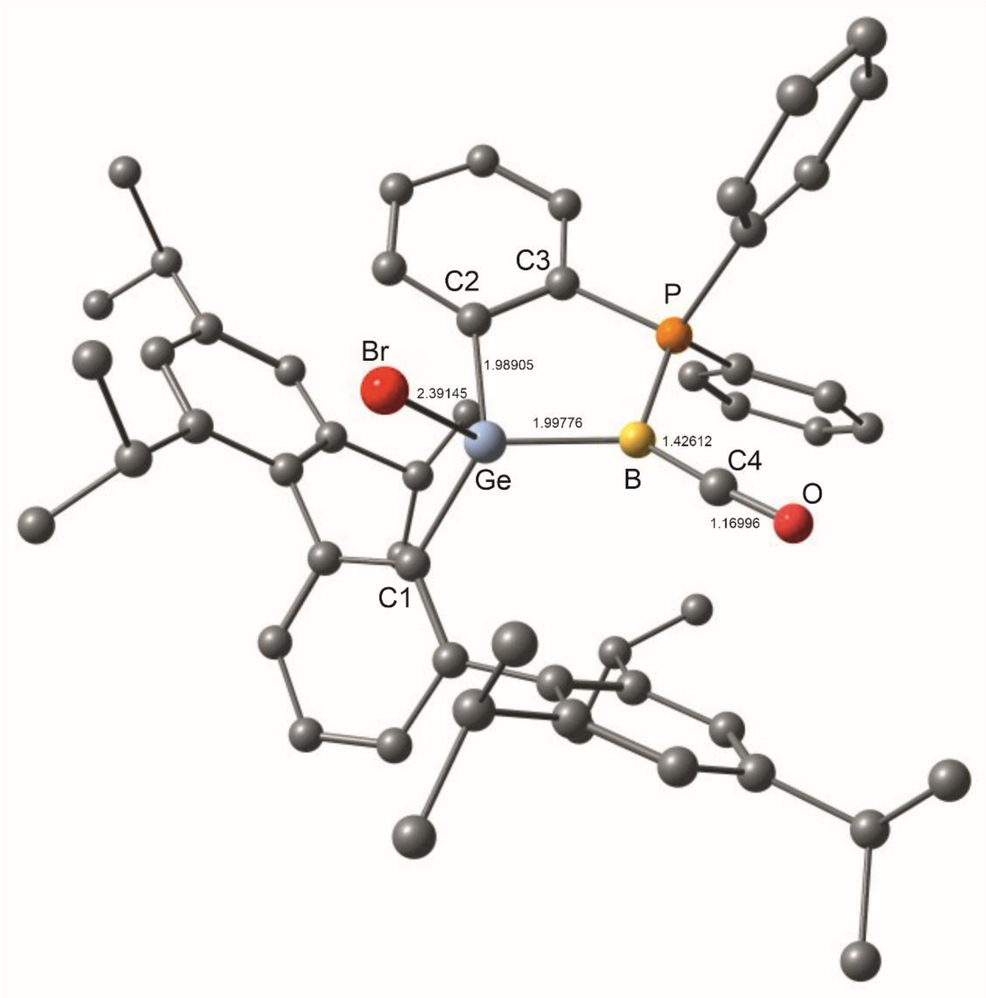

Figure SI68. Optimized structure of compound **5**.

Coordinates of compound **5**.

123

Coordinates from ORCA-job bp86optfreq_hnumac

Ge	8.39011966193191	11.31978555611721	4.08195161213207
Br	10.71315194668353	11.88073443883334	4.17073454003249
P	6.66077110373234	11.44925625909456	6.63185581765842
O	6.90578663975409	15.03651769950891	5.54208071940677
C	7.66065569838049	12.34691877185781	0.01868547613992
H	7.31063385986888	13.25508542859548	-0.49483047499824
C	8.04578774001800	11.22072566277264	-0.71543621692838
H	8.01995458561290	11.23586421430715	-1.81565990764192
C	7.14398900878035	13.59399099933947	2.06568090247956
C	5.76160823051695	13.65098093303293	2.39739368782927
C	7.33779354360809	15.98411982192724	2.47131613744026
H	7.96123593855260	16.89034645463711	2.51957524706281
C	5.20022295435149	14.89766445091136	2.72198219443992
H	4.13013850322031	14.95904352894405	2.97347323607380
C	7.19018099734913	11.97124226489244	8.29661775399750
C	8.42906549688755	10.06281359857806	-0.03109034445012
H	8.68109076492396	9.14618945814166	-0.58500755824998

C 3.82368062241616 12.37762598875943 3.46514701661129

H 4.26858249303264 12.59327731190332 4.45501080742943

H 3.33755860171131 11.38262438353505 3.51947250847521

H 3.02293194110031 13.12192785600647 3.27490047240777

C 4.89793794296462 12.39158572179344 2.36927492807889

H 5.57573455987821 11.53456565127281 2.56864824372615

C 9.44606636474628 14.70199590201483 1.86335647823151

H 9.73758394577201 13.63137279189562 1.83324174291585

C 4.27247416697443 12.17126277280583 0.97797955033343

H 3.60450852469195 13.01746155070498 0.71277911748023

H 3.66910709856590 11.23958911778131 0.96063332438235

H 5.04766734610787 12.08953958289049 0.19163833522669

C 5.96231455983141 16.07973247909202 2.73834899196579

C 7.94771707957078 14.75842774156193 2.14416027678029

C 9.78388936981659 15.31668815430002 0.49241301140898

H 9.25198532508958 14.79290420531261 -0.32677236567388

H 10.87298831314696 15.25256649283151 0.28818977918939

H 9.49230530838914 16.38745799333828 0.45265661701354

C 9.48582288848344 6.07360675287937 2.80606817020607

C 7.68584398208103 12.34584762091960 1.43164347813356

C 8.13506633057939 11.18950700978084 2.12490895094920

C 5.30593281166955 17.42217605211171 3.03302610404703

H 4.25377659392762 17.20764839354798 3.32459217379376

C 5.97415603786109 18.15977277984456 4.20671548919354

H 5.98994612742592 17.53129000654338 5.11798853506929

H 5.43542355687129 19.10253639724952 4.43674862018129

H 7.02439498806790 18.42854518988629 3.96821853844466

C 10.26124644672891 15.35781612745387 2.99085303308566

H 10.08131649224249 16.45177352990324 3.04796350215285

H 11.34685784803044 15.20474838545024 2.82284401381288

H 10.00786368246167 14.91313723674532 3.97317492786955

C 8.16999639825123 6.56274829662345 2.89055201407438

H 7.39242140508673 5.89930218365843 3.29758569221560

C 8.48466101621379 10.03241707643107 1.37980549213297

C 10.48451546192874 6.94134675988407 2.33413315716018

H 11.52668096364148 6.59108870533792 2.28715961464432

C 4.86418349432771 11.22441016737384 6.76675053477529

C 5.26881597581865 18.30013891783667 1.76660243633459

H 6.29674670533428 18.54367823589579 1.42483548272385

H 4.74095036364046 19.25769840409934 1.95901964325498

H 4.75397157210783 17.78156053814825 0.93270175249158

C 9.79754522194104 4.63453245058010 3.19636060364821

H 8.92949596268063 4.26865197560014 3.78876285370406

C 11.32353756467093 9.16392897431876 1.44129162682868

H 10.92448399098710 10.19917488027993 1.41793816710580

C 11.77174909720117 8.79194461497598 0.01497586709559

H 12.16107487630622 7.75246384067076 -0.01996588122791

H	12.57956901448667	9.46907430238991	-0.33194170380826
H	10.93591219967237	8.86181097298204	-0.70825975256951
C	8.42506119209134	12.64396782850145	8.39822848971465
H	8.98606507646393	12.87320411321530	7.47802311666841
C	8.32711020440276	9.71929140506368	5.26125512292769
C	10.19403983524726	8.25702568442784	1.92353902887418
C	7.82857742161123	7.86846618392887	2.49914999980973
C	6.45124000018439	11.66993511350512	9.45902408115268
H	5.48441369958662	11.15086915112275	9.37859947886510
C	6.39093526796169	8.36835025482032	2.63336353537940
H	6.44551071080375	9.44787509068513	2.89207617755888
C	4.07585650846833	12.31655613805131	7.19228603865391
H	4.55930106803257	13.24639173894228	7.53012212926522
C	7.30076788595615	8.80277185890436	7.30675454688112
H	6.61162140030562	8.91767162392025	8.15739788804290
C	2.67781322131689	12.22242680270170	7.17066451272927
H	2.06782235267273	13.07762801325447	7.49888402140834
C	7.44025769285020	9.83671611687659	6.35928489560840
C	8.85039840965934	8.71252618279805	1.98152802316405
C	8.91790042894996	13.01119778040004	9.65889810733386
H	9.88179825868507	13.53654264847917	9.73643469666520
C	12.51948681528867	9.16095078082351	2.40957148812826
H	12.19979949887388	9.41770839867497	3.43838003408379
H	13.26973348376779	9.91443036740478	2.09396952583862
H	13.03094947826048	8.17584919755838	2.43421318483146
C	5.60988985887022	7.67887816966957	3.76023047737193
H	5.39448463528664	6.61541403253293	3.52683658502287
H	4.63222270853720	8.18199727241650	3.90144217112110
H	6.16561252198427	7.71207138245331	4.71904288492692
C	11.05175434541986	4.50661277444986	4.07685830710641
H	11.19018735452641	3.45922886423383	4.41494804322750
H	10.98609836662484	5.15204570798699	4.97627061717698
H	11.96992631015765	4.79710563645050	3.52560699420567
C	5.63175450544418	8.26711162857496	1.29592526651094
H	6.14057554550596	8.84048388870915	0.49749390773071
H	4.60217181793238	8.66804001661244	1.39904470731303
H	5.55977402853181	7.20925118912180	0.96719508027931
C	9.13333725465367	8.57101145234918	5.18286666866211
H	9.87393405642763	8.47527944044746	4.37855473830332
C	6.94758333764533	12.04484666926585	10.71741166532617
H	6.36784226968561	11.81569070178763	11.62467782622597
C	8.17952597094781	12.71426880084966	10.81804487237413
H	8.56458526573558	13.00969604274356	11.80610113626400
C	9.90910330779725	3.75018373238853	1.93793131830471
H	8.98731404441070	3.80788667034251	1.32501742246752
H	10.08112158501172	2.68729724060033	2.20799568749102
H	10.75547612551788	4.08207127507363	1.30064031141352

C 9.00420493032965 7.53923286367872 6.12685438428336

H 9.63874406195863 6.64500217624299 6.03632886980627

C 7.05775658919542 13.89092839483892 5.35952749085910

C 2.05633891703117 11.04215545673664 6.72331868796872

H 0.95819075198697 10.97262941779587 6.70230310385035

C 8.07219514072058 7.63876813794306 7.17390894958321

H 7.96853991999372 6.82364415558615 7.90606632725317

C 4.23991713598270 10.04365599689132 6.32134478907846

H 4.85305263254437 9.20011262131859 5.97662059806927

C 2.83902977255441 9.95498442999577 6.30118109900980

H 2.35736314418427 9.02977367592174 5.94963405649880

B 7.23845356671352 12.48245259123168 5.22778503539608

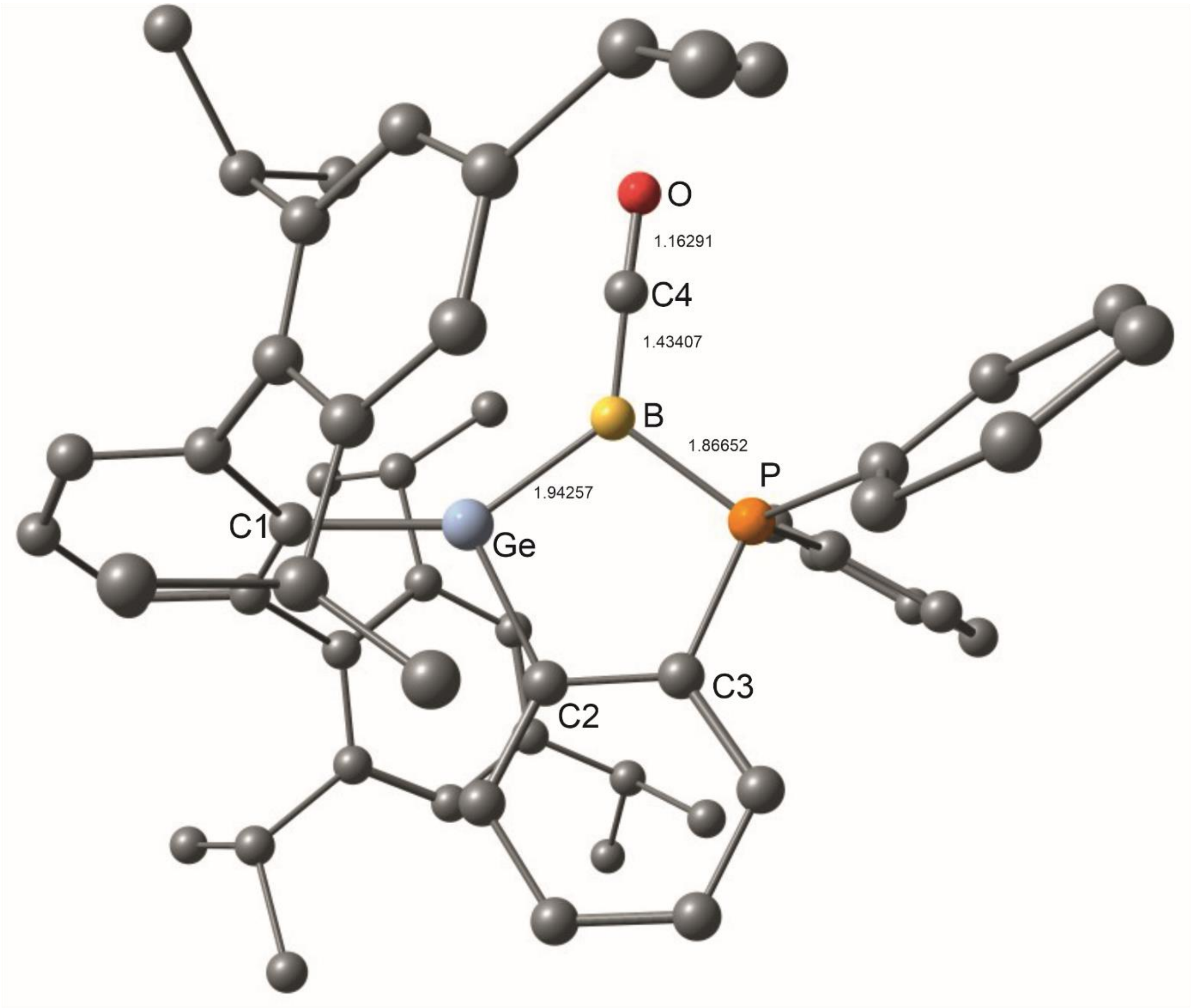

Figure SI69. Optimized structure of compound **6**.

Coordinates of compound **6**.

122

Coordinates from ORCA-job bp86optfreq_hnumac

C -3.44873261680661 18.39252149854298 8.85844574364726

C -5.19135727400420 17.25258488919203 10.11694862537998

H -5.81701381699256 16.36177534226328 10.27761703292192

C -5.33541616917114 18.35511750338483 10.98112577449560

H -6.05685957535666 18.30733404562270 11.80840800961656

C -4.54280671461086 19.50485039000031 10.79589509944845

C -2.37924981732968 18.57847186410383 7.82760258693809

C -1.08429022459392 18.04651565445913 8.06966413049915

C -0.02862787875544 18.44923547901478 7.23501425277064

H 0.98185192463048 18.05202120305676 7.42302528565174

C -0.21248321026705 19.34335069531712 6.16299919848994

Ge -2.58246245337577 21.07965707298633 9.38435150499244

P -0.96879304429066 23.51326607957021 8.59432847373997

C -1.50893743722555 19.82827857350443 5.92210833652298

H -1.67804224829925 20.49913342609119 5.07052231013289

C -2.60403965545794 19.46595661300156 6.73071816931549

C -0.81755457870332 17.09982477335591 9.23627179475852

H -1.79965962957717 16.81417021650627 9.66348565479347

C -0.12082349821272 15.80510049601647 8.78271902386212

H 0.89808958319590 16.00147523974312 8.39002057719197

H -0.69690110087691 15.29497059090803 7.98501159574665

H -0.01524450709821 15.10277749962017 9.63420126979402

C -0.02790689092263 17.80701840878162 10.35214801768013

H 0.98649628080858 18.09537744880393 10.01096563484224

H 0.08493970653185 17.14756968823407 11.23637980856241

H -0.54482830593233 18.73047981327275 10.68333347602595

C 0.98898227521170 19.75840296523852 5.32233574540464

H 1.61448944551790 18.84605339438367 5.19916641629360

C 1.83549213826258 20.79782086441802 6.08537772263888

H 2.72394076493645 21.09645138434677 5.49296117836624

H 2.19020905848041 20.40069514973893 7.05620590534074

H 1.23816329050634 21.70785844930700 6.28997451030098

C -4.02266799443015 19.90090948163438 6.36215224845095

H -4.57923565603877 20.07886318396039 7.30722383526989

C -4.74225805644750 18.74636983245798 5.63309468507209

H -5.78647876166999 19.02598018220094 5.38483086134261

H -4.77162404176963 17.83158721109305 6.25659652236875

H -4.21752848286804 18.49544835929434 4.68854100493848

O 1.56019241573922 20.70839190145442 9.64828739955026

C -3.61380156568024 19.48469769975753 9.72925899817057

B -0.77186030137401 21.75919574976191 9.20125743456474

C -4.08745557278880 21.19257524368362 5.53625310553695

H -3.67385192090134 21.05163424000940 4.51673565784729

H -3.53445815640913 22.01938152450793 6.02444803167449

H -5.13906773781563 21.51995595048316 5.41855175650502

C -4.55473588854703 20.78940898502948 11.56045672974779

C -3.30757978443723 21.23106018356595 12.12263230592889

C -3.17482325287841 22.58887612526305 12.48271786841694

H -2.22949927723009 22.94165874992300 12.91712470187602

C -4.22478021758484 23.50742240639446 12.32657386914243

C -5.47260040969079 23.01469737648198 11.89970123294994

H	-6.32044524138740	23.71021856279249	11.82760708022539
C	-5.67047946201452	21.67132755251286	11.53082859088275
C	-2.26573268689514	20.19746830263976	12.57445070267129
H	-2.14960019479030	19.44160309582663	11.77044369978730
C	-0.87815247749895	20.77637893094543	12.86858945475477
H	-0.16483907803875	19.95935134602042	13.09208549914480
H	-0.88690854218332	21.45160330347670	13.74846225517937
H	-0.48149464934549	21.33734285995303	11.99883013135993
C	-2.82323553510942	19.44788709994773	13.80383171577372
H	-3.78346113376953	18.94755227856171	13.56970032182206
H	-3.00063706016380	20.15053814277575	14.64373755090754
H	-2.10657162727165	18.67408597900382	14.14601097505011
C	-4.00606039146198	24.98763893063273	12.59796858983416
H	-3.03020130237339	25.07711206146635	13.12364802503254
C	-3.88143198890999	25.74908553243610	11.26355083105444
H	-3.05248943490117	25.34659753732330	10.65105195138523
H	-3.68755732837341	26.82675859790668	11.43846502406299
H	-4.81254548008767	25.65859614722061	10.66621798718494
C	-5.08950436702784	25.59933038529479	13.50133642658162
H	-4.84165296271566	26.65168166928763	13.74660847505900
H	-5.19062679040004	25.03907107662085	14.45226554365029
H	-6.08137382809562	25.60662458799593	13.00365566833134
C	-7.05910645428201	21.16700256781256	11.15139391244182
H	-6.92590577195763	20.35380990498079	10.40460995315978
C	-7.72990263623462	20.55264808142815	12.40112992167974
H	-8.70257433704931	20.08813133011529	12.14063516684589
H	-7.91702131107732	21.33861905250525	13.16147931640460
H	-7.09317576094364	19.78371776580985	12.88049840496397
C	-7.98492398252009	22.22792266434555	10.53651875602946
H	-7.51915149739471	22.74628809843423	9.67659593052149
H	-8.27465690360577	23.00086332961713	11.27735251002862
H	-8.92256232433250	21.75498202908889	10.18330721661766
C	-3.57819297954007	22.60463568462015	8.70585331786424
C	-4.96643258493595	22.68693852193768	8.51341400803648
H	-5.60767264793252	21.83952644124576	8.79294087794949
C	-5.52780809766915	23.83913974282287	7.93805697599251
H	-6.61466591380869	23.89886647662020	7.77643143470281
C	-4.70669212671354	24.91472084985149	7.55649942142937
H	-5.15098267289704	25.81295244959961	7.10282081571408
C	-3.31599262291744	24.84558303852032	7.74418336652696
H	-2.67737058262771	25.68613133646856	7.43632410476100
C	-2.75865523213128	23.68807968411294	8.31427708757373
C	-0.51647201430996	24.78656658540999	9.79945459512486
C	-0.35586663763649	24.41222553197342	11.14780648569124
H	-0.40355992635251	23.34752919562496	11.41699979741314
C	-0.14673111260958	25.39630040236851	12.12569203696440
H	-0.01595519565023	25.10302046039094	13.17800742671363

C	-0.09980513986968	26.75152903107518	11.75900702796832
H	0.06190413169235	27.52331055434127	12.52637215809292
C	-0.24530290629724	27.12506735156134	10.41025130600928
H	-0.19424115468680	28.18591993902799	10.12333586168494
C	-0.45121003556577	26.14717833993356	9.42732707018948
H	-0.55299876695401	26.44160462096831	8.37219585861554
C	-0.12763601313232	23.91733826481251	7.05002775341572
C	-0.76216939468845	23.66899742919234	5.81459745009405
H	-1.79324800577646	23.28880488101959	5.78983875794635
C	-0.06953407573853	23.90854007353424	4.61917584599023
H	-0.56837615097498	23.73096957013018	3.65503809384777
C	1.25536011086900	24.37778705498666	4.65182835556722
H	1.79557188908196	24.56184526695633	3.71130064791824
C	1.89123126294231	24.61193703678809	5.88294990735884
H	2.92893902484743	24.97597614239085	5.90876410024063
C	1.20344587626956	24.38584553949034	7.08459762844662
H	1.69977510023789	24.57591171592294	8.04814246801034
C	0.50954389257417	21.16088462494488	9.43912164900736
C	-4.26284013222961	17.26107645709369	9.06023399554640
H	-4.16576807761231	16.38805236732058	8.39769695518281
C	0.62188376577870	20.26388768209760	3.92171991507372
H	1.53815370178778	20.44324573958185	3.32485431600473
H	-0.00731154513254	19.53558975708664	3.37175223127493
H	0.07381362012369	21.22669960264811	3.97094908110507

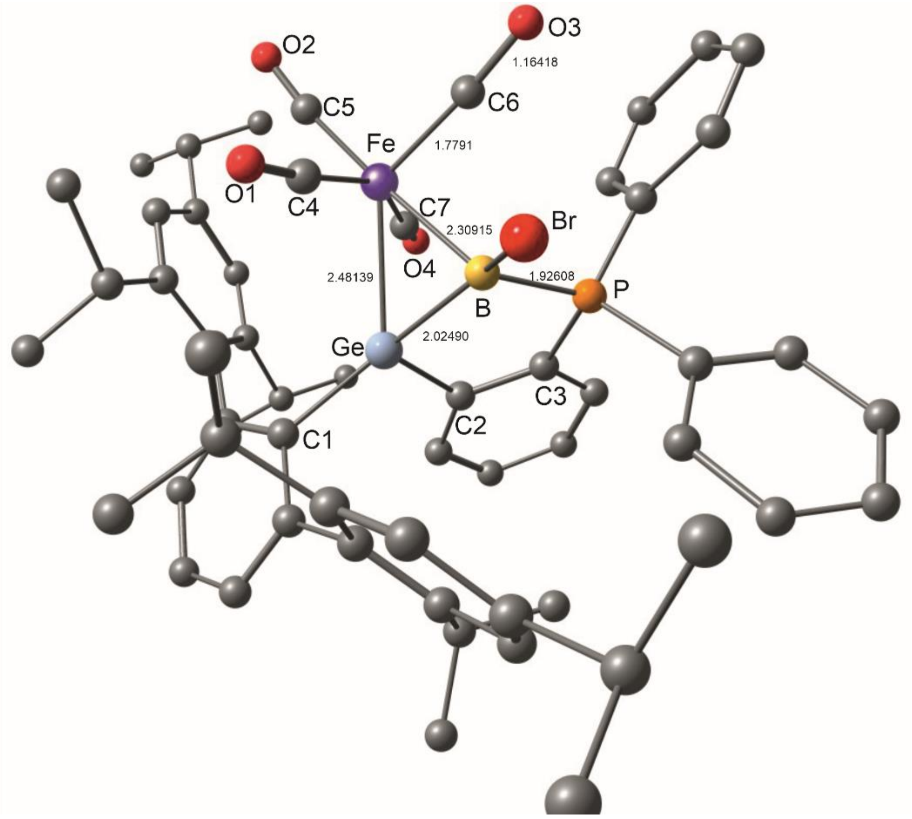

Figure SI70. Optimized structure of compound **10**.

Coordinates of compound **10**.

130

Coordinates from ORCA-job bp86optfreq_hnumac

Br 0.56838003704364 19.59768603160963 12.62090763485107

Ge 0.87708911376169 16.03624236220041 11.73498404072398

Fe 1.72754381524681 17.41192486870792 9.85308762346248

P 3.14761101220444 17.66271670353552 13.03395558713847

O 3.17813792822604 19.96745687703398 9.91849995374445

O 3.73745605180843 15.24892613511823 9.86912502443855

C -1.37537152240265 14.65806360390425 12.88966960241900

O 1.83412810550848 17.28614547000449 6.92142277687076

C -2.17895219699581 13.52188015292254 13.12476125324276

H -2.79004704477175 13.48431261760966 14.03894929907278

O -1.00714660892428 18.51433780920210 9.65405266966838

C -2.20940700858597 12.46586076802770 12.20593753104911

H -2.82783560161761 11.57710793105333 12.40339513814177

C -1.47953930876887 12.56429211574631 11.01456063595413

H -1.53777261338950 11.76281771071760 10.26316033038890

C -0.67035081324144 13.68913029170366 10.73841527965984

C 0.02558745330668 13.76060699847579 9.41142014685680

C 1.16292596205175 12.95375238379256 9.13814964825599

C 1.81411042575265 13.08710961866977 7.89802262093774

H 2.71796703788349 12.48863058480893 7.70928822583886

C 1.34328153604481 13.95146153733423 6.90121140197371

C 0.15931365998585 14.66485386094006 7.15572223166995

H -0.23106424245603 15.31733742978831 6.36278179831185

C -0.50926047664979 14.60021263590199 8.38876332827848

C -1.81263377374465 15.37426661458165 8.59268967566926

H -1.75697763218698 15.85014354722798 9.59562612224856

C -3.03310946678074 14.42903355521381 8.60097971765105

H -3.11200257917924 13.89380639680222 7.63174292402685

H -3.96820359479899 15.00731089644512 8.75059044840916

H -2.97286151012951 13.67375824536935 9.40561291655947

C -2.04876782705379 16.48167975246281 7.55518890475058

H -1.17252610632110 17.14393907001059 7.42867930601837

H -2.90462305523290 17.11383051625588 7.86359089778894

H -2.29462550335059 16.05360411951082 6.56098049341363

C 1.65967253812729 11.88166254959066 10.10317882478745

H 1.17122121394859 12.06140879705383 11.08087574080775

C 1.20663516524767 10.48760282152633 9.62245106718076

H 0.10600455424861 10.43141122612825 9.50620577865300

H 1.51792750714865 9.70016252506591 10.34047856291339

H 1.65567574027669 10.24720750364645 8.63624182007339

C 3.18199943021350 11.91236037958805 10.31681480335992

H 3.72614173575960 11.60894941026611 9.39835277283534

H 3.47567837379666 11.20328888478165 11.11792623503760

H	3.53362754870714	12.92178836928758	10.59788529416778
C	-1.41741708504404	15.78323695735383	13.87774467329370
C	-0.84364414077156	15.62730447843505	15.16698096018731
C	-1.01289938945608	16.65574597006833	16.11521804416580
H	-0.58377740001204	16.53033742494343	17.12221522134939
C	-1.69090863982011	17.84461459495521	15.81069457216774
C	-2.22935016635741	17.98649138597476	14.51853796088778
H	-2.75723420025790	18.91499162161818	14.26316860809677
C	-2.12716847941610	16.97867906037393	13.54765529133338
C	-2.82473510360430	17.14334334043193	12.19647160953981
H	-2.12976249053718	16.76258175749379	11.41786144017030
C	-4.10139483069855	16.27970499097450	12.12377955479344
H	-4.58534533424255	16.39073235394522	11.13165234427492
H	-4.82890830218831	16.59974454828590	12.89876937685698
H	-3.88837866974655	15.20529988802294	12.27730218226744
C	-3.15880902739378	18.59774232781249	11.84192491425425
H	-2.27164452651320	19.25446949940854	11.92184842470084
H	-3.95809324556705	19.00267593860544	12.49768499270086
H	-3.52717594782443	18.65598727989441	10.79942856263446
C	-0.05640662905802	14.37843385956926	15.55592399896345
H	0.10222452598920	13.78806254113918	14.63063943615605
C	-0.85269905905813	13.50081204406609	16.54026816519247
H	-1.83387638043916	13.20281584854682	16.12033625447600
H	-1.04744712230563	14.04333917948771	17.48913210788825
H	-0.29095895879060	12.57635318632376	16.78862948916117
C	1.33252797505173	14.71829728551244	16.12278737456221
H	1.90844513770617	13.79129854362977	16.31815086773524
H	1.26467569118740	15.27804305145523	17.07835431739446
H	1.91876269387337	15.33432089589908	15.41538122914781
C	-1.85022343463155	18.95358657886074	16.84190501643025
H	-1.25092085956017	18.65690953089588	17.73251037646378
C	-3.31683022106683	19.08007797700852	17.29759863067638
H	-3.97006993372909	19.37024663026977	16.44838976419824
H	-3.42428007183250	19.85424759183339	18.08566486318689
H	-3.69737080322165	18.11962155179803	17.69971172873530
C	-1.30383260235391	20.29839439163388	16.32798764551836
H	-0.25259138708479	20.20916617315041	15.98950153793517
H	-1.35161260513575	21.07301639649342	17.12101304351688
H	-1.89647548029034	20.66778918883789	15.46588348018783
C	2.32625171832183	15.06720393262994	12.67044921075208
C	3.38730088948557	15.86938120566080	13.15244799680173
C	4.46903765670669	15.31201228346361	13.85732041204948
H	5.28087923759660	15.95445197079661	14.23118069511531
C	4.48428577351018	13.93163583594678	14.11515066366506
H	5.32200670113241	13.48612937620119	14.67224194134404
C	3.40876906280232	13.13207644734256	13.69219765280488
H	3.40111798108489	12.05610524687869	13.92463176119500

C	2.34146564267581	13.69651402953285	12.97287138579896
H	1.50337914505704	13.06300830907011	12.65009957684590
C	3.12598703287349	18.20473194210081	14.76571072562403
C	4.31611762226573	18.57078499715058	15.43028541313263
H	5.26455105711384	18.62099119117499	14.87639410901529
C	4.28720351709224	18.87852934643142	16.79848654133539
H	5.21641996313406	19.16757137222759	17.31260569654122
C	3.07518628289044	18.81760293995498	17.50714681614443
H	3.05186244366045	19.06105597137752	18.58033386176688
C	1.89352738245265	18.44537059216382	16.84583497855037
H	0.94471569115739	18.39151927769420	17.39734026250066
C	1.91218682081344	18.13926198832593	15.47740589199453
H	0.98519288187033	17.84416046236718	14.96702470001534
C	4.62030308314754	18.41473422225848	12.31296026044104
C	5.49978244239822	17.66304213088544	11.50773018153098
H	5.34244972832380	16.58339287496208	11.37736656429995
C	6.56871215263774	18.30242163249324	10.86181701389607
H	7.25170630334789	17.71427966443072	10.23053681796952
C	6.76368767219408	19.68500834943626	11.01773638138172
H	7.60168947408062	20.18317186820801	10.50690894560982
C	5.88548200871415	20.43448456611419	11.81968379491819
H	6.02876384379574	21.51949184914093	11.93255133191319
C	4.81212545258285	19.80569640748174	12.46375542493939
H	4.10902975169750	20.39517902759479	13.07076402283017
C	2.63749530374572	18.93670710372991	9.94258900491241
C	2.91116127162355	16.07414138890902	9.94960672041943
C	1.75882689402303	17.25891904565908	8.08297924200759
C	0.04753846116909	18.03583958108607	9.76381312223837
C	-0.59188161288648	14.72520701006188	11.70862557556920
B	1.49052278038343	17.93301913257455	12.09015148177599
C	2.06606594956148	14.09581464566748	5.56959855729853
H	1.60854114497234	14.96587252425712	5.05050046359170
C	1.84958593634122	12.85094922885547	4.68721107358983
C	3.56295690576456	14.39997846724131	5.75580310851140
H	3.71292181294219	15.29070366146056	6.39651966659941
H	4.09442744230672	13.54932751154806	6.23113641607230
H	4.05038454225871	14.59026606176577	4.77735821070140
H	2.28357685024430	11.94750071348691	5.16524324093032
H	2.33299274184647	12.97479637195219	3.69560897386360
H	0.77023408357032	12.65676727260942	4.52420777768848

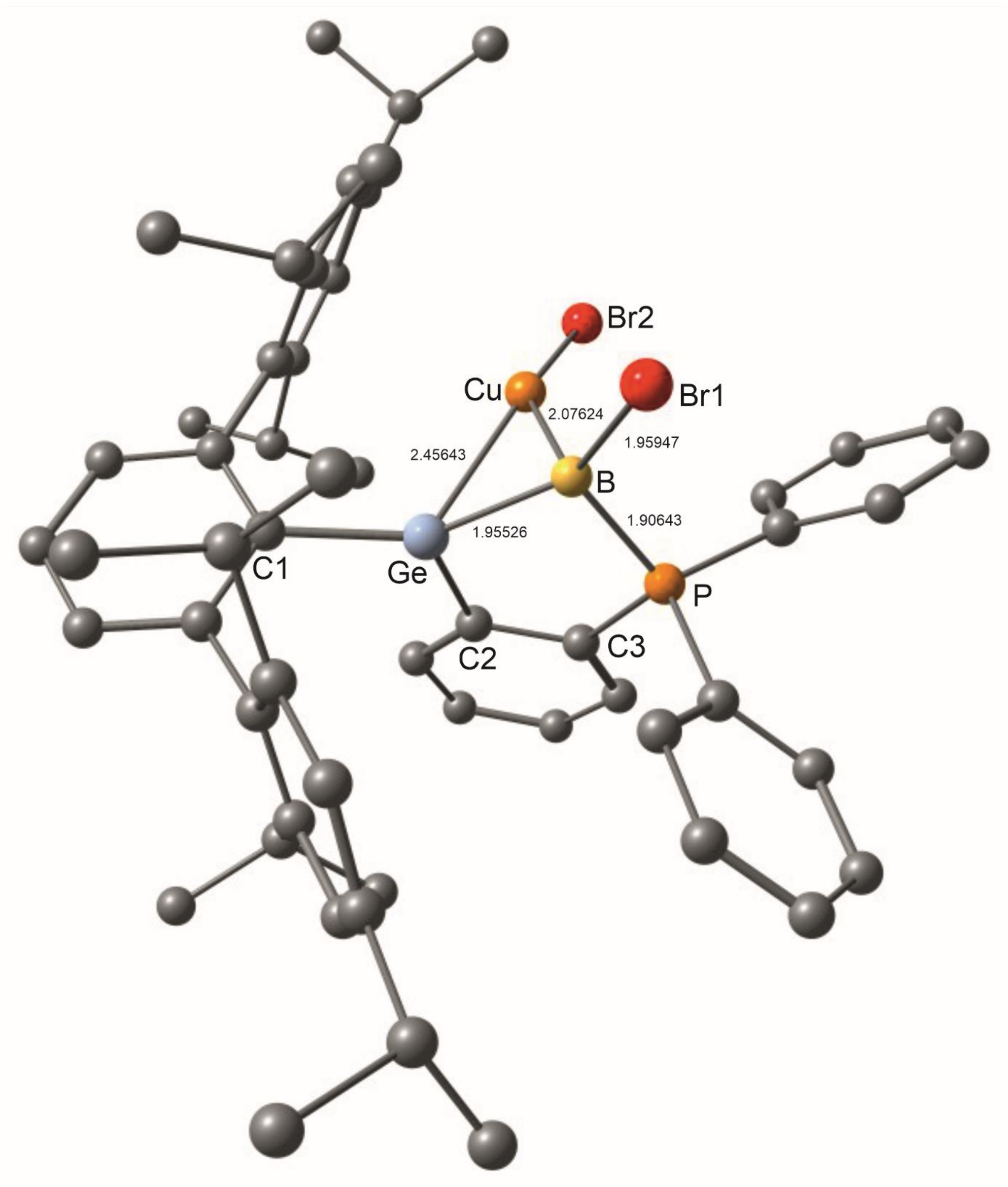

Figure SI71. Optimized structure of compound **11**.

Coordinates of compound **11**.

123

Coordinates from ORCA-job bp86optfreq_hnumac

Ge 2.25685260456348 6.89464170415967 11.88001016411649

Cu 3.47447728452337 8.85801934011980 12.71462013274384

Br 5.12277055048088 10.42641052879249 12.60160106059973

B 2.79708854977087 7.18267595037331 13.73695111394647

P 4.39359956978919 6.14201488436934 13.78857230061691

C 0.92451380738524 7.11876670260850 10.49389591285158

Br	1.90702991615295	7.43370937201732	15.46446464103729
C	0.20672186138343	5.96501560688249	10.09587652602554
C	-0.63811040675761	6.04918282699763	8.97151396575311
H	-1.21176000491094	5.16254894209251	8.66179492378905
C	-0.76532690893552	7.25980138101216	8.27008562170065
H	-1.42321653571502	7.31564459989322	7.38932181587199
C	-0.09145921866992	8.40783682365474	8.71077394438414
H	-0.23183532015969	9.36902788477883	8.19356860811761
C	0.75778741410843	8.35833742265755	9.83911400652067
C	1.39473203751185	9.61058083912355	10.35369988789202
C	0.91233813861625	10.20757661677792	11.56073783412569
C	1.43582686471183	11.45398133426676	11.94699665418195
H	1.07676801043885	11.91264730915713	12.87653345492401
C	2.41555852676358	12.12441439105324	11.19374906795023
C	2.89969081892038	11.49740003678015	10.03670970265820
H	3.71268150895280	11.98389875953227	9.47509083172785
C	2.42336200196654	10.24633813596362	9.61001273068766
C	4.59339140394999	9.41266358194578	8.63195008712728
H	5.04632395098206	8.79126902121435	7.83167330765194
H	4.81079107209142	8.94121106260856	9.61023613304484
H	5.10612403080674	10.39616563071783	8.62460368796827
C	2.76610711007567	10.29734837255975	7.09332659647121
H	3.23457653615518	9.77797341283165	6.23143416847896
H	3.15564100460184	11.33659221617448	7.11288702248690
H	1.67444439144530	10.35380603148935	6.90789668663968
C	3.07676672675313	9.56651569456672	8.41155330963743
H	2.64346839681427	8.54687350713132	8.33641150913238
C	2.95481079179459	13.48654519291202	11.60526286926065
H	3.99305667942745	13.54113226204820	11.21047470845932
C	2.13320577873041	14.60633231169062	10.93330415612110
H	2.54971498886041	15.60657849393363	11.17493236358765
H	1.07968131270962	14.58097421566485	11.28370241746181
H	2.12095477805882	14.49371031799539	9.83015863479930
C	-0.18093716949381	9.54439711992503	12.40173489141143
H	0.04922784312782	8.45844253425808	12.44528047561666
C	3.02919454716263	13.67956718392200	13.12673826971905
H	3.55046250495408	14.62771378286528	13.37068264491874
H	3.58080999402580	12.84398789028055	13.60200591978348
H	2.01893459603883	13.73830347511485	13.58397710149575
C	-1.56564116559435	9.68261282458470	11.73943241617673
H	-2.34125012960066	9.17592162034714	12.35033636217945
H	-1.58587857503318	9.23623549668964	10.72681318962395
H	-1.84644735213511	10.75240945829496	11.64691317254680
C	-0.22326761572201	10.03773661080997	13.85335289019184
H	-0.90643240069081	9.40050287192011	14.44903439471961
H	-0.59608233788199	11.08062802130333	13.92454734900181
H	0.77568661616808	9.99173862668230	14.32904009175769

C	0.25603825955083	4.71774433266635	10.92809242883894
C	-0.56420270188168	4.65110209177586	12.09274903495615
C	-0.60433164719685	3.45179757181045	12.82366918552789
H	-1.25144338501605	3.38966099917680	13.71347203044069
C	0.12911303237312	2.31341221588077	12.43749196881628
C	0.97418230161653	2.42381132608369	11.32279421583307
H	1.58497278491571	1.56174776037306	11.02171488563857
C	1.05649702170724	3.60547594005640	10.56106718185099
C	1.96920600502394	3.65180910427408	9.34149163591346
H	2.13097707730365	4.72207888553456	9.09851124488185
C	1.28980887757574	3.00151982535607	8.12054090520371
H	1.94452936712350	3.05894564424303	7.22615280875671
H	0.33252956821244	3.50241120812978	7.87611383168976
H	1.06993080131146	1.93092266003980	8.31552011419052
C	3.34422875972057	3.01955053290922	9.61356011997846
H	4.03069531213462	3.20316917223667	8.76303884258528
H	3.27149437835884	1.91993002071945	9.74498386614558
H	3.81197023079072	3.44367290960936	10.52363287675368
C	-1.45648677017548	5.82182978022903	12.49513382842850
H	-1.05773714678311	6.72839410160402	11.99879154083295
C	-1.45163852093108	6.09747138704300	14.00503594259928
H	-2.04033505400998	7.01029779282958	14.22921392638953
H	-0.42267791687191	6.26010310894546	14.38349677819316
H	-1.90560390243520	5.26763164969704	14.58562975360370
C	-2.88424793979022	5.61033660223465	11.95495380030291
H	-3.52904398724795	6.48070700387884	12.19587544915842
H	-3.34668022765157	4.70472801769908	12.40074510191481
H	-2.88036167281941	5.48172352724125	10.85402227129997
C	3.95661990640162	6.28511568103756	11.09932782580553
C	4.29560677565090	6.20289475733473	9.73986814502682
H	3.56248123144075	6.50090763717438	8.97492790534062
C	5.57188380157635	5.75326872040971	9.35464804666545
H	5.83131378777019	5.69838984740665	8.28624916419170
C	6.51682214656648	5.37386900039216	10.32237788438651
H	7.51061603450058	5.01608782129495	10.01435619801633
C	6.19836373944395	5.46268050820396	11.69082703341727
H	6.94199103974414	5.18533548429431	12.45322713689103
C	4.92919152718911	5.92637494218313	12.06736178808156
C	4.09310093497688	4.48374235886875	14.46449461377407
C	2.77613009968263	3.98272705243125	14.47741743043965
H	1.94982058933403	4.59273952994006	14.08277854795314
C	2.52626986617688	2.70459896946802	14.99689178542513
H	1.49437614837941	2.32938136209748	15.02521253593771
C	3.58332295684757	1.91721687715888	15.48198222101039
H	3.38042872247972	0.91525917725091	15.89008146804172
C	4.90062046848192	2.40882717366058	15.44940033539228
H	5.73125609897229	1.79210843973274	15.82498747039438

C	5.15706432477931	3.69340715901603	14.94879935660525
H	6.18336875116660	4.08993544915744	14.94578962212483
C	5.73190709596529	6.85875015271153	14.75811514568645
C	6.69533273318613	7.69018244156017	14.15007251611577
H	6.69011370305765	7.84443607076414	13.06279926853025
C	7.64029077655873	8.35333003583002	14.94655404185586
H	8.38162648915026	9.01026185074375	14.46848647661320
C	7.62247895217135	8.19922675225315	16.34210639347080
H	8.36315594727557	8.72633222653491	16.96274616415030
C	6.65142663147748	7.38354714102311	16.95030814349889
H	6.62782091690694	7.27229351362993	18.04486683672859
C	5.70329482612789	6.71502527351279	16.16390859365199
H	4.93601103792700	6.08559935654058	16.63814543798134
C	-0.07532593535544	0.99571315329808	13.17778536195602
H	-0.18947425843901	1.24440105194796	14.25810955112708
C	1.09215319042865	0.00932054634634	13.03840084968286
C	-1.39960574196461	0.34503958377337	12.72323178833646
H	-2.25817880036433	1.03082562338543	12.86718026821058
H	-1.35314527189686	0.08925836419303	11.64411522293356
H	-1.60173334627619	-0.58754959698331	13.29004156553257
H	1.17746167162184	-0.37123629438107	11.99932135636415
H	0.93646674203156	-0.87137852250180	13.69383211861651
H	2.06035820046621	0.47594042347766	13.30809409161187

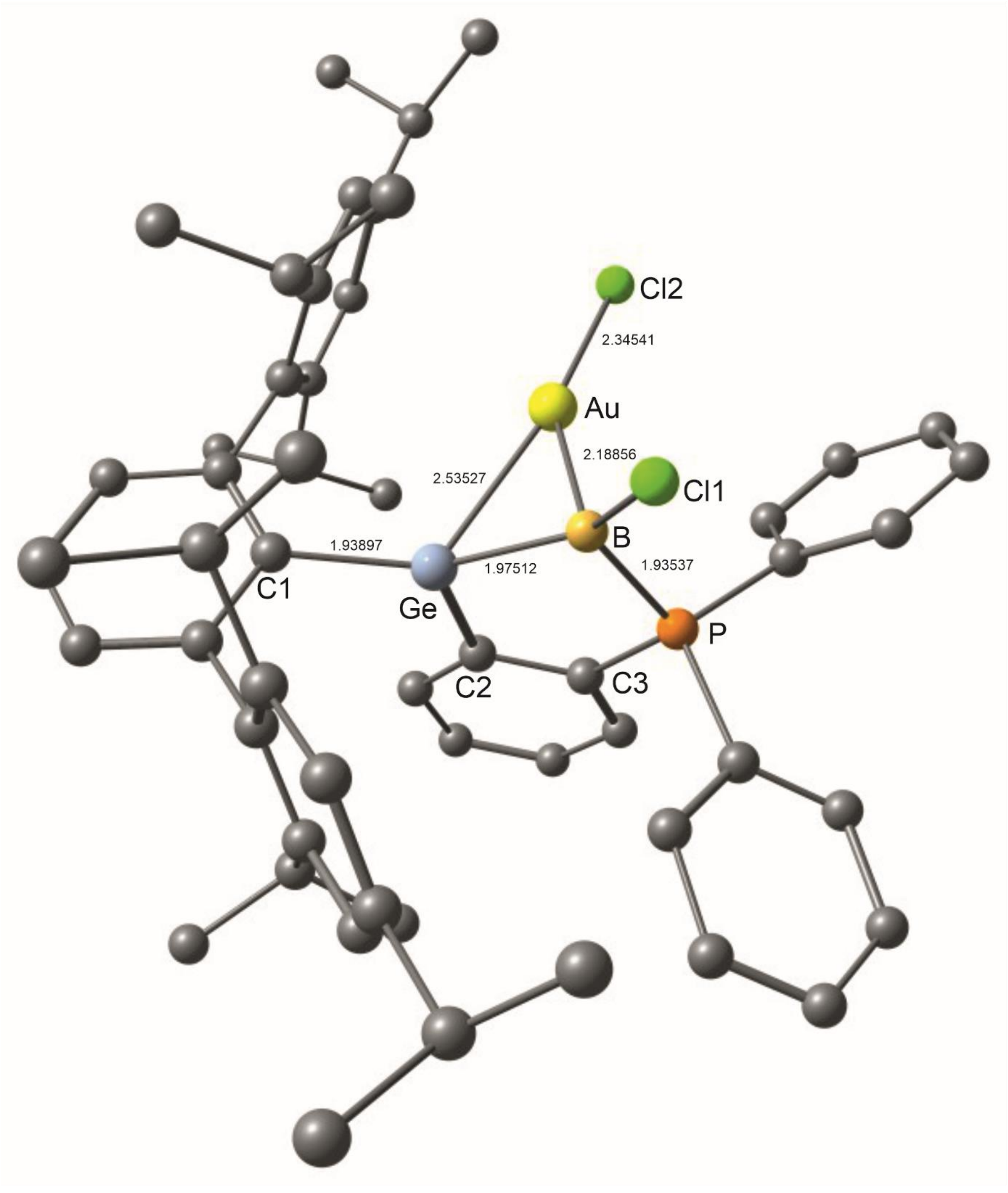

Figure SI72. Optimized structure of compound **12**.

Coordinates of compound **12**.

123

Coordinates from ORCA-job bp86optfreq_hnumac

Au 3.44791209679037 8.69748748709887 13.14984482669344

Ge 2.41332693376395 6.78123102586069 11.85170321260872

P 4.61900643007594 5.85758785288506 13.68741017905733

Cl 1.93707002044885 6.47016256260548 15.30383758230984

Cl 4.62039610285712 10.67824708193703 13.60018296995513

C 3.40840315184911 2.98310131651637 9.87343478315201

H	3.85098105713629	3.44483127641654	10.77803730615645
H	4.12788932351617	3.11784475989392	9.04135536353017
H	3.31700125544046	1.89245493823181	10.05686625762340
C	2.05443114600248	3.61896648681914	9.52060659805927
H	2.24308850228039	4.67511050659563	9.23772557165458
C	6.36972432761749	5.39947101808356	11.49190764731064
H	7.12999077949664	5.08010182070694	12.22174424431887
C	2.49356832783793	12.12583393162348	11.41409020145763
C	3.08591579228042	11.49377070444586	10.30920911716386
H	3.95355584107602	11.97453860072440	9.82930732942405
C	4.71107677996751	1.33717713364777	14.65666794863933
H	4.73376356643085	0.25261333240128	14.84291518496889
C	2.46683735030144	14.59084519470966	10.93144168678963
H	2.72083599035221	14.38807621528310	9.87117098618674
H	2.88483222985979	15.58282062724211	11.20292267384232
H	1.36042781468091	14.65442322678376	11.00291777912585
C	6.64610650276186	5.35739062938094	10.11295232838950
H	7.62861097512184	5.00775168257902	9.76211106909772
C	1.41064775272409	2.92998314018516	8.30159481429725
H	1.16481337476272	1.87189547039575	8.53088155842649
H	2.09894935756147	2.94116116925660	7.43094227514988
H	0.47160692765090	3.43654190699621	8.00269796356853
C	-0.61905901291586	7.36360682728552	8.28086170903499
H	-1.26743600965814	7.45970691510962	7.39643209327335
C	0.97577591540095	2.52348174700750	11.54521679379872
H	1.60717820030034	1.64165331510184	11.35403950632127
C	1.08627725450772	3.64306836127921	10.69701256038090
C	5.87039141336347	6.73188390250350	14.66873365980965
C	0.07195130279912	8.48537377087424	8.76374066928626
H	-0.05214508576320	9.46628062909528	8.28009433241837
C	5.11099669325574	5.84627336030458	11.92320911163875
C	3.58182238015203	1.92256246974406	14.05915172546189
H	2.72356191260452	1.30004632694604	13.77139469001718
C	-0.53178864437050	6.13295386957788	8.95265393050725
H	-1.13166604028369	5.27205421551855	8.62049709142989
C	4.87261368753127	9.42220295837572	9.01701481762078
H	5.38349792202395	10.40557956257437	9.06849796551706
H	5.39207599617698	8.81360002044390	8.24845140114157
H	5.00420635768198	8.92607496460176	9.99884401652614
C	4.64982718301897	4.10054254704149	14.17524309621839
C	4.11099343928464	6.24627419719855	11.00244377486883
C	3.00589393454529	13.48721846118228	11.86549859006599
H	4.11185835037195	13.45106726329473	11.75045057695862
C	0.26117111820691	4.77104530993247	10.94295414702001
C	5.81809894390547	6.61592025811412	16.07531685996171
H	5.11458751263173	5.91284467170936	16.54673769340443
C	5.78605248396641	3.51670509140284	14.77156881538598

H	6.64849132363809	4.14322171707898	15.04454020347628
C	5.66576190131146	5.75545836598226	9.18864487208654
H	5.88484089809058	5.72122334185410	8.11026468799924
C	3.54036122938877	3.30432034363354	13.82352748358809
H	2.65254571578381	3.76072900413278	13.35836583576750
C	3.17417740526019	10.32358262826698	7.34366157619830
H	2.09957009555056	10.38240502711722	7.07712562153442
H	3.70606757263065	9.80943310273773	6.51587735235098
H	3.56139904570884	11.36233363936175	7.40083822832906
C	5.81028410283620	2.13474878128977	15.01682841733055
H	6.69510520788931	1.67908457005280	15.48679866684500
C	4.40577746825538	6.19739378161422	9.63063892109307
H	3.64589816266194	6.51119405880937	8.89838827494389
C	1.53751049802235	9.62331584621716	10.46354374793507
C	3.37933232391976	9.58324559487270	8.67703993364344
H	2.94911938850732	8.56625688111764	8.56053518658762
C	-1.56371347503438	5.95085490692777	12.32274335298234
H	-1.14581799394650	6.83167906775048	11.79730932022069
C	-0.63455330436376	4.77188767360906	12.05167419102263
C	2.63542406507279	10.25492320501944	9.82492121829665
C	-2.95395756727348	5.68451600639711	11.71341191742455
H	-3.42923793373355	4.79791328970907	12.18344829939452
H	-2.88464026041682	5.49283210517000	10.62401382981187
H	-3.62372382358070	6.55602078623247	11.86600013280964
C	2.70746267013095	13.80711806833461	13.33543585622988
H	1.61922429536171	13.94124548428328	13.51287504397542
H	3.20340707562687	14.75386109387257	13.63197050562476
H	3.07653322783426	12.99627802901349	13.99399706466600
C	7.55648996961687	8.47071795887688	14.86593684522452
H	8.21344597452069	9.21474694333770	14.39265058840199
C	0.09002086969693	2.49680910210411	12.63535022994354
C	0.91925733562955	8.38540017289608	9.88990366903850
C	0.29784199698257	6.00136212132158	10.08314494482475
C	1.44409105967343	11.45806859422515	12.06533083151841
H	1.00711180859628	11.90385796613576	12.96753236120393
C	-1.65689068035378	6.31275675262673	13.81246801968468
H	-2.16205063567855	5.52042794423515	14.40334699208486
H	-2.25070551693850	7.24005311388653	13.94289164540597
H	-0.65324183793820	6.47679873620300	14.25275088058774
C	-0.69599932929175	3.63702146925390	12.87575542209834
H	-1.38797750918483	3.64243904426114	13.73069842057135
C	7.51969286899232	8.34498881394647	16.26396968991450
H	8.16082040973480	8.98399720525768	16.88987193176799
B	2.86917442544932	6.68025526726501	13.77084406669969
C	6.73318516563081	7.66809908182048	14.06399356113485
H	6.73618777557817	7.79083691237588	12.97146050331615
C	1.06130805361874	7.11981524824713	10.50373990159612

C 6.65033189334666 7.41943080121018 16.86744102351917

H 6.60881766275775 7.33242085066602 17.96365996512154

C -0.09766165619297 9.77399689154812 13.91575564097474

H 0.90523723177800 9.52460474230528 14.31991770288368

H -0.84154762783053 9.14228612245996 14.43954465335448

H -0.32047009638764 10.82936672647132 14.17488685032296

C -1.53562941271278 9.87775833033914 11.81486292538125

H -1.72753882548281 10.96760813736833 11.90202515350374

H -2.34689136310313 9.34186942611356 12.35065637574625

H -1.59742370232502 9.60715836688943 10.74174993038657

C -0.15807845595693 9.51991506009210 12.40275588146335

H -0.02323894667318 8.42795532445752 12.26380448442123

C 0.01977686850271 1.57828955744779 15.01064937265733

H 0.92702755210260 2.16256587550523 15.26293775516642

H 0.02653962680567 0.64670899042097 15.61296819594744

H -0.85887243278364 2.17260663822170 15.33580238745492

C -0.04900823164912 1.25597174731988 13.50751055861646

H 0.80597817335267 0.58731114309415 13.25803318752500

C 0.96493640643089 10.21036762402824 11.63130672605053

C -1.34119039865166 0.49123910838143 13.15700721566062

H -2.23477017928859 1.11434135910021 13.37012743410429

H -1.42738221345254 -0.44178850999569 13.75195789671375

H -1.36971731143753 0.22213572229351 12.08207734881048

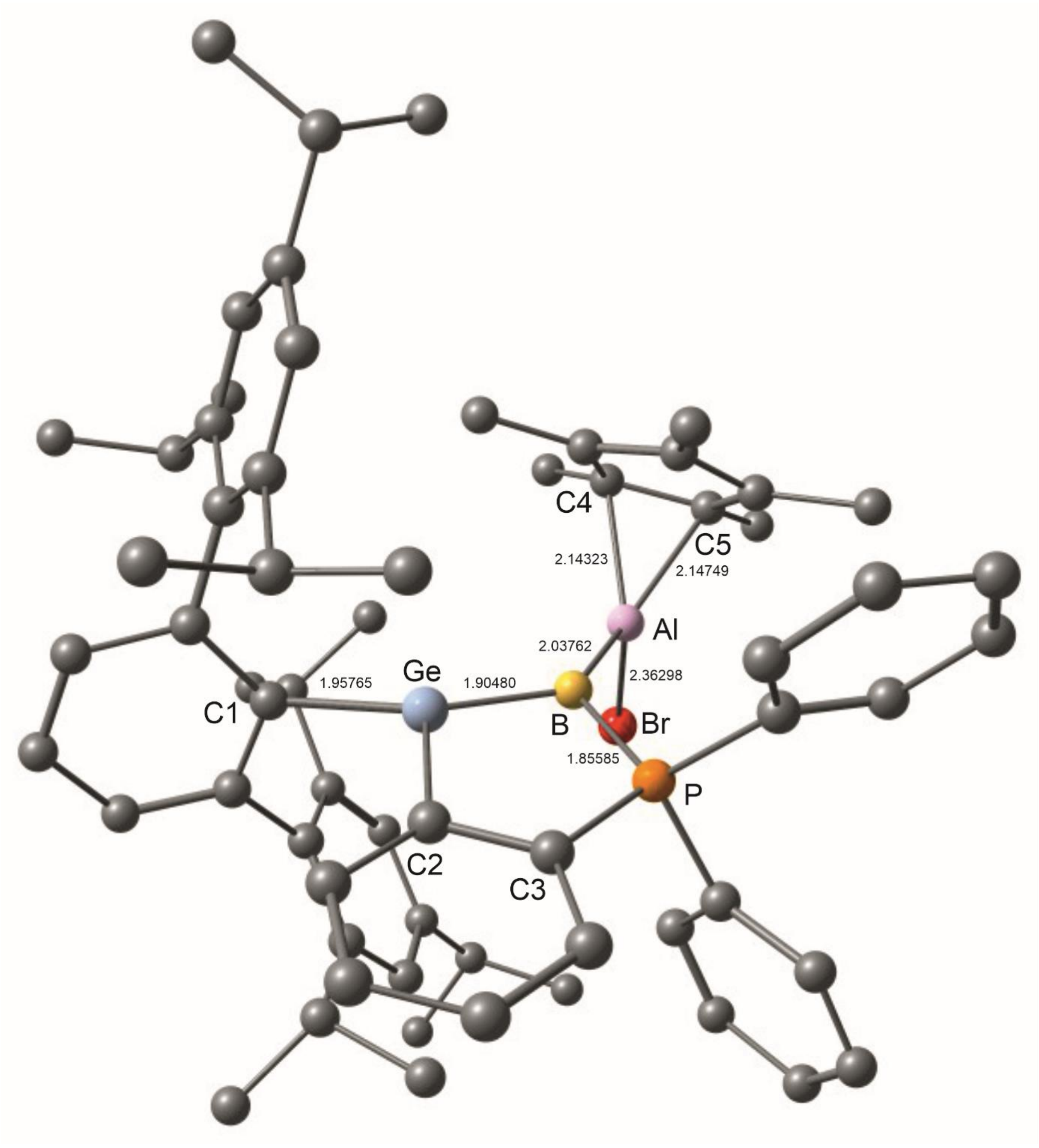

Figure SI73. Optimized structure of compound **13**.

Coordinates of compound **13**.

147

Coordinates from ORCA-job bp86optfreq_hnumac

C 17.68862734135121 12.09775570485812 -0.44813337892083

H 17.44805552379160 11.20239032228177 -1.04103993756095

C 18.34319808766499 13.18455878768072 -1.04677958341872

H 18.63512239881710 13.13427849045355 -2.10700736425806

C 18.56853014782171 14.35607361520444 -0.30895862327684

H 18.98773151622339 15.25060432150732 -0.79407623737638

C 18.24234522035087 14.41029496351451 1.06173182661887

C 18.31494254623474 15.71717473505107 1.80085150516379

C 19.52264812648763 16.24396150217270 2.33116621680842

C	19.49888184414727	17.49783587209183	2.97092456008696
H	20.44016223640575	17.90098375901511	3.37628030728754
C	18.31748187728107	18.25182978537743	3.08934816836098
C	17.68397641179012	13.26211007484915	1.68974781281397
Br	15.99251802603836	16.88658126112641	6.04237880842903
C	17.13604315152459	17.70688631056566	2.56585879380583
H	16.20277391985496	18.28210589144325	2.66756112781600
C	17.10831622823072	16.46146728928633	1.91811592984215
C	20.84207231944216	15.49943043432827	2.17480160150953
H	20.59342273197126	14.46073505694992	1.88085609430151
C	21.68256328191967	16.10764028986581	1.03569374685498
H	22.62440065962628	15.53774659165340	0.89220574844878
H	21.95184193898606	17.16115203204575	1.26024869966197
H	21.12829461110979	16.10231241412535	0.07572172577945
C	21.64403675642944	15.42568959139033	3.48309198061210
H	22.51448221445263	14.74861350470399	3.36780467983787
H	21.02339611253335	15.03867291276100	4.31259539273240
H	22.03089071697890	16.41983440032949	3.78932576379407
C	18.30291307513773	19.64413483789495	3.70529502114645
H	17.23729972341174	19.95869503835207	3.75010875975548
C	19.05074427469928	20.64707525802715	2.80531662080750
H	18.62893128198297	20.65852657687235	1.78008883566970
H	20.12520825635162	20.37950615629838	2.72178711891441
H	18.98986171285065	21.67615002365340	3.21733439677746
C	18.84732583831940	19.66460977930873	5.14141204657002
H	19.92603716465338	19.40210884800508	5.16046549528391
H	18.29821460647550	18.94476492737099	5.78068598462941
H	18.74658796594049	20.67526328785479	5.58853470839264
C	15.78008481419493	15.93518508040201	1.38584926751190
H	15.99762031226873	15.07790001753123	0.71786391341071
C	15.01630892536163	16.97273698027911	0.54600755365888
H	15.64473773753777	17.36270210707980	-0.28003440621453
H	14.68331452924930	17.83777595108395	1.15585391336626
H	14.10773472629089	16.51556948970399	0.10218439440672
Ge	17.96923474650515	13.15866517007733	3.62373929563192
P	19.05324260602270	13.16180786988431	6.29761005658367
Al	15.83114647502317	14.53185758646220	6.15608488011458
C	17.31047189740162	12.13244348127612	0.91237905165809
B	17.48066083805869	13.59488646102441	5.41238588528486
C	14.92090277785352	15.41342044483505	2.54759923372298
H	13.95122427202616	15.02338898027199	2.18142604216869
H	14.72208110237137	16.21485507758310	3.28633298715153
H	15.44156121170075	14.59572886981280	3.08675356000683
C	16.41237245691788	11.04816213990682	1.44407274250573
C	15.01295254600600	11.13988666840504	1.17579984150169
C	14.16501931429798	10.11741316477329	1.63512543267571
H	13.08673765242620	10.19272570272576	1.42981354813079

C	14.64478635968706	9.01402765636186	2.36386351456856
C	16.02208959169325	8.94606525605342	2.61742231036184
H	16.41588790743452	8.08480592277149	3.18033204011289
C	16.91563940817525	9.93442704653188	2.16145576362444
C	14.42421276624086	12.29911989811478	0.37020217099280
H	15.10954977930092	13.16345212989286	0.49324999260172
C	13.02886730672690	12.73774928873314	0.84713782823354
H	12.73014296169684	13.67697980961741	0.33891287752586
H	12.99859899317221	12.91225682902101	1.93934756647992
H	12.25373350136807	11.98087164899859	0.60623661451486
C	14.36651294564580	11.95489711997537	-1.13239369694903
H	13.95541408010378	12.80563711447912	-1.71479640024304
H	13.71241178390227	11.07400635223532	-1.30215613157343
H	15.36654314966422	11.71690314686313	-1.53936668275907
C	13.70206805744765	7.93456023586631	2.87920222238971
H	14.33940413685824	7.14313907184503	3.33213078324270
C	12.78016967038429	8.47746908876241	3.98758759335056
H	12.11396423106888	9.27495165422183	3.59650566551577
H	13.36689158971411	8.91167040852141	4.82153659451323
H	12.13539319813793	7.67321261389071	4.39894631103085
C	12.89149580574631	7.28705979370154	1.74198378154773
H	12.21238237602673	8.02223193568179	1.26178505302233
H	12.26301658240069	6.45706770928856	2.12622513911512
H	13.55729421410982	6.88119239627709	0.95392498267812
C	18.40838837198478	9.75675972391409	2.38344567405770
H	18.88182638444658	10.73219854646367	2.17199471282003
C	19.00014631543701	8.75409307740562	1.37605631698373
H	18.79883203755497	9.07111392477593	0.33282745185192
H	18.56134730307042	7.74321656884259	1.51175843273394
H	20.09960791045836	8.67195102235648	1.50535387028069
C	18.75677130708063	9.38693328810185	3.83174122347883
H	18.43429990218817	8.35671707519507	4.08958819241828
H	18.26353812565772	10.08700248467841	4.53551223928216
H	19.85235310857761	9.44995785872867	3.99390423065076
C	19.83676984884390	12.52489205393982	3.76902835425146
C	20.27213448431775	12.49215501284988	5.11120564397102
C	21.55743970875577	12.05110681371170	5.45874600324395
H	21.88173850853338	12.03412651537757	6.50993535133076
C	22.43651203193570	11.63104862458039	4.44227482688558
H	23.44707193653871	11.28044092206733	4.70074706546840
C	22.02475745534187	11.67596020866381	3.09843511610531
H	22.71855290207192	11.36191566630247	2.30296066450185
C	20.73348953486857	12.12248119848390	2.76178061693784
H	20.42460310640724	12.15604352446856	1.70475739532673
C	18.95016608928931	11.93768369002360	7.64211554545060
C	18.57019534470535	12.34389829461461	8.93722554937783
H	18.39768719968253	13.40975398882631	9.14789829723980

C	18.41752564512659	11.39296493323255	9.95800395653388
H	18.12639522465733	11.71932209980017	10.96804418199657
C	18.63115205656948	10.02983993529107	9.69133247776854
H	18.50862418051934	9.28545550898601	10.49272966967266
C	19.00727163796363	9.62107435477247	8.39963127082154
H	19.17930647174413	8.55547391377616	8.18470528692425
C	19.17067683370680	10.56950888855106	7.37994362042246
H	19.47062469376487	10.25064617772193	6.37254097020038
C	19.91931114845984	14.57529390168436	7.05698706234790
C	20.92154293268008	14.37979196617997	8.03236904608053
H	21.12990701792179	13.36952313961285	8.41433062187557
C	21.64409739070557	15.47461914131362	8.52619633228179
H	22.42445941339989	15.31795634515944	9.28668337492396
C	21.36686410301644	16.76865229787855	8.05088706633514
H	21.92985904577266	17.63065218518057	8.44075583365295
C	20.36779654580367	16.96304551671303	7.08416411495429
H	20.14303003500300	17.97229071690650	6.71631454597671
C	19.64227341144334	15.87145379959831	6.58067001220880
H	18.85887001423714	16.01828786605008	5.82023898361494
C	13.76086548282604	13.99774212467486	6.00751067353634
C	12.80479330764818	14.81323123009328	5.18114127954389
H	12.80975014772197	14.49200109178639	4.12317059128271
H	13.05082347641165	15.89412931710753	5.20632328084229
H	11.76544216174468	14.69910723721224	5.55886103884877
C	14.09842444531117	14.27975309413658	7.39940688453323
C	13.54619057265065	15.41215682490724	8.21737956203680
H	13.44958667685740	16.33860184200118	7.61927801472461
H	14.19560500125032	15.64770202591767	9.08317417640225
H	12.53900732015206	15.15558655881491	8.61479111093039
C	14.82040570010724	13.13791303569367	7.90605421682673
C	15.25826541224840	12.99168128921043	9.33520741605113
H	15.88480226078627	12.09296339508514	9.47598518152482
H	14.37835271339269	12.89686790284495	10.00826300103152
H	15.84021990773316	13.86639399091688	9.69216264883068
C	14.91701261719216	12.16140425683522	6.86567109504505
C	15.67729352705867	10.86944464794138	6.89975198180263
H	16.45917756856081	10.85737565628306	6.10936382142307
H	15.01488276723238	9.99648868050847	6.71995146847031
H	16.18783564404687	10.71193869821168	7.86851078028853
C	14.28591285498221	12.68865814388706	5.70122490508270
C	14.25564896037079	12.01844775727217	4.36346713210386
H	14.29694229331682	12.75257830702801	3.53829697617857
H	13.33522197691435	11.41563109407524	4.21740005478687
H	15.11754629771315	11.33672000566604	4.22691806375002

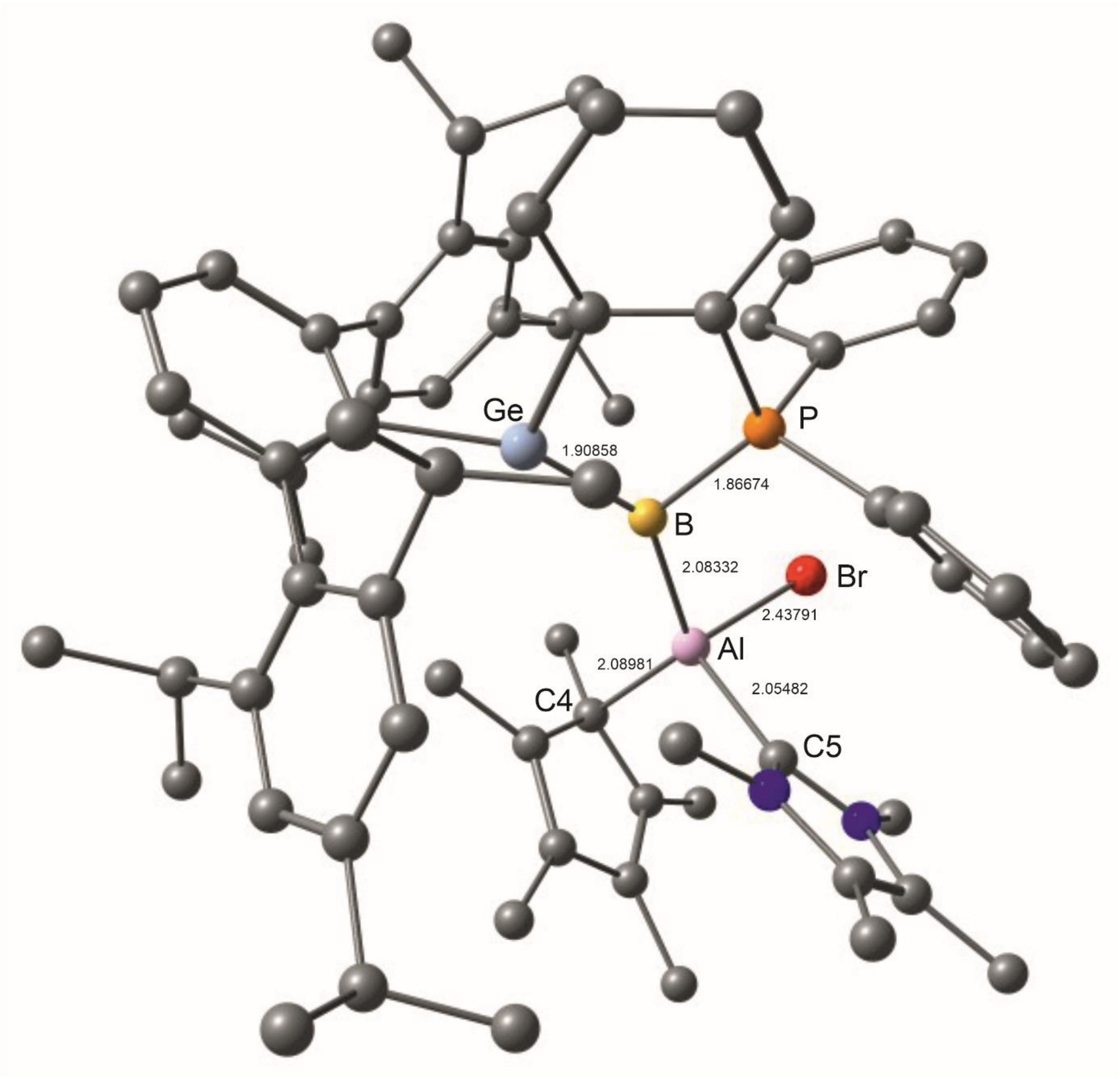

Figure SI74. Optimized structure of compound **14**.

Coordinates of compound **14**.

168

Coordinates from ORCA-job bp86optfreq_hnumac

P	3.71194727586736	11.24841233081702	6.12698679810001
Al	1.59212312216452	8.64330561371071	5.96006290767154
N	0.43977538579433	9.87996306859948	8.50505993451976
C	0.63026635894563	12.89893175957393	2.84476306815699
N	1.14273985917607	7.89138240900524	8.94584905222643
C	-0.43059015501459	13.78953210250782	3.15505285004850
C	-0.70837441567946	14.84243642942361	2.25430402613191
H	-1.49150510737939	15.57095483006273	2.51431119295855
C	-0.00100361418094	14.97027423555885	1.04954667030127
H	-0.21633997603844	15.81104746194069	0.37265141569537

C	0.94104995057102	13.99591655558179	0.68322737971179
H	1.43995617385413	14.04713224110948	-0.29640406670775
C	1.25779221212411	12.94614917003898	1.56725735362823
C	-1.31692929577797	13.59254247096710	4.35683234844626
C	-2.51771215847621	12.83475334327519	4.20992510686149
C	-3.35986563880483	12.68027550682649	5.32745916060032
H	-4.29022629997868	12.10268310706537	5.22267568956598
C	-3.06380676981080	13.24288803674103	6.58043386633527
C	0.51949974897721	5.07967504971853	5.96200019286780
H	0.21725102676757	4.45204280685366	5.09364088867107
H	0.21132838584817	4.53428416447528	6.87654299832309
H	1.62942870468519	5.12227013400207	5.94687242472500
C	-0.09204795249020	6.44751460275564	5.88572513315101
C	0.27169026812540	7.44010130894925	4.87559621034925
C	-0.66431581977859	8.56274550272582	5.03808330270614
Br	3.63267093685366	7.30942052603764	5.97830616429573
Ge	1.70331138507148	12.02481651305989	4.23932247477206
C	-1.13479272209851	6.98113311272991	6.66484283326952
C	-1.87646398163019	13.98253717743799	6.70253916498302
H	-1.62408951597640	14.44772807495974	7.66860877068074
C	-1.00701022937823	14.17699545789263	5.61279062156813
C	-2.93334294000730	12.23101212620134	2.86705256771016
H	-1.99941105243888	11.92257293628004	2.34964806887642
C	-3.63988857373949	13.27388045171432	1.97529108169342
H	-2.99046370179982	14.13947564376145	1.75338191491931
H	-4.55708172485238	13.65015692448724	2.47497436439926
H	-3.93866908017287	12.81798952854106	1.00858444658720
C	-3.83926216570405	10.99395234956280	2.99579516950692
H	-3.92427640304353	10.48402933505446	2.01488467658344
H	-4.86851852350606	11.27455389231441	3.30260664911836
H	-3.45553061261023	10.26076905152189	3.72924218361150
C	-4.03663785983663	13.09948462111414	7.74425460783015
H	-4.86349995214261	12.44367727040926	7.39347928113200
C	-4.64847674134377	14.46287999173833	8.12026171979645
H	-5.13345642163343	14.93813996073197	7.24416346517263
H	-3.86881386213009	15.16121563248385	8.49026818004739
H	-5.40952521393309	14.35208814342246	8.92059033701732
C	-3.39444508845251	12.41990209767731	8.96597026356289

H -3.02711547714398 11.40643245827053 8.70865659444755

H -4.12501424635930 12.31833241611542 9.79506574656434

H -2.53524927250547 13.01054260691764 9.34716159740411

C 0.20533921446441 15.08066691580416 5.77629070038385

H 0.84240319532736 14.93079935655766 4.88601873136768

C -0.21293655072836 16.56248654993089 5.78021722577750

H 0.67887466541061 17.22062945883712 5.83969285828085

H -0.86785364867134 16.79308246706915 6.64683190112498

H -0.77190453012401 16.82296065264996 4.85888516110696

C 1.05549598534705 14.72393134986820 7.00229630307869

H 1.31998900430739 13.64951175928022 6.98219998800183

H 0.52715396383288 14.93600837930885 7.95564016112604

H 2.00078009073952 15.30412687985114 7.00097281318606

C 2.16290672035816 11.80455120945228 1.19632071457737

C 1.55508647540283 10.55055993094265 0.87132453849274

C 2.37526778719936 9.42095301139044 0.72436221396848

H 1.91096244912260 8.45021614804070 0.50128218802469

C -1.48171646853065 8.28297567574648 6.15112422047537

C -1.76731673869357 6.37547430383411 7.88548266878976

H -2.87432276760909 6.32191902328965 7.79766425762549

H -1.56005656272389 6.97272056701950 8.80385959472932

H -1.40758843482163 5.34335967389123 8.07123125857538

C 0.94931198427506 7.08566223410415 3.56697080472157

H 0.23319506460543 6.62958087612190 2.84738654649317

H 1.77336452139047 6.36458157943588 3.72917367840252

H 1.40063484786949 7.97272882744598 3.07766831263320

C -2.54829586769615 9.16662638748733 6.72944404456842

H -2.54950219839599 10.17311167718173 6.26562267153069

H -2.42966375759570 9.29872272137481 7.82873243736746

H -3.56564052633407 8.73940217344103 6.58346867331955

B 2.21489331229918 10.51419976978328 5.28765339372990

C -0.79553434046255 9.69770512656046 4.07025990419863

H 0.15975239955164 9.89008262455391 3.54579731041625

H -1.09789537626852 10.64256559662835 4.56337180644256

H -1.54715834988220 9.47311853357230 3.28593960083049

C 3.77608214084873 9.49162518718534 0.85240524338728

C 4.35346805517790 10.74895025749399 1.07494280726635

H 5.44979494512119 10.82367990443534 1.13870312269182

C	3.57693206772589	11.91200577463485	1.24591773413982
C	0.05016452899607	10.45425339435300	0.61278859906511
H	-0.45784468873377	11.02937168710972	1.41634397347973
C	-0.50864294713654	9.02584369164935	0.63534488250647
H	-1.61580511467112	9.05207794962603	0.58211158718086
H	-0.22609844962683	8.47882253312440	1.55203594067884
H	-0.15530089718968	8.43649805562013	-0.23662081241096
C	-0.30990451932639	11.12140691377083	-0.73178849103367
H	0.19612775855138	10.59748921258277	-1.56939290390632
H	-0.00804777974567	12.18456487005608	-0.76189729449575
H	-1.40474847164391	11.07469369151447	-0.90828678754383
C	4.63972688807447	8.24384038830866	0.73380093517590
H	5.69134005713342	8.56844891313931	0.90066432181482
C	4.28793377543603	7.20334994344207	1.81278625369999
H	3.26622374005717	6.80074312800659	1.65739826561270
H	4.31965468621516	7.63309628451637	2.83372746117425
H	4.99154918926267	6.34607200883181	1.77901457017439
C	4.56417964221152	7.63678627683738	-0.67989948678670
H	4.84839858514684	8.37816454506753	-1.45386258205729
H	3.53481128120160	7.28975875895555	-0.90883092948838
H	5.23986317551895	6.76114612582711	-0.77216087473961
C	4.26969956144104	13.25727078361018	1.41973524895086
H	3.53812365565731	13.94265166368996	1.89024845353264
C	5.50620698930092	13.20155340813203	2.32930030769079
H	6.34211417469107	12.64320005285550	1.85924839929986
H	5.27765056243080	12.71656415398412	3.29689290573500
H	5.87227676509751	14.22550558672021	2.54376561130418
C	4.62917491700889	13.84435266510965	0.03939259185682
H	5.35348625856506	13.19006216078254	-0.48976608437572
H	5.08929899706657	14.84905614607703	0.14537434794109
H	3.73540222633439	13.94087751198738	-0.60884460783767
C	3.02332010411454	13.43746979729353	4.67746554911075
C	3.13713403031594	14.75665832171041	4.19866215146184
H	2.43797786499007	15.12502550150671	3.43077563074934
C	4.13938500462103	15.60996638849534	4.69657750601130
H	4.21473894631826	16.64186399003072	4.31888710070853
C	5.05240880131267	15.15311531930484	5.66412821600262
H	5.83978076691036	15.82263572230477	6.04230535450387

C 4.96429167728449 13.83018723160502 6.13946240238789

H 5.68736803988090 13.45651382837211 6.88090690163036

C 3.94533739860155 12.99771695782109 5.65417069257017

C 5.29646808627239 10.48339059717525 5.66016449513236

C 5.40083102632661 9.98600176254463 4.34674023446725

H 4.51678231725940 10.01706396932611 3.69068947128004

C 6.61653905069608 9.45154196733652 3.90040993421457

H 6.69467661664485 9.05926795925563 2.87554244673692

C 7.72397485609484 9.39719019158209 4.76450769935252

H 8.67362179739895 8.96294103000018 4.41591796955278

C 7.61741961068754 9.88982896852797 6.07640424870052

H 8.48260728567449 9.84393740766385 6.75564588481520

C 6.40586317835979 10.43901797047274 6.52564677614225

H 6.31965734728134 10.82008408721389 7.55423838512271

C 3.64996890428500 11.19065287663283 7.94648318604126

C 3.29462169711478 12.32618624277508 8.70321068036950

H 3.16940189739274 13.29622145496911 8.20319448350538

C 3.10674641412576 12.22057775421333 10.09147884071895

H 2.83650951555899 13.11559810297022 10.67312220661472

C 3.27910411889921 10.98382724323116 10.73596745743635

H 3.13542033574146 10.90268864485609 11.82434530269724

C 3.64350760701833 9.85207260739326 9.98583518792711

H 3.79139200134857 8.88278836139437 10.48565289812013

C 3.82557089286109 9.94796092289964 8.59906418136718

H 4.10083349674235 9.05747621862068 8.01200611121886

C 1.00579389815939 8.78229088273650 7.92454901249979

C 0.22064522832817 9.68509784417448 9.86724917367467

C 0.66798565843089 8.41072139303555 10.15026210808125

C 0.07434639733984 11.10746467152963 7.81413234010329

H 0.54430846139431 11.10653214809843 6.81103303425047

H 0.45687951980602 11.97814829701770 8.37635510113463

H -1.02082715744633 11.19196244711330 7.70539849950861

C -0.35135518418849 10.73906460691695 10.74863381561621

H 0.31488820282224 11.62725784544499 10.78985653557678

H -0.47079112658175 10.35797439752452 11.77989730127275

H -1.34317400861597 11.08316217549602 10.39264256126256

C 0.69994542222386 7.64699083811220 11.42913511453346

H 0.14044756084435 6.69097415614230 11.35377020648635

H	0.24257017055876	8.24115947469838	12.24208413170706
H	1.73698332213379	7.39713502726792	11.73947045577822
C	1.68242320180406	6.54791220683138	8.82476953641339
H	2.13488639128266	6.43121694703347	7.82555043480871
H	0.88041431302991	5.79805202077336	8.96265820292382
H	2.46933849239783	6.38772360833002	9.58745860406774

References

1. S. R. Bahr and P. Boudjouk, *J. Org. Chem.*, 1992, **57**, 5545-5547.
2. D. Raiser, H. Schubert, H. F. Bettinger and L. Wesemann, *Chem.-Eur. J.*, 2021, **27**, 1981-1983.
3. D. Raiser, C. P. Sindlinger, H. Schubert and L. Wesemann, *Angew. Chem., Int. Ed.*, 2020, **59**, 3151-3155.
4. N. Kuhn and T. Kratz, *Synthesis*, 1993, **1993**, 561-562.
5. I. Krossing, *Chem.-Eur. J.*, 2001, **7**, 490-502.
6. I. Krossing, H. Brands, R. Feuerhake and S. Koenig, *J. Fluorine Chem.*, 2001, **112**, 83-90.
7. C. Ganesamoorthy, S. Loerke, C. Gemel, P. Jerabek, M. Winter, G. Frenking and R. A. Fischer, *Chem. Commun.*, 2013, **49**, 2858-2860.
8. R. K. Harris, E. D. Becker, S. M. C. d. Menezes, R. Goodfellow and P. Granger, *Pure Appl. Chem.*, 2001, **73**, 1795-1818.
9. K. Eichele, WSolids1 ver. 1.21.7, 2021.
10. F. Neese, *Wiley Interdiscip. Rev. Comput. Mol. Sci.*, 2018, **8**, e1327.
11. F. Neese, *Wiley Interdiscip. Rev. Comput. Mol. Sci.*, 2012, **2**, 73-78.
12. F. Neese, *WIREs Comput Mol Sci.*, 2022, **12**, e1606.
13. A. D. Becke, *Phys. Rev. A*, 1988, **38**, 3098-3100.
14. J. P. Perdew and W. Yue, *Phys. Rev. B*, 1986, **33**, 8800-8802.
15. S. Grimme, S. Ehrlich and L. Goerigk, *J. Comput. Chem.*, 2011, **32**, 1456-1465.
16. F. Weigend, *Phys. Chem. Chem. Phys.*, 2006, **8**, 1057-1065.
17. F. Weigend and R. Ahlrichs, *Phys. Chem. Chem. Phys.*, 2005, **7**, 3297-3305.
18. B. Metz, H. Stoll and M. Dolg, *J. Chem. Phys.*, 2000, **113**, 2563-2569.
19. D. Andrae, U. Häussermann, M. Dolg, H. Stoll and H. Preuß, *Theoret. Chim. Acta*, 1990, **77**, 123-141.
20. G. A. Zhurko, *CHEMCRAFT (http://www.chemcraftprog.com)*.
